# Surface boulder banding indicates Martian debris-covered glaciers formed over multiple glaciations

**DOI:** 10.1073/pnas.2015971118

**Published:** 2021-01-18

**Authors:** Joseph S. Levy, Caleb I. Fassett, John W. Holt, Reid Parsons, Will Cipolli, Timothy A. Goudge, Michelle Tebolt, Lily Kuentz, Jessica Johnson, Fairuz Ishraque, Bronson Cvijanovich, Ian Armstrong

**Affiliations:** ^a^Department of Geology, Colgate University, Hamilton, NY 13346;; ^b^NASA Marshall Space Flight Center, Huntsville, AL 35801;; ^c^Lunar and Planetary Laboratory, University of Arizona, Tucson, AZ 85721;; ^d^Department of Geosciences, University of Arizona, Tucson, AZ 85721;; ^e^Earth and Geographic Sciences Department, Fitchburg State University, Fitchburg, MA 01420;; ^f^Department of Geological Sciences, Jackson School of Geosciences, University of Texas at Austin, Austin, TX 78712

**Keywords:** Mars, glaciers, Ice Age, obliquity, climate

## Abstract

Glacial landforms, including lobate debris aprons, are a global water ice reservoir on Mars preserving ice from past periods when high orbital obliquity permitted nonpolar ice accumulation. Numerous studies have noted morphological similarities between lobate debris aprons and terrestrial debris-covered glaciers, an interpretation supported by radar observations. On Earth and Mars, these landforms consist of a core of flowing ice covered by a rocky lag. Terrestrial debris-covered glaciers advance in response to climate forcing driven by obliquity-paced changes to ice mass balance. However, on Mars, it is not known whether glacial landforms emplaced over the past 300 to 800 formed during a single, long deposition event or during multiple glaciations. Here, we show that boulders atop 45 lobate debris aprons exhibit no evidence of monotonic comminution but are clustered into bands that become more numerous with increasing latitude, debris apron length, and pole-facing flow orientation. Boulder bands are prominent at glacier headwalls, consistent with debris accumulation during the current Martian interglacial. Terrestrial glacier boulder bands occur near flow discontinuities caused by obliquity-driven hiatuses in ice accumulation, forming internal debris layers. By analogy, we suggest that Martian lobate debris aprons experienced multiple cycles of ice deposition, followed by ice destabilization in the accumulation zone, leading to boulder-dominated lenses and subsequent ice deposition and continued flow. Correlation between latitude and boulder clustering suggests that ice mass-balance works across global scales on Mars. Lobate debris aprons may preserve ice spanning multiple glacial/interglacial cycles, extending Mars climate records back hundreds of millions of years.

## Significance

Significant debate exists whether the global population of Martian debris-covered glacier deposits formed continuously over the past 300 to 800 Ma, or whether they formed during punctuated episodes of ice accumulation during obliquity maxima (lasting ∼10–100 ka). We show that, like ancient debris-covered glaciers on Earth, boulder banding on Martian glacial deposits indicates multiple episodes of ice accumulation and advance. In our analysis, glacial periods are followed by ice removal from the glacier accumulation zone, forming debris bands. We report a median of five to six glacial/interglacial transitions recorded on Martian debris-covered glaciers, suggesting the cadence of glaciation on Mars is set by orbital forcing over tens to hundreds of Ma, not individual ∼120 ka obliquity cycles.

Debris-covered glacial landforms, including lobate debris aprons (LDAs), are widespread on Mars between ∼30 and 60° latitude (1, 2) and formed during the Amazonian period (3). Like Earth, Mars experiences glaciation and interglacial climate conditions. Midlatitude glaciation occurs on Mars when moderately high orbital obliquity (25 to 35°) mobilizes low-latitude ice deposits as vapor into a dust-rich atmosphere, driving topographically forced precipitation and positive ice mass-balance at midlatitudes (4). Interglacial conditions persist when low orbital obliquity drives surface ice stability poleward, preventing accumulation of ice at LDA sites and causing sublimation of LDA ice and the formation of surficial lag deposits that impede further mass loss from within LDA (1, 4–7).

While nearly all Martian debris-covered glacial landforms date to the recent Amazonian period (the last 300 to 800 Ma) (3), the chaotic evolution of the obliquity of Mars’ orbit beyond the last 20 Ma (8) makes it impossible to determine a priori whether nonpolar ice was deposited quasicontinuously throughout the entire recent Amazonian “glacial” period during every ∼120 ka obliquity maximum (9) or whether glacial accumulation and flow was highly punctuated, occurring only during episodic, short-lived obliquity optima when midlatitude ice deposition could occur due to the presence of low-latitude ice reservoirs (4). In particular, numerical models of ice sheet flow and debris cover generation strongly disagree on the timescales required for glacial advance to generate the observed extent of LDA. Experimental rheology-based models predict flow timescales of ∼100 to 300 Ma to form LDA, although the lack of a uniform best-fit rheology for all Martian LDA suggests that spatial heterogeneity exists at global scales in the number of ice advance events, the flow history, or ice properties (10, 11). In contrast, models that assume an initial global ice sheet and ice rheology constrained by Glen’s Flow Law predict much shorter LDA growth timescales on the order of 400 to 500 ka (12). Direct orbital observation of boulder size and distribution on LDAs, the largest and most volumetrically significant debris-covered glacier group on Mars (1, 2), make it possible to distinguish between these two endmember models.

We make use of supraglacial debris observed in High Resolution Imaging Science Experiment (HiRISE) observations of LDA. Supraglacial rocky debris on LDAs is thought to result partially from sublimation of LDA ice that releases fine sediments (<∼25 cm) that were entrained in the LDA accumulation zone (e.g., ref. 13) and partially from rafting of large (>1 m) rockfall that travels supraglacially, in cases, forming longitudinal rocky zones analogous to medial moraines (14). Both mechanisms generate debris layers atop terrestrial debris-covered glaciers (15), as does the delivery of rolling boulders from upslope scarps which bypass the accumulation zone (16).

If ice accumulation occurred in a single, long episode, boulders derived from headwall erosion and entrained as surficial debris deposits (14, 17) should be randomly distributed on the surface and might be expected to show evidence of boulder comminution over tens to hundreds of millions of years of exposure. A single, long period of deposition predicts that boulder populations should be smaller at the toe of LDAs than at the headwall.

If, however, the ice accumulation and flow was discontinuous over time, boulder distributions should be analogous to patterns produced on terrestrial debris-covered glaciers where surface debris is unevenly distributed. Debris distribution is controlled by changes in precipitation and surface energy balance paced by climatic cycles (16). At insolation minima, ice accumulates along headwall scarps, resulting in debris-poor layers (18). In contrast, during insolation maxima (19), boulder-rich debris concentrates in headwall regions as rockfall accumulates on ice-free surfaces (18). Debris-rich layers inclined relative to the surface topography are then advected down-glacier during the next period of positive mass-balance as ice accumulates in the headwall region, trapping boulders as an englacial debris band and deforming it into an inclined layer through cold-based glacial flow dominated by internal deformation of the ice (18). These debris-rich bands outcrop at the glacier surface as bands of enhanced boulder concentration. Generally, these bands are located downslope from arcuate surface discontinuities where younger and older ice are in contact (19). Breakdown and erosion of boulders within the interior of debris-covered, cold-based glaciers is close to absent (15, 20, 21). Accordingly, episodic accumulation and flow of martian debris-covered glaciers should result in clusters of boulders where accumulation-paced internal debris layers intersect the glacier surface, and little to no comminution of boulders because englacial transport dominates clast transit through the glacier until sublimation and deflation of fines brings the boulders to the surface.

To test these two endmember scenarios, we mapped boulders along 100-m-wide centerline swaths of 45 LDAs located across the Martian surface (5 in the southern hemisphere, 40 in the northern hemisphere) using full-resolution, 25 cm/pixel HiRISE image data. For 18 of the sites for which overlapping HiRISE stereo acquisitions were available, we measured boulder position and boulder width normal to the solar azimuth (after 22); for the remainder, only boulder position was mapped.

Spatial clusters of boulders were identified using a K-means clustering approach (23) (see boulder_analysis.R in code repository). K-means clustering is an unsupervised algorithm that groups boulders based on their location without grouping boundaries defined a priori. The algorithm starts with randomly selected points that center a number of groups, k, and iteratively refits to optimize the position of these group center points. We selected k in a manner informed by the Bayesian Information Criterion (BIC) based on the fit of a Gaussian mixture model (24), which balances the fit of the model with a penalty on complexity and is aimed at keeping the number of clusters at a minimum without sacrificing fit. We fit the boulder position data with K-means clustering models for a sequence of k values and assess the BIC values for the collection of models. The model with the largest BIC is selected as the corresponding value of k as the nominal number of boulder bands at each site.

K-means clustering analysis is sensitive to boulder bands at all spatial scales, but only in one dimension (down-profile) and so a second banding parameter, preferred number of bands, was determined subjectively by examining each LDA for natural breaks in boulder density that might reflect longer-range clustering or boulder bands that do not completely cross the survey transect. These breaks are apparent in kernel density analysis (boulders per square meter, see *SI Appendix*) as discontinuities in spatial boulder density at the hundreds of meters to kilometer scale. These break points are commonly reflected in the K-means clustering report as rollover points in the BIC plot, above which adding additional cluster centroids (bands) produced only a small marginal improvement in BIC score.

**Fig. 1.**
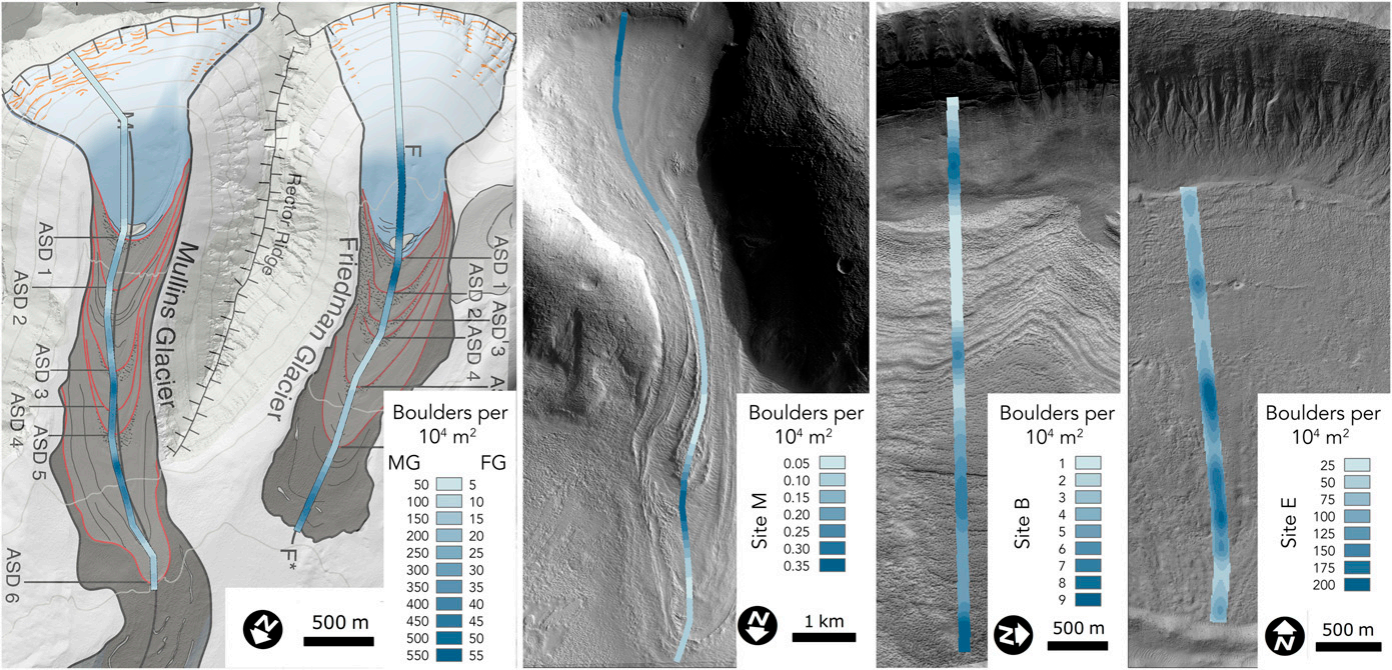
Comparison of surface boulder densities for Mullins and Friedman glaciers, Earth, and three sites on Mars. All Martian lobate debris aprons are oriented with downslope to image bottom. Color coding shows kernel densities of boulders. Boulders are clustered at all sites, and on Earth, boulder bands align with arcuate surface discontinuities (labeled as ASDs) mapped by ref. 19. Kernel density plots for all sites are available in the *SI Appendix*.

## Results

**Fig. 2.**
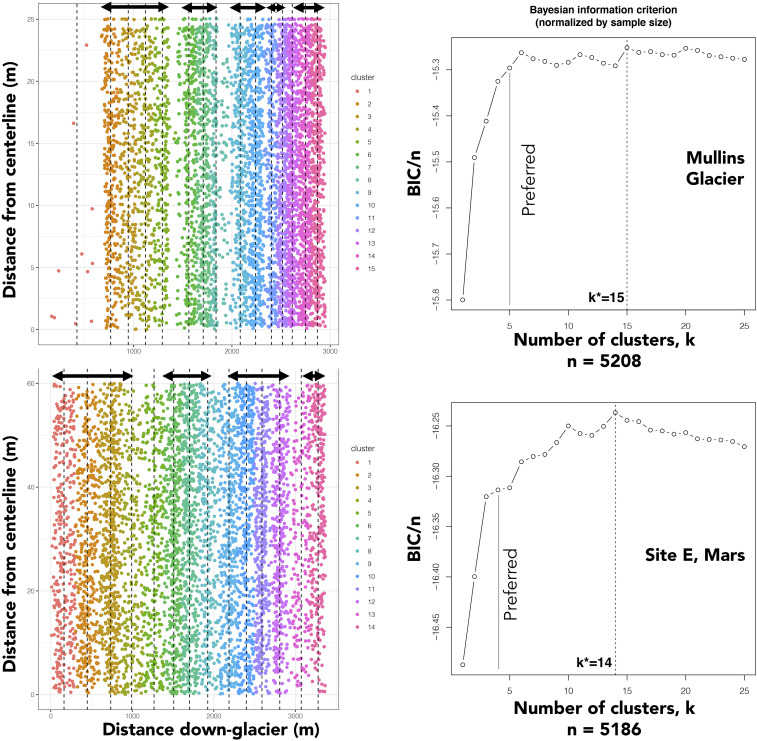
Comparison of boulder clustering and K-means clustering analysis for Mullins glacier and the site E LDA, Mars. *Left* plots show boulder distribution with distance down each landform (one dot per boulder), with clusters color-coded. Arrows show natural breaks in density from which preferred numbers of bands were determined. *Right* plots show BIC score and k*, the nominal number of bands per site. Preferred band numbers typically coincide with inflection points in BIC/*n* in K-means analysis shown on the right.

As on terrestrial debris-covered glaciers, boulder bands are apparent upon visual inspection of Martian LDA (Fig. 2). On Earth and Mars, the number of bands at each site was quantified using a K-means clustering approach (23) based on manual mapping of boulder position (Fig. 1 and *SI Appendix*). Boulder bands are common across all LDAs, and range in abundance from 2 to 22 bands, with a median of 6 across all sites.

The preferred number of bands highlights only long-range clustering that may aggregate several small clusters. For example, while K-means clustering finds 15 as the nominal number of clusters for Mullins glacier on Earth, long-range clustering can also be seen in the form of the arcuate surface discontinuities mapped by ref. 19, which manifest as breaks in boulder density (Fig. 1), producing a preferred cluster number of 5. In the Earth case, boulder clusters emerge immediately upslope or downslope from internal debris layers that are expressed on the surface as arcuate surface discontinuities. Across Mars, preferred boulder band numbers range from 2 to 23 bands per LDA, with a median of 5.

**Fig. 3.**
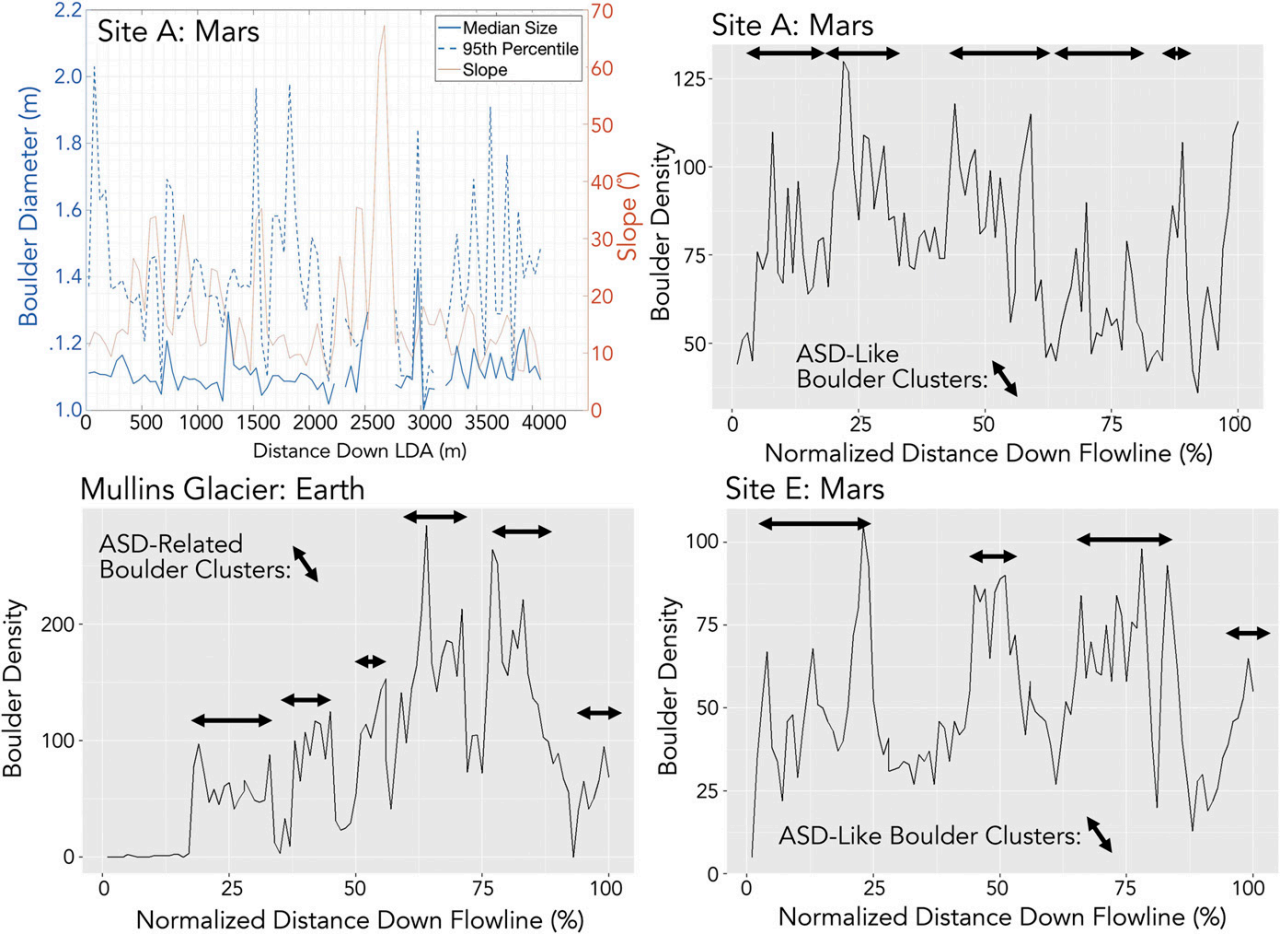
Boulder size distribution and boulder spatial density at LDA sites A, E, and Mullins Glacier. Boulders are binned into 50-m segments down-glacier to compute median and 95th percentile boulder size. The mean surface slope in each bin is derived from an underlying HiRISE stereo elevation model produced for the site using MOLA-tied Ames Stereo Pipeline methods described in ref. 35. All boulder size data are available in the *SI Appendix*. All sites show boulder bands consistent with ASD-like clustering. Arrows show the width of the ASD-like clusters (in the Mars case) and the width of the ASD-related boulder clusters on Earth.

While boulders are strongly clustered, their size distribution along LDA profiles shows little to no structure. Boulder size does not decrease or increase monotonically down LDA centerlines (Fig. 3). In addition, where boulders were mapped in multiple centerline transects or where whole-LDA counts have been made, boulder bands are found to be concentric across LDA, not longitudinally or obliquely oriented, suggesting boulders were not emplaced as ejecta rays (see *SI Appendix* for noncorrelation between bands and proximal craters). Likewise, boulder banding is not more common on LDA with steep upslope bedrock scarps, suggesting that most boulders are delivered to the glacier accumulation zone in a manner that is not dependent on the slope of the bounding scarps. The direct delivery of rocks to LDA surfaces is not a dominant transport process.

**Fig. 4.**
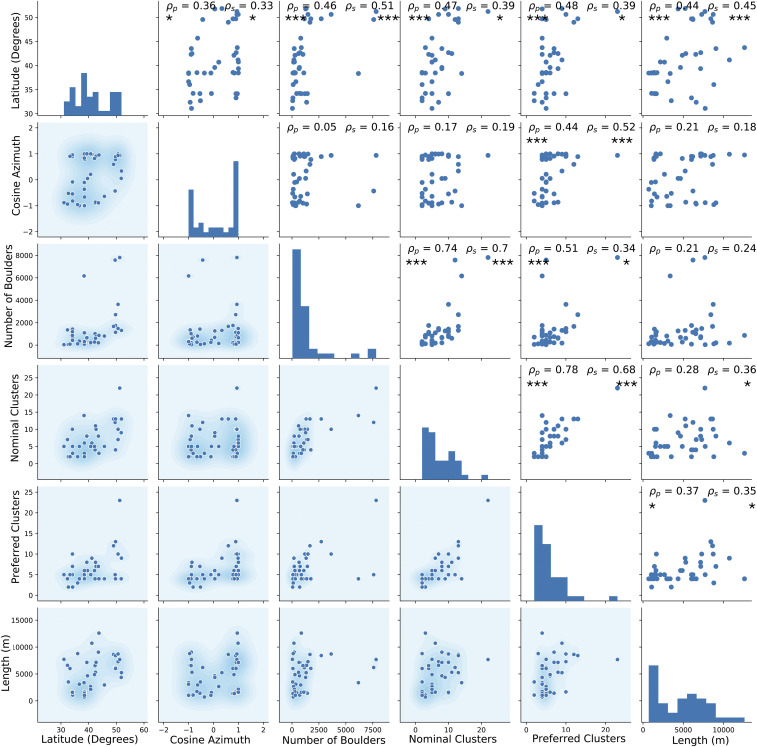
The spearman correlation (ρs) and Pearson correlation (ρρ) matrix for Martian LDA. Boulder banding on LDA increases with latitude and LDA length for both nominal and preferred band number. Significance levels: *P < 0.05, ***P < 0.005. The lower diagonal section of the matrix scatterplots include kernel density of the data in the background. Histograms in the diagonal each have a plot-specific vertical scale and uniform bin sizes of 10 for all ordinate parameters.

Boulder clustering on Martian LDA shows global geographic patterns (Fig. 4). The number of boulder bands (both nominal and preferred) is positively correlated with latitude and LDA length. The preferred number of boulder bands also increases with increasingly pole-facing LDA flow direction (cosine of the downslope direction azimuth for the LDA). Taken as a group, pole-facing LDAs have more boulder bands than equator-facing LDAs: nominal average 8.4 bands for pole-facing LDAs and 5.6 for equator-facing (*P* = 0.046), while preferred band number averages 7.5 for pole-facing and 4.9 for equator-facing (*P* = 0.013). Testing for statistical differences between group means was determined via one-way ANOVA.

## Discussion

Across Mars, two-thirds of mapped sites have a boulder cluster within the first 10% of the centerline, in the vicinity of the bedrock headwall immediately upslope of the LDA. Of sites that lack a headwall boulder cluster, 11 of 15 show evidence of younger Amazonian mantling sediments and ice (14, 25) overprinting LDA headwalls, which could mask some boulder concentrations located in LDA accumulation zones. In addition to headwall-vicinity concentrations, 32 of 45 Martian sites also have clusters of boulders in the final 10% of the centerline profile, suggesting the presence of drop-moraine–like clusters (e.g., ref. 25) at many sites.

Together, the clustering of boulders on LDAs into bands and the lack of a monotonic comminution trend down-flow suggests that englacial transport, rather than exclusively surficial transport, occurs on Martian LDAs. The presence of boulder clusters on LDA that are associated with arcuate surface discontinuities and other surface lineations (Fig. 2 and *SI Appendix*) suggests that rocky debris is incorporated into LDA at accumulation zone headwall scarps and is advected down-glacier as fine-scale internal debris layers. These debris layers outcrop at the surface in response to sublimation, producing boulder clusters. If, as on Earth, debris-layer accumulation occurs predominantly during periods of negative mass-balance in the glacier’s headwall region, it suggests that multiple ice deposition events are recorded in each LDA. Accordingly, these findings provide a geomorphic climate proxy that extends the record of orbitally paced climate change on Mars over hundreds of millions of years. Longer duration climate records preserved in LDA provide a framework for linking recent Amazonian climate change recorded in polar layered deposits (e.g., ref. 7) to long-term changes in Martian-volatile reservoirs. These glacial indicators of ice accumulation and hiatus provide empirical evidence supporting multiple, large-scale obliquity change episodes over the past ∼300 to 800 Ma, extending evidence for Martian climate change beyond the 20 Ma window provided by numerical modeling (8).

Particular support for this model comes from the widespread presence of headwall boulder clusters currently found on LDA. Midlatitude ice accumulation is not presently occurring at LDA sites, however, boulders are accumulating through rockfall near LDA apices. This observation suggests that the clasts that will form internal debris layers during the next period of positive mass-balance are presently accumulating in the headwall regions of LDAs. Debris layers currently growing in the absence of ice deposition in LDA headwalls are a prediction of the internal debris-layer formation process models suggested by terrestrial debris-covered glaciers.

The presence of multiple boulder bands on all mapped LDAs suggests that boulder position atop Martian glacial landforms may largely be controlled by subsurface debris layers intersecting the surficial sublimation lag. If boulder clusters reflect the presence of subsurface debris layers, it implies that LDAs have formed over multiple climate cycles in which LDA accumulation in headwall region ceases, talus accumulates, and the debris layer is ultimately reburied by subsequent glacial advance. Subsurface debris in this model must be within layers that are too thin or too steep to be detected by recent orbital radars that have confirmed LDA bulk composition to be consistent with water ice (26–28). For comparison, boulder bands on terrestrial debris-covered glaciers can form from englacial debris-rich layers as thin as 5 to 10 m thick that steeply intersect the glacier surface at angles exceeding 25 to 35° (18).

Intriguingly, the increase in debris band number at higher latitudes suggests that near-polar sites have experienced more cycles of ice deposition and ablation than low-latitude sites. This suggests that near-surface ice stability is a key factor in controlling LDA accumulation and ice flow, and that polar sites reach the threshold at which ice can flow more regularly than lower latitude sites where surface ice deposition occurs only at the highest of obliquities (5). This would be consistent with prior observations of multiple topographic undulations on LDA and middle and high latitudes that are inferred to result from multiple obliquity-paced ice deposition events (6, 29, 30).

The positive correlation between latitude, flow orientation, and boulder bands suggests that energy balance and ice mass balance work across global length scales on Mars to drive glaciation at LDA sites. Boulder clustering on short length scales (e.g., high numbers of nominal bands) may reflect site-specific idiosyncrasies of rockfall frequency (e.g., ref. 31) related to local geology or ice mass balance.

Boulder banding provides independent evidence of episodic accumulation and flow of LDA over long timescales, supporting observations of ridges and folds in LDAs that have been interpreted as evidence of climate-driven change in LDA accumulation (6, 32). If boulder band accumulation occurred evenly over the past ∼800-Ma period of LDA emplacement the number of boulder bands observed on LDA suggests accumulation zone deposition hiatuses occur on timescales ranging from ∼10 to 100 Ma. Along with variable resurfacing, this process helps explain the multiple surface ages recorded by crater size-frequency measurements on LDAs (13, 33); some portions of LDAs may be tens to hundreds of Ma older than other segments, as indicated by crater counts.

If topographic ridges on some LDAs are generated by deformation of a surface lag under conditions of episodic accumulation and flow, this may provide a mechanism for forming terminal, moraine-like ridges co-located with moraine-like boulder clusters. Under such a model, a boulder band might originate at the headwall scarp base, while undulations and ridges on LDA might form further down-glacier where thicker, faster ice (e.g., ref. 34) “pulses” merge into the preexisting LDA. However, because boulder bands on Earth (19) and Mars (e.g., Fig. 3) are not always associated with undulating topography or breaks in slope, it suggests that the ice flow mechanisms that thicken or thin LDA ice are different from the mechanism that generates most boulder bands (i.e., accumulation hiatus and resumption).

Future in situ investigations of LDAs and other Martian glacial landforms may need to consider the role of internal debris layers and the presence of ice of greatly different ages as factors in exploration. Landing sites or in situ resource utilization sites, where LDA ice could be used for human consumption or habitat shielding, should consider avoiding surfaces upslope of boulder clusters, where subsurface debris layers may impede drilling or sampling.

## Materials and Methods

Boulders were mapped on full-resolution, 25 cm/pixel HiRISE (35) images that overlapped LDAs identified in the ref. 2 catalog. Sites were required to have the entire LDA flow path, from headwall scarp to LDA toe, visible in the image or overlapping neighboring images. For the 18 sites for which boulder size was measured, sites were selected where overlapping stereo HiRISE acquisitions and overlapping stereo Mars Reconnaissance Orbiter context camera images were available, which were then processed into Mars Orbiter Laser Altimeter (MOLA) tied stereo digital elevation models (DEMs) (36–38). LDA surface slope and centerline targeting was determined from these DEMs. For the remainder of the sites, LDAs were selected randomly from HiRISE images that intersect LDA footprints in the catalog (2).

Boulders were mapped along a 50-m buffer surrounding an inferred LDA flow centerline (transect width of 100 m). LDA centerlines were defined by examining topography for convex-out, down-glacier contours that allowed centerlines to follow paths with positive planform curvature, ensuring that flow on the LDA was divergent at the site and that no boulders could be delivered to the study region by convergent flow. Centerlines were also selected to avoid medial-moraine–like features (14) emerging from bedrock spurs where convergence between LDA lobes occurs.

Boulders were identified on the basis of image features with clearly visible edges, bright, sun-facing sides, and elongated shadows extending in the down-sun direction after ref. 22. Boulders were required to be at least three pixels wide and, for boulder width analysis, were filtered to remove boulders <1 m in width. Boulder size was determined by fitting line segments to the boulders in the sun-transverse direction after ref. 22. Boulders identified by location-only were mapped as points. Boulders we mapped at ∼1:200 to 1:300 scale to ensure that individual HiRISE pixels could be inspected and counted. Boulders were mapped in multiple passes up and down transects, and boulders were spot checked for quality assurance.

Boulder location was then transformed from Mars geographic coordinates to centerline path coordinates to produce measures of boulder location relative to the LDA headwall scarp and distance along the centerline (see MatchingBoulders_v2.m in code repository). Boulder size statistics (median and 95th percentile) were determined for groups of boulders in 50-m distance bins along LDA centerlines where width was measured.

Spatial clusters of boulders were identified using a K-means clustering approach (23) (see boulder_analysis.R in code repository). The nominal number of bands at each site was determined by maximizing the BIC. Because K-means clustering analysis is sensitive to boulder clustering at all spatial scales, a second banding parameter, “preferred number of clusters,” was determined by examining each LDA for natural breaks in boulder density revealed through kernel density analysis (39) and inspection of the K-means clustering report in order to highlight long-range clustering. Kernel density analysis fits a probability density function to each mapped boulder and sums them over a moving window to generate a spatial statistical representation of boulder density that accounts for the fact that some boulders may be missed where boulders are closely spaced. Kernel density plots were used to identify clusters of boulders spatially proximal to LDA headwalls, LDA termini, and boulder bands located along LDA mapped transects (Fig. 2 and *SI Appendix*).

Boulder spatial density was determined summing boulders by length increments down LDA flowlines. Number density is reported as boulders per 1% of centerline length (see Bin_Density.Rmd in code repository).

## Supplementary Material

Supplementary File

## Data Availability

Geospatial data and reduced data records data have been deposited in the University of Texas Data Repository (https://doi.org/10.26153/tsw/9330) (40). Code has been archived at GitHub (https://github.com/fairaque1999/mars_glacier_boulders).
