# Supplementary material for: Surface boulder banding indicates Martian debris-covered glaciers formed over multiple glaciations
Source: Proc Natl Acad Sci U S A. 2021 Jan 18;118(4):e2015971118. doi: 10.1073/pnas.2015971118 (PMC7848752; doi:10.1073/pnas.2015971118)
Supplement: Supplementary File [file pnas.2015971118.sapp.pdf]

Supporting Information

Supplementary Data. A geodatabase package containing all ArcGIS shapefiles produced in this analysis is available for download from the University of Texas data repository. Doi: 10.26153/tsw/9300. All HiRISE images used in this study are archived at the NASA PDS and are available for direct download via: hirise.lpl.arizona.edu

Supplementary Table 1. Dataset summary for all LDA sites. Image number(s) are reported, as it image center latitude and longitude. Abslat indicates absolute latitude. Nominal indicates K-means derived number of boulder clusters. Preferred indicates preferred number of boulder bands, focusing on broad-scale structure. FirstPick indicates the observed inflection point on the K-means BIC report beyond which reductions in BIC penalty is small in comparison to the nominal value. Length indicates LDA centerline length. Azimuth is the orientation of the centerline from 0˚ = north, determined by comparing the centerline start point to its end-point. PC_Azimuth is the pole-corrected azimuth, correcting azimuths to their equivalent in the northern hemisphere (i.e., southern hemisphere sites with a flow direction of 180˚ are pole-facing, and would be corrected to 0˚). Cos_PC_Azimuth is the cosine of the pole-corrected azimuth. Num_mapped_boulders is the number of boulders mapped along each centerline. LDA relief is the elevation change from the top of the centerline to the bottom, computed as the difference between the minimum LDA elevation and the maximum LDA elevation (Min_LDA_elev and Max_LDA elev). Max_scarp_elev is the elevation of the headwall scarp immediately upslope of the LDA centerline. Dist_LDA_Scarp is the distance in meters between the top of the bedrock scarp and the top of the LDA. Scarp_slope is the average slope along the bedrock exposure immediately upslope of the LDA. LDA_slope is the average surface slope of the LDA. Phase_angle indicates the HiRISE image phase angle. Craters + CLF indicates the number of craters >100 m and crater-like-forms (CLFs) in the HiRISE image surrounding the transect. Brain Terrain? Indicates whether brain terrain surface textures are observed at the site. Polygons? Indicates whether polygonally-patterned mantle material overlies the LDA. Scarp cluster indicates whether a boulder cluster is present in the first 10% of the transect. Moraine like cluster indicates whether a boulder cluster is present in the final 10% of the centerline transect. ASD-like bands indicates whether arcuate surface discontinuities are present at the site. ASD_num indicates how many ASD-like clusters are apparent in the boulder kernel density plots.

Site	Image	Lat	Long	Abslat	Nominal	Preferred	FirstPick	Length	Azimuth	PC_Azimuth	Cos_PC_Azimuth	num_mapped_boulders	LDA_relief	Min_LDA_elev	Max_LDA_elev	Max_scarp_elev	Dist_LDA_Scarp	scarp_slope	LDA_slope	phase_angle	Craters + CLF	Brain Terrain? (Y/N)	Polygons?(Y/N)	Scarp_cluster	Moraine_like_cluster	ASD_like_bands	ASD_num
A	PSP_007693_2300_PSP_008550_2300	49.573	286.336	49.573	12	5	5	6200.1	115.67	115.67	-0.433187222	7583	393	-3281	-2888	-2008	2300	0.383	0.063	51.9	16	N	Y	Y	Y	Y	8
B	PSP_004167_1400	-39.604	87.912	39.604	8	6	5	4649.72	92.78	272.78	0.048501118	692	143	-5186	-5043	-4949	953	0.099	0.031	56.5	1	Y	Y	N	Y	Y	5
C	ESP_016471_2260_ESP_016194_2260	45.696	33.444	45.696	5	4	3	2944.4	129.7	129.7	-0.638767818	811	249	-2546	-2297	-2192	1520	0.069	0.085	44	6	N	Y	N	Y	Y	4
D1	ESP_028324_2145_ESP_028601_2145	34.171	73.621	34.171	10	7	5	1416.51	123.04	123.04	-0.545224405	1429	194	-2263	-2069	-2007	358	0.173	0.137	53.6	27	N	N	Y	Y	Y	9
D2	ESP_028324_2145_ESP_028601_2145	34.22	73.636	34.22	3	2	3	1067	215.1	215.1	-0.818149717	715	57	-2301	-2244	-2129	888	0.13	0.053	53.6	27	Y	N	Y	Y	Y	7
D3	ESP_028324_2145_ESP_028601_2145	34.187	73.662	34.187	5	5	5	1543	265.2	265.2	-0.083677843	433	48	-2306	-2258	-2233	519	0.048	0.031	53.6	27	N	N	N	Y	Y	4
D4	ESP_028324_2145_ESP_028601_2145	34.15	73.625	34.15	10	10	4	1657	334.086	334.086	0.899451021	1193	101	-2273	-2172	-2050	518	0.236	0.061	53.6	27	N	N	Y	Y	Y	6
D5	ESP_028324_2145_ESP_028601_2145	34.147	73.592	34.147	10	5	4	1580	16.8	16.8	0.957319498	871	59	-2251	-2192	-2168	283	0.085	0.037	53.6	27	N	N	Y	N	Y	5
E	ESP_015979_1415_PSP_007250_1415	-38.351	155.371	38.351	14	4	6	3371	176.9	176.9	-0.99853667	6167	122	695	817	1267	1395	0.323	0.036	56	13	N	N	Y	Y	Y	8
F	ESP_023937_2240_PSP_010487_2240	43.462	319.42	43.462	21	5	4	7845	343.1	343.1	0.956813584	4118	806	-4651	-3845	-3845	0	N/A	0.103	47.5	0	Y	Y	Y	N	Y	4
H1	PSP_008563_2320_PSP_008774_2320	51.777	288.932	51.777	9	4	6	4354	86.9	86.9	0.054078813	1267	414	-3466	-3052	-2089	3570	0.27	0.095	36.7	29	N	Y	N	Y	Y	4
H2	PSP_008563_2320_PSP_008774_2320	51.862	288.902	51.862	13	9	6	5234	70.3	70.3	0.337095258	1314	433	-3459	-3026	-2291	2766	0.266	0.083	36.7	29	N	Y	Y	Y	Y	5
J1	ESP_016132_2300_ESP_016277_2300	49.721	285.257	49.721	4	4	2	6173	26.8	26.8	0.892585819	1145	708	-3186	-2478	-2194	1108	0.256	0.115	47.1	5	N	Y	Y	Y	Y	4
J2	ESP_016132_2300_ESP_016277_2300	49.721	285.257	49.721	4	4	2	6021	38.7	38.7	0.780430407	1750	687	-3156	-2469	-2194	1267	0.217	0.114	47.1	5	N	Y	N	N	Y	4
K	PSP_001357_2300	39.48	105.449	39.48	12	9	10	8790.45	343.421	343.421	0.958428012	962	1032	-5662	-4630	-4630	0	N/A	0.117	43.3	1	Y	N	N	N	Y	8
L	ESP_016271_1475_ESP_016416_1475	-32.371	100.848	32.371	7	4	5	6600.97	339.444	159.444	-0.936329179	1358	1455	-1120	335	832	773.565	0.642	0.22	67.8	10	N	N	Y	N	Y	4
M	ESP_020558_2215_ESP_020835_2215	41.173	50.871	41.173	6	9	5	10722.44	349.923	349.923	0.984574038	182	1567	-2017	-450	-194	1068	0.24	0.146	65.5	0	N	Y	Y	Y	Y	4
N2	ESP_03363_2225_ESP_033353_2225	42.328	18.338	42.328	4	4	3	6545.83	154.026	154.026	-0.898992873	1018	1281	-3671	-2390	-2128	1476.57	0.177	0.196	51.6	13	N	N	Y	Y	Y	5
P	PSP_001882_1410_PSP_001816_1410	-38.726	193.993	38.726	10	5	5	2967	157.9	157.9	-0.926528631	1023	726	185	911	911	0	N/A	0.245	72.5	15	N	Y	N	Y	Y	6
Q1	ESP_028115_2225 + Q1	42.688	18.375	42.688	10	7	4	7164.39	24.433	24.433	0.910445868	757	392	-3541	-3149	-2114	2403.92	0.431	0.055	59.2	16	Y	Y	Y	N	Y	4
Q2	ESP_028115_2225 + Q1	42.496	18.32	42.496	11	8	3	5306.57	210.3	210.3	-0.863396916	611	345	-3659	-3314	-3225	1010.083	0.088	0.065	59.2	16	Y	N	Y	Y	Y	6
R	ESP_025319_2240	43.534	28.034	43.534	8	7	2	9047.7	207.289	207.289	-0.888705271	210	1359	-3615	-2256	-2133	595	0.207	0.15	44.3	3	Y	Y	N	Y	Y	6
S	ESP_018515_2225	42.131	27.346	42.131	5	6	3	4872.82	34.844	34.844	0.820710691	275	443	-3540	-3097	-2819	977	0.285	0.091	41.9	2	Y	Y	N	Y	N	2
T	ESP_020319_1470	-32.691	105.271	32.691	2	2	1	3509.84	121.61	121.61	-0.524134552	65	944	1101	2045	2946	2484	0.363	0.269	60.5	22	N	N	Y	N	Y	2
U	ESP_037675_2170	36.651	17.849	36.651	5	4	2	3087.21	179.199	179.199	-0.999902236	362	58	-2857	-2799	-2159	1980.66	0.323	0.019	61.2	10	Y	Y	N	Y	N	3
V	ESP_026698_2115	31.095	-177.628	31.095	5	5	3	7717.55	332.019	152.019	-0.883102261	41	298	-3763	-3465	-2712	3127.03	0.241	0.039	45.9	12	N	N	Y	N	Y	3
W	ESP_016266_2165	36.048	229.768	36.048	2	3	3	8786.73	196.911	196.911	-0.956757756	115	370	414	784	1413	3545	0.177	0.042	35.1	15	Y	N	N	N	Y	3
X	ESP_019020_2310	50.635	278.4	50.635	7	10	3	7284.55	7.58	7.58	0.991261646	1483	545	-2638	-2093	-1137	2882.92	0.332	0.075	47.9	3	N	Y	Y	Y	Y	7
Y	ESP_019890_2295	49.007	285.502	49.007	13	12	3	8628.89	306.453	306.453	0.594158111	1669	1091	-3257	-2166	-1919	1221.23	0.202	0.126	68.6	7	N	Y	Y	Y	Y	7
Z	PSP_009064_2315	51.241	288.891	51.241	22	23	4	7682.41	339.921	339.921	0.939217954	7811	1022	-3415	-2393	-2299	880.524	0.107	0.133	46.1	11	N	N	Y	Y	Y	6
AA	ESP_019794_2240	43.732	28.125	43.732	3	4	3	12599.18	17.463	17.463	0.953910939	867	1359	-3530	-2171	-2107	210	0.305	0.108	59.8	8	Y	Y	Y	N	Y	3
BB	ESP_035819_2300	49.736	285.369	49.736	13	13	3	8417.66	26.929	26.929	0.891568418	2717	826	-3230	-2404	-1856	1317	0.416	0.098	44.7	8	N	Y	Y	Y	Y	6
CC1	ESP_055396_2210	40.713	62.411	40.713	5	5	4	2659.95	77.824	77.824	0.210922559	152	386	-2280	-1894	-1787	518.805	0.206	0.145	54.5	3	N	Y	Y	N	Y	4
CC2	ESP_055396_2210	40.75	62.359	40.75	5	5	2	2151.82	3.356	3.356	0.998284915	230	345	-2290	-1945	-1875	878.194	0.08	0.16	54.5	3	N	Y	Y	N	Y	4
DD1	ESP_028324_2145_ESP_028601_2145	38.572	171.92	38.572	2	5	2	1360.79	321.489	321.489	0.782492225	212	59	-3946	-3887	-3877	803.102	0.012	0.043	33.2	65	N	Y	Y	Y	N	2
DD2	ESP_028324_2145_ESP_028601_2145	38.525	171.915	38.525	2	4	2	1736.19	228.359	228.359	-0.664463699	263	38	-3931	-3893	-3884	1036.58	0.009	0.022	33.2	65	N	Y	N	Y	Y	3
DD3	ESP_028324_2145_ESP_028601_2145	38.448	171.841	38.448	2	5	2	1031.78	264.014	264.014	-0.104277885	250	40	-3914	-3874	-3755	736.85	0.161	0.039	33.2	65	Y	Y	N	Y	Y	2
DD4	ESP_028324_2145_ESP_028601_2145	38.47	171.877	38.47	6	6	3	1604.79	4.335	4.335	0.997139709	1099	94	-3932	-3838	-3774	541.168	0.118	0.059	33.2	65	N	Y	Y	Y	Y	3
DD5	ESP_028324_2145_ESP_028601_2145	38.441	171.899	38.441	3	4	1	711.72	111.445	111.445	-0.365600172	122	30	-3891	-3861	-3764	797.433	0.122	0.042	33.2	65	Y	Y	Y	N	Y	4
DD6	ESP_028324_2145_ESP_028601_2145	38.448	171.941	38.448	2	4	2	1354.74	326.427	326.427	0.833181108	90	17	-3938	-3921	-3919	373.721	0.005	0.013	33.2	65	Y	N	Y	Y	Y	4
DD7	ESP_028324_2145_ESP_028601_2145	38.479	171.945	38.479	2	4	2	1027.56	179.007	179.007	-0.999849692	313	5	-3939	-3934	-3928	462.741	0.013	0.005	33.2	65	Y	N	N	Y	Y	3
EE	ESP_019020_2310	50.635	278.4	50.635	10	10	6	8713.51	20.181	20.181	0.938607476	3635	737	-2681	-1944	-881	3110	0.342	0.085	47.9	3	N	Y	Y	Y	Y	6
FF	ESP_025494_2320 and PSP_008563_2320	51.777	288.932	51.777	9	11	5	11825.53	109.162	109.162	-0.328240356	2995	1489	-3444	-1955	-1955	0	N/A	0.126	47.6	21	N	Y	Y	Y	Y	10
GG	ESP_026071_2195	39.363	19.321	39.363	8	8	2	7144.75	2.598	2.598	0.998971759	656	854	-3485	-2631	-2552	723	0.109	0.12	40.3	14	Y	Y	Y	Y	Y	4
HH	PSP_007558_2135	33.275	16.965	33.275	2	5	2	6014.7	25.285	25.285	0.904197897	88	238	-3027	-2789	-1758	2465	0.418	0.04	35.3	3	Y	Y	N	Y	Y	3

Supplementary Table 1. Site locations and observations

	Latitude (Degrees)	Pole Corr. Azimuth	Cosine Azimuth	Scarp Slope	LDA Slope	Max LDA Elevation	Number of Boulders	Nominal Clusters	Preferred Clusters	First Pick Clusters	Length (m)	Craters + CLF	Number of ASD
**Latitude (Degrees)**	-	0.15	0.02	0.462	0.414	0.302	0.002	0.002	0.002	0.159	0.004	0.072	0.063
**Pole Corr. Azimuth**	-0.229	-	0.081	0.017	0.328	0.817	0.824	0.716	0.23	0.784	0.327	0.086	0.683
**Cosine Azimuth**	0.362	-0.276	-	0.867	0.86	0.248	0.762	0.274	0.004	0.934	0.187	0.454	0.586
**Scarp Slope**	0.118	-0.371	0.027	-	0.016	0.003	0.125	0.196	0.81	0.065	0.001	0.001	0.448
**LDA Slope**	0.131	-0.157	0.028	0.373	-	0	0.814	0.481	0.451	0.997	0.026	0.004	0.704
**Max LDA Elevation**	-0.165	-0.037	-0.185	0.456	0.576	-	0.319	0.637	0.644	0.287	0.075	0.007	0.416
**Number of Boulders**	0.461	0.036	0.049	0.243	0.038	0.16	-	0	0.001	0.002	0.18	0.202	0
**Nominal Clusters**	0.467	0.059	0.175	0.206	0.113	0.076	0.74	-	0	0	0.074	0.034	0
**Preferred Clusters**	0.476	0.192	0.436	0.039	0.121	-0.074	0.512	0.78	-	0.127	0.018	0.138	0.012
**First Pick Clusters**	0.224	0.044	0.013	0.291	-0.001	0.17	0.464	0.584	0.242	-	0.287	0.072	0.001
**Length (m)**	0.439	-0.157	0.21	0.494	0.348	0.281	0.213	0.282	0.369	0.17	-	0	0.483
**Craters + CLF**	-0.284	0.272	-0.12	-0.504	-0.442	-0.417	-0.203	-0.331	-0.236	-0.284	-0.656	-	0.061
**Number of ASD**	0.294	0.066	-0.088	0.122	0.061	0.13	0.593	0.667	0.387	0.489	0.113	-0.295	-

Supplementary Table 2. Pearson correlation coefficients (lower left of diagonal) and p values (upper right of diagonal) for dataset summary in supplementary table 1.

	Latitude (Degrees)	Pole Corr. Azimuth	Cosine Azimuth	Scarp Slope	LDA Slope	Max LDA Elevation	Number of Boulders	Nominal Clusters	Preferred Clusters	First Pick Clusters	Length (m)	Craters + CLF	Number of ASD
**Latitude (Degrees)**	-	0.178	0.038	0.386	0.025	0.626	0.001	0.011	0.013	0.235	0.003	0.046	0.044
**Pole Corr. Azimuth**	-0.214	-	0.004	0.018	0.152	0.463	0.28	0.607	0.853	0.741	0.301	0.192	0.686
**Cosine Azimuth**	0.326	-0.444	-	0.85	0.161	0.763	0.324	0.245	0	0.807	0.251	0.163	0.919
**Scarp Slope**	0.139	-0.369	0.03	-	0.052	0.018	0.068	0.076	0.526	0.09	0	0.009	0.507
**LDA Slope**	0.35	-0.228	0.223	0.306	-	0	0.161	0.124	0.189	0.635	0.002	0.003	0.188
**Max LDA Elevation**	-0.078	-0.118	0.049	0.368	0.565	-	0.182	0.161	0.963	0.051	0.018	0.005	0.033
**Number of Boulders**	0.513	-0.173	0.158	0.287	0.223	0.213	-	0	0.029	0	0.135	0.607	0
**Nominal Clusters**	0.394	-0.083	0.186	0.281	0.244	0.223	0.7	-	0	0	0.022	0.203	0
**Preferred Clusters**	0.387	-0.03	0.525	0.102	0.209	0.008	0.341	0.684	-	0.026	0.023	0.084	0.005
**First Pick Clusters**	0.19	0.053	0.039	0.268	0.076	0.307	0.552	0.682	0.347	-	0.153	0.423	0
**Length (m)**	0.451	-0.166	0.183	0.549	0.473	0.368	0.237	0.356	0.354	0.227	-	0	0.24
**Craters + CLF**	-0.314	0.208	-0.222	-0.404	-0.453	-0.429	-0.083	-0.203	-0.273	-0.129	-0.638	-	0.297
**Number of ASD**	0.316	0.065	0.016	0.107	0.21	0.334	0.662	0.74	0.429	0.52	0.188	-0.167	-

Supplementary Table 3. Spearman correlation coefficients (lower left of diagonal) and p values (upper right of diagonal) for dataset summary in supplementary table 1.

Supplementary Figure 1. Spearman correlation (ρ_s) and Pearson correlation (ρ_P) summary for all measured LDA variables, expanded from Fig. 4 in main text. Across all measures of boulder clustering, number of boulder bands increase with absolute latitude, suggesting climate-linked accumulation dynamics controls boulder banding. Pair-plotting accomplished with seaborn in python. The diagonal of the pair-plots contain histograms of each data column with binning of 10. Upper half of the pairplots: Only scatterplot (using matplotlib.pyplot);lower half of the pairplts: Scatterplot (with seaborn) overlaid on kernel density plots of the data (with seaborn)

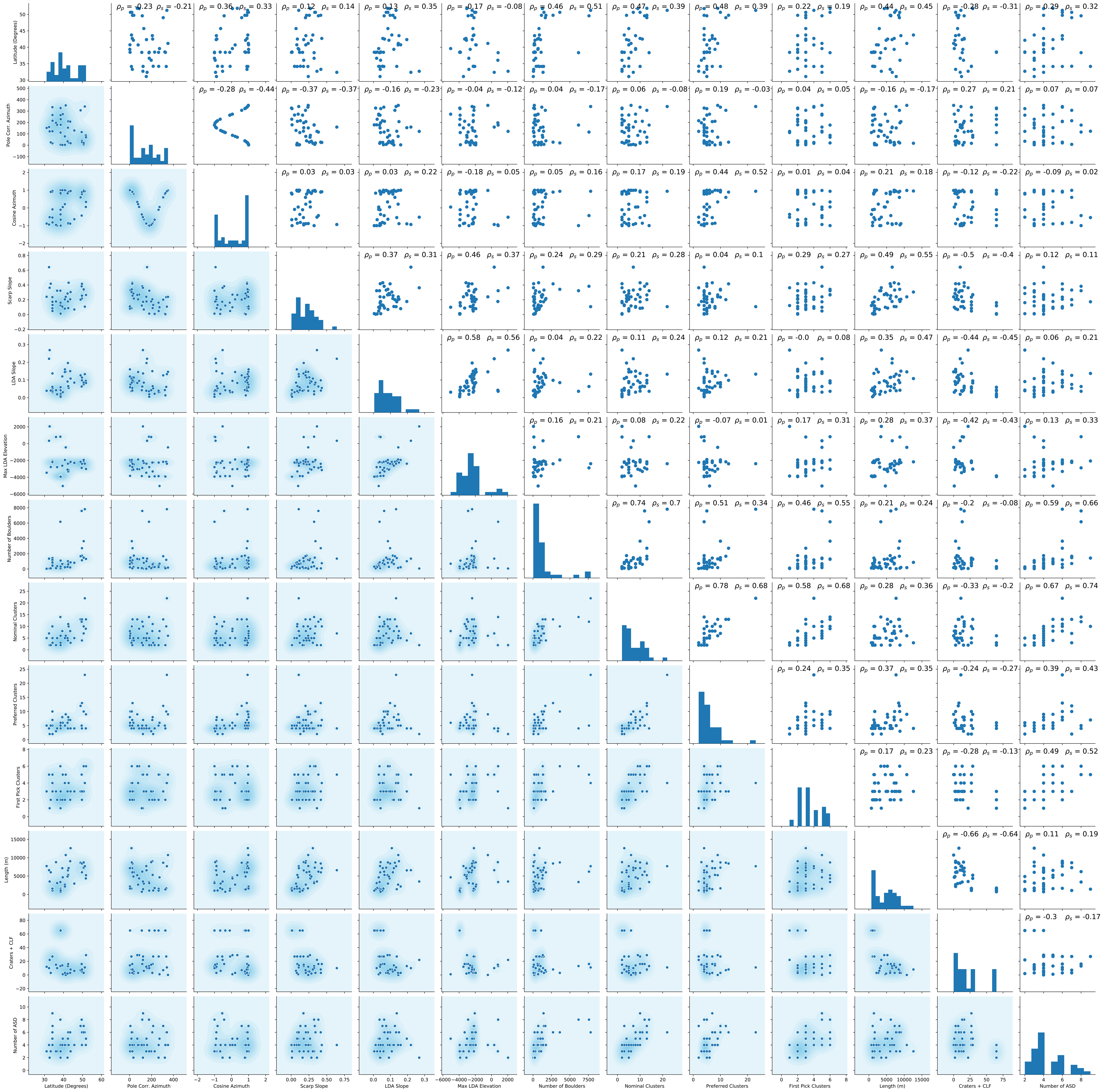

Latitude (Degrees)
Pole Corr. Azimuth
Cosine Azimuth
Scarp Slope
LDA Slope
Max LDA Elevation
Number of Boulders
Nominal Clusters
Preferred Clusters
First Pick Clusters
Length (m)
Craters + CLF
Number of ASD

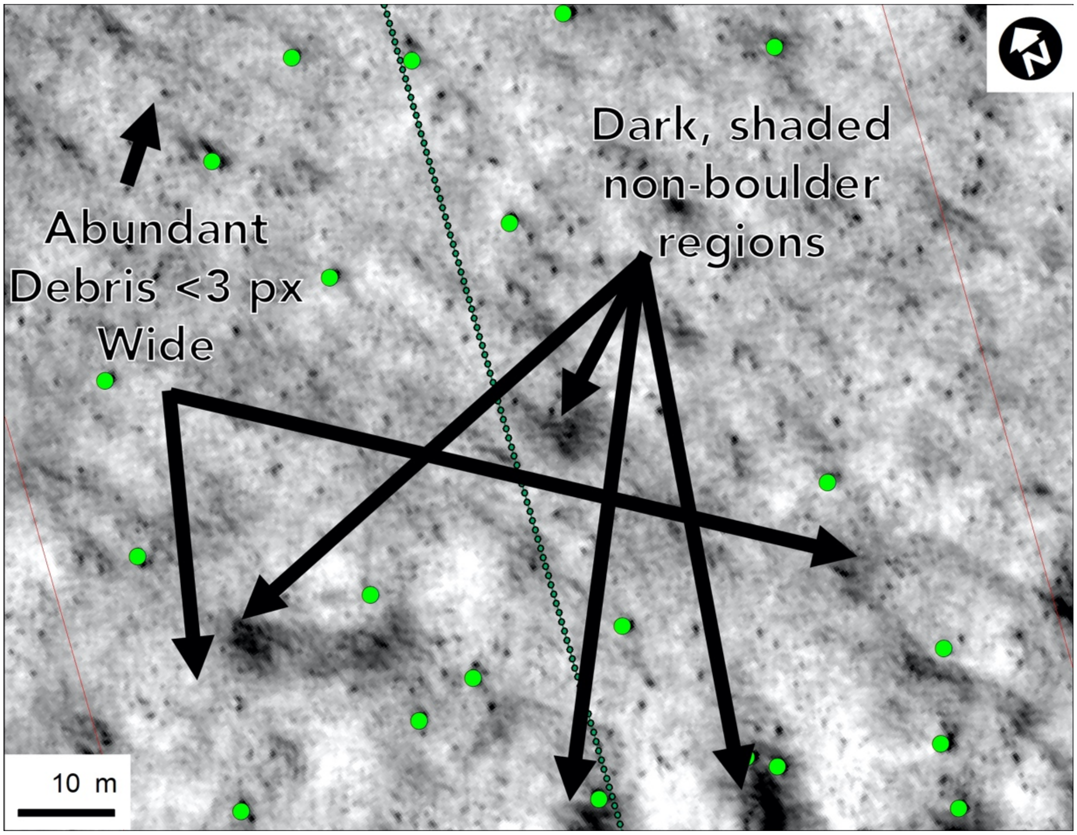
Dark, shaded
non-boulder
regions
Abundant
Debris <3 px
Wide
10 m

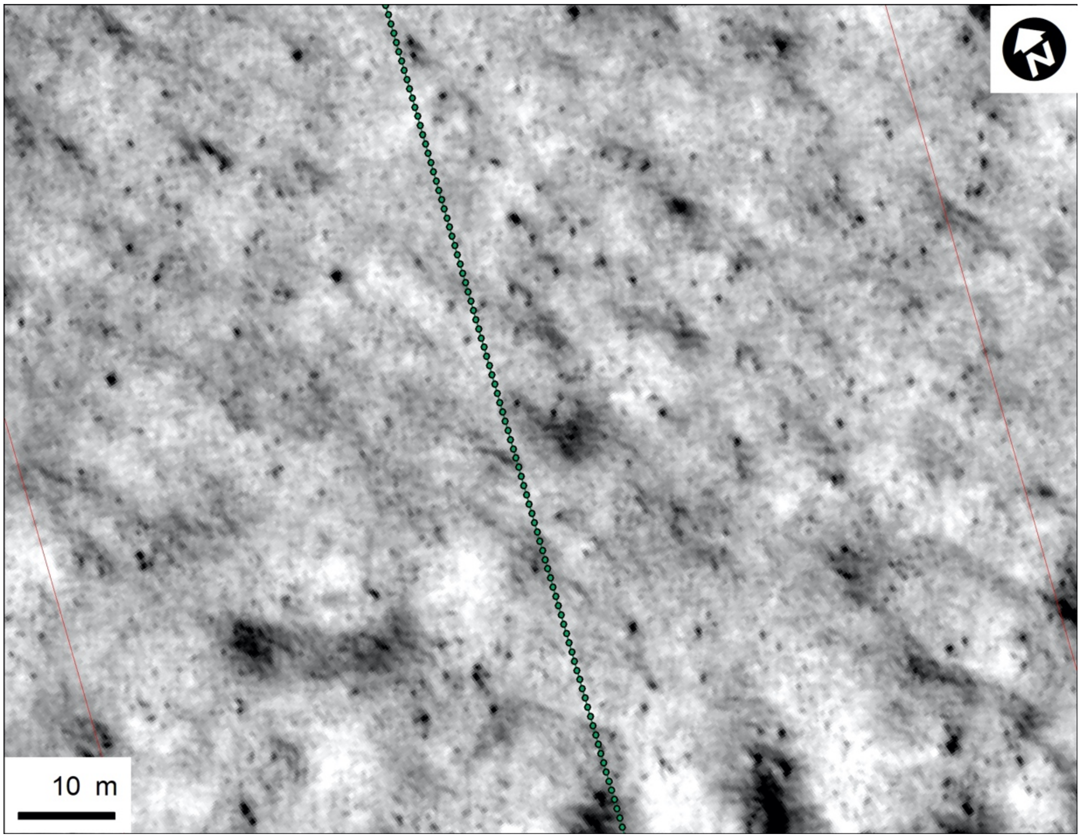
10 m

Supplementary Figure 2 (Previous Page). Detail showing individual boulder counts at example site (Q) at approximate mapping scale (~1:300), both with and without annotations. Counted boulders are marked with green dots. LDA are complex landforms which make autonomous boulder detection challenging. They include multiple speckles below the ~3 pixel threshold needed to identify landforms, as well as topographic and/or compositional dark or shaded areas not associated with boulder shadows. Transect centerline (closely spaced teal dots) and boundaries (dark red lines) are also plotted.

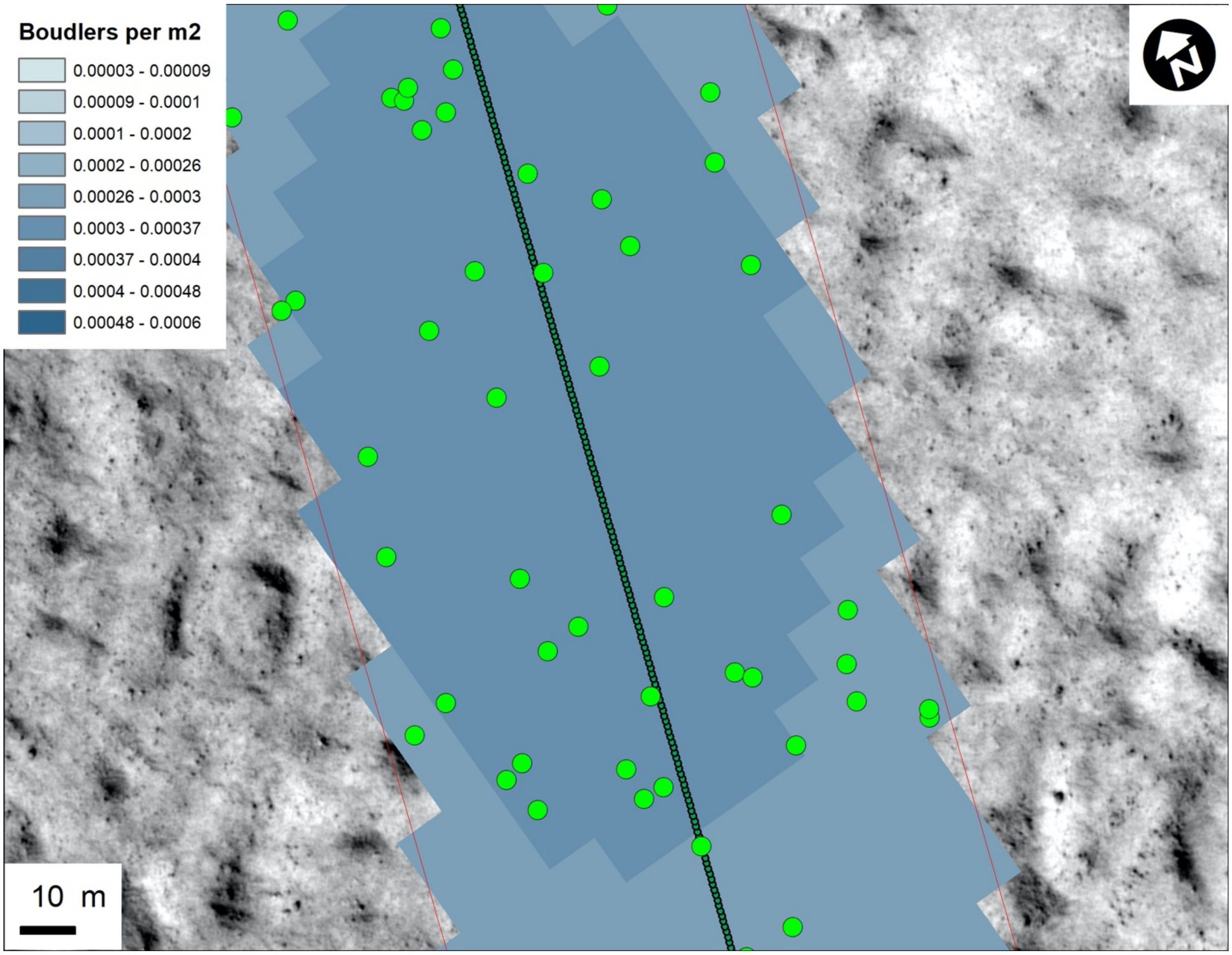

Supplementary Figure 3. The same region as supplementary figure 2, zoomed out slightly to show kernel density shading overlain. Boulder density is inhomogeneous over ~100 m length scales, and decreases upslope and downslope from the band.

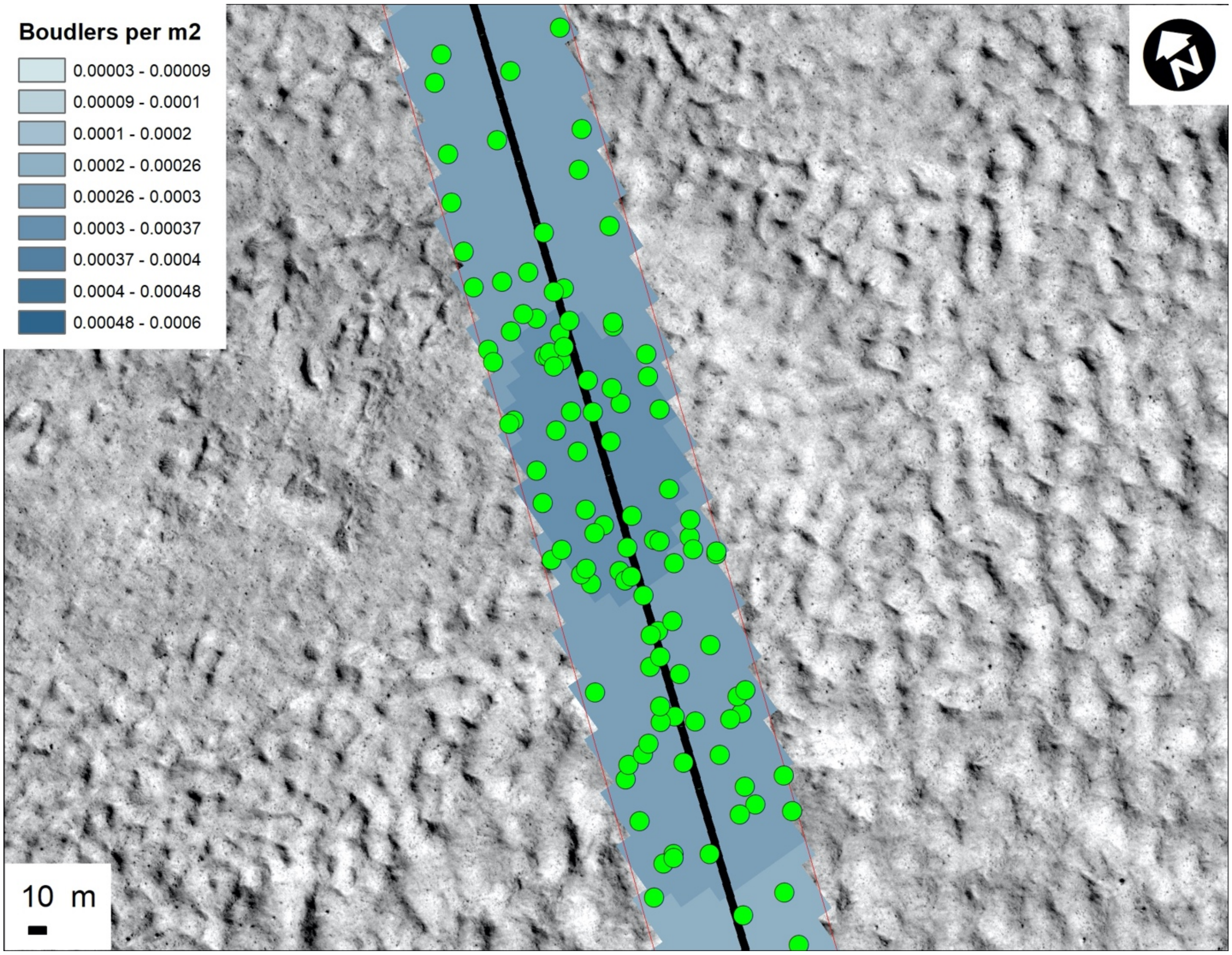

Supplementary Figure 4. Zoomed out version of supplementary figure 3. Boulder density is concentrated near the center of the field of view and falls off above and below this location. High kernel density sites are interpreted as locations where mapping transects cross boulder bands.

Supplementary Galleries.

Four collections of supplementary data are provided: 1) Plots of boulder size versus distance down-LDA for all measured sites; 2) K-means clustering plots and BIC reports for all sites; 3) maps of boulder kernel density for all sites; and 4) 1D boulder density plots for all sites.

Supplementary Galleries.

Four collections of supplementary data are provided: 1) Plots of boulder size versus distance down-LDA for all measured sites; 2) K-means clustering plots and BIC reports for all sites; 3) maps of boulder kernel density for all sites; and 4) 1D boulder density plots for all sites.

Boulder Diameter Plots

A_PSP_007693_2300_PSP_008550_2300

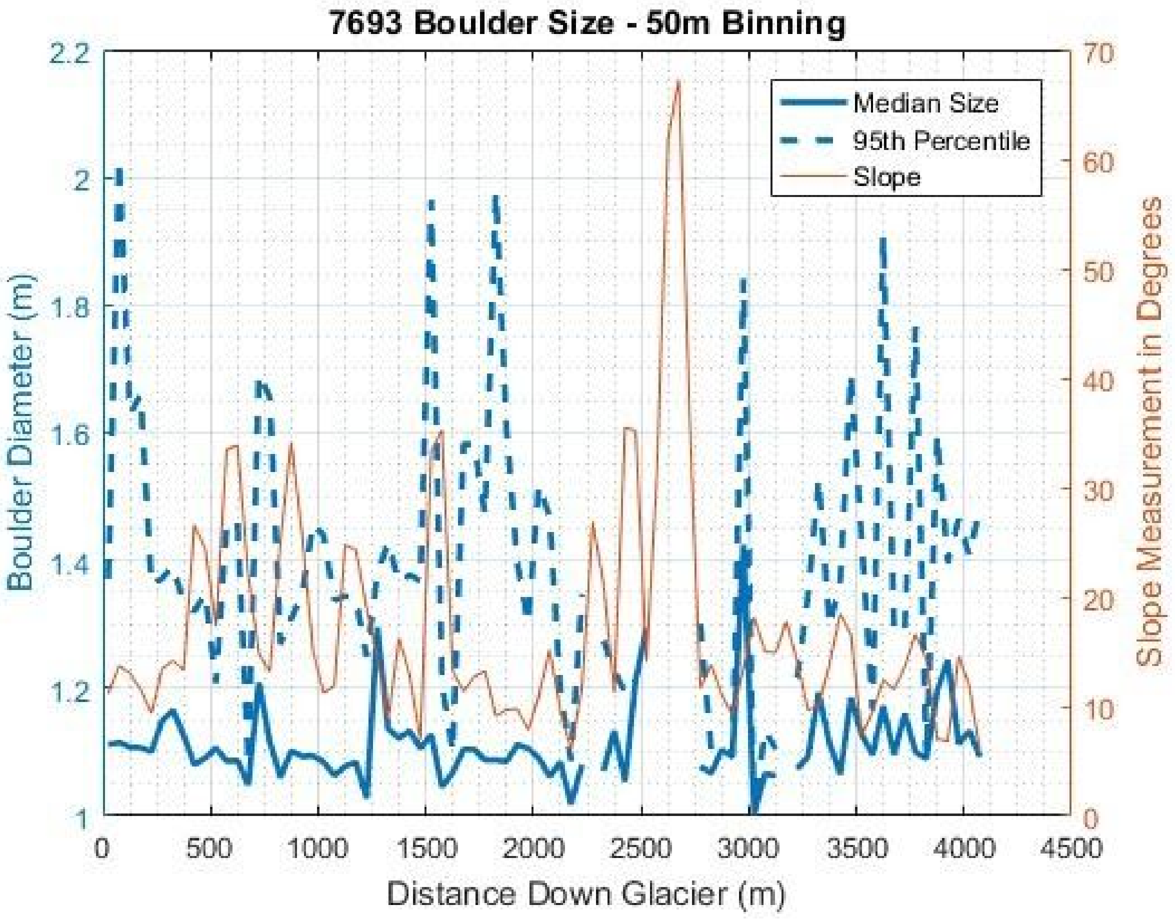

B_PSP_004167_1400b

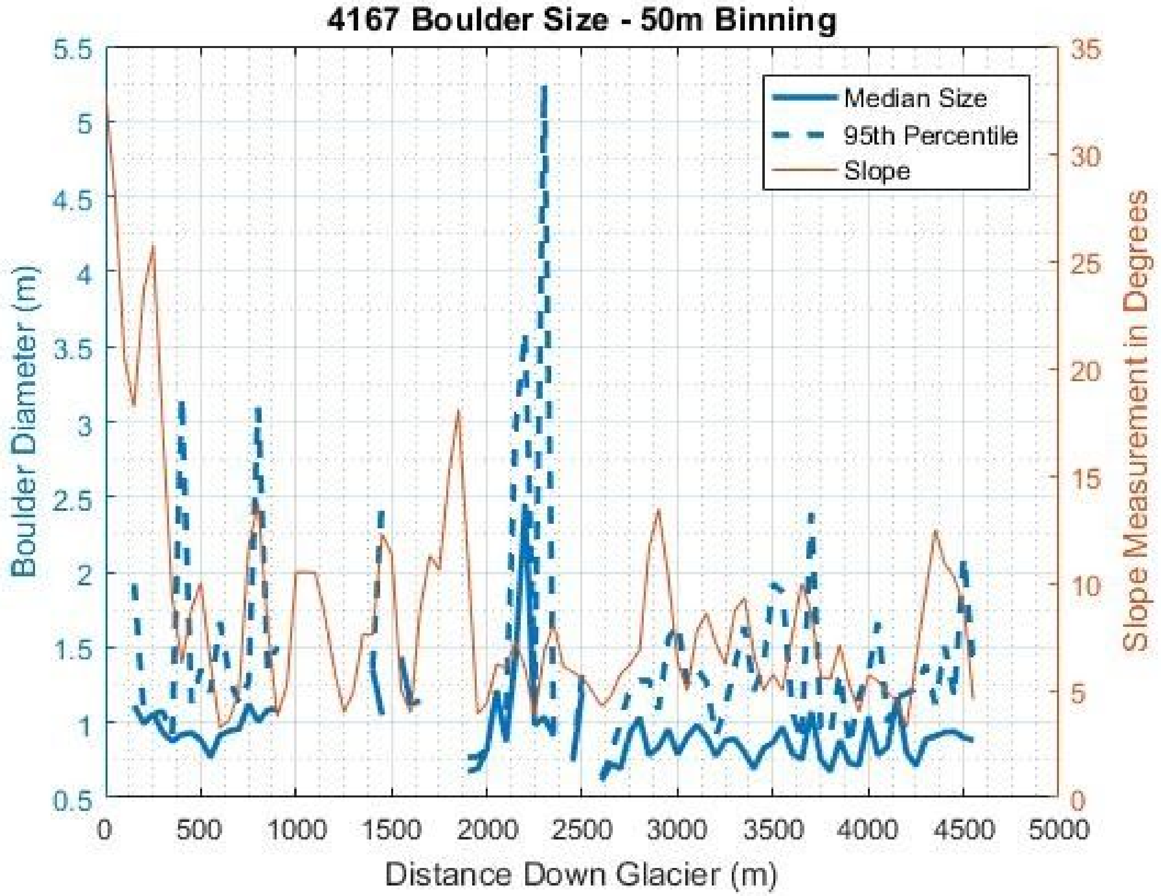

C_ESP_016471_2260_ESP_016194_2260

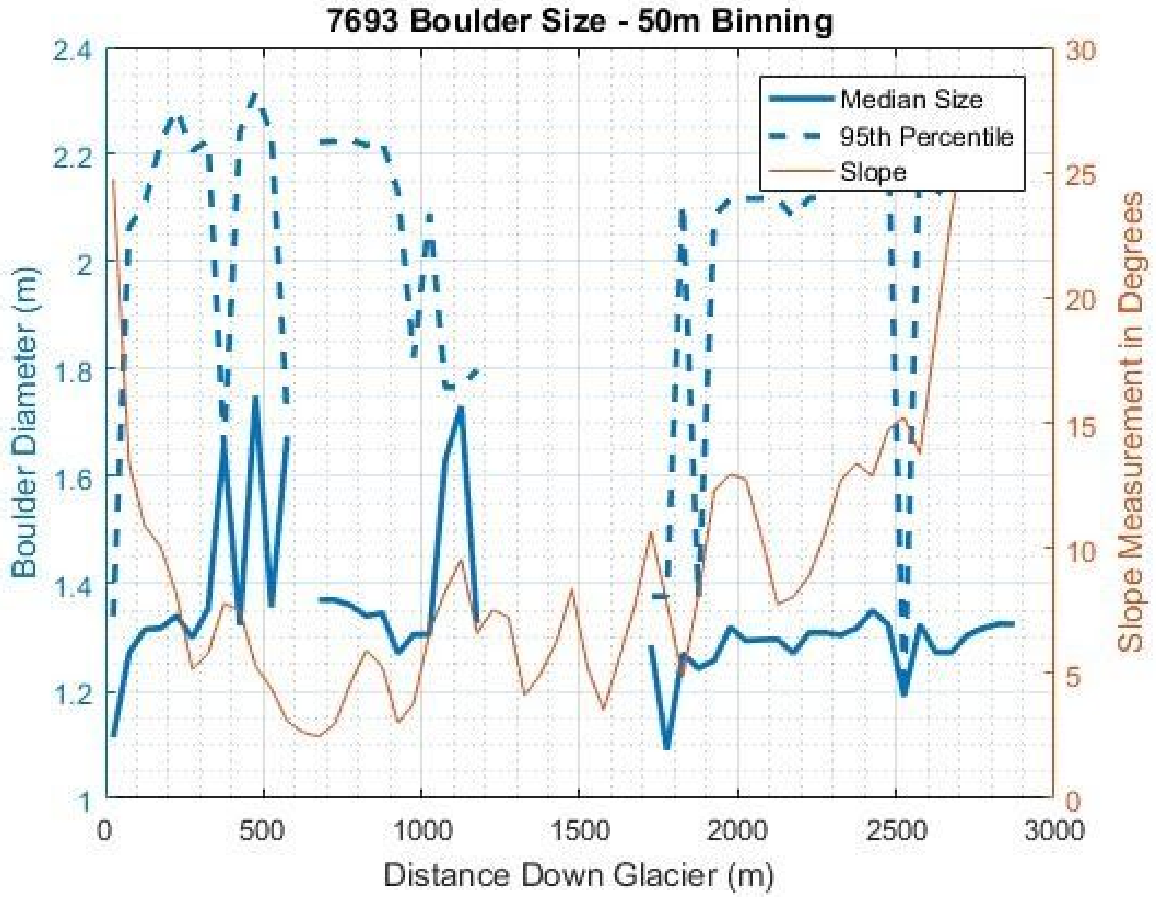

D_ESP_028324_2145_ESP_028601_2145

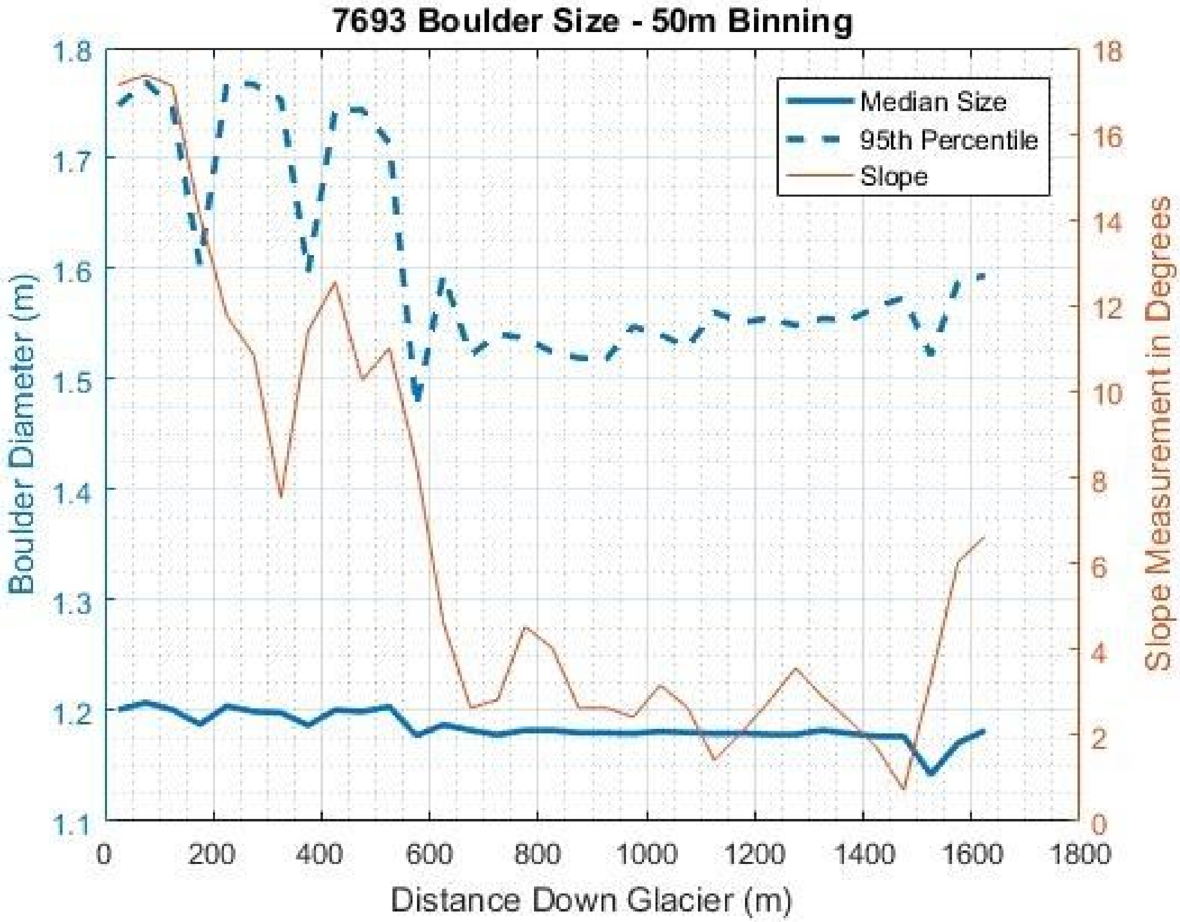

D2

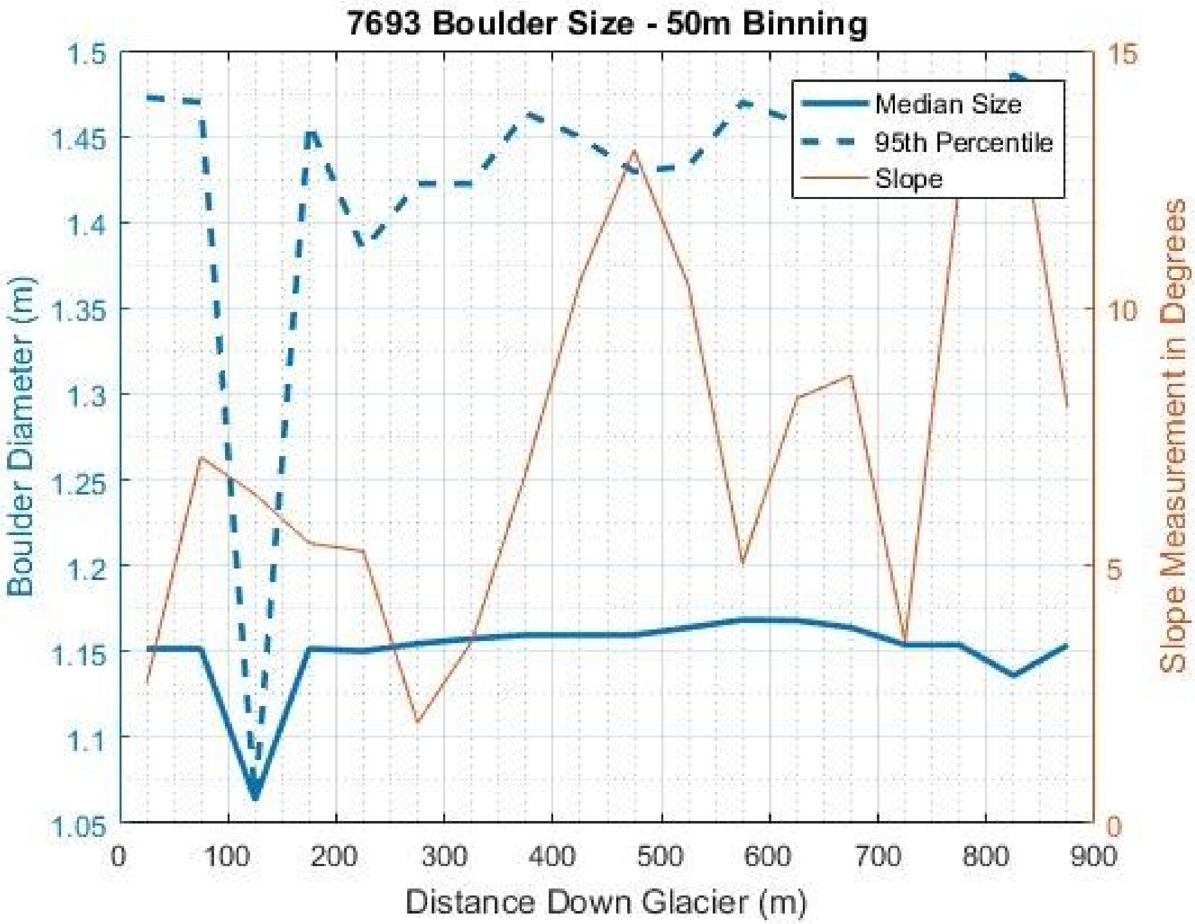
7693 Boulder Size - 50m Binning
Median Size
95th Percentile
Slope
Boulder Diameter (m)
Slope Measurement in Degrees
Distance Down Glacier (m)
1.5
1.45
1.4
1.35
1.3
1.25
1.2
1.15
1.1
1.05
15
10
5
0
0
100
200
300
400
500
600
700
800
900

D3

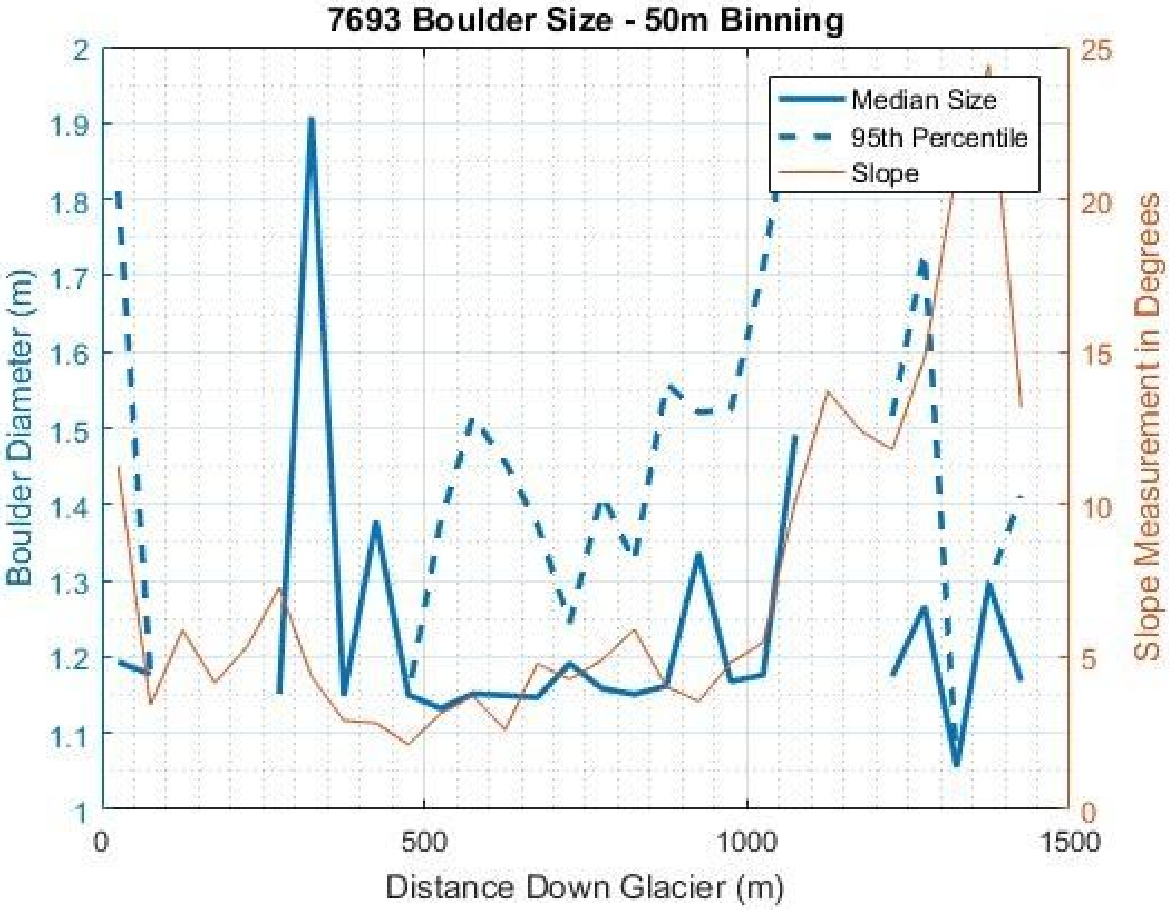
7693 Boulder Size - 50m Binning
Median Size
95th Percentile
Slope
Boulder Diameter (m)
Slope Measurement in Degrees
Distance Down Glacier (m)
2
1.9
1.8
1.7
1.6
1.5
1.4
1.3
1.2
1.1
1
25
20
15
10
5
0
0
500
1000
1500

D4

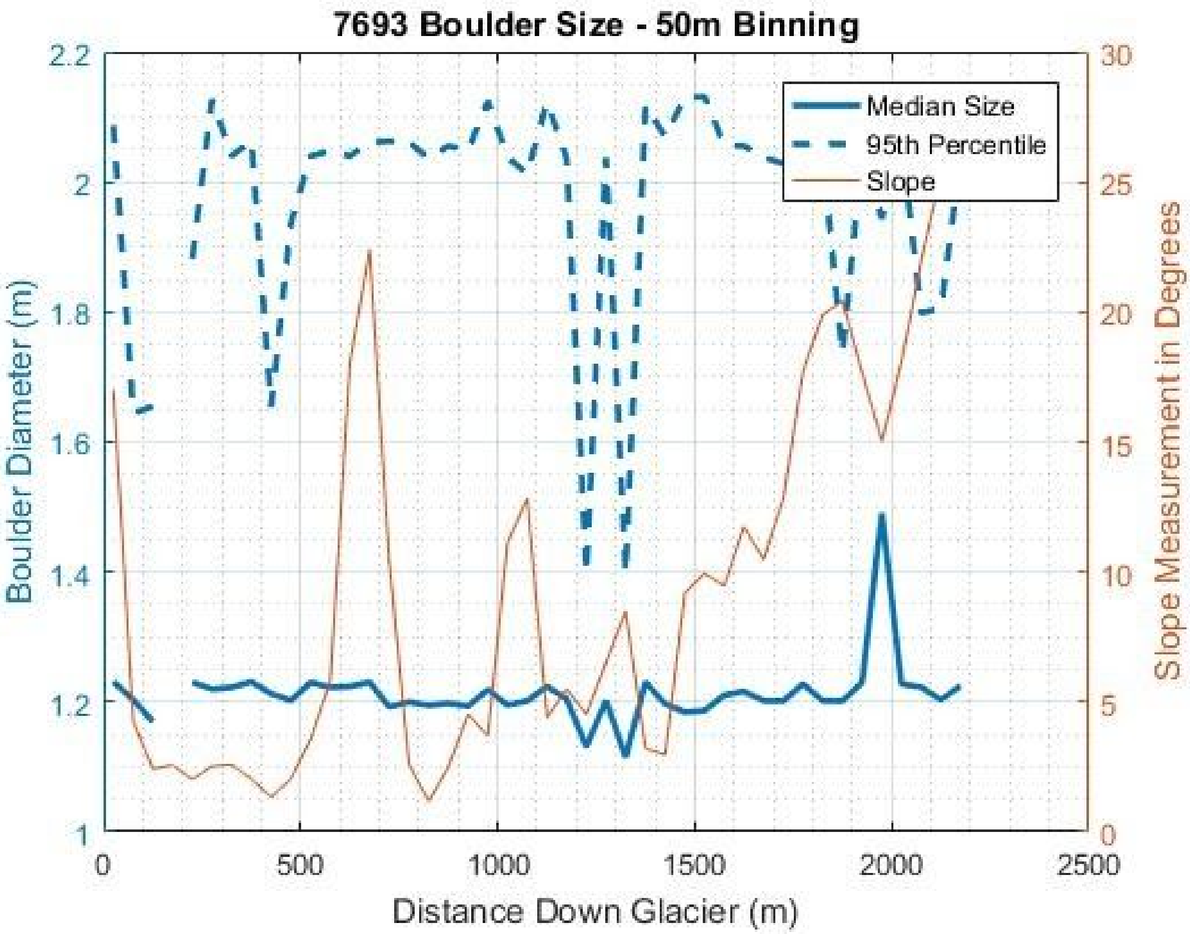
7693 Boulder Size - 50m Binning
Boulder Diameter (m)
Slope Measurement in Degrees
Distance Down Glacier (m)
Median Size
95th Percentile
Slope
2.2
2
1.8
1.6
1.4
1.2
1
30
25
20
15
10
5
0
0
500
1000
1500
2000
2500

D5

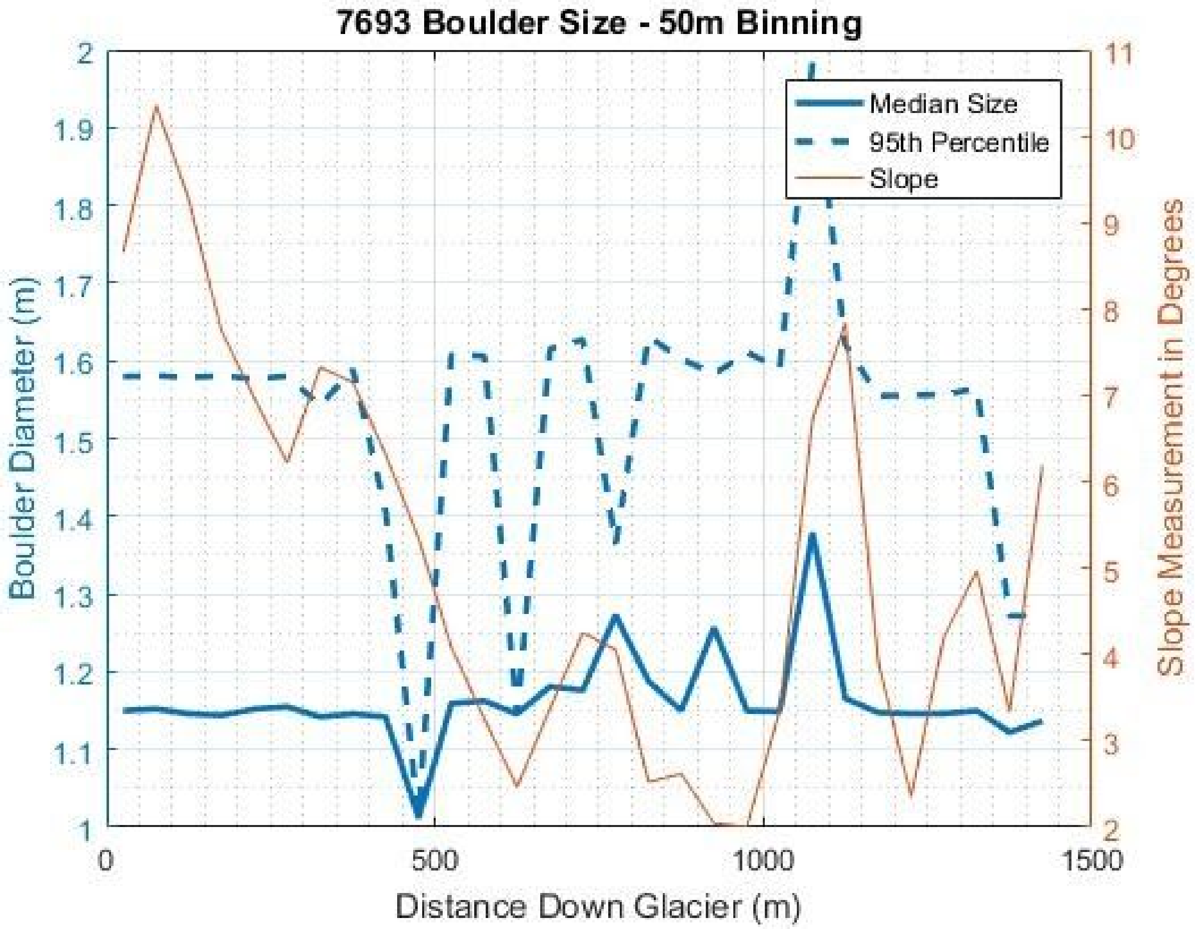
7693 Boulder Size - 50m Binning
Median Size
95th Percentile
Slope
Boulder Diameter (m)
Slope Measurement in Degrees
Distance Down Glacier (m)
2
1.9
1.8
1.7
1.6
1.5
1.4
1.3
1.2
1.1
1
11
10
9
8
7
6
5
4
3
2
0
500
1000
1500

F_ESP_023937_2240_PSP_010487_2240

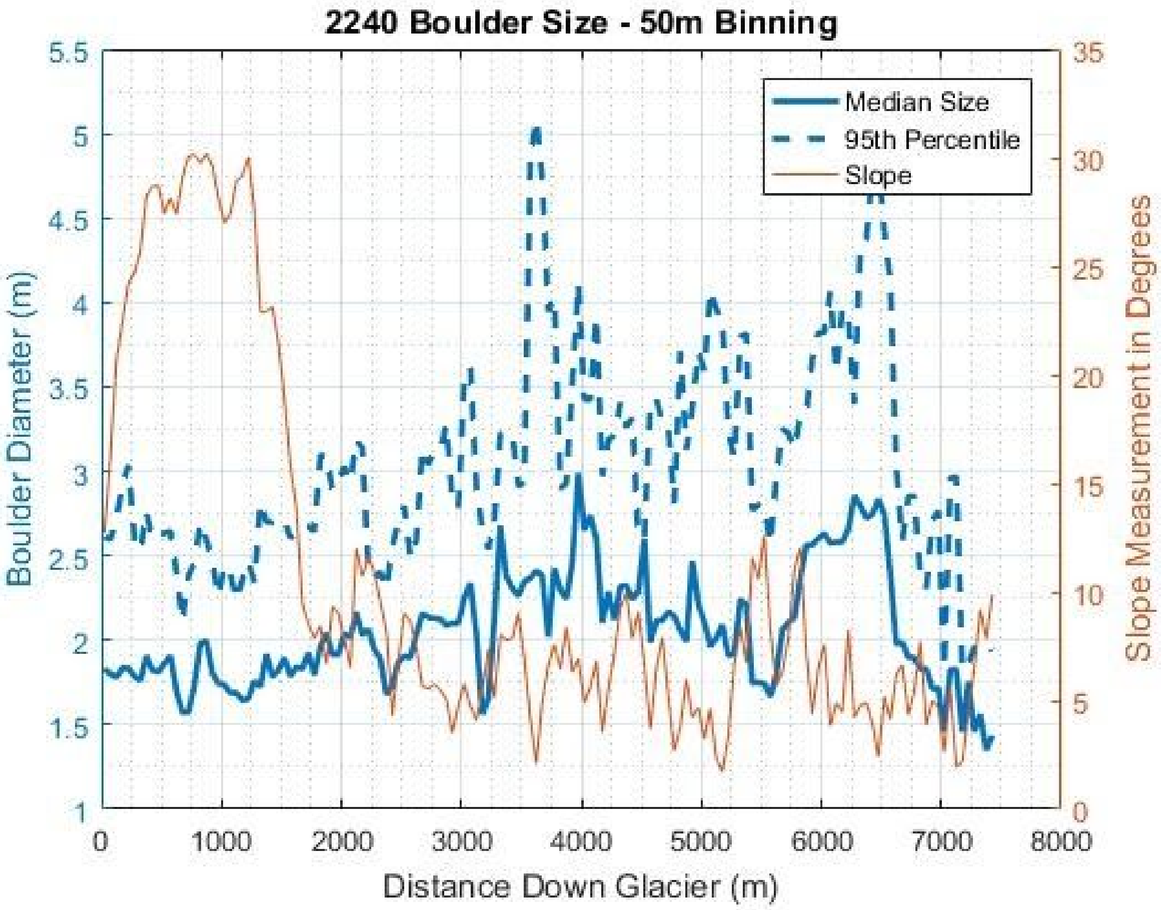

G_PSP_009956_2160_PSP_007016_2160

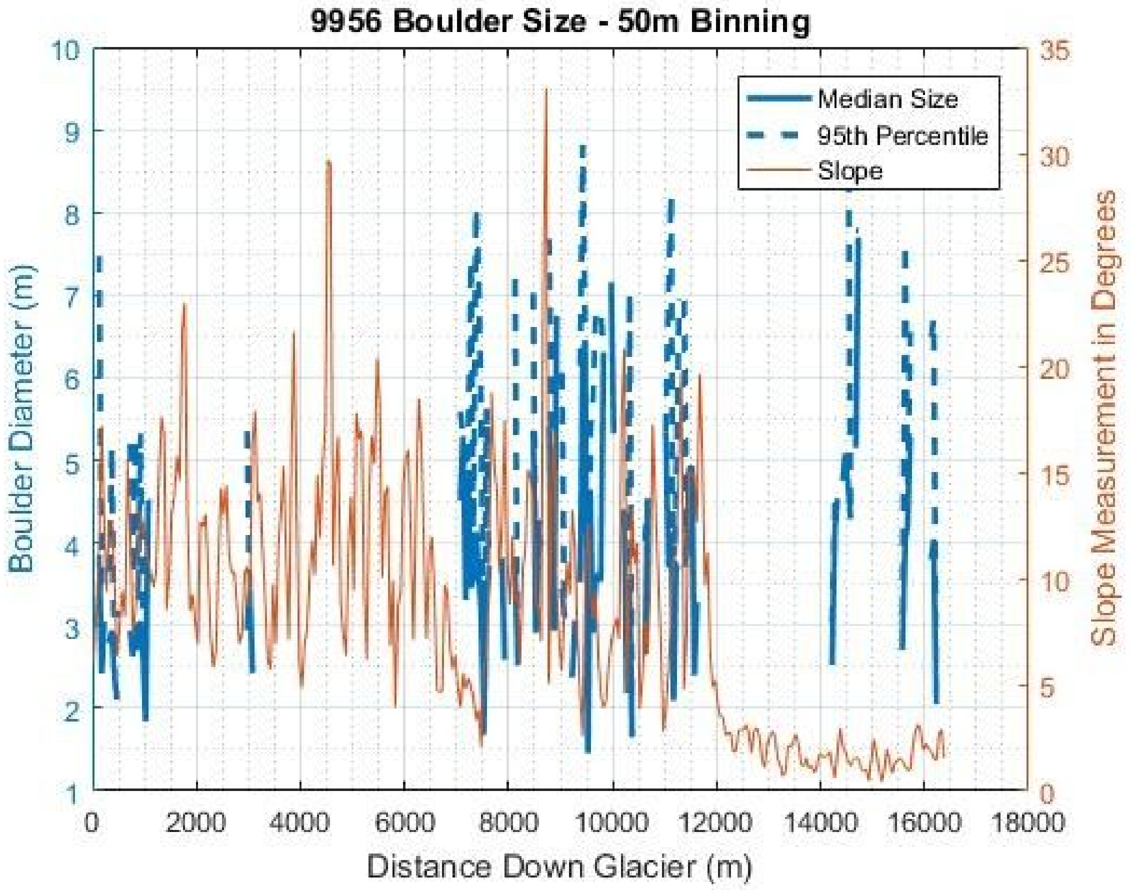

I_PSP_007795_2175_PSP_009588_2175

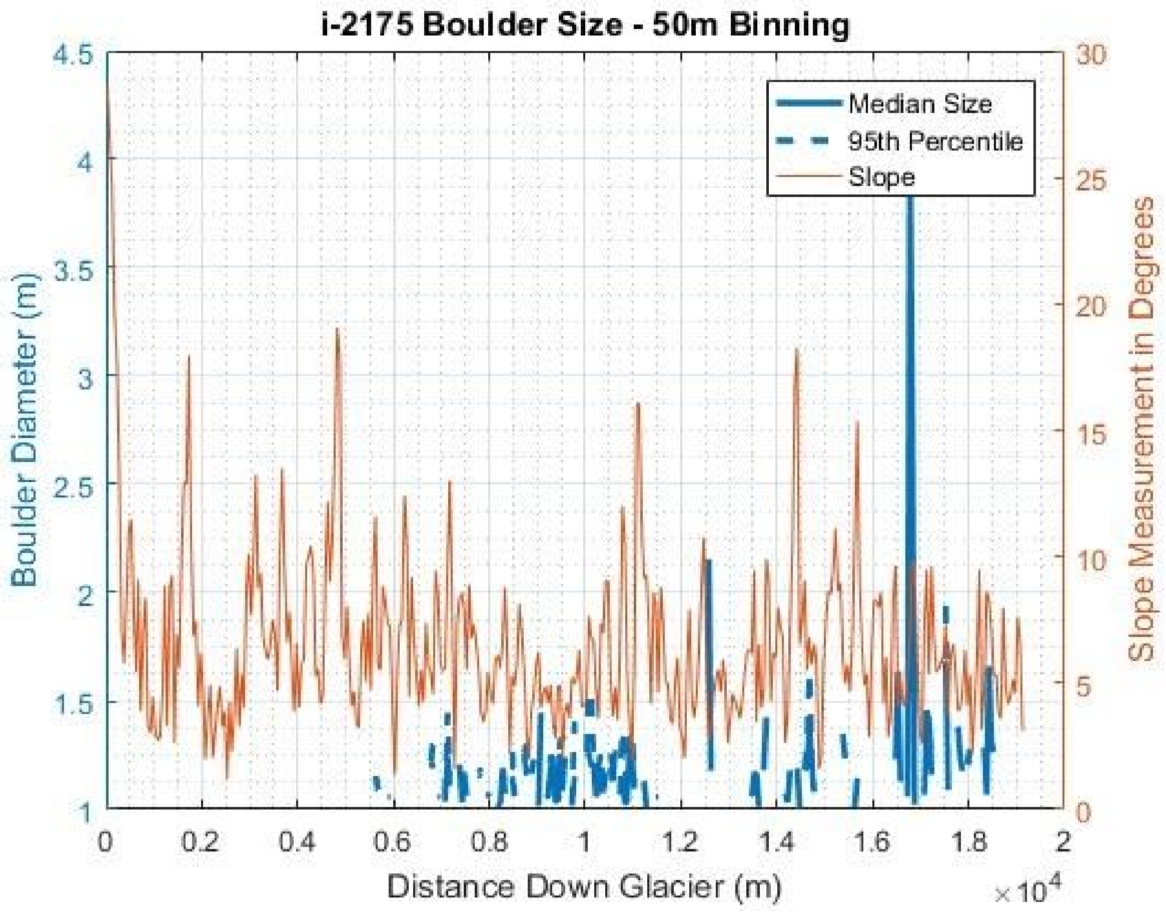

J_ESP_016132_2300_ESP_016277_2300

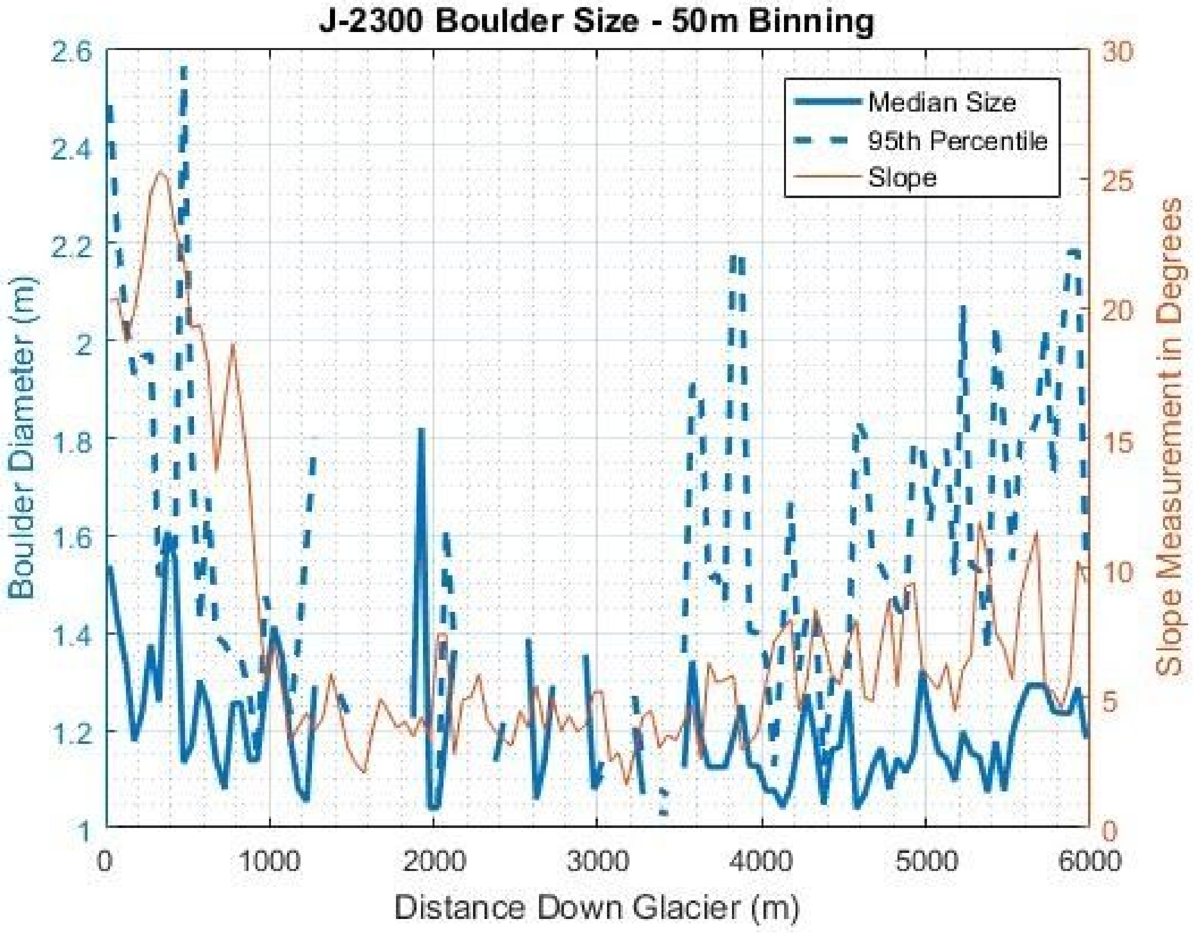

K_PSP_001357_2300

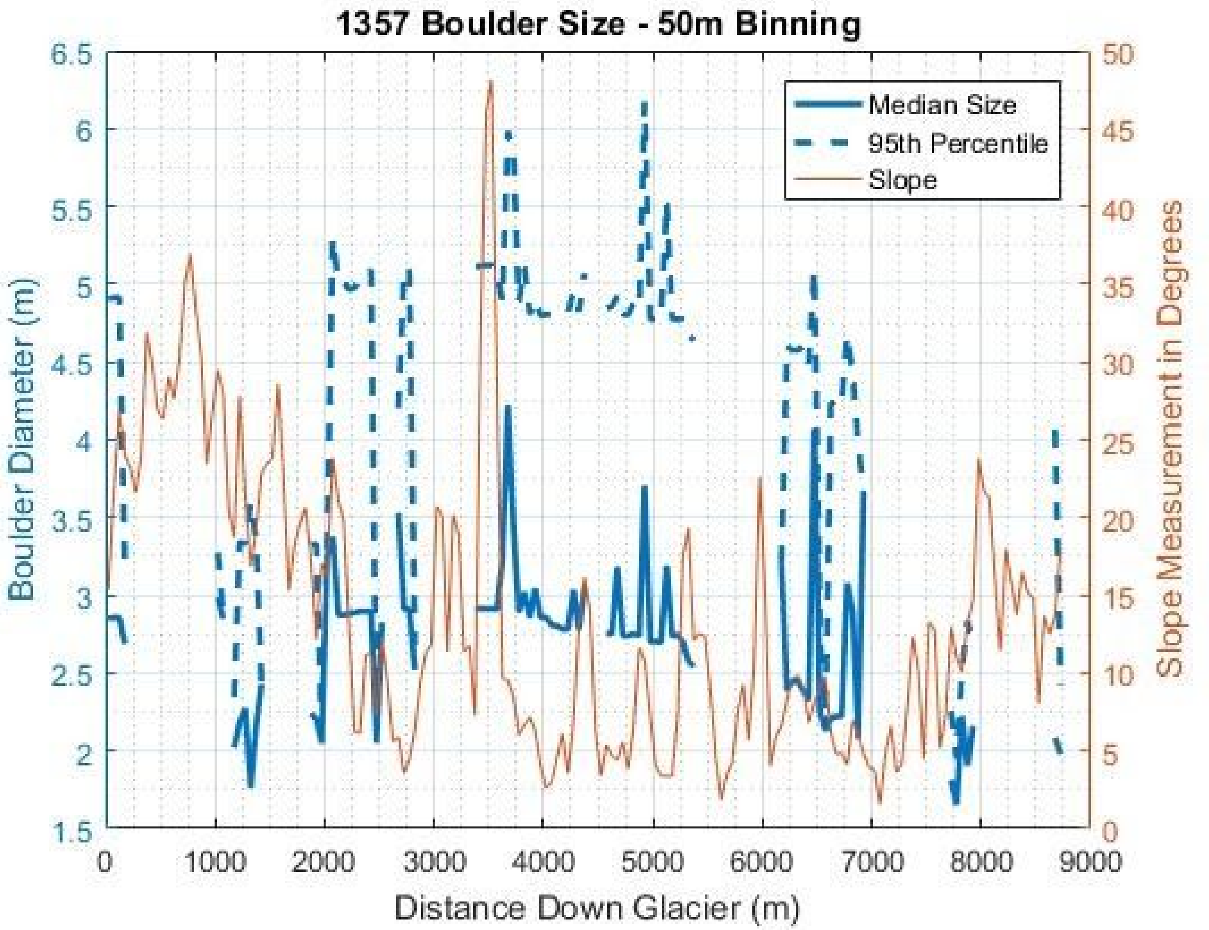

L_ESP_016271_1475_ESP_016416_1475 --- Michelle Tebolt

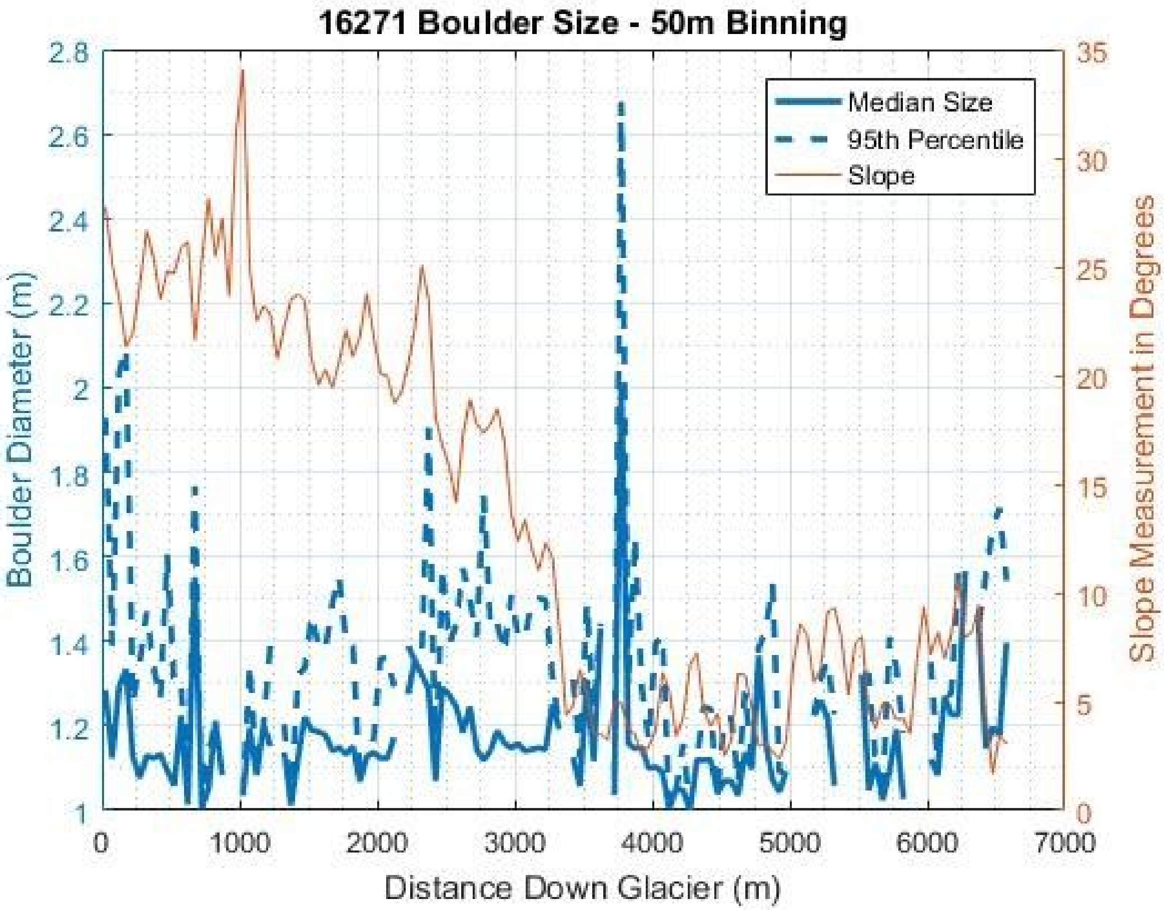

N_ESP_033653_2225_ESP_33363_2225 - Michelle

Track 2

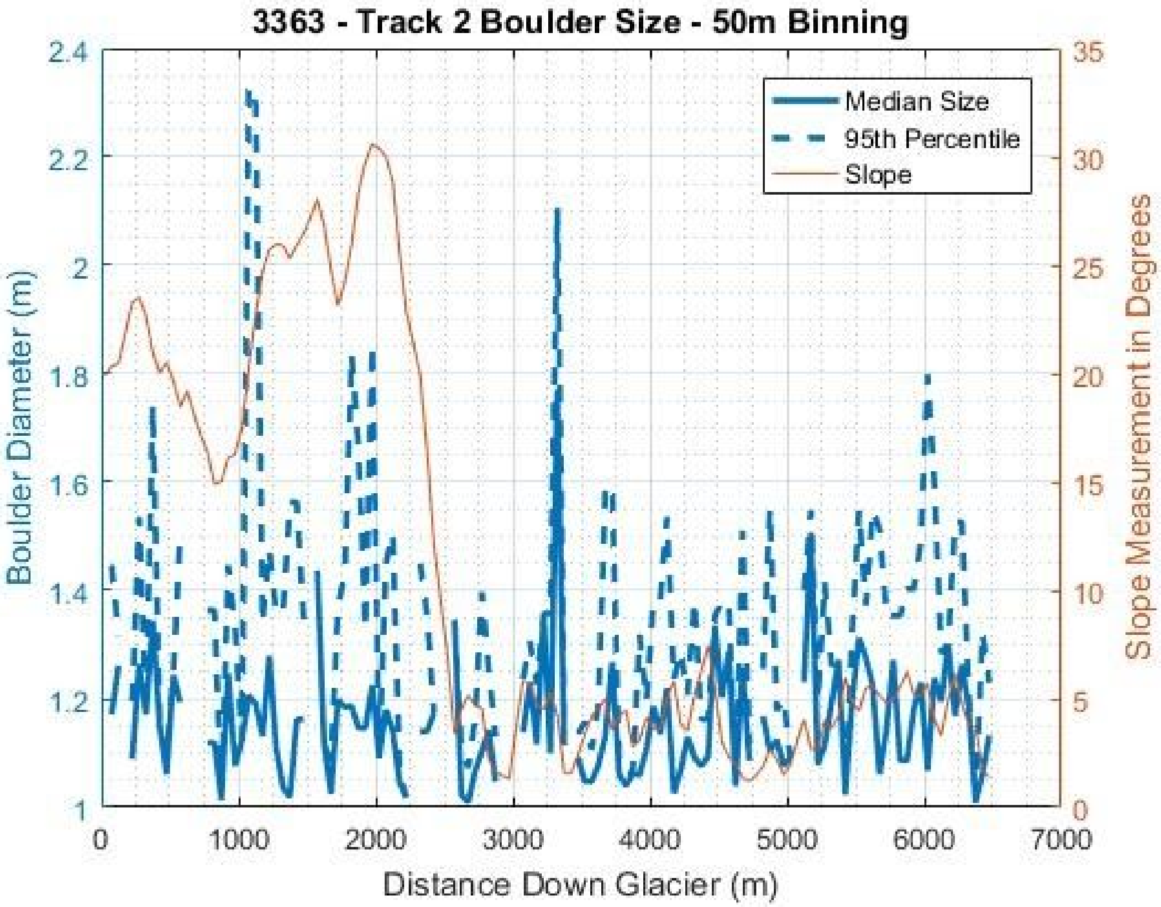

P_PSP_001882_1410_PSP_001816_1410

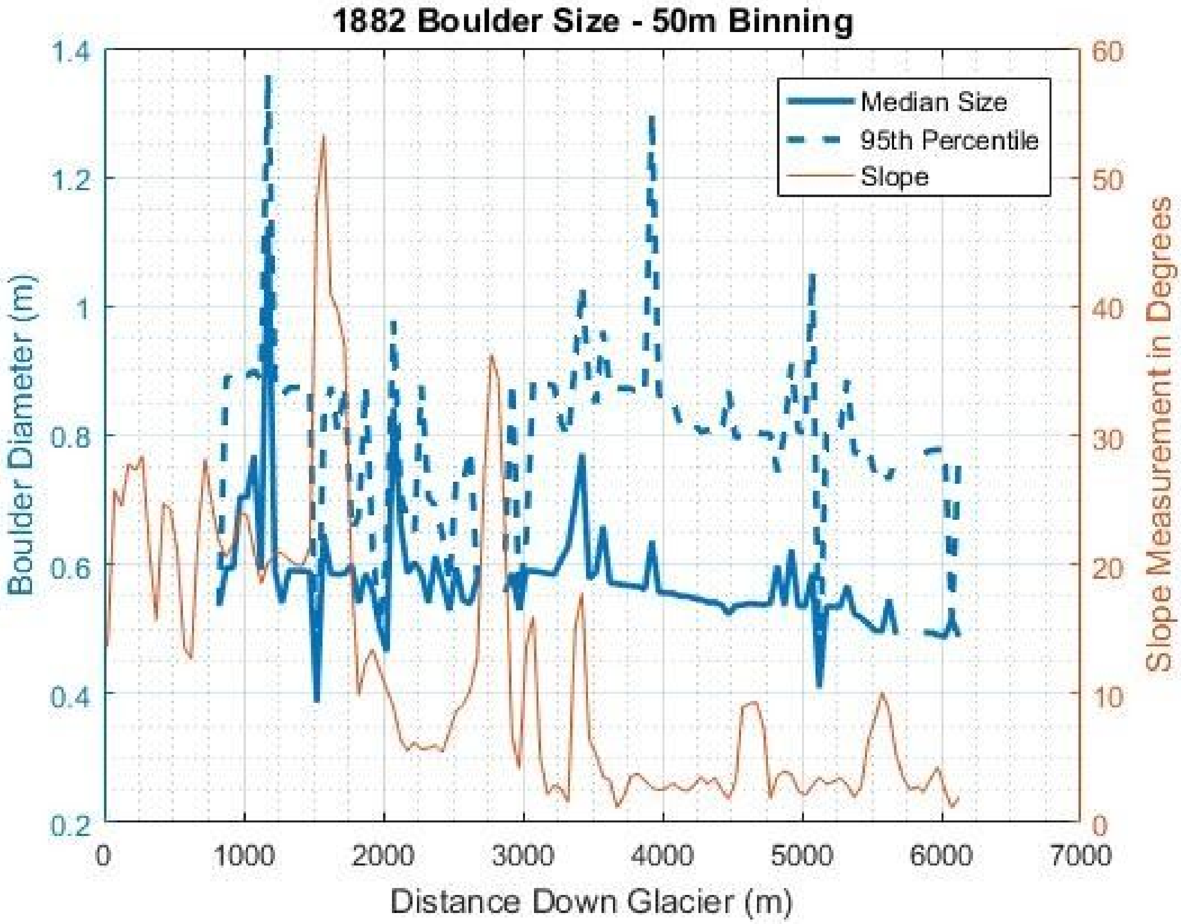

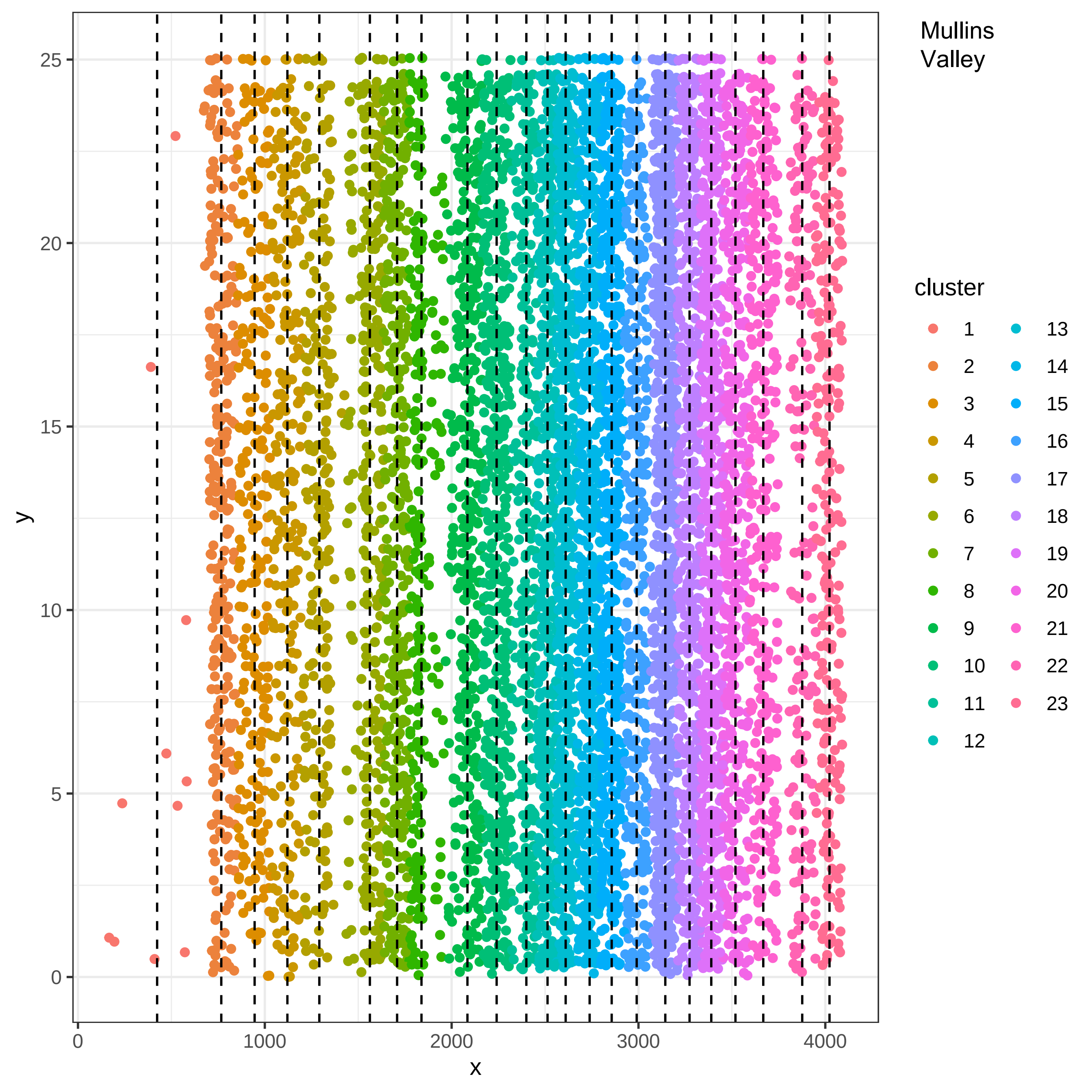

Mullins Valley
y
x
0
5
10
15
20
25
0
1000
2000
3000
4000
cluster
1
2
3
4
5
6
7
8
9
10
11
12
13
14
15
16
17
18
19
20
21
22
23

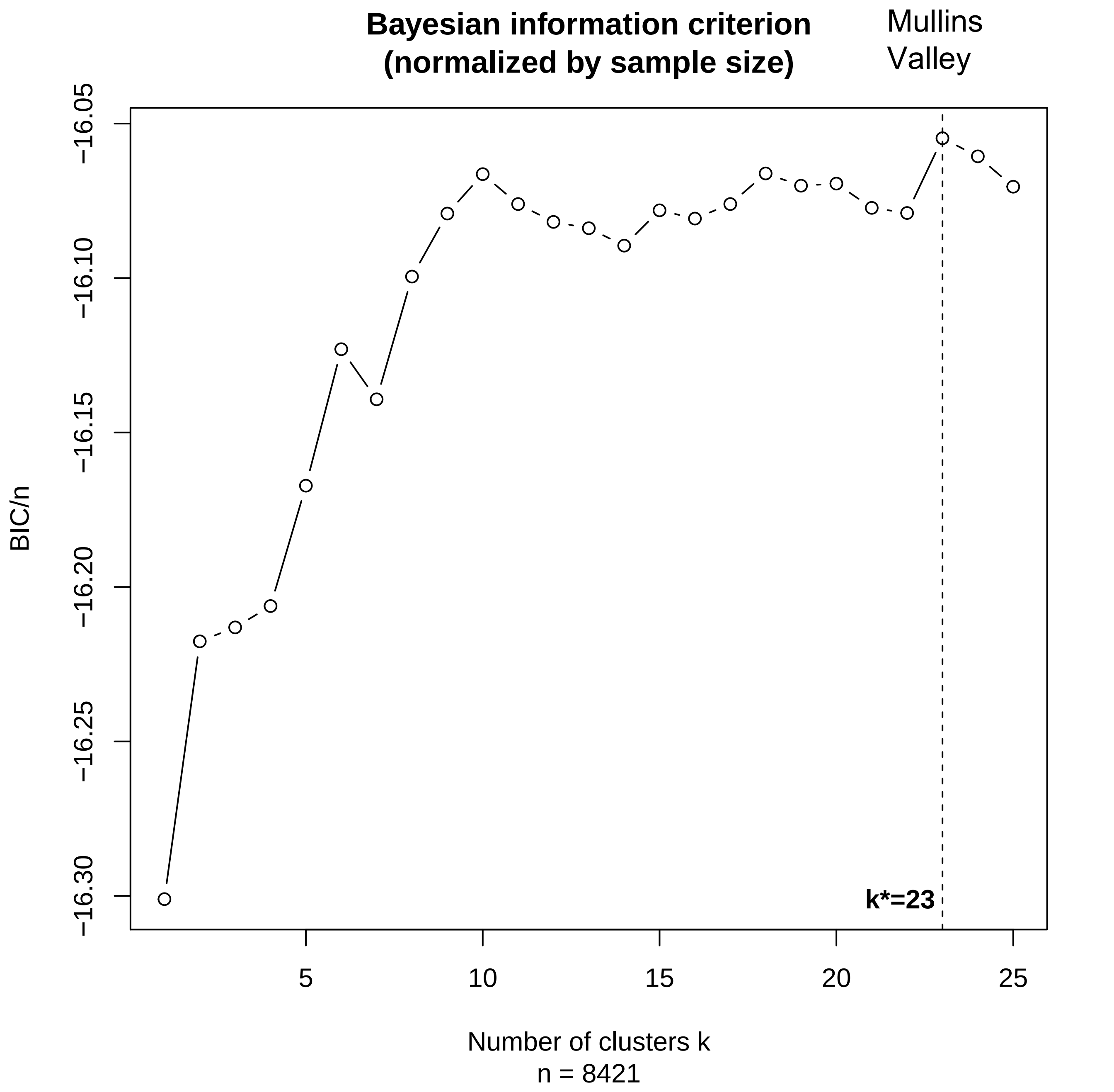

Bayesian information criterion
(normalized by sample size)
Mullins
Valley
−16.05
−16.10
−16.15
−16.20
−16.25
−16.30
BIC/n
k*=23
5
10
15
20
25
Number of clusters k
n = 8421

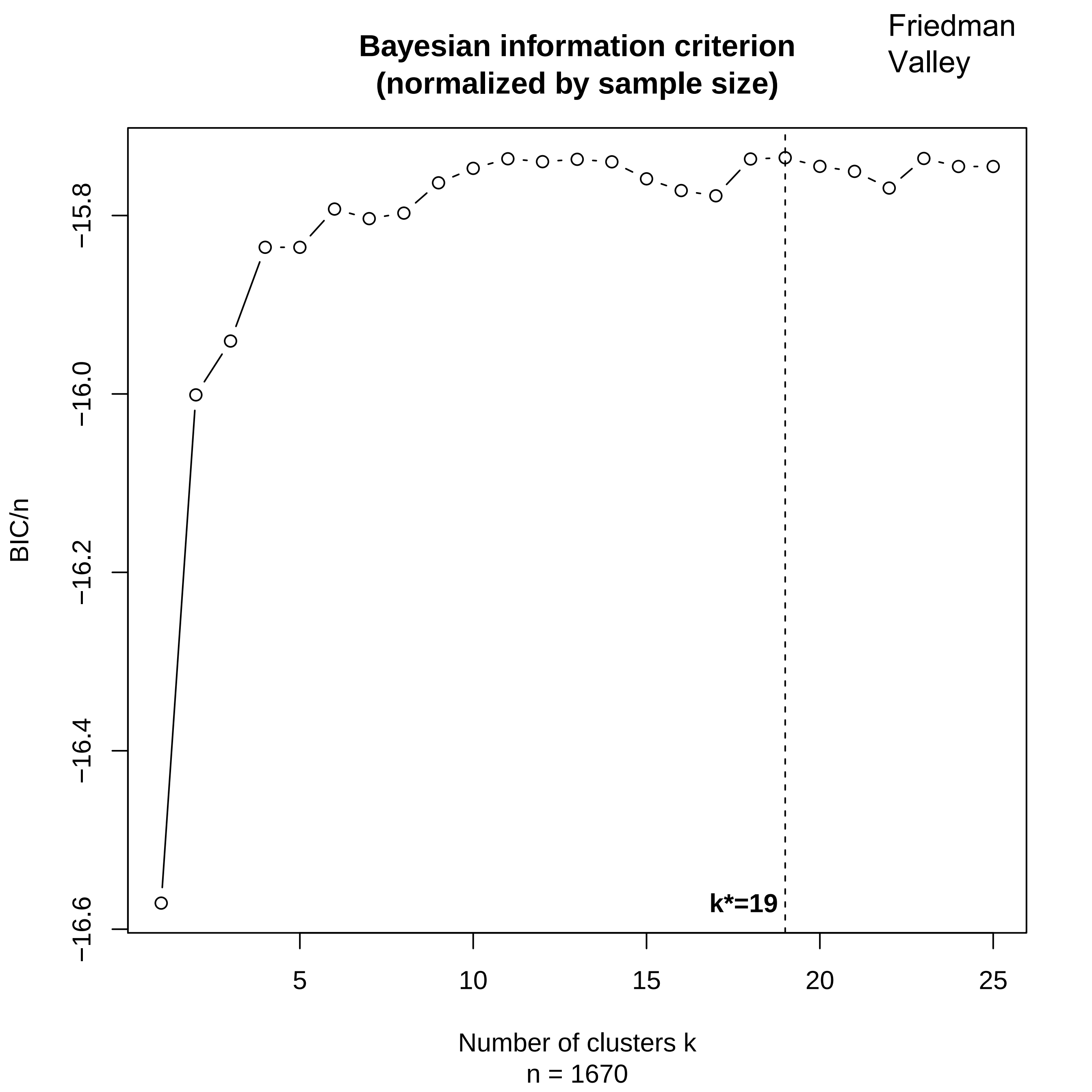
Bayesian information criterion
(normalized by sample size)
Friedman
Valley
BIC/n
−15.8
−16.0
−16.2
−16.4
−16.6
k*=19
5
10
15
20
25
Number of clusters k
n = 1670

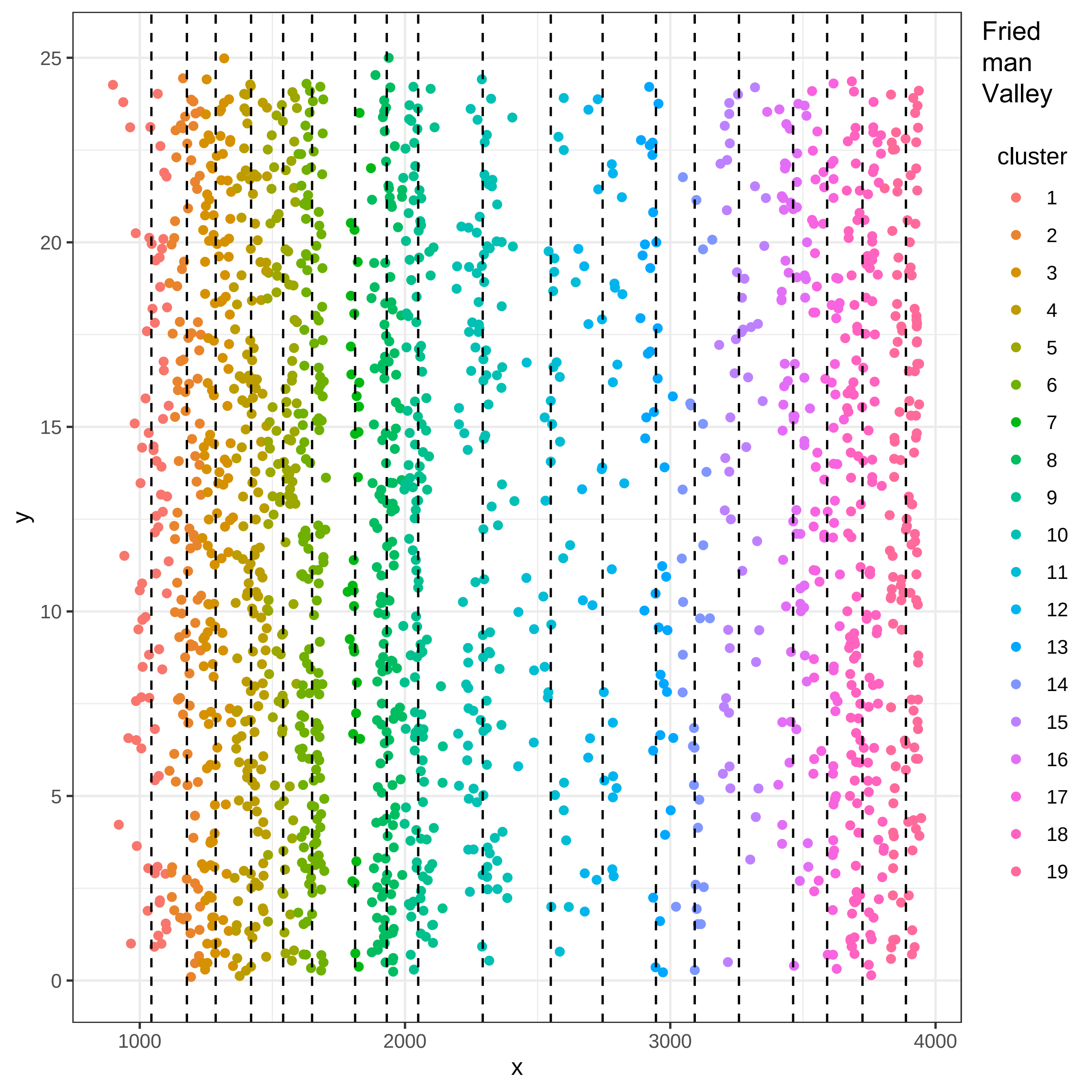
Fried man Valley
cluster
1
2
3
4
5
6
7
8
9
10
11
12
13
14
15
16
17
18
19
y
x
0
5
10
15
20
25
1000
2000
3000
4000

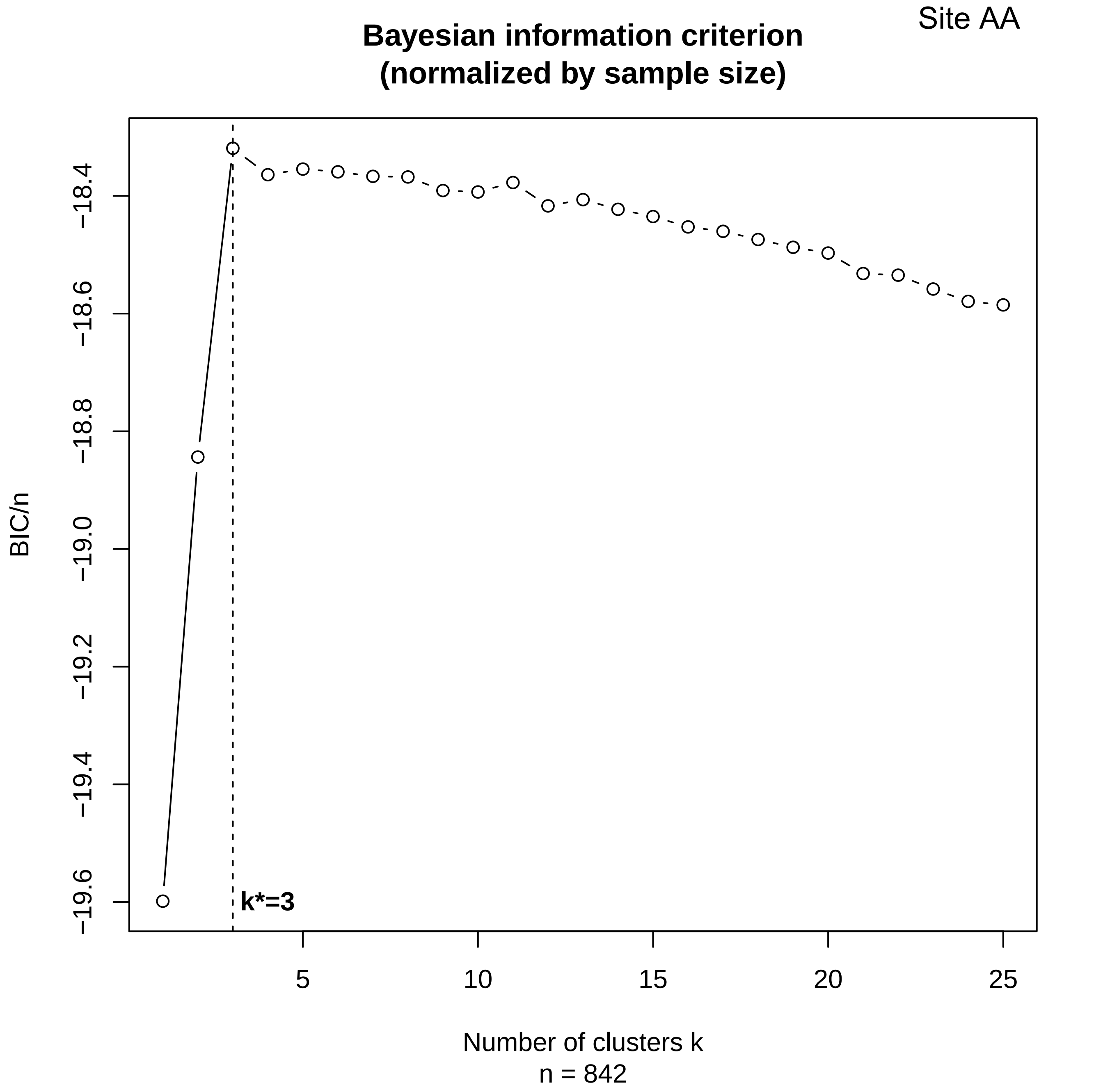

Site AA
Bayesian information criterion
(normalized by sample size)
BIC/n
−18.4
−18.6
−18.8
−19.0
−19.2
−19.4
−19.6
k*=3
5
10
15
20
25
Number of clusters k
n = 842

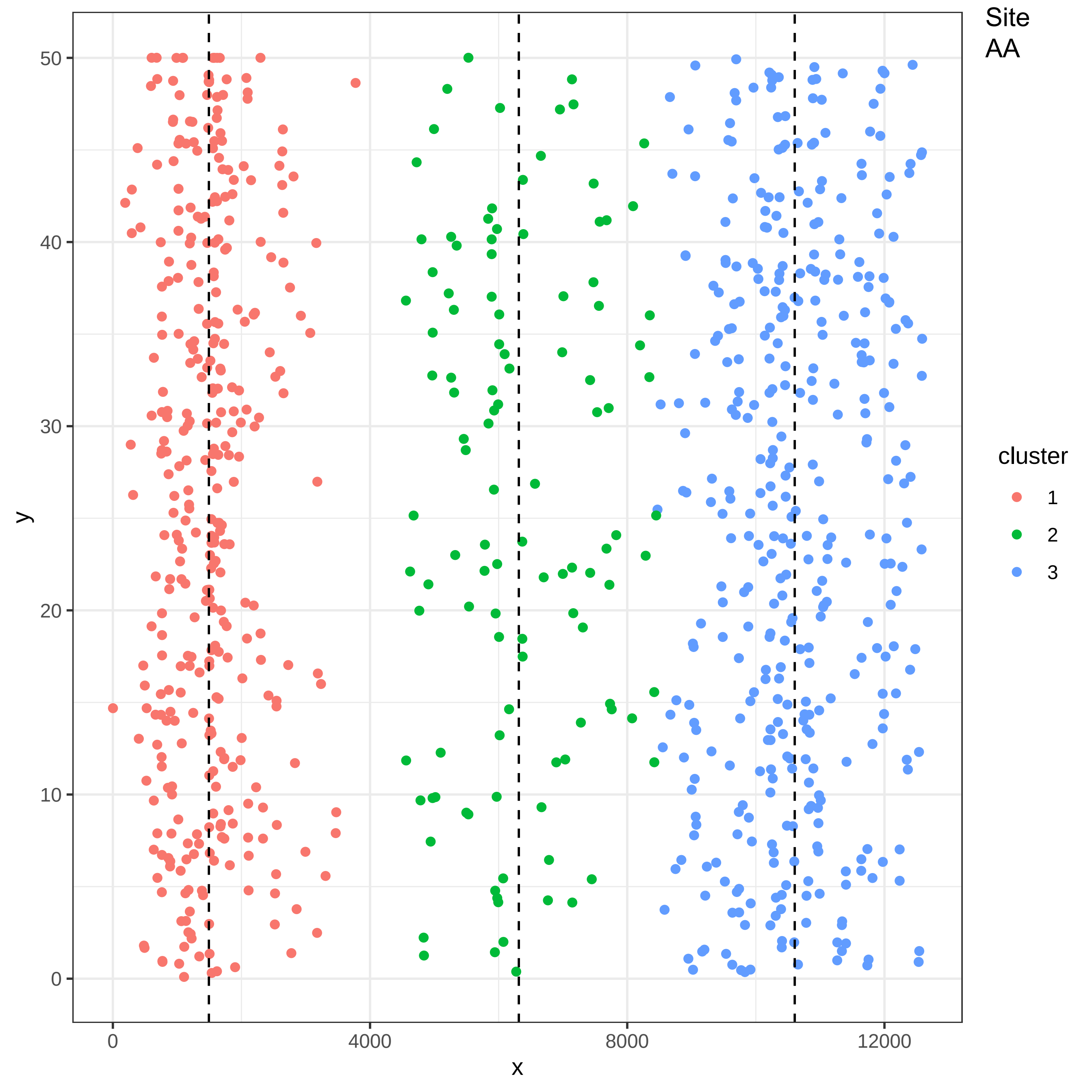

Site
AA
cluster
1
2
3
y
x
0
10
20
30
40
50
0
4000
8000
12000

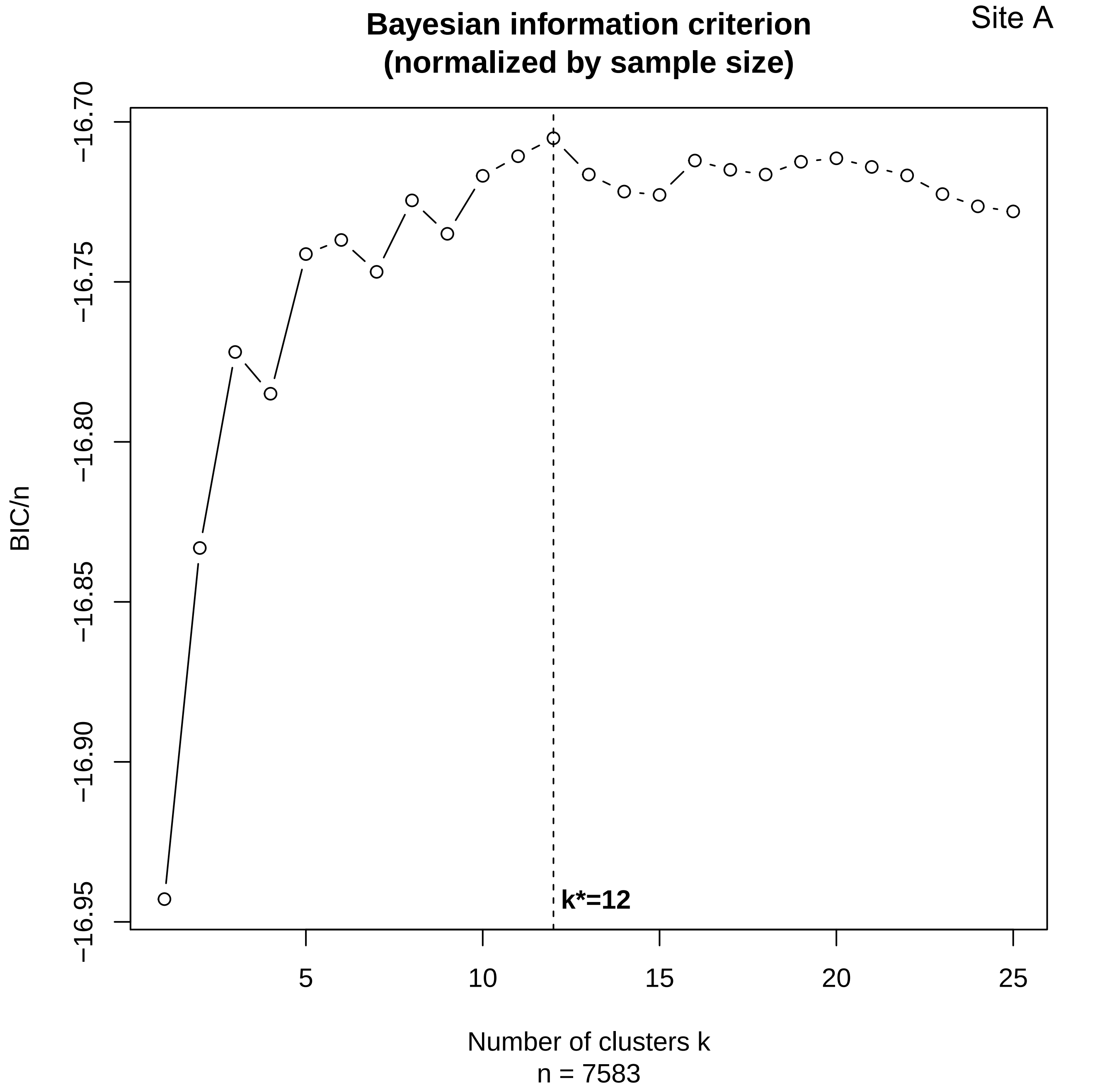

Bayesian information criterion
(normalized by sample size)
Site A
BIC/n
−16.70
−16.75
−16.80
−16.85
−16.90
−16.95
k*=12
5
10
15
20
25
Number of clusters k
n = 7583

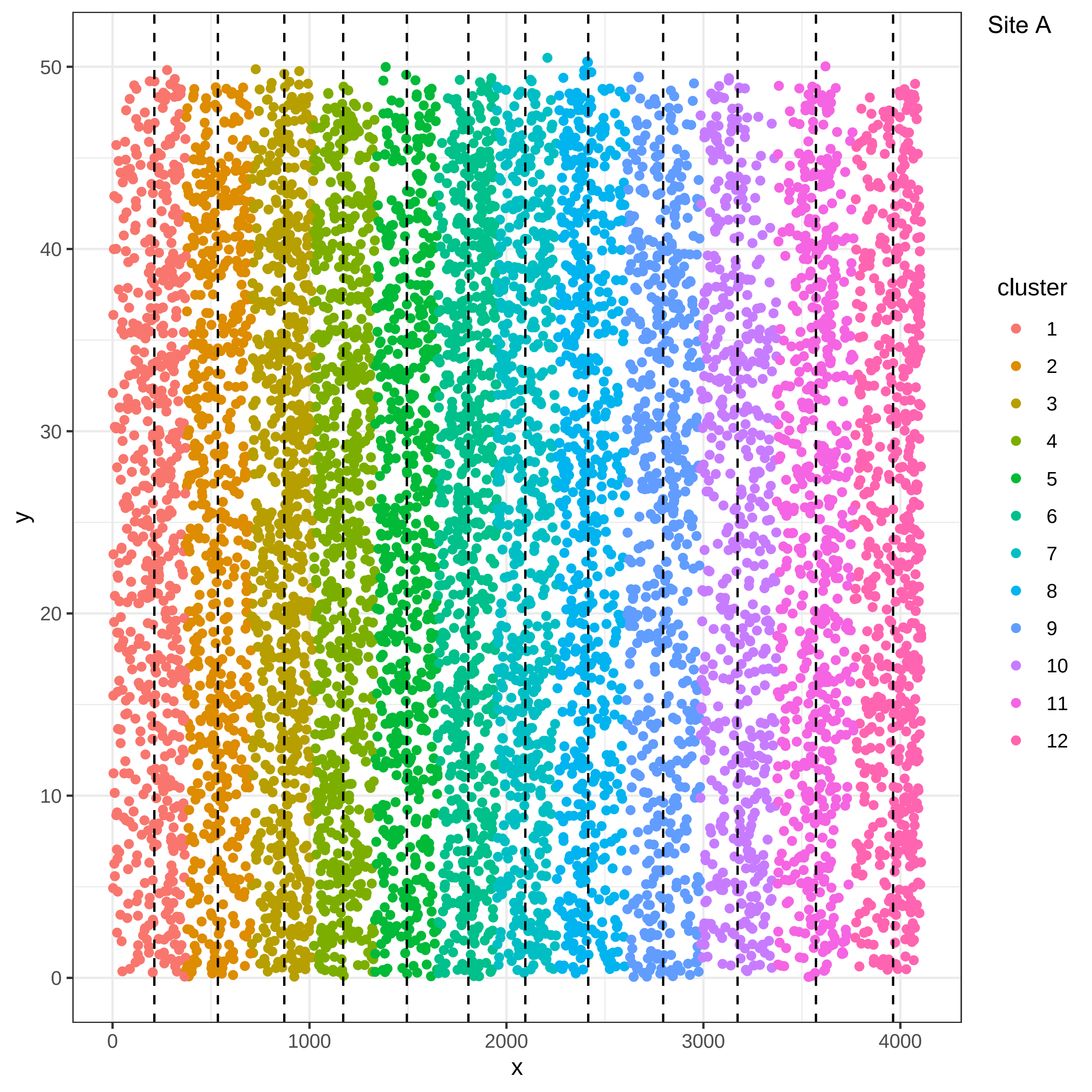

Site A
cluster
1
2
3
4
5
6
7
8
9
10
11
12
y
x
0
10
20
30
40
50
0
1000
2000
3000
4000

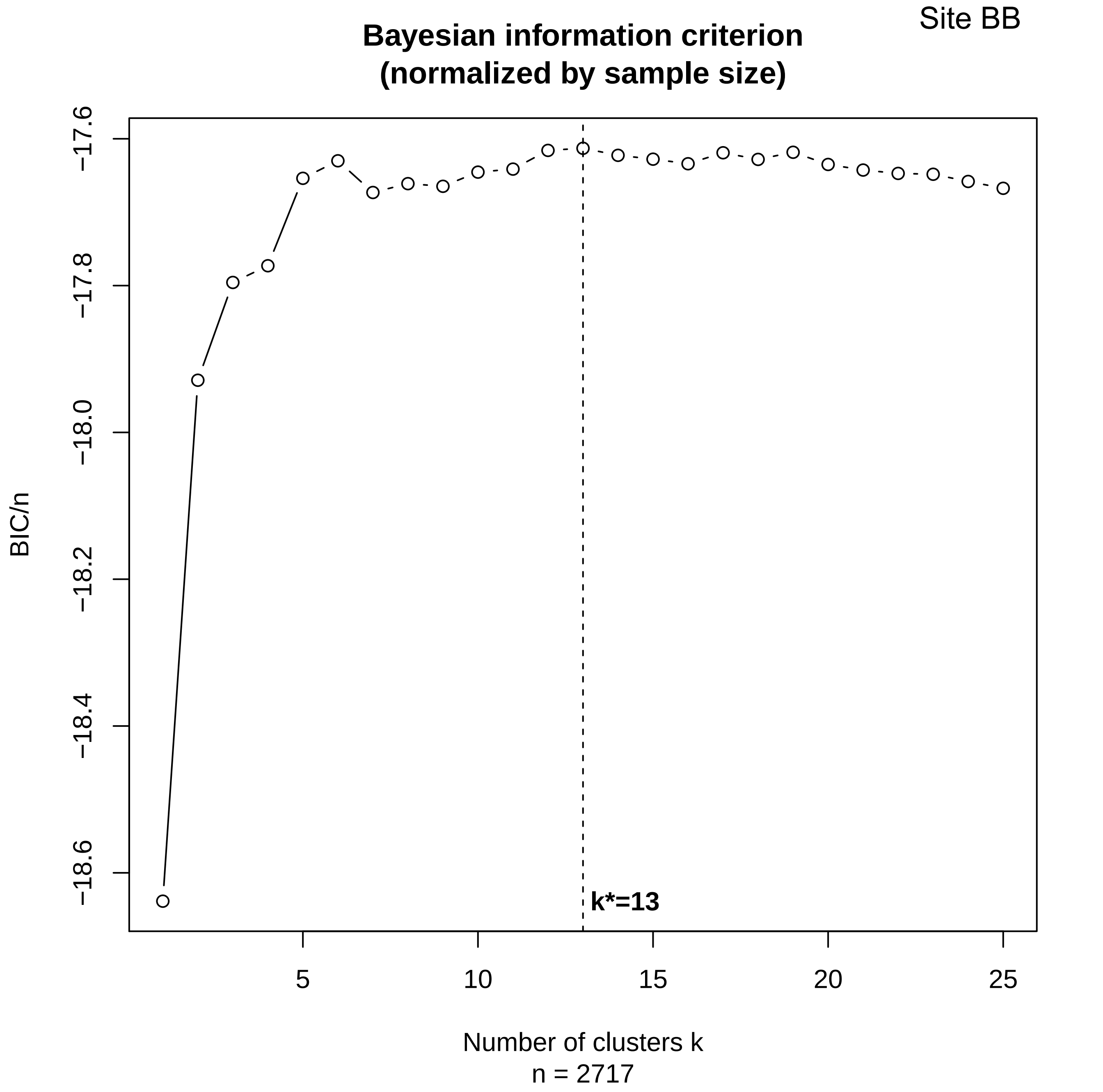

Site BB
Bayesian information criterion
(normalized by sample size)
−17.6
−17.8
−18.0
−18.2
−18.4
−18.6
BIC/n
k*=13
5
10
15
20
25
Number of clusters k
n = 2717

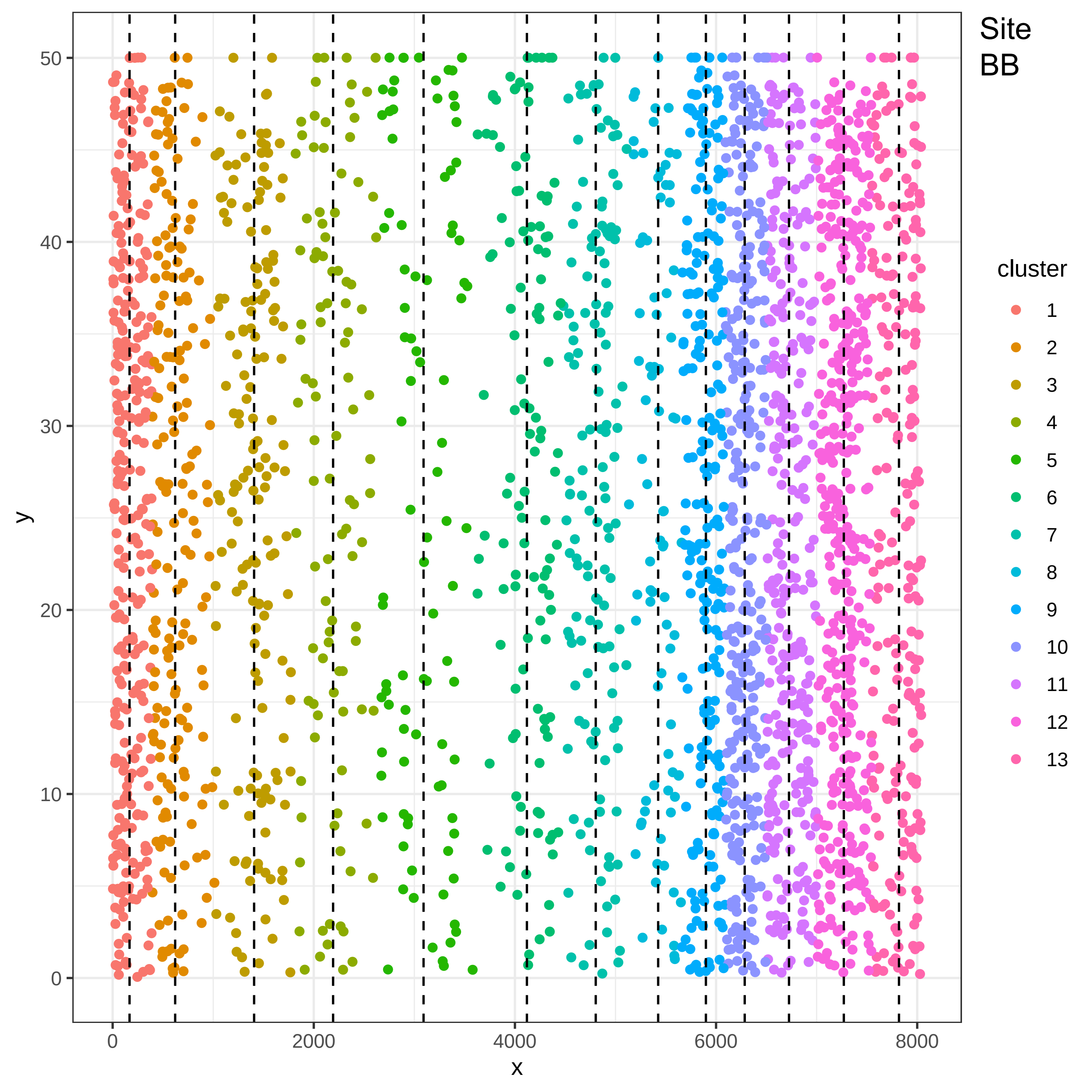
Site
BB
cluster
1
2
3
4
5
6
7
8
9
10
11
12
13
y
x
0
10
20
30
40
50
0
2000
4000
6000
8000

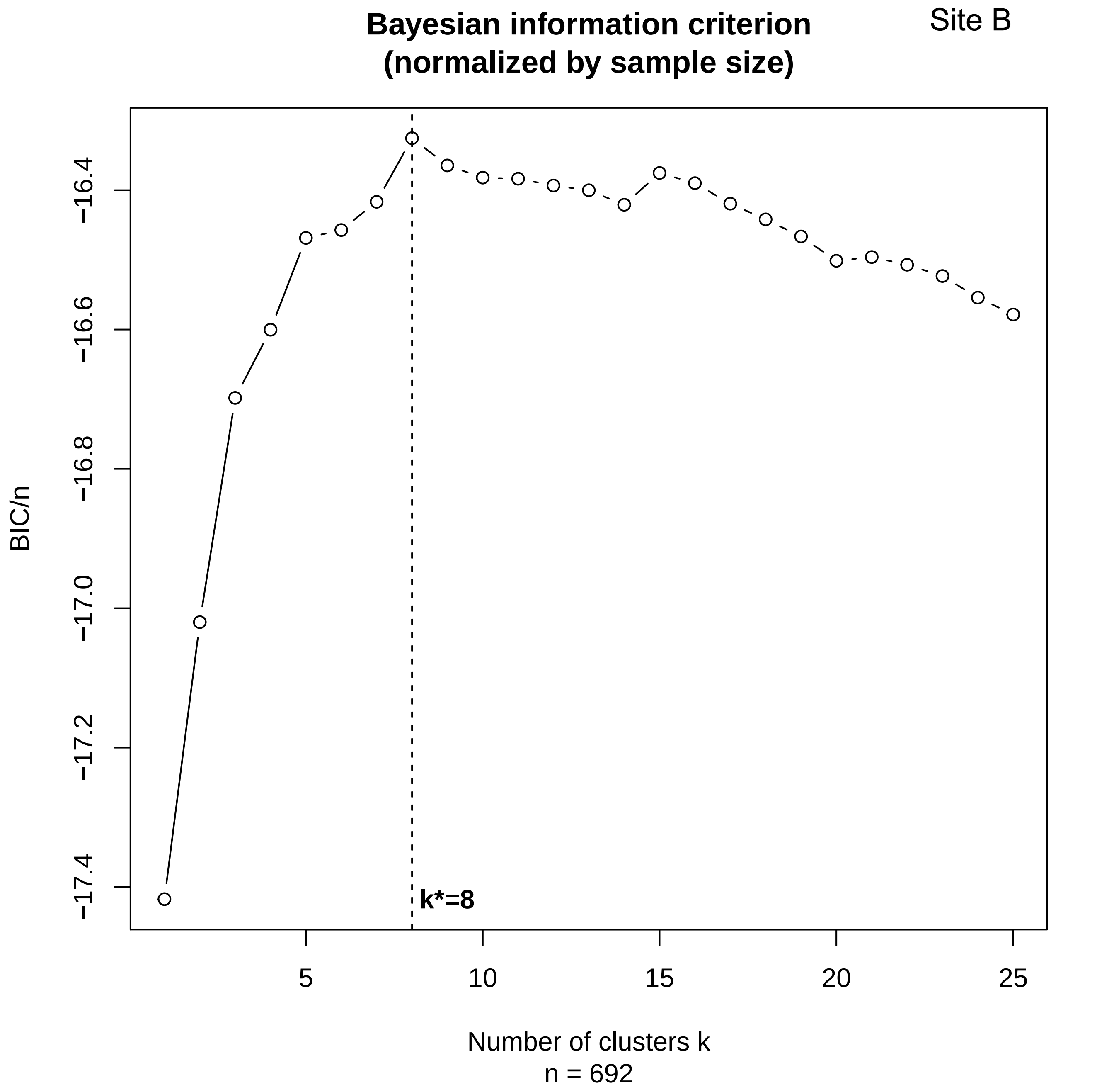

Bayesian information criterion
(normalized by sample size)
Site B
BIC/n
−16.4
−16.6
−16.8
−17.0
−17.2
−17.4
k*=8
5
10
15
20
25
Number of clusters k
n = 692

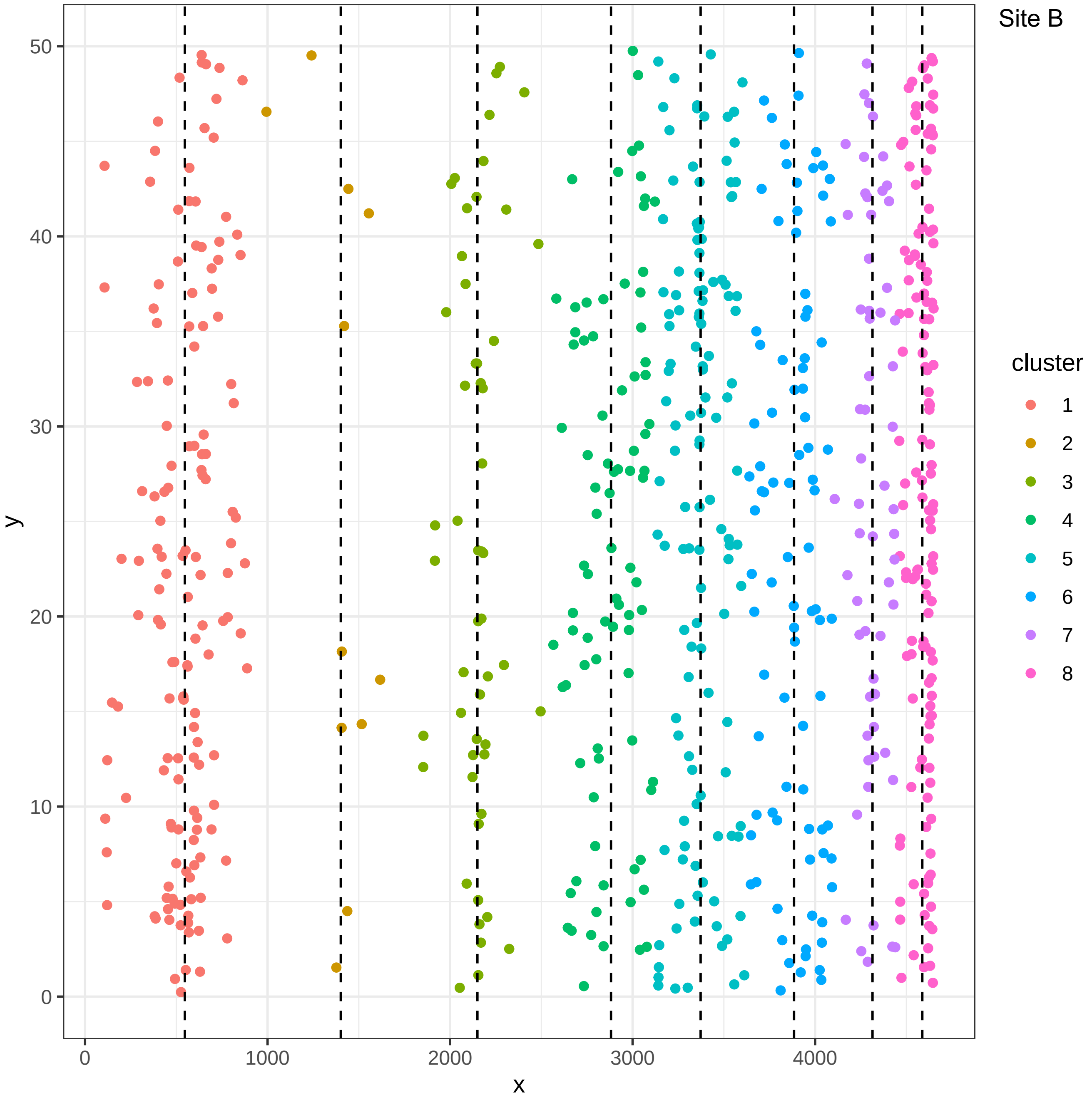
Site B
y
x
0
1000
2000
3000
4000
0
10
20
30
40
50
cluster
1
2
3
4
5
6
7
8

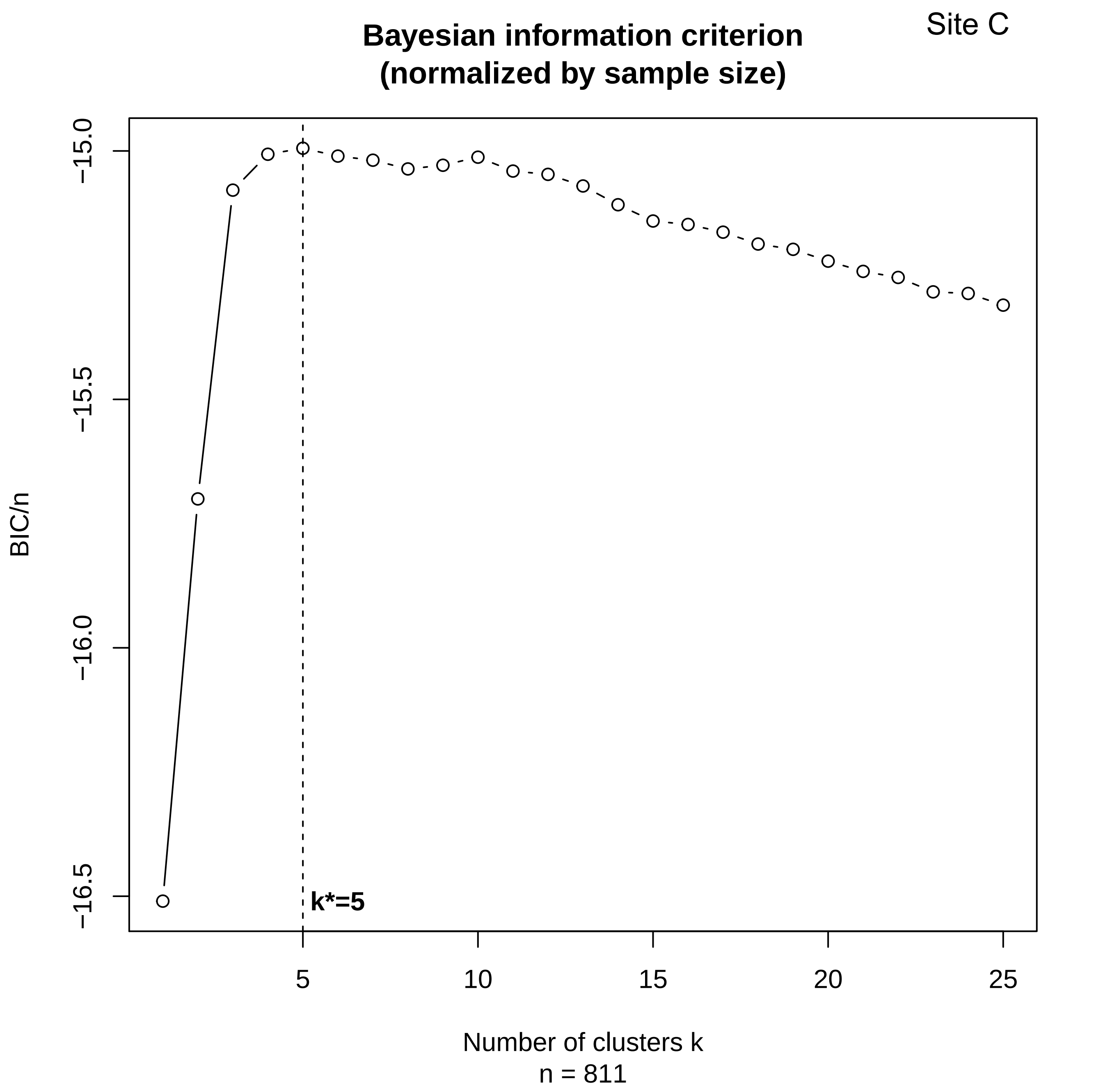
Site C
Bayesian information criterion
(normalized by sample size)
−15.0
−15.5
−16.0
−16.5
BIC/n
k*=5
5
10
15
20
25
Number of clusters k
n = 811

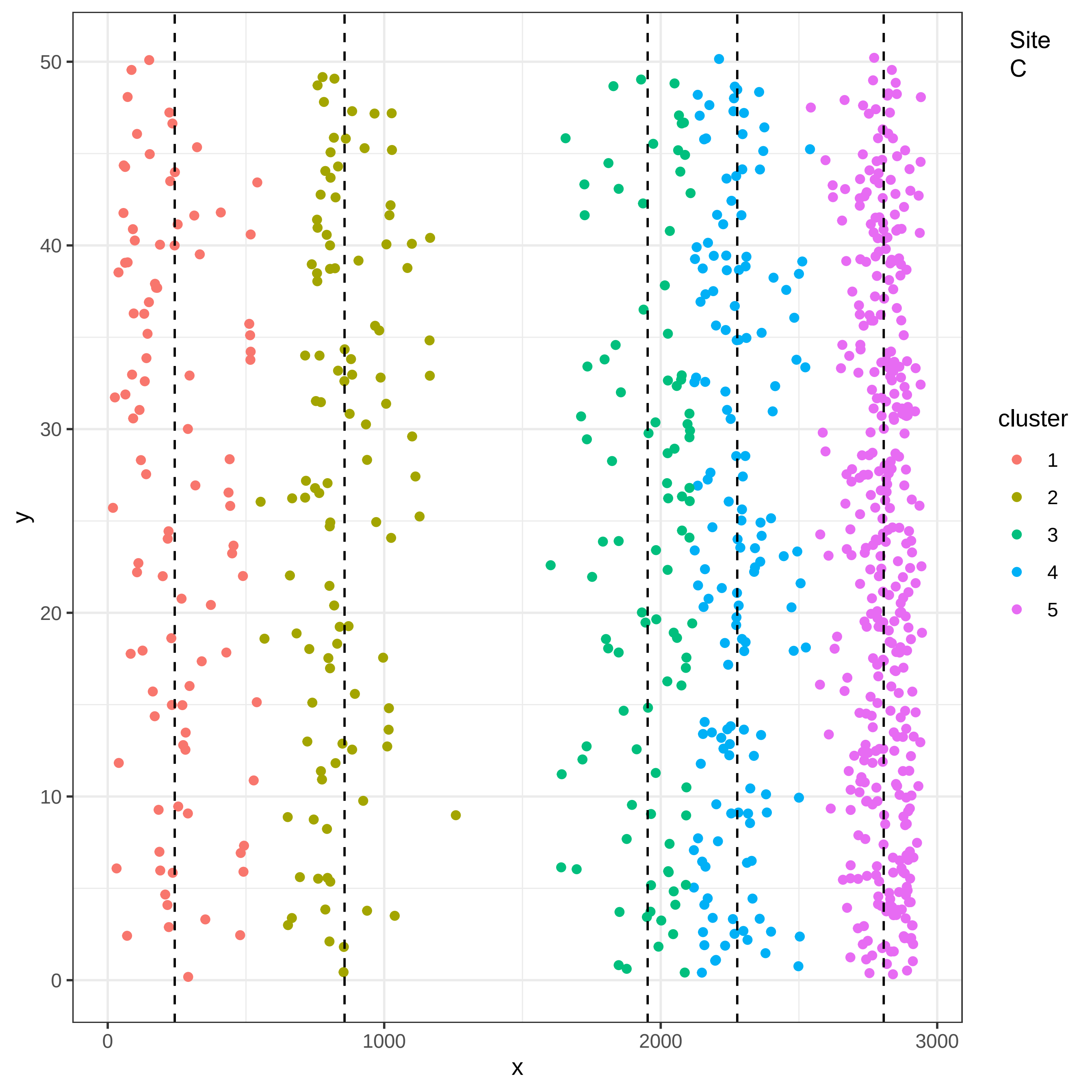

Site
C
cluster
1
2
3
4
5
y
x
0
10
20
30
40
50
0
1000
2000
3000

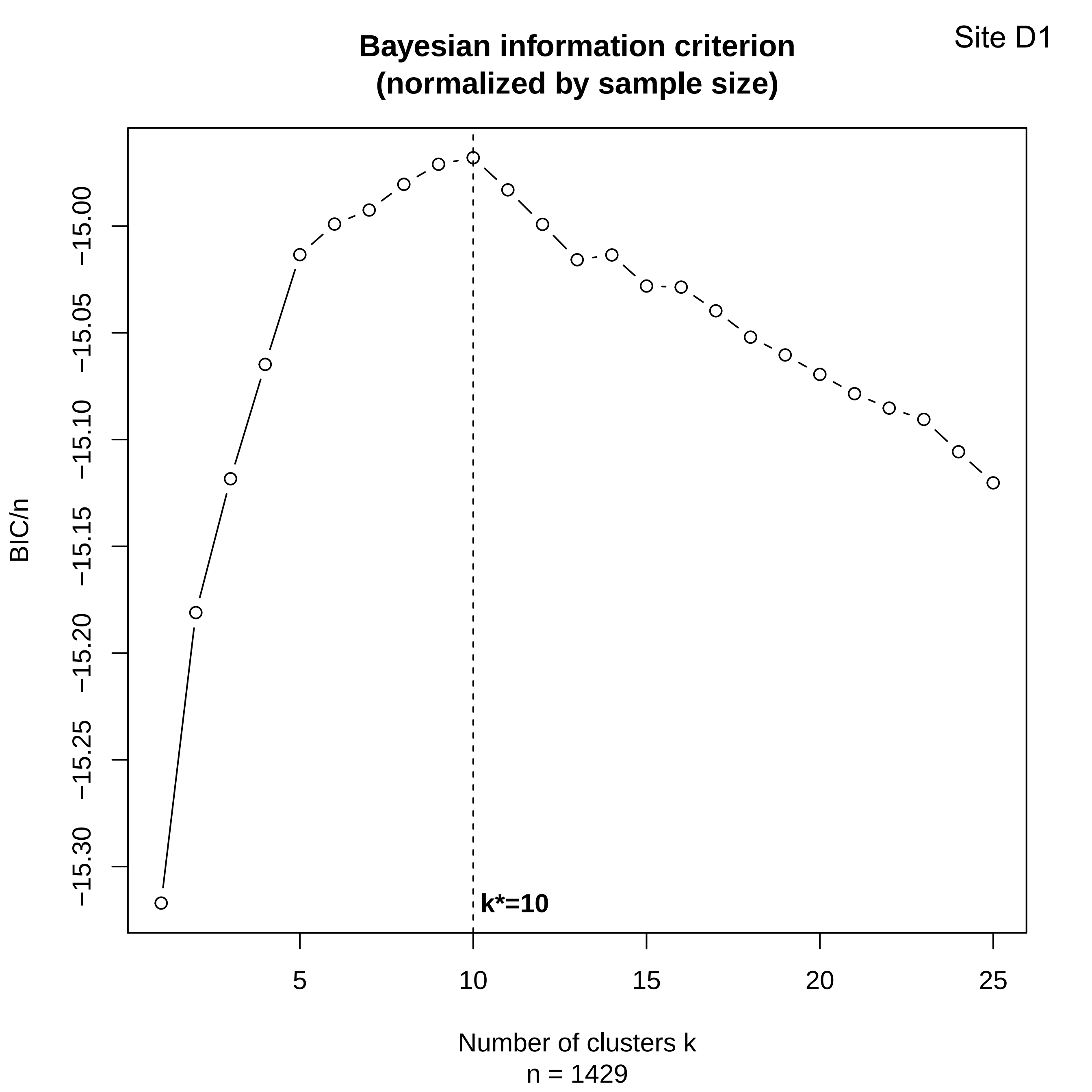

Site D1
Bayesian information criterion
(normalized by sample size)
BIC/n
−15.00
−15.05
−15.10
−15.15
−15.20
−15.25
−15.30
k*=10
5
10
15
20
25
Number of clusters k
n = 1429

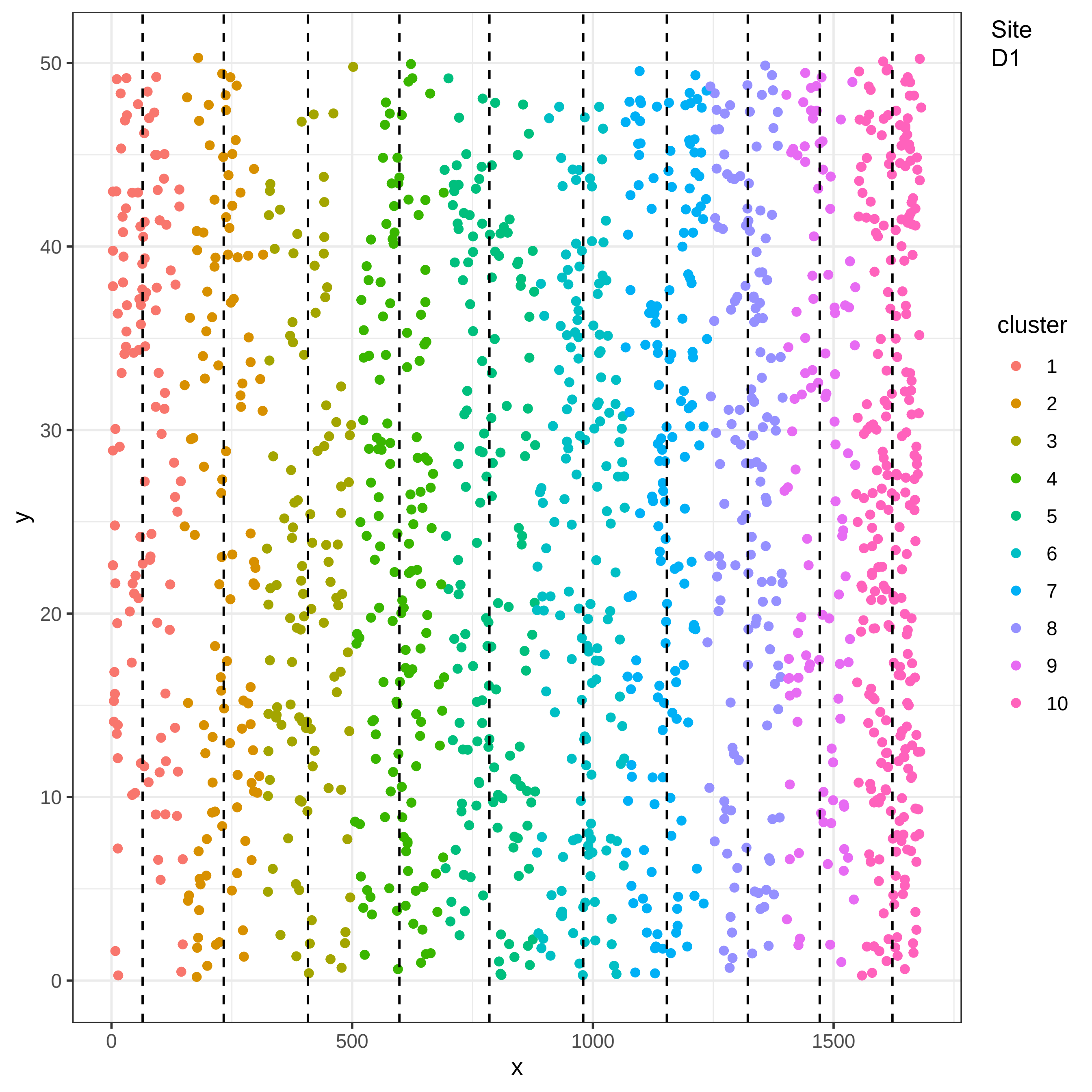

Site
D1
cluster
1
2
3
4
5
6
7
8
9
10
0
10
20
30
40
50
y
0
500
1000
1500
x

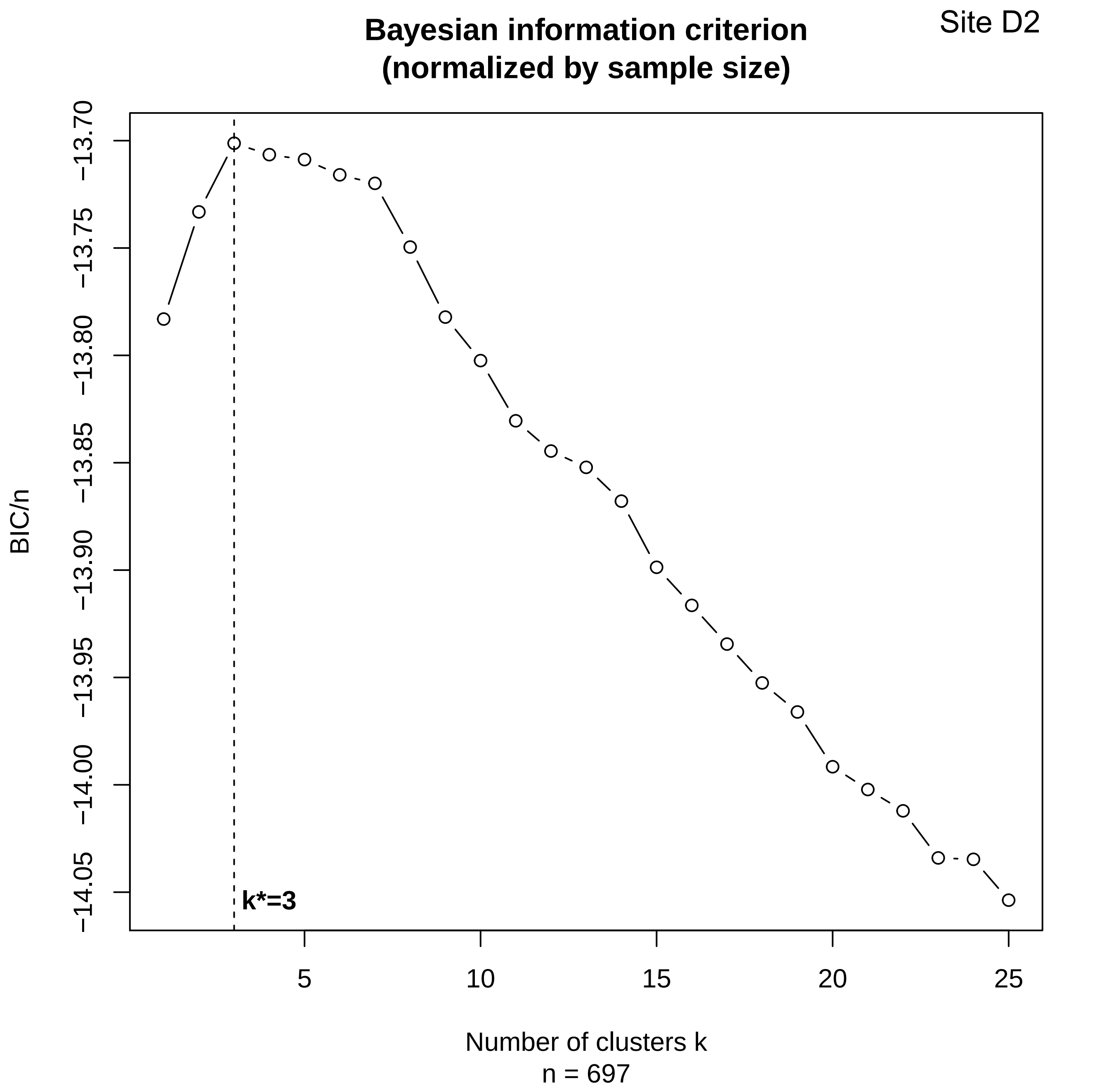
Site D2
Bayesian information criterion
(normalized by sample size)
−13.70
−13.75
−13.80
−13.85
−13.90
−13.95
−14.00
−14.05
BIC/n
k*=3
5
10
15
20
25
Number of clusters k
n = 697

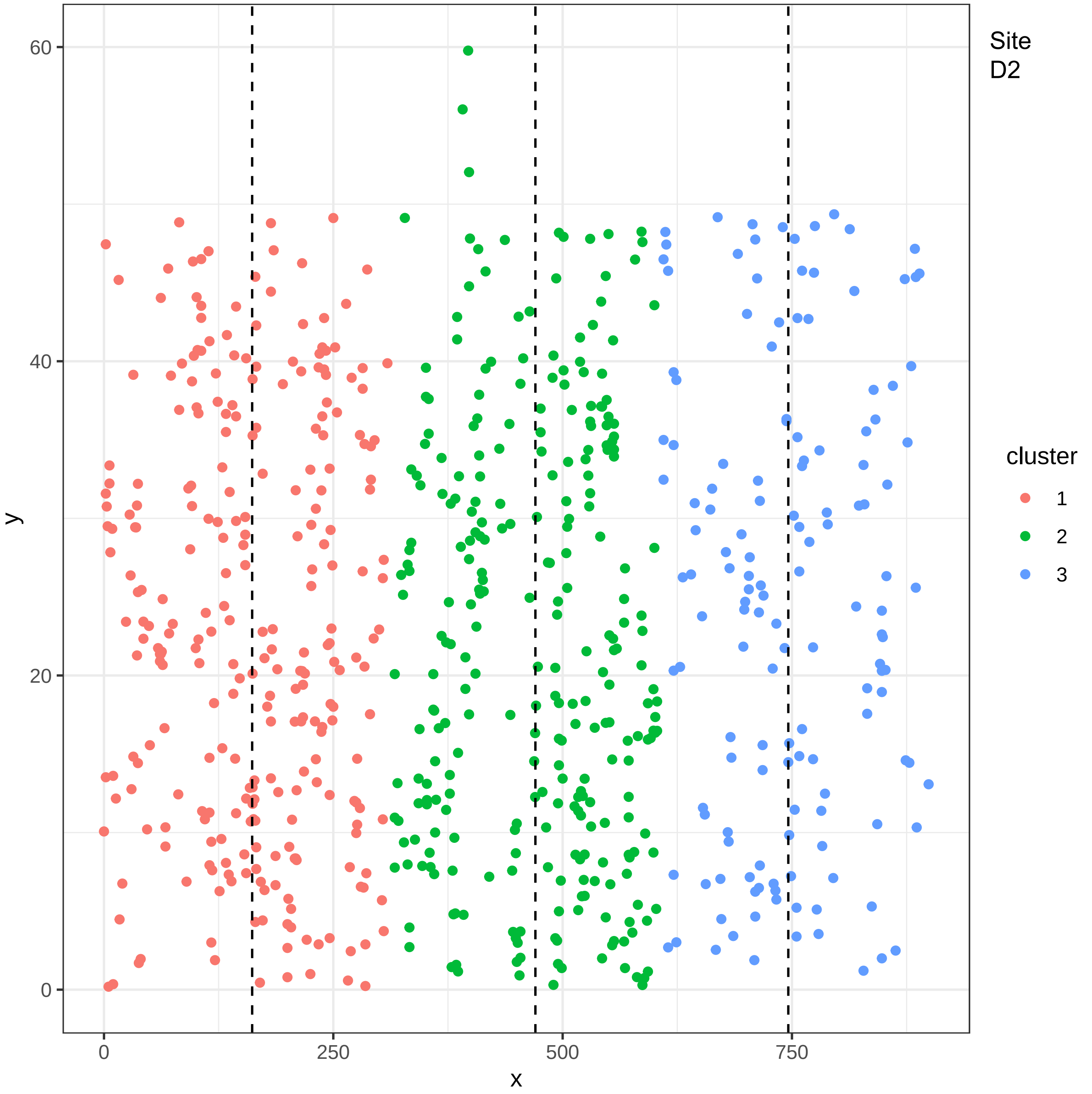

Site
D2
cluster
1
2
3
y
x
0
20
40
60
0
250
500
750

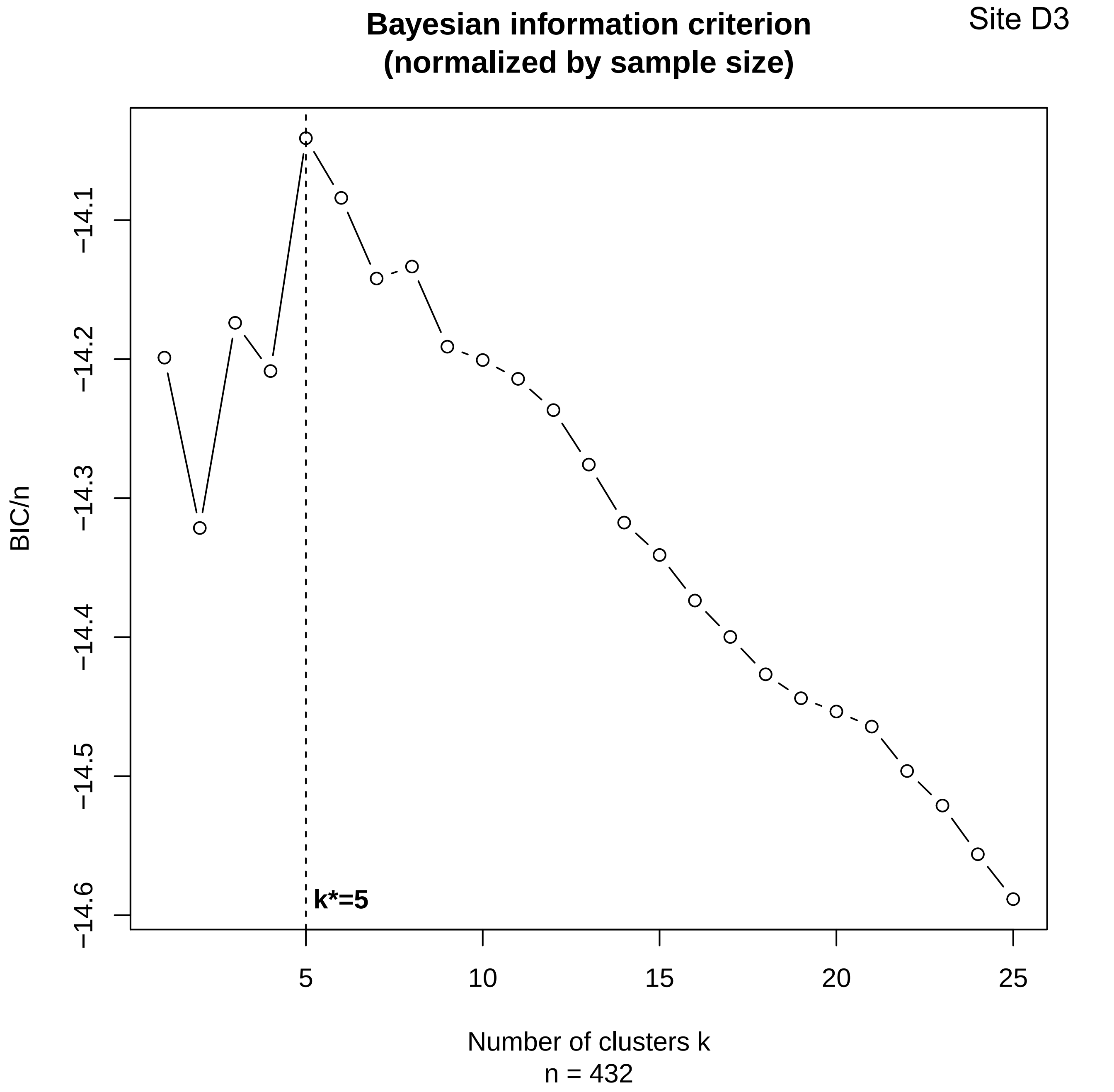

Bayesian information criterion
(normalized by sample size)
Site D3
BIC/n
−14.1
−14.2
−14.3
−14.4
−14.5
−14.6
k*=5
5
10
15
20
25
Number of clusters k
n = 432

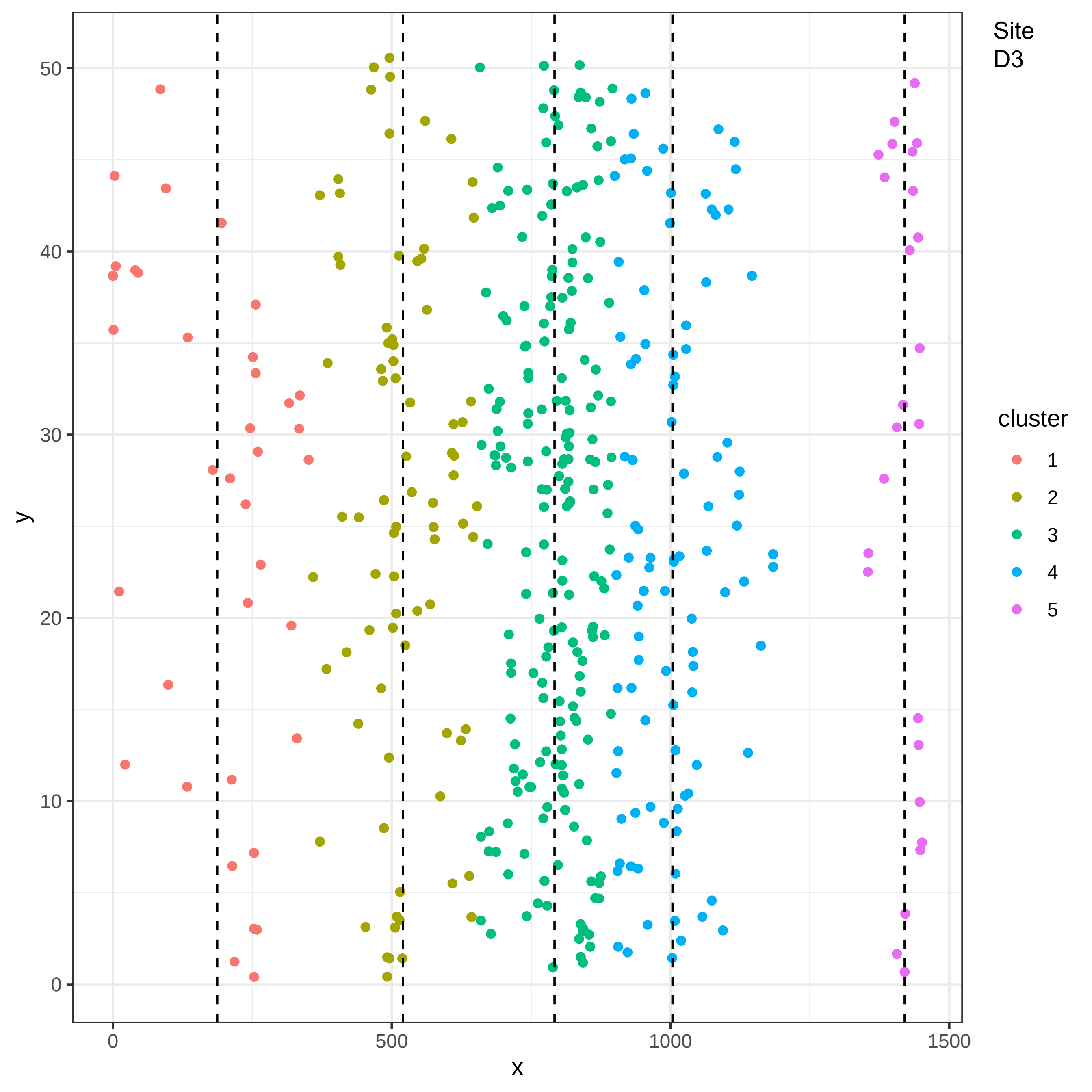

Site
D3
cluster
1
2
3
4
5
y
x
0
10
20
30
40
50
0
500
1000
1500

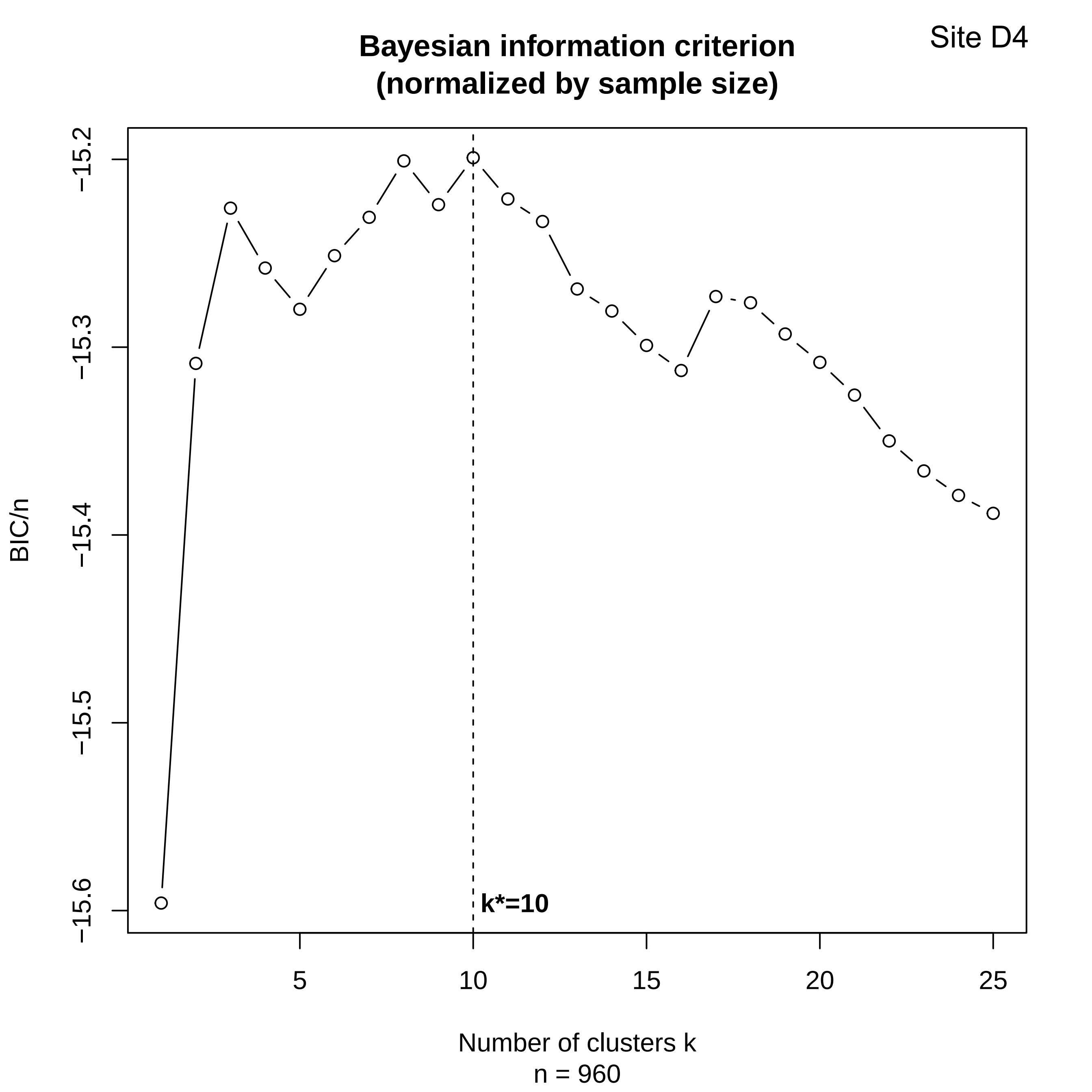
Site D4
Bayesian information criterion
(normalized by sample size)
BIC/n
−15.2
−15.3
−15.4
−15.5
−15.6
k*=10
5
10
15
20
25
Number of clusters k
n = 960

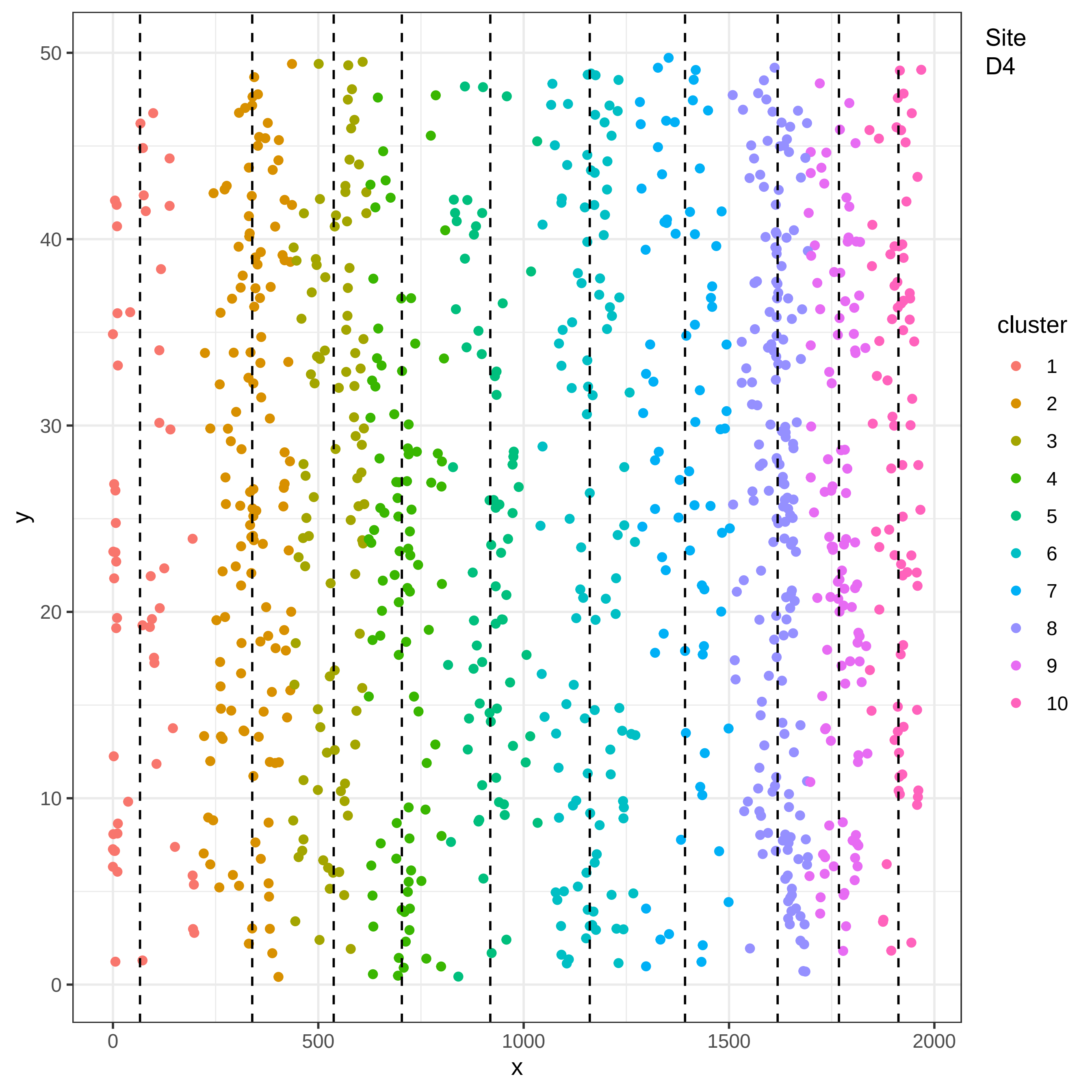

Site
D4
cluster
1
2
3
4
5
6
7
8
9
10
y
x
0
10
20
30
40
50
0
500
1000
1500
2000

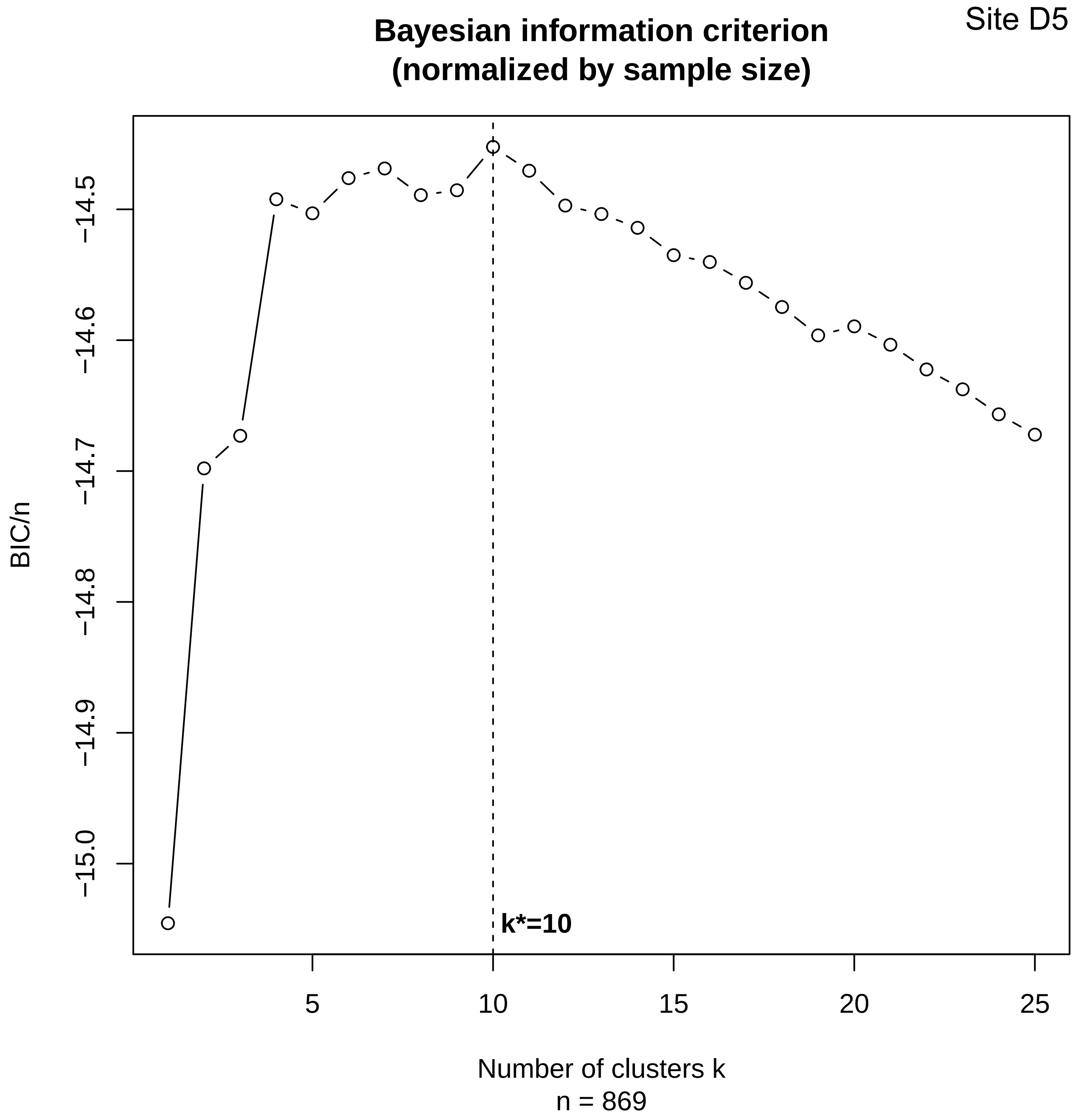
Site D5
Bayesian information criterion
(normalized by sample size)
BIC/n
−14.5
−14.6
−14.7
−14.8
−14.9
−15.0
k*=10
5
10
15
20
25
Number of clusters k
n = 869

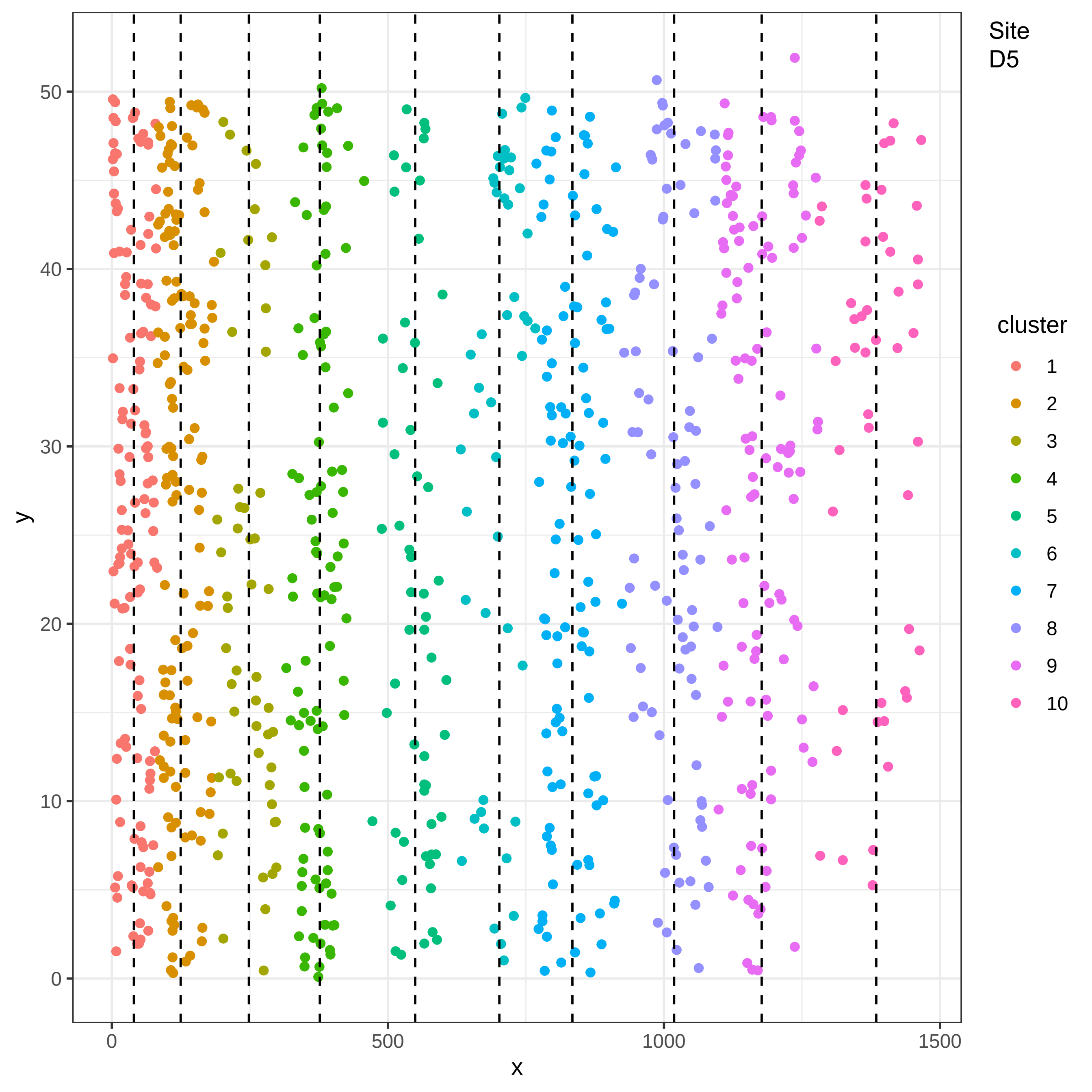
Site
D5
cluster
1
2
3
4
5
6
7
8
9
10
y
x
0
10
20
30
40
50
0
500
1000
1500

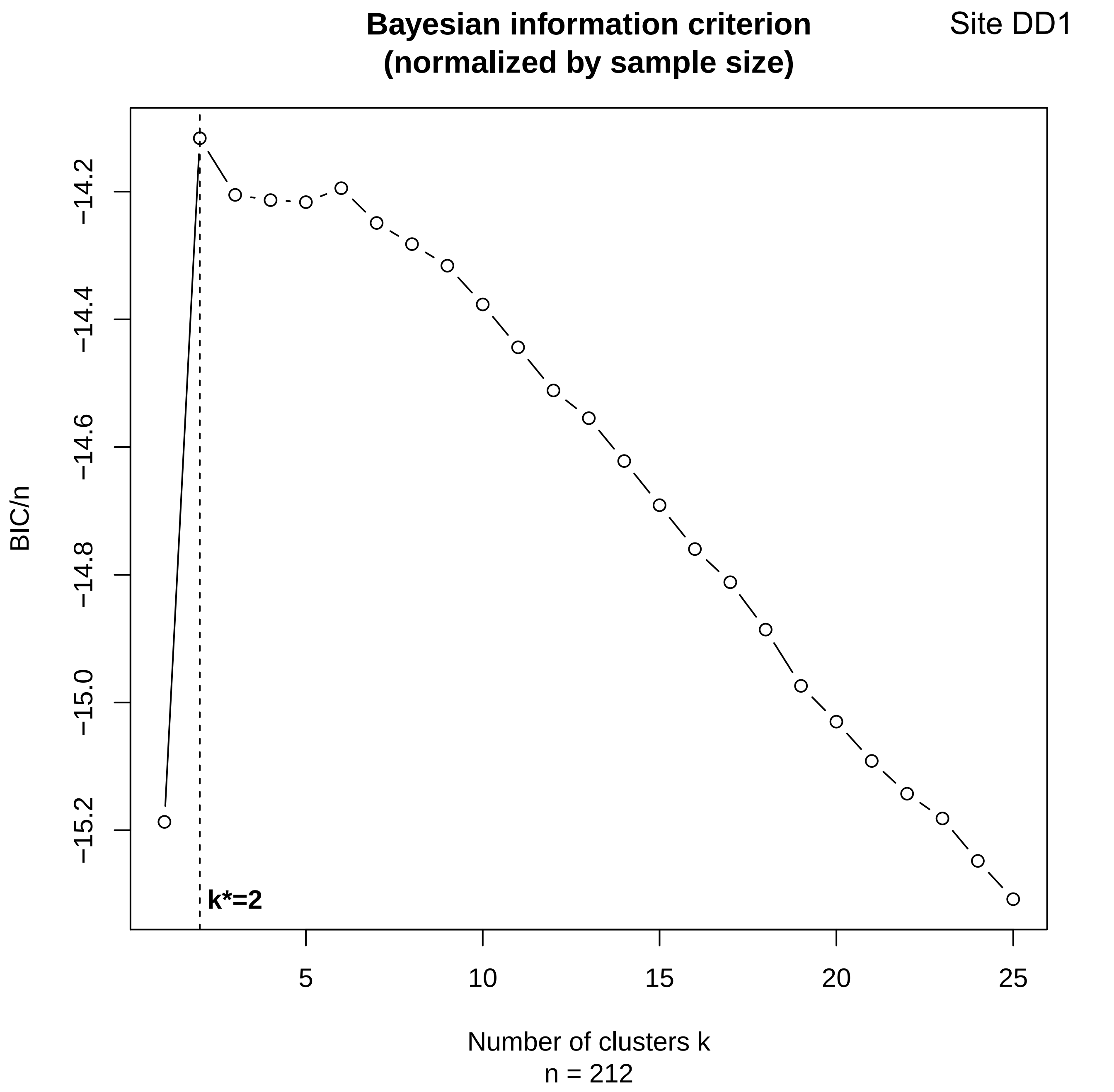

Bayesian information criterion
(normalized by sample size)
Site DD1
BIC/n
−14.2
−14.4
−14.6
−14.8
−15.0
−15.2
k*=2
5
10
15
20
25
Number of clusters k
n = 212

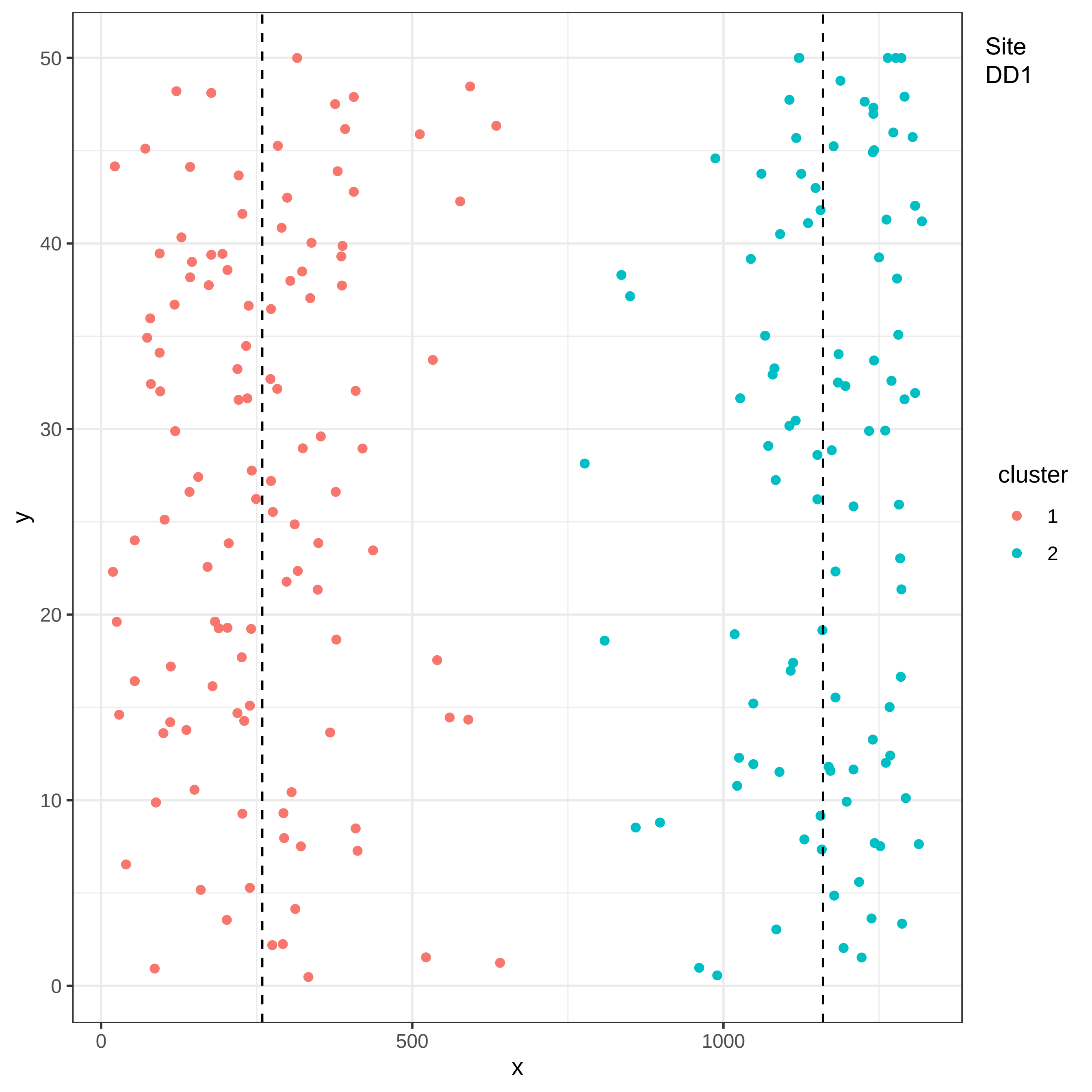

Site
DD1
cluster
1
2
y
x
0
10
20
30
40
50
0
500
1000

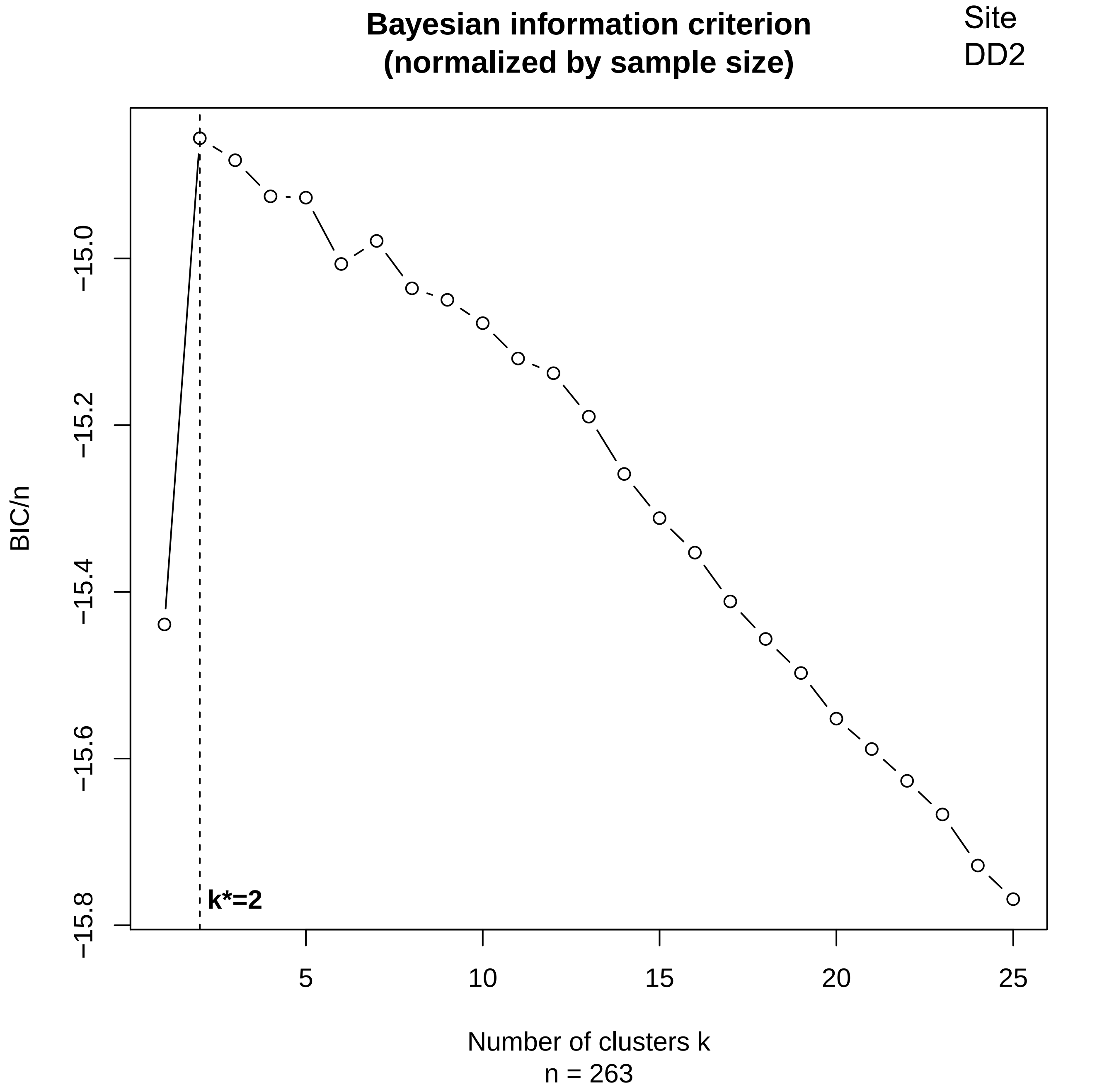

Bayesian information criterion
(normalized by sample size)
Site
DD2
BIC/n
−15.0
−15.2
−15.4
−15.6
−15.8
k*=2
5
10
15
20
25
Number of clusters k
n = 263

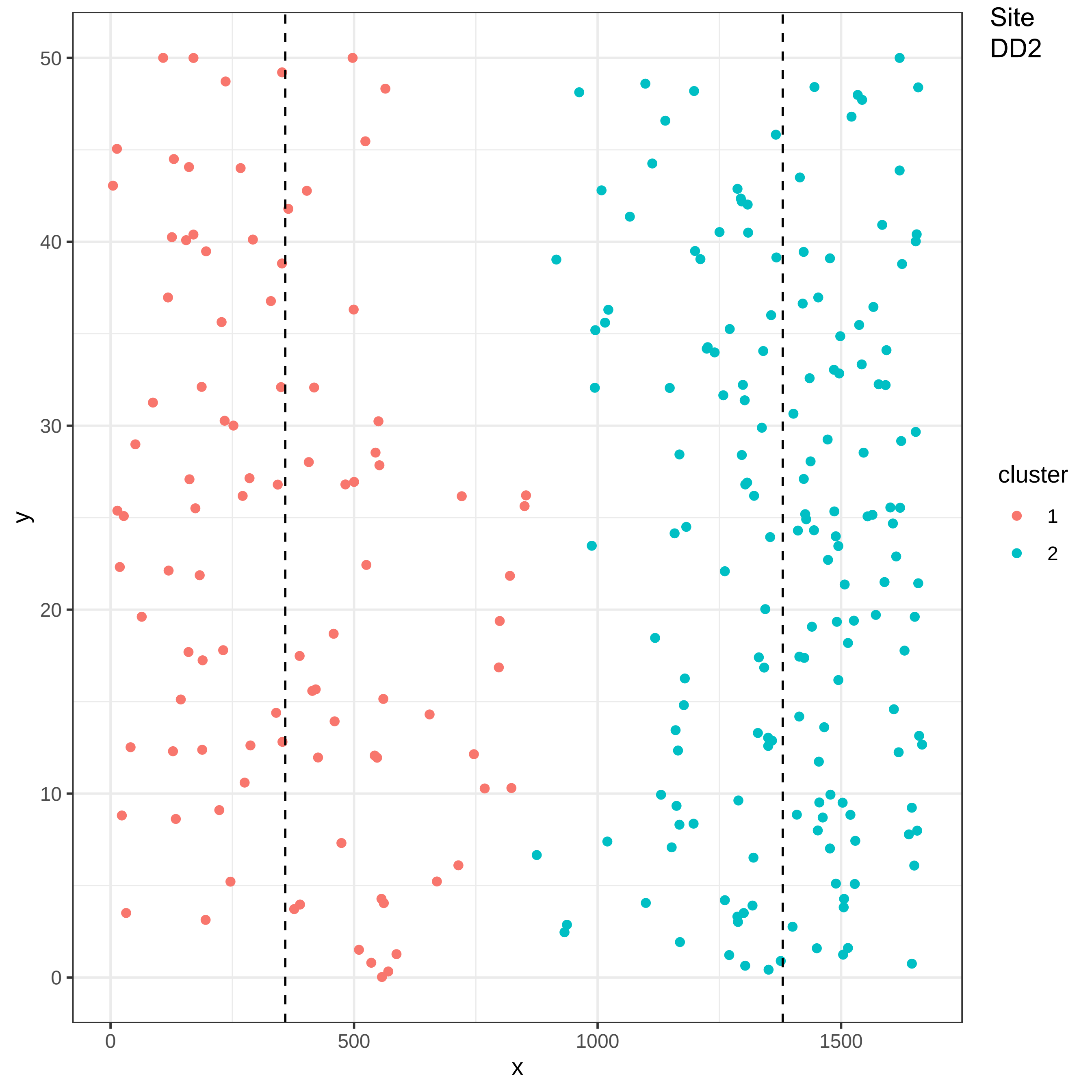

Site
DD2
cluster
1
2
y
x
0
10
20
30
40
50
0
500
1000
1500

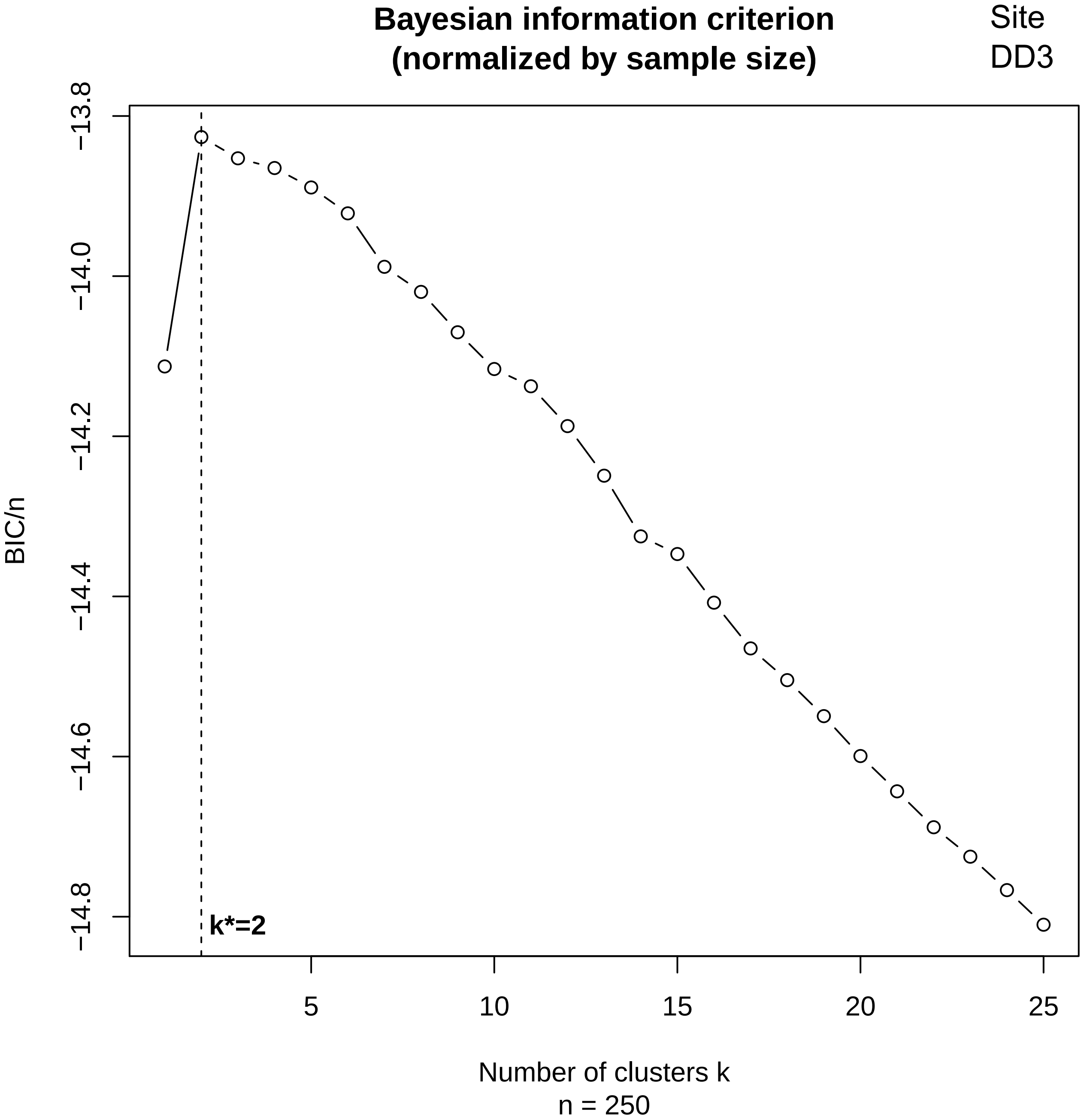
Bayesian information criterion
(normalized by sample size)
Site
DD3
BIC/n
−13.8
−14.0
−14.2
−14.4
−14.6
−14.8
k*=2
5
10
15
20
25
Number of clusters k
n = 250

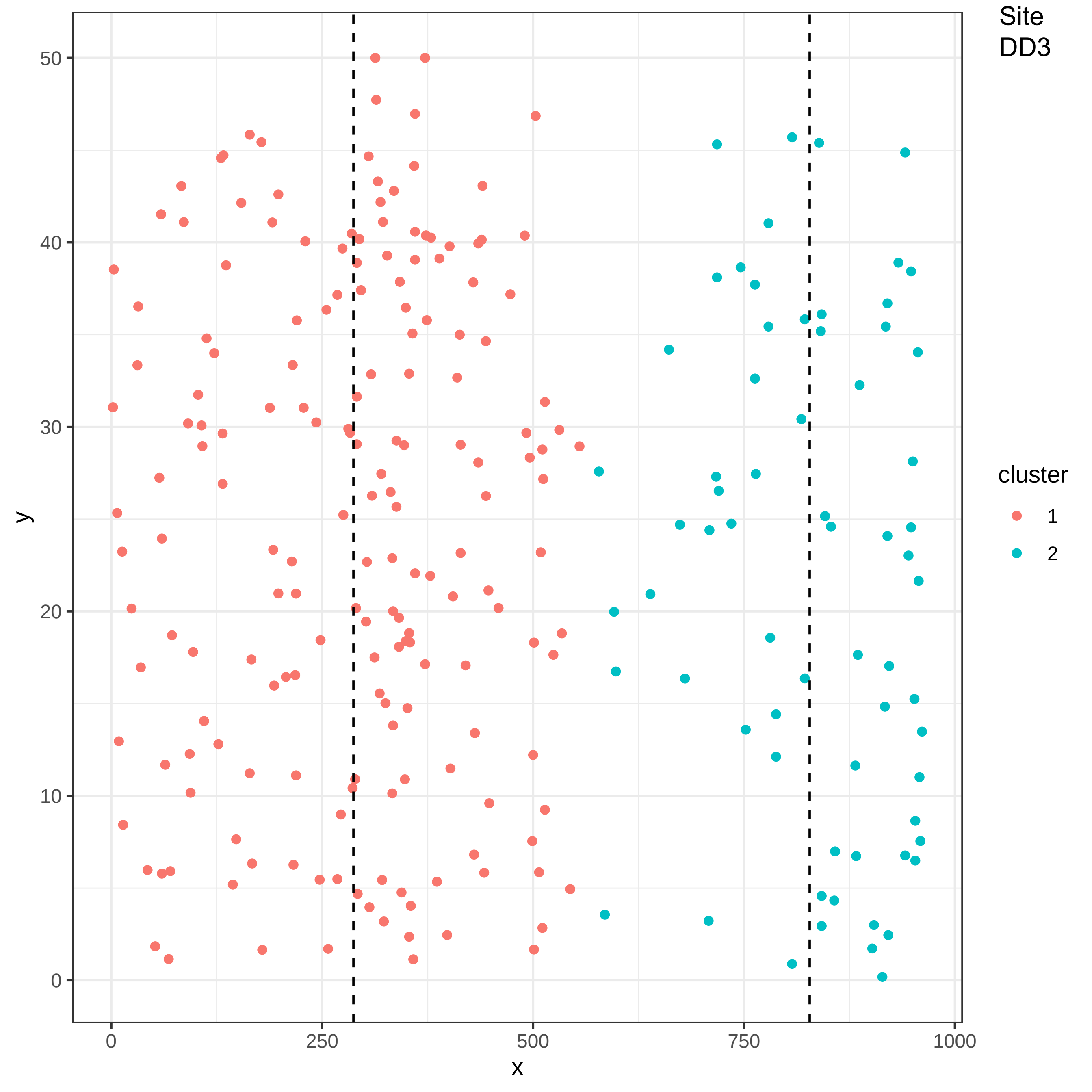

Site
DD3
50
40
30
20
10
0
0
250
500
750
1000
x
y
cluster
1
2

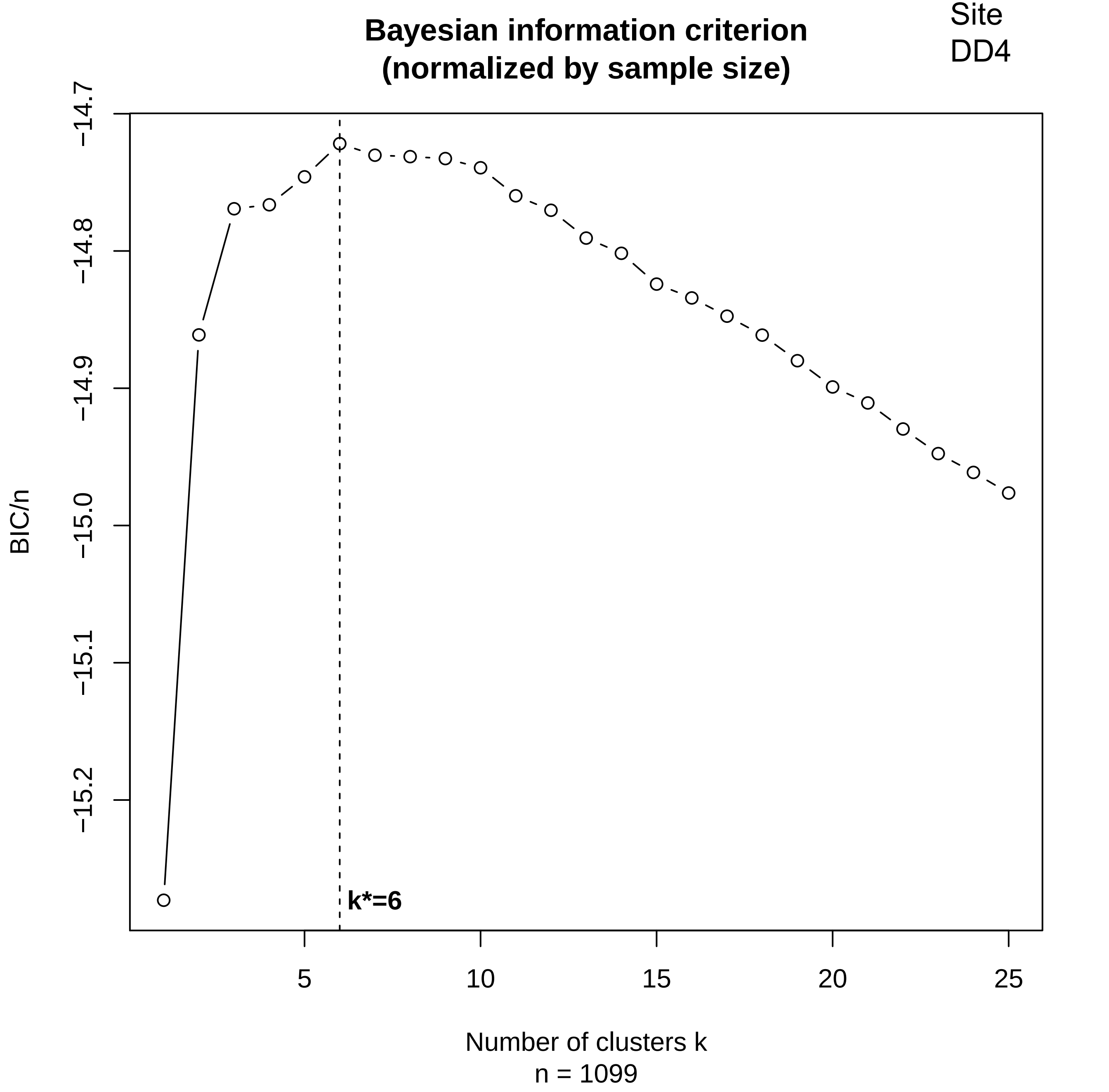

Bayesian information criterion
(normalized by sample size)
Site
DD4
−14.7
−14.8
−14.9
−15.0
−15.1
−15.2
BIC/n
k*=6
5
10
15
20
25
Number of clusters k
n = 1099

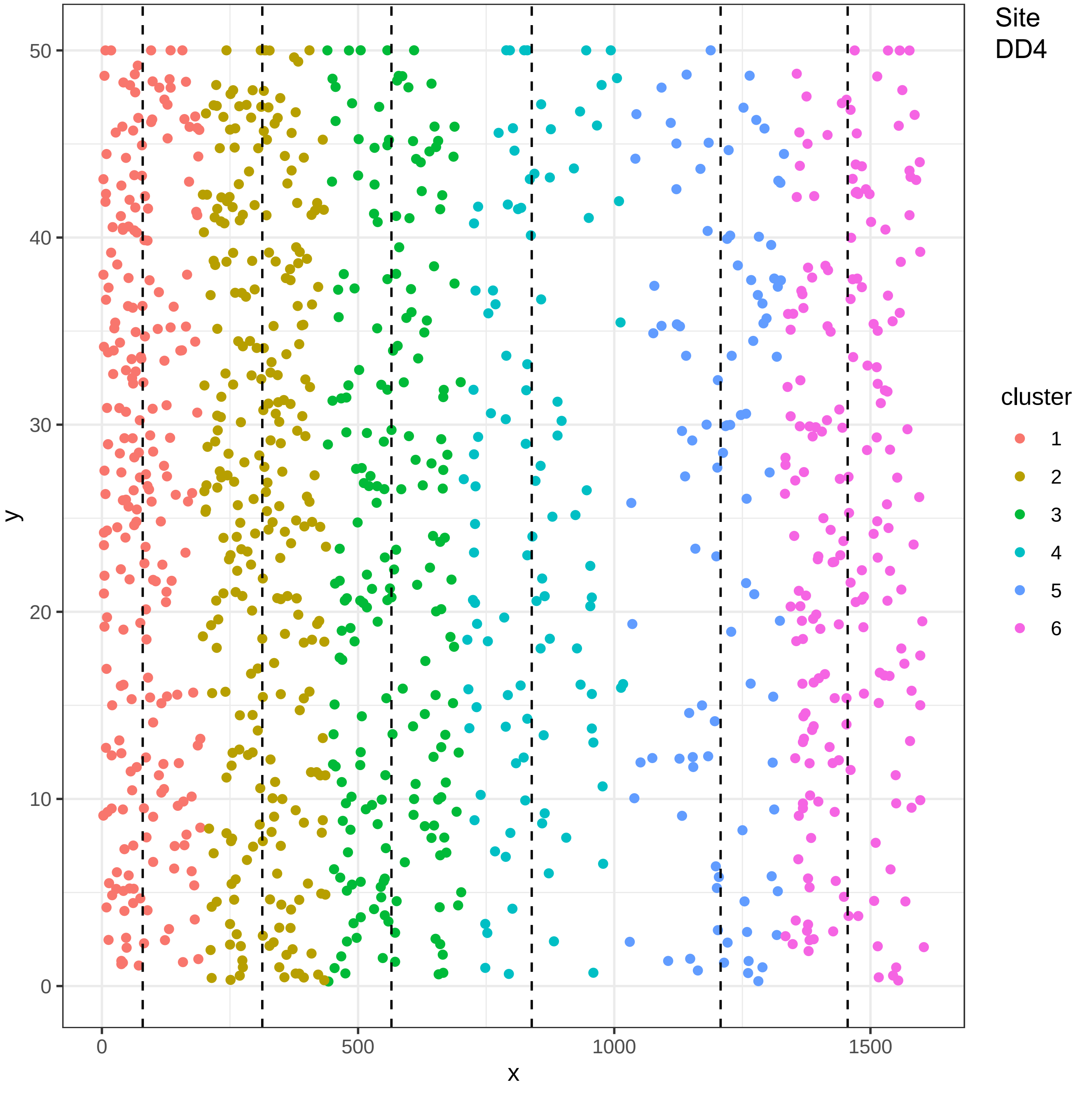

Site
DD4
cluster
1
2
3
4
5
6
y
x
0
10
20
30
40
50
0
500
1000
1500

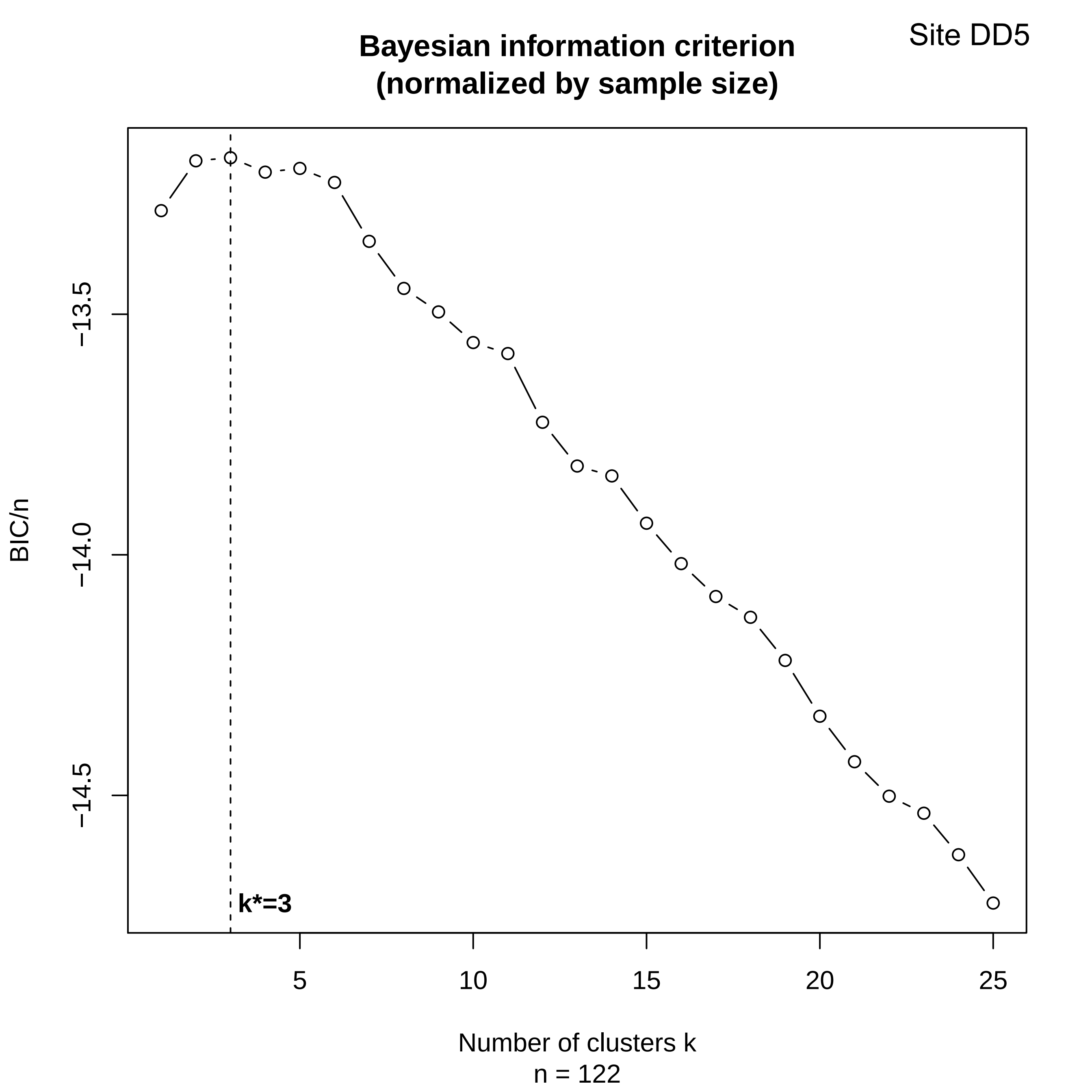
Site DD5
Bayesian information criterion
(normalized by sample size)
BIC/n
−13.5
−14.0
−14.5
k*=3
5
10
15
20
25
Number of clusters k
n = 122

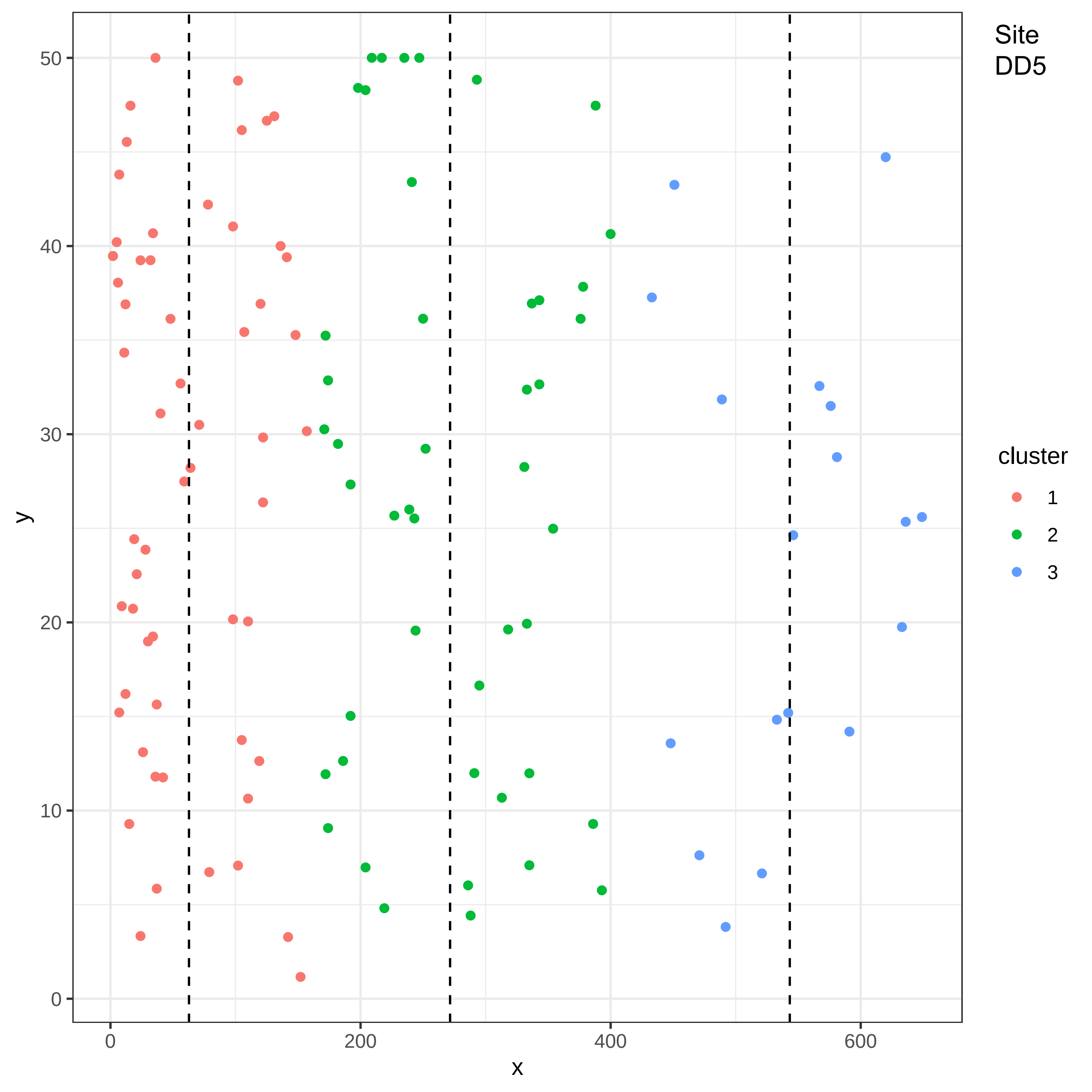
Site
DD5
cluster
1
2
3
y
x
0
10
20
30
40
50
0
200
400
600

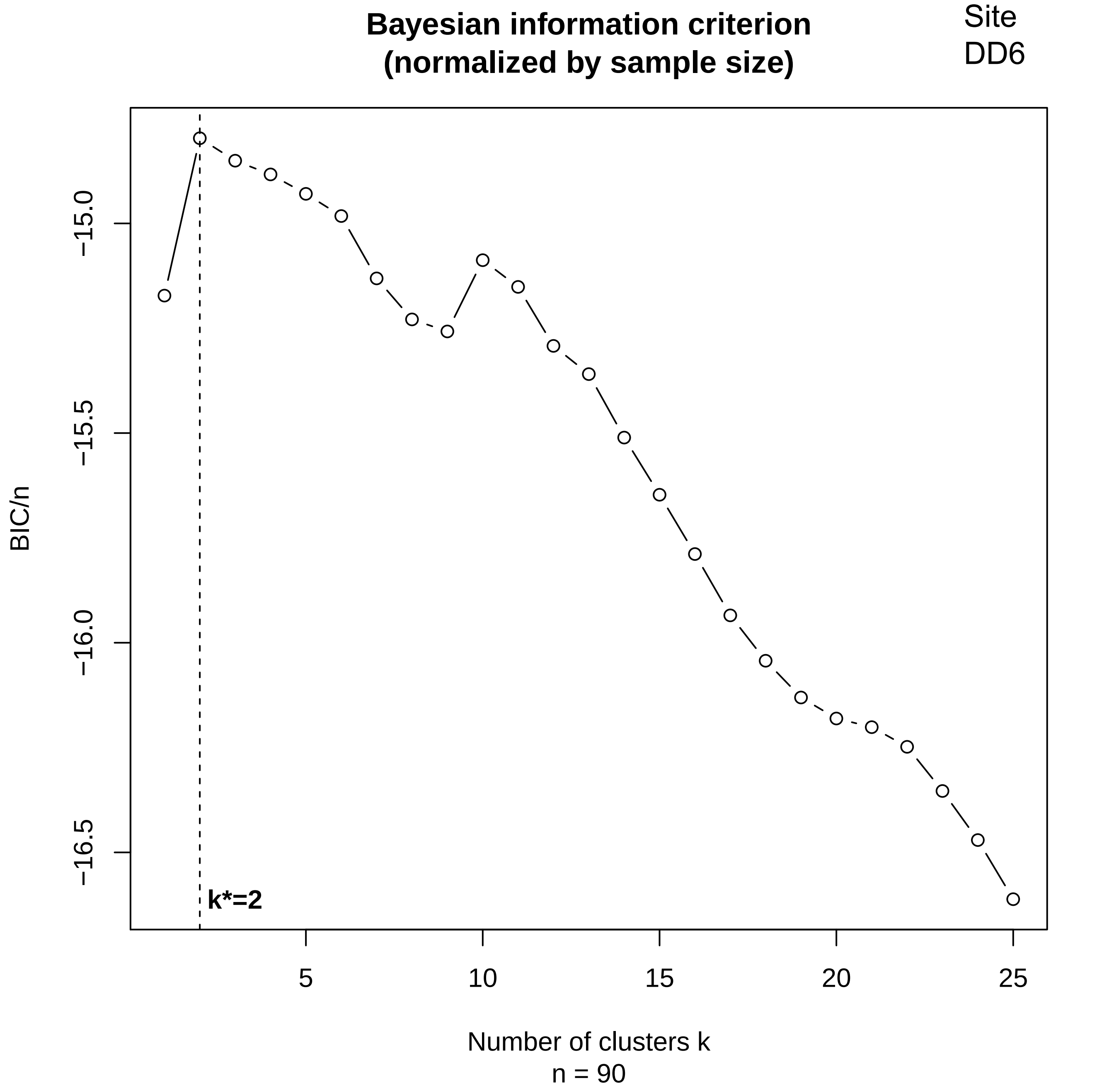

Bayesian information criterion
(normalized by sample size)
Site
DD6
BIC/n
−15.0
−15.5
−16.0
−16.5
k*=2
5
10
15
20
25
Number of clusters k
n = 90

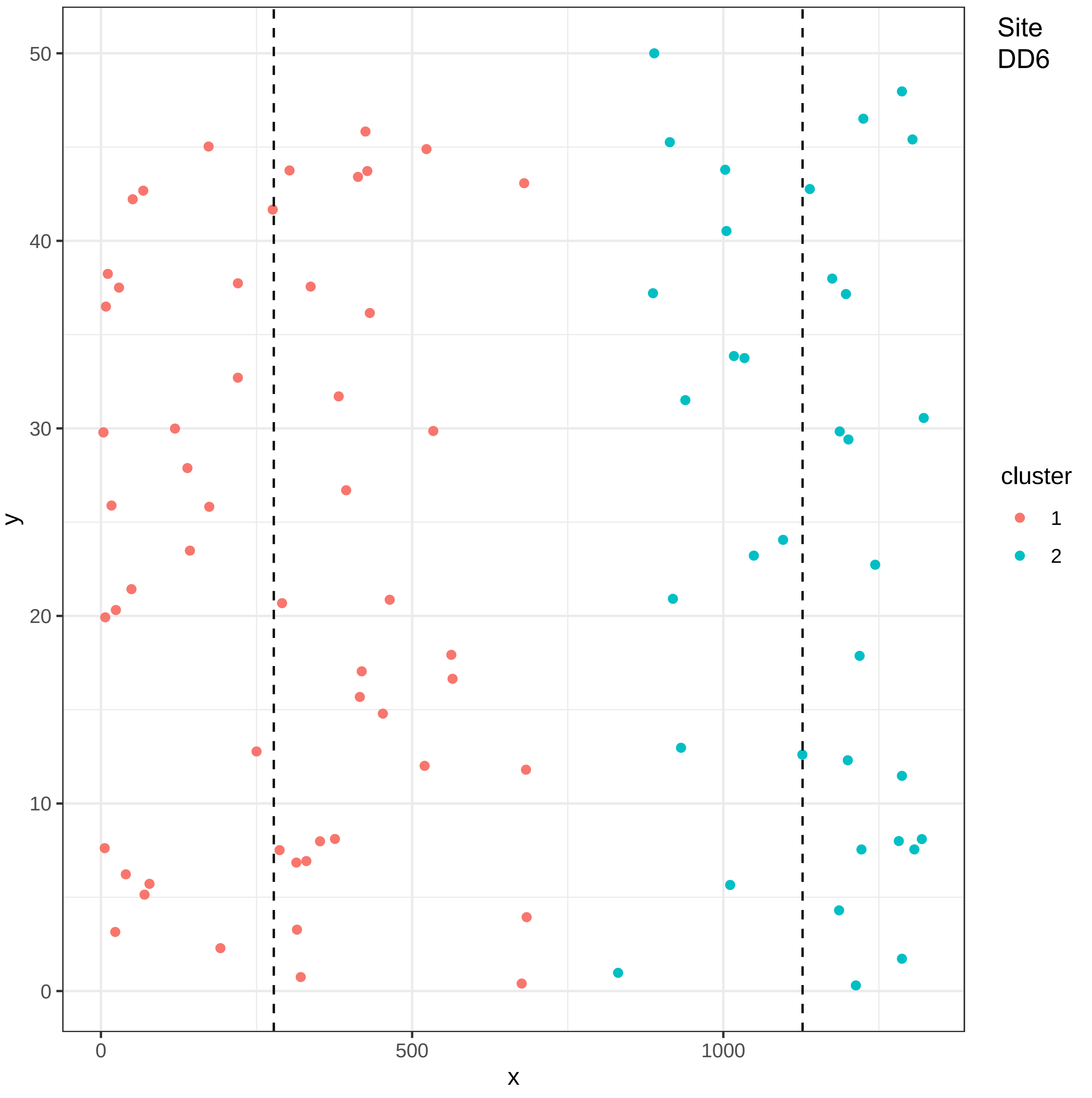
Site
DD6
cluster
1
2
y
x
0
10
20
30
40
50
0
500
1000

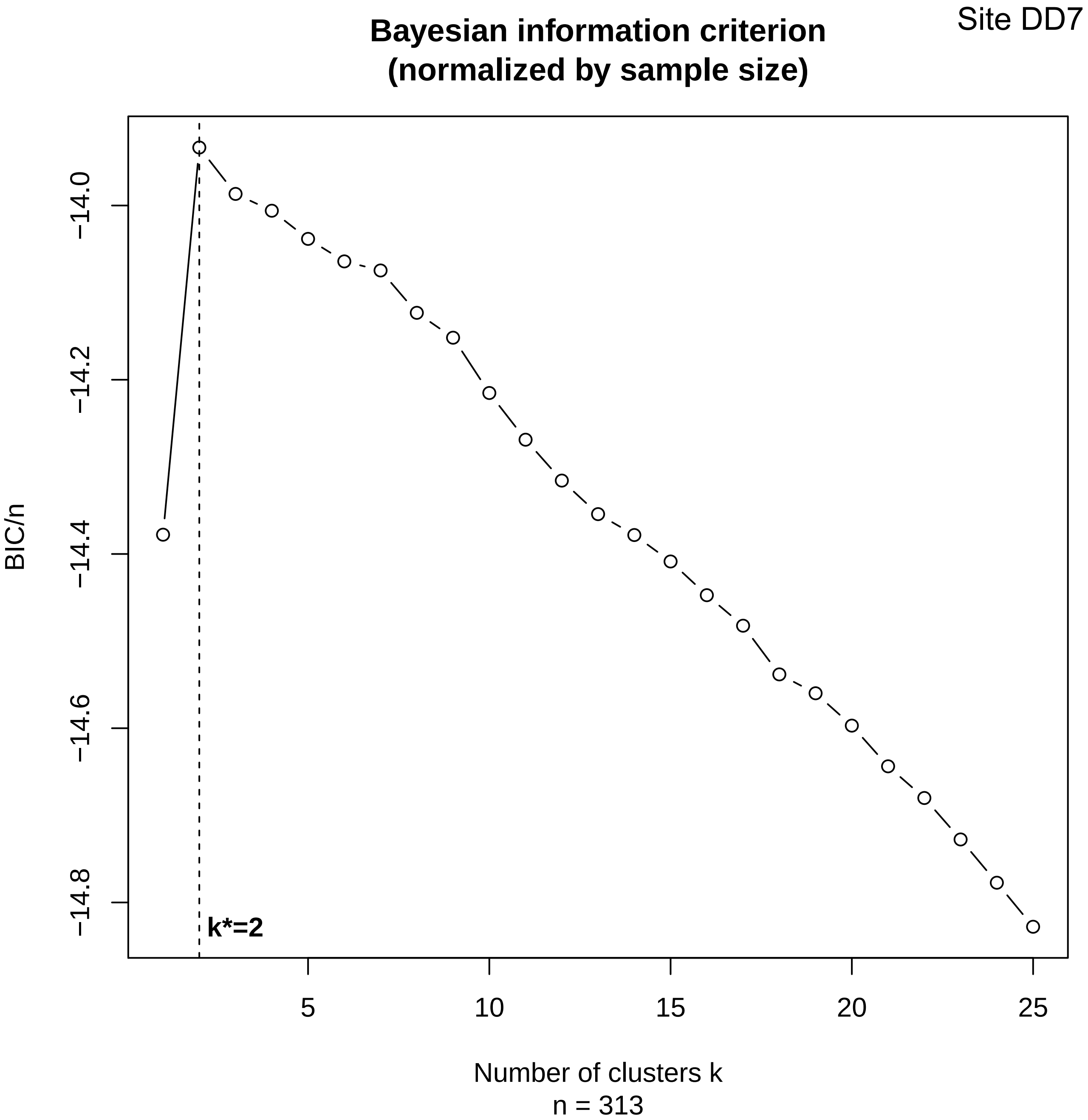

Site DD7
Bayesian information criterion
(normalized by sample size)
−14.0
−14.2
−14.4
−14.6
−14.8
BIC/n
k*=2
5
10
15
20
25
Number of clusters k
n = 313

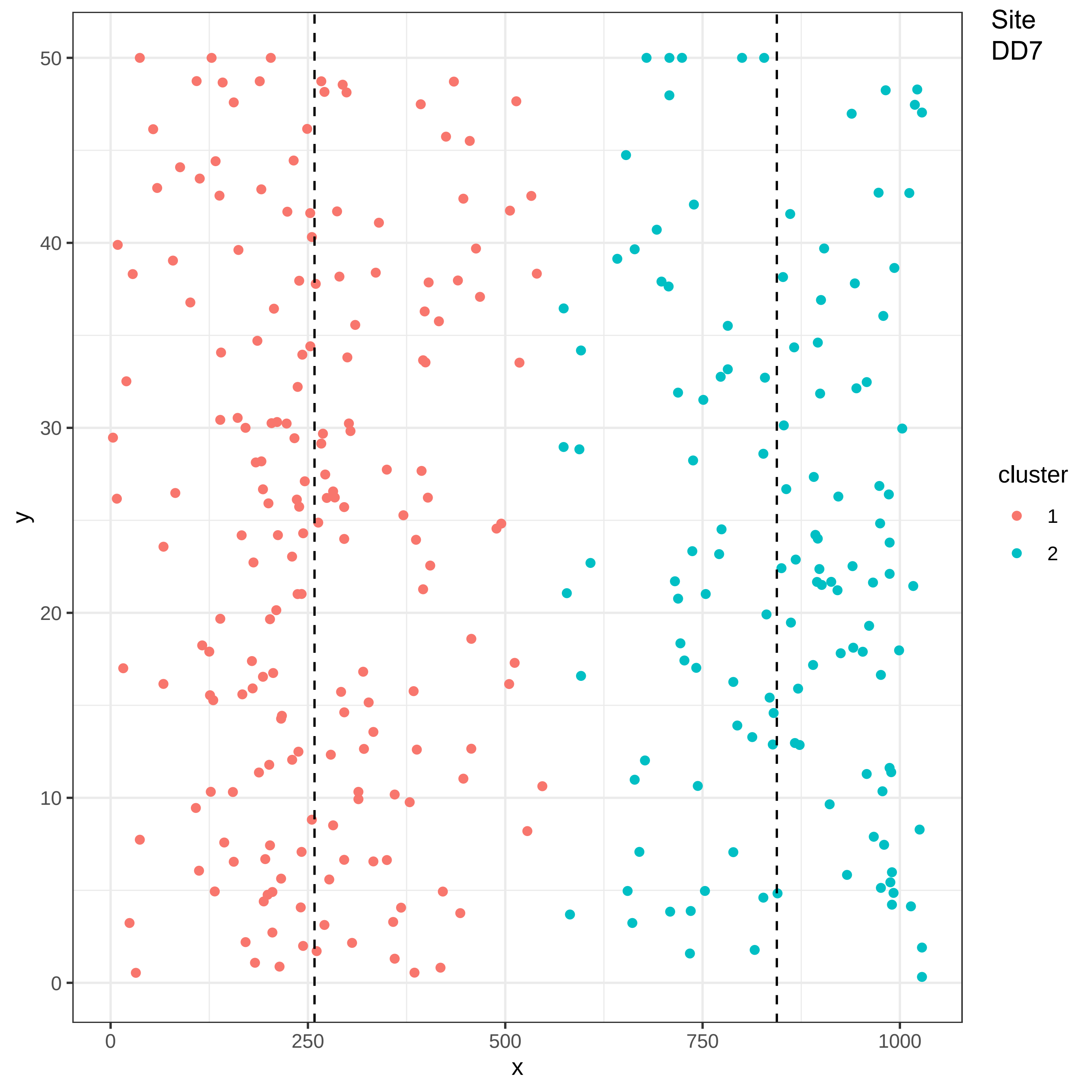

Site
DD7
y
x
0
10
20
30
40
50
0
250
500
750
1000
cluster
1
2

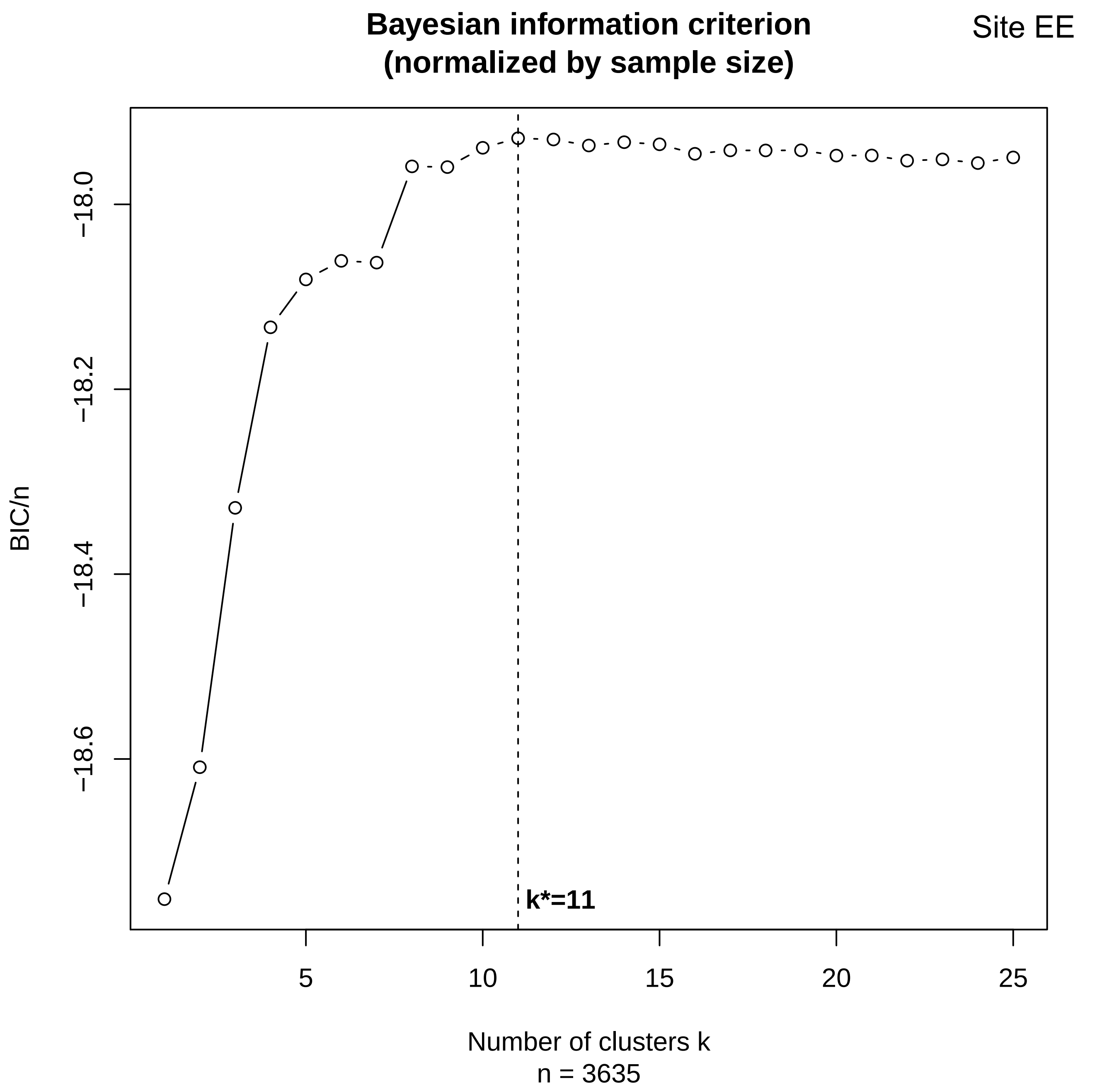
Bayesian information criterion
(normalized by sample size)
Site EE
−18.0
−18.2
−18.4
−18.6
BIC/n
k*=11
5
10
15
20
25
Number of clusters k
n = 3635

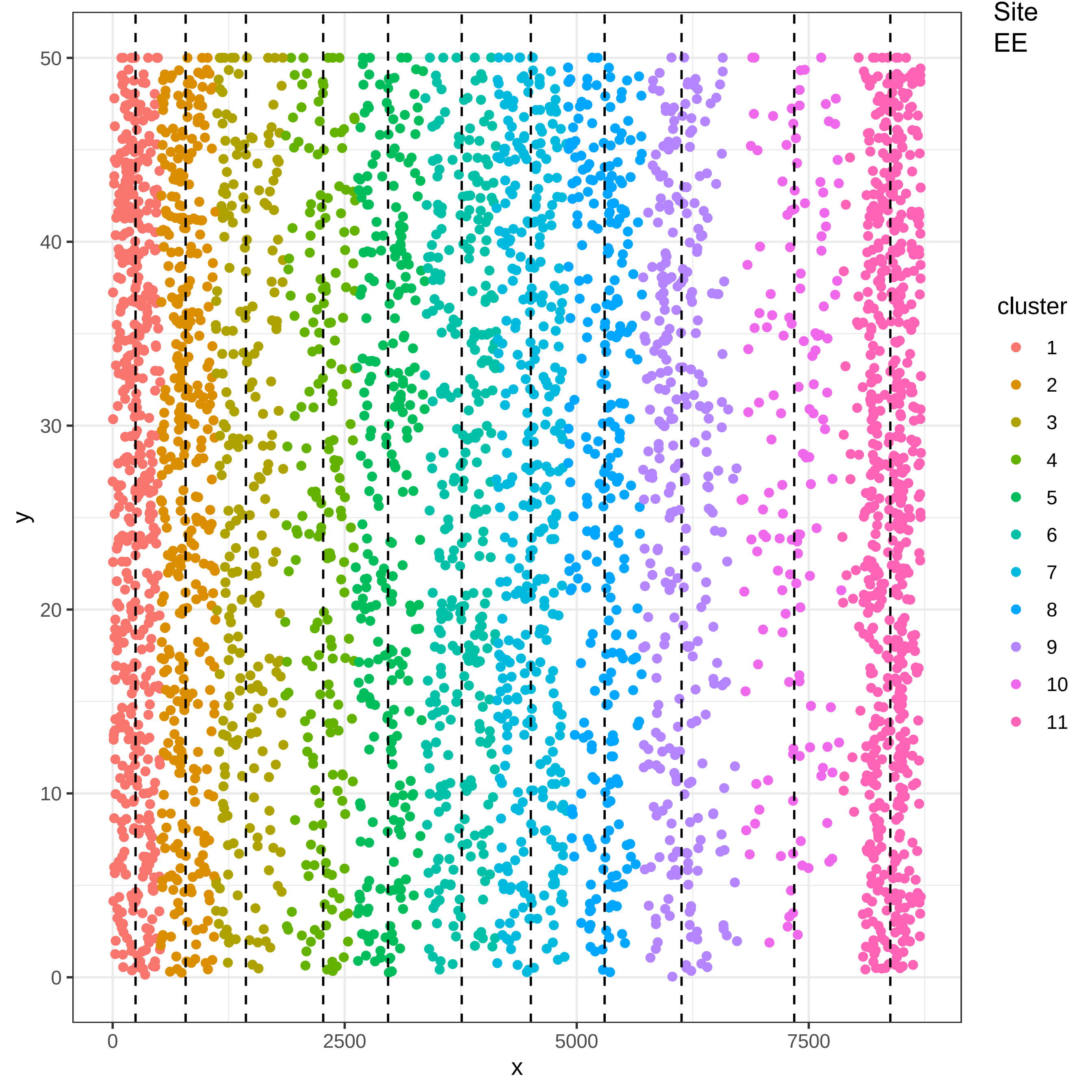
Site
EE
cluster
1
2
3
4
5
6
7
8
9
10
11
y
x
0
10
20
30
40
50
0
2500
5000
7500

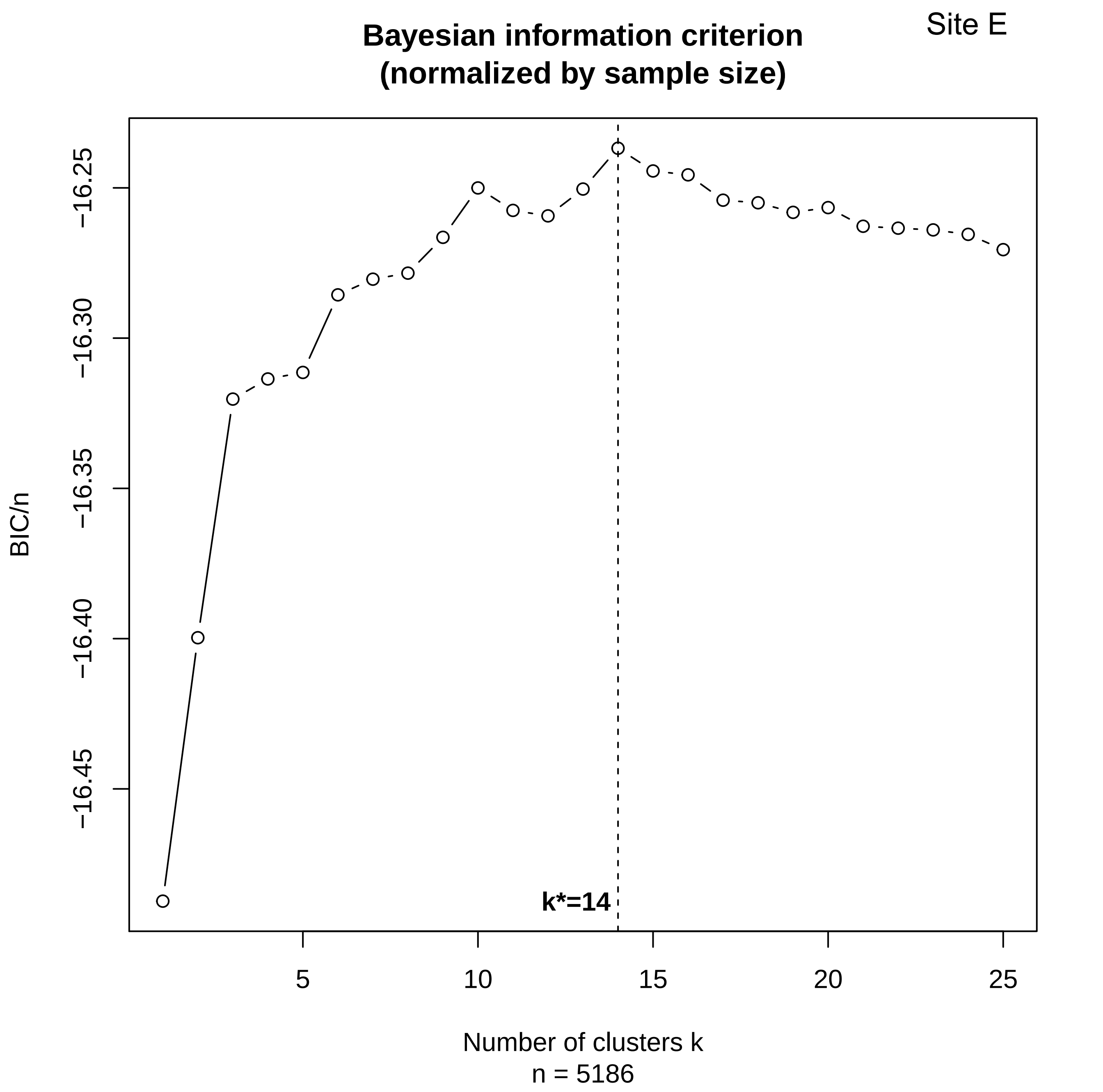
Site E
Bayesian information criterion
(normalized by sample size)
BIC/n
−16.25
−16.30
−16.35
−16.40
−16.45
k*=14
5
10
15
20
25
Number of clusters k
n = 5186

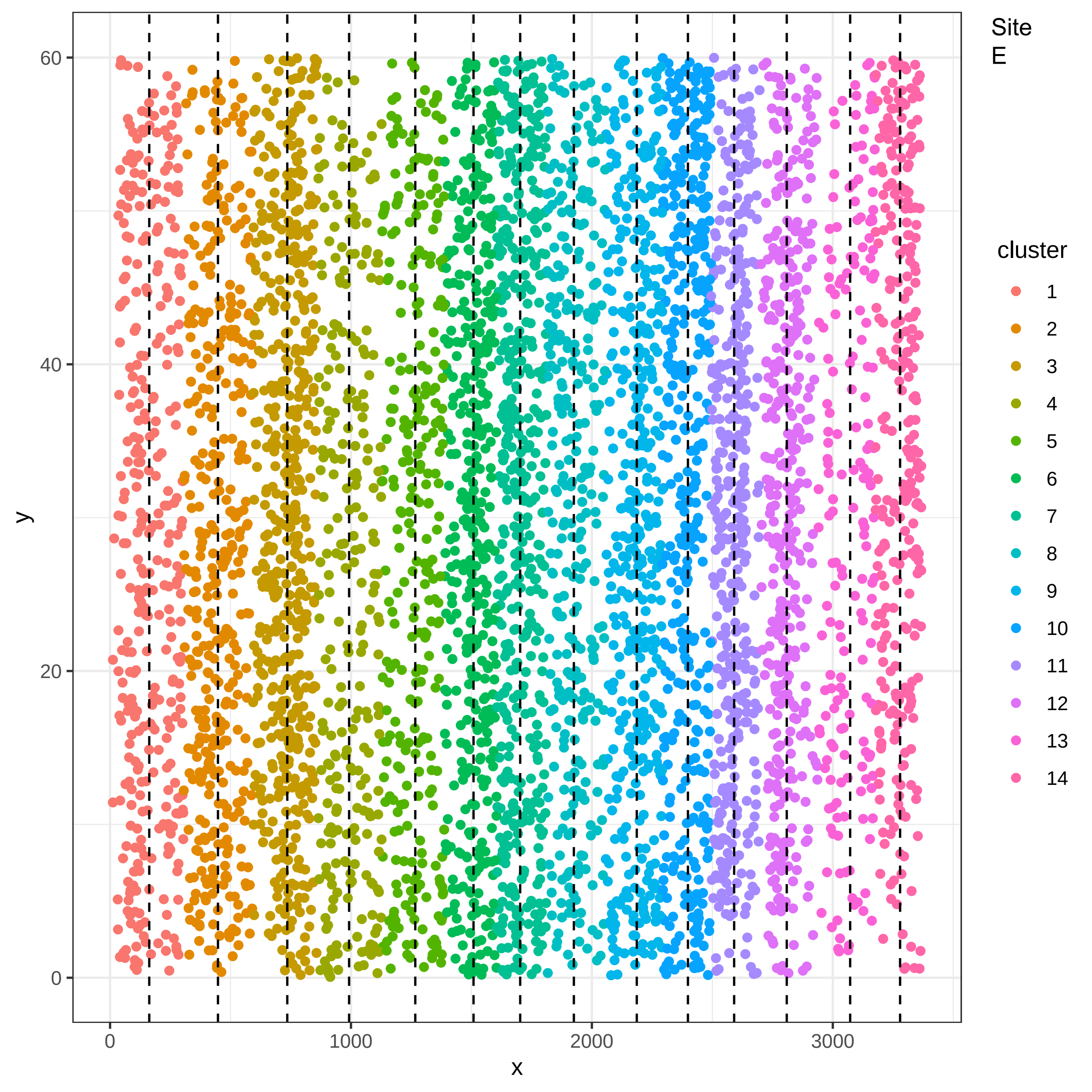
Site
E
cluster
1
2
3
4
5
6
7
8
9
10
11
12
13
14
y
60
40
20
0
x
0
1000
2000
3000

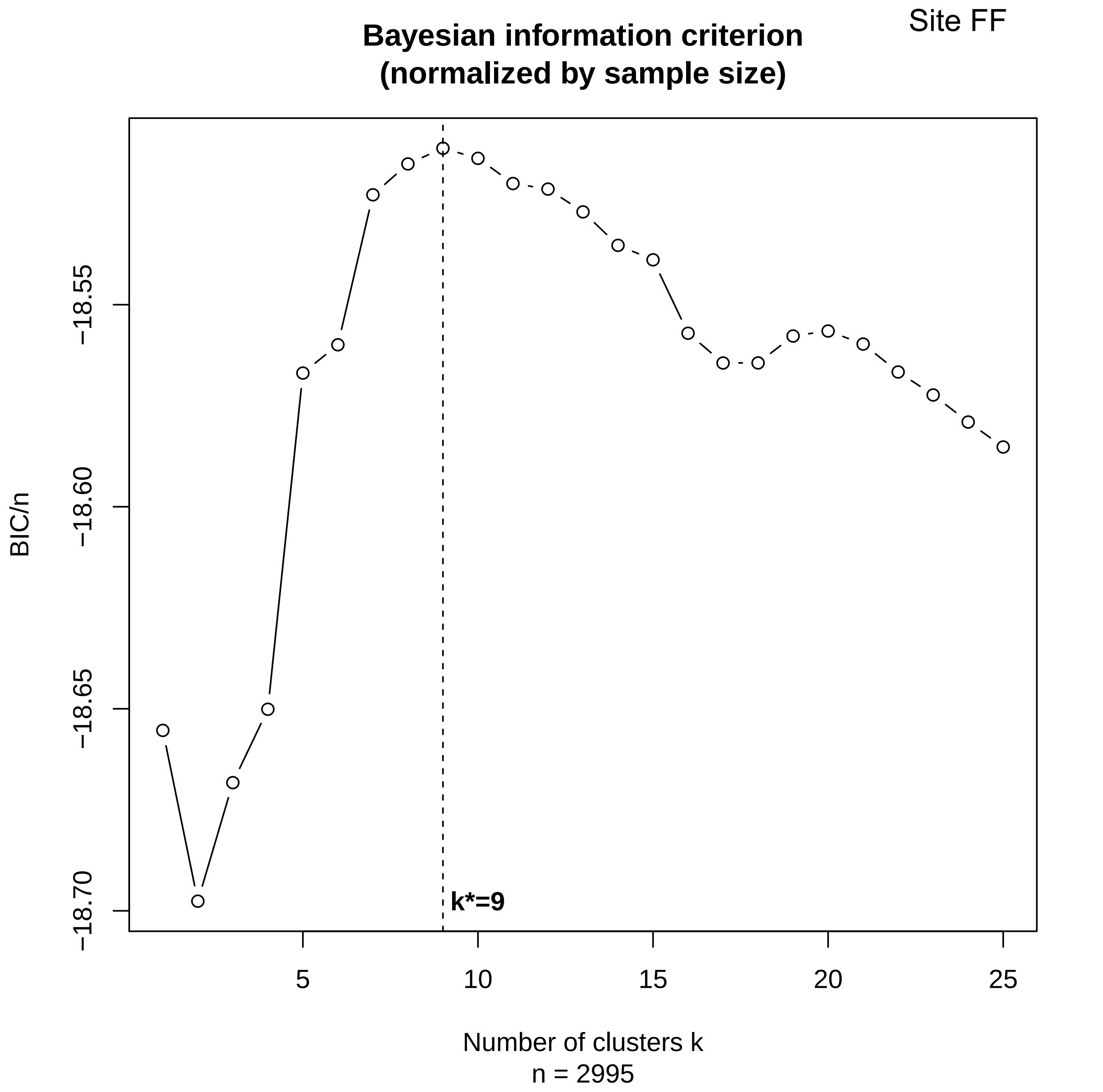
Site FF
Bayesian information criterion
(normalized by sample size)
BIC/n
−18.55
−18.60
−18.65
−18.70
k*=9
5
10
15
20
25
Number of clusters k
n = 2995

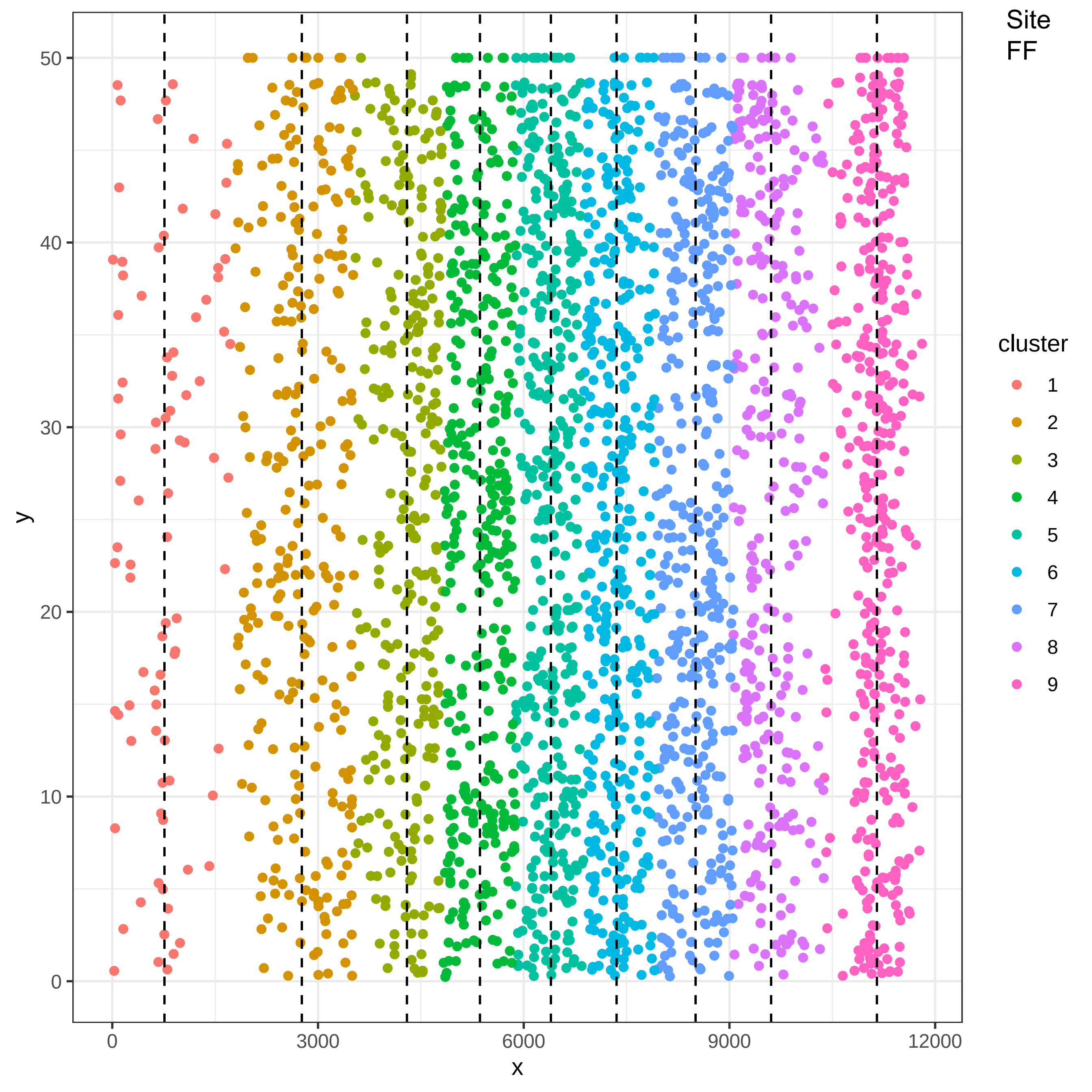

Site
FF
cluster
1
2
3
4
5
6
7
8
9
y
x
0
10
20
30
40
50
0
3000
6000
9000
12000

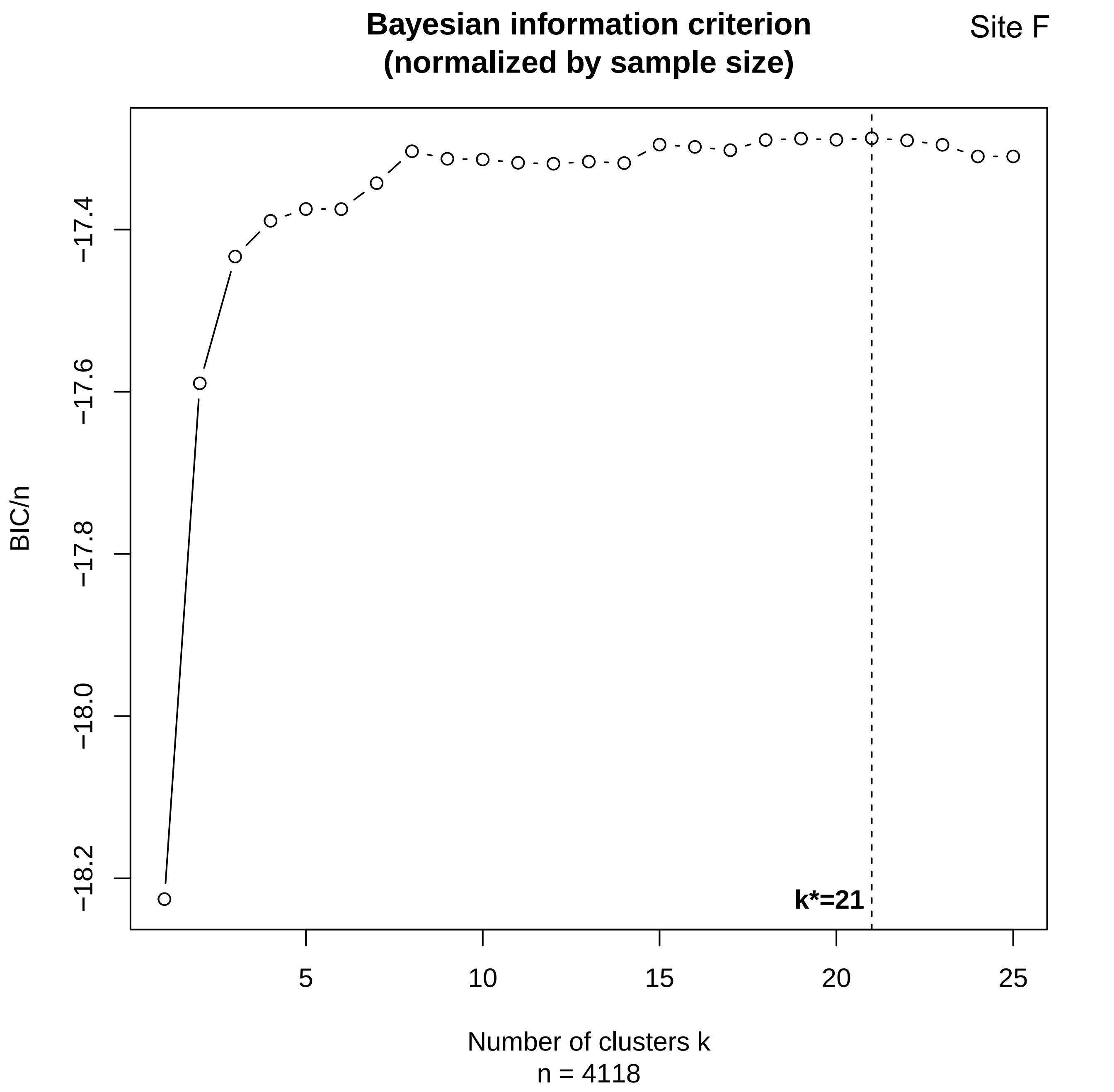

Bayesian information criterion
(normalized by sample size)
Site F
BIC/n
−17.4
−17.6
−17.8
−18.0
−18.2
k*=21
5
10
15
20
25
Number of clusters k
n = 4118

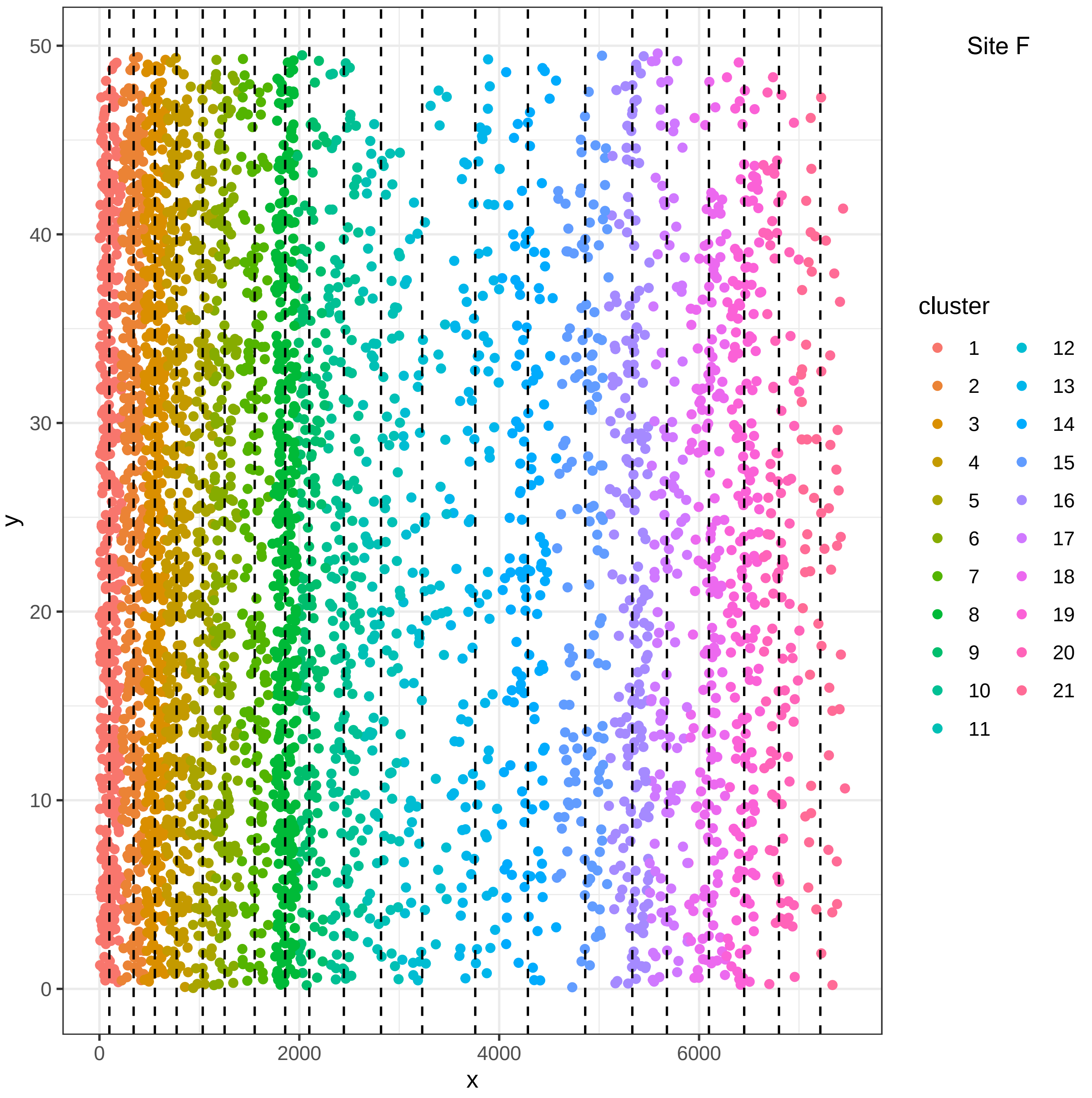

Site F
cluster
1
2
3
4
5
6
7
8
9
10
11
12
13
14
15
16
17
18
19
20
21
y
x
0
10
20
30
40
50
0
2000
4000
6000

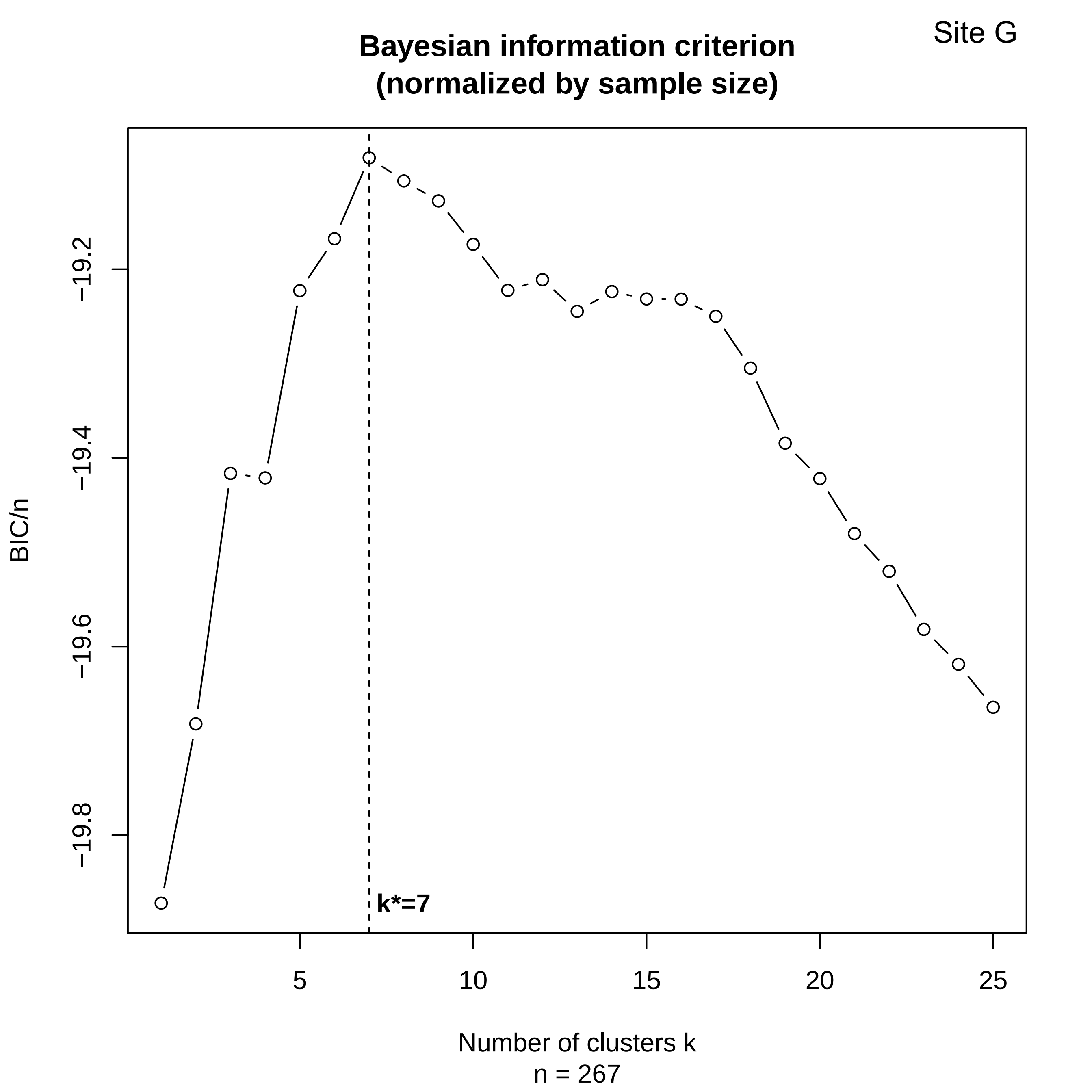
Site G
Bayesian information criterion
(normalized by sample size)
BIC/n
−19.2
−19.4
−19.6
−19.8
k*=7
5
10
15
20
25
Number of clusters k
n = 267

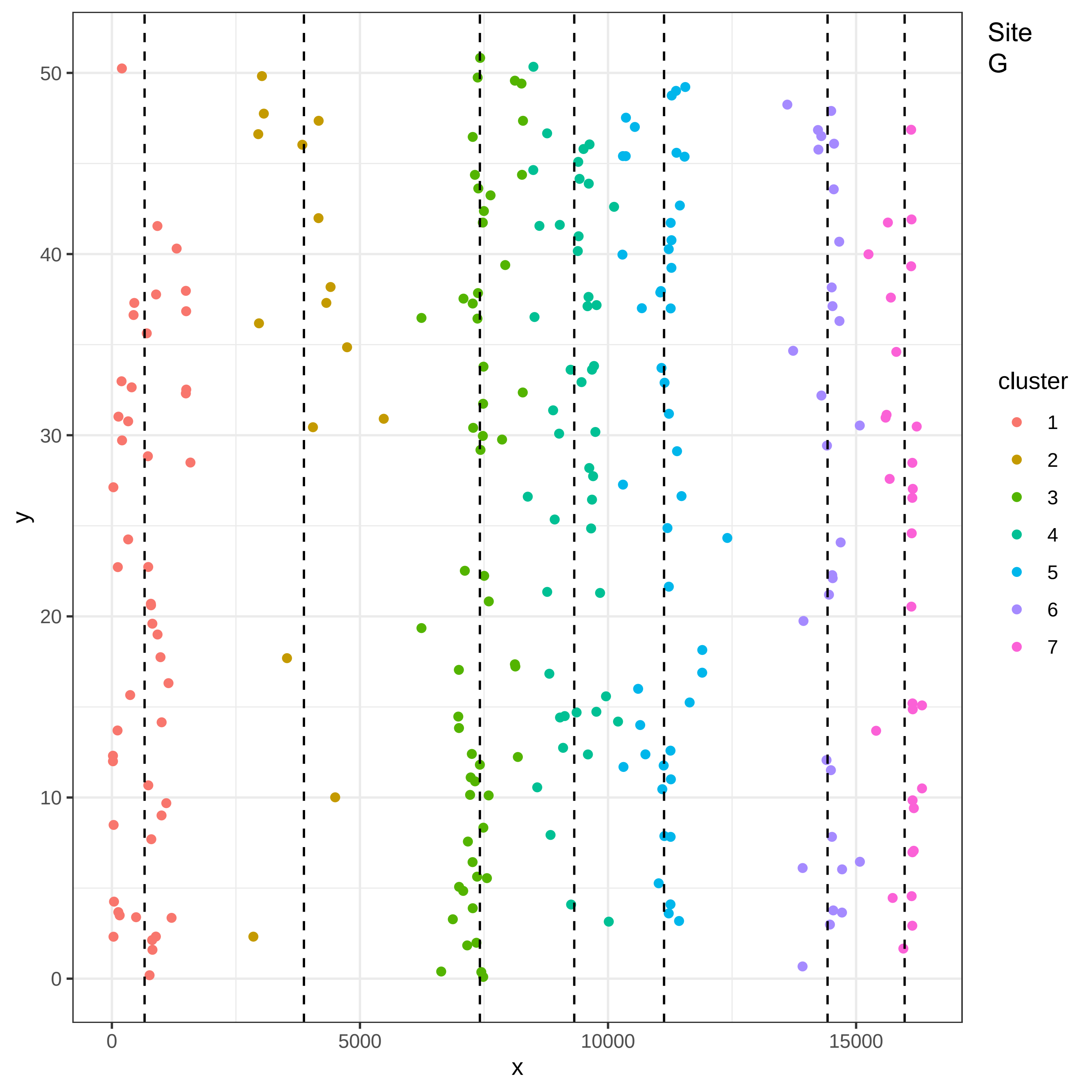
Site
G
cluster
1
2
3
4
5
6
7
y
x
0
10
20
30
40
50
0
5000
10000
15000

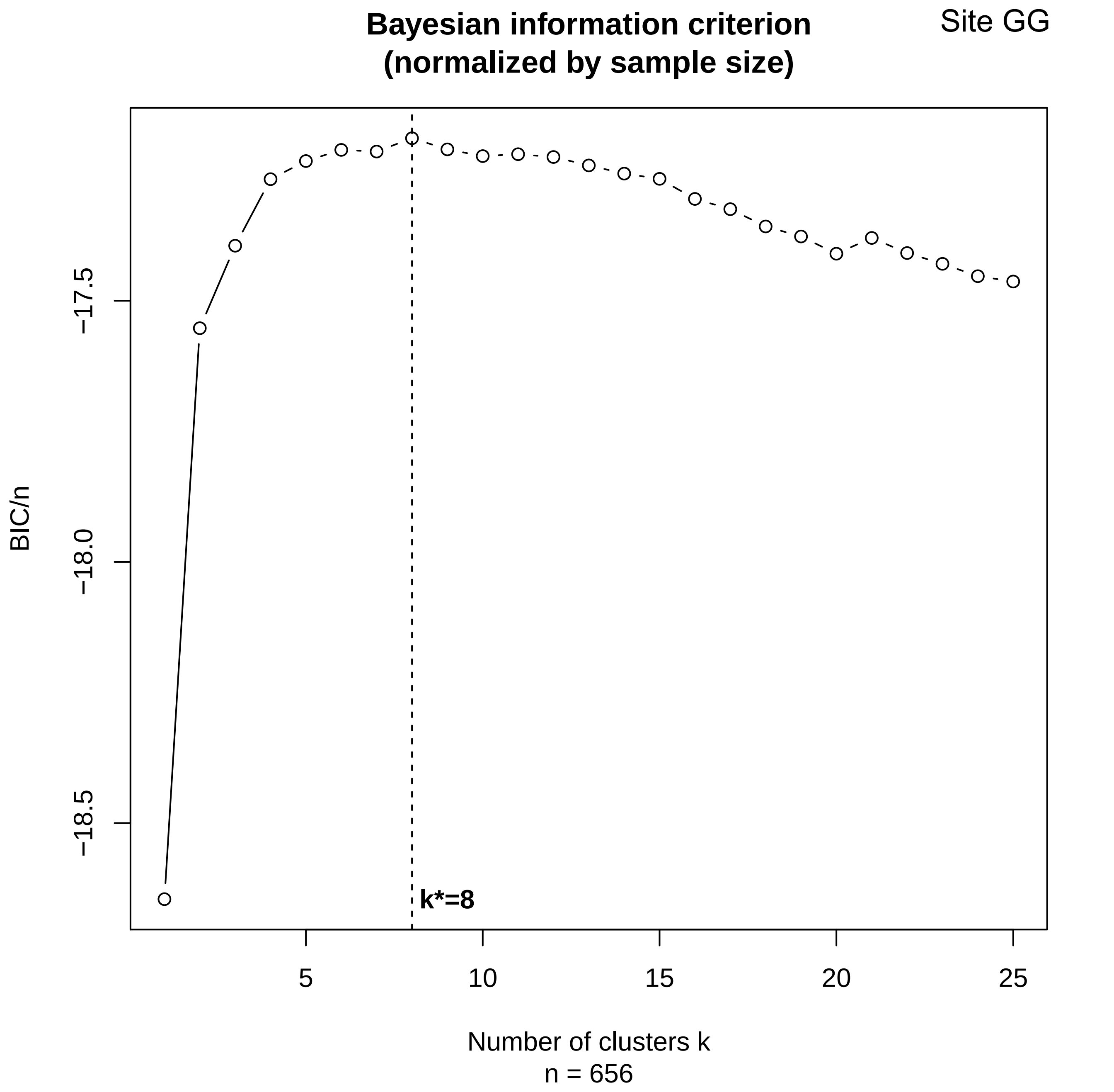

Bayesian information criterion
(normalized by sample size)
Site GG
BIC/n
−17.5
−18.0
−18.5
k*=8
5
10
15
20
25
Number of clusters k
n = 656

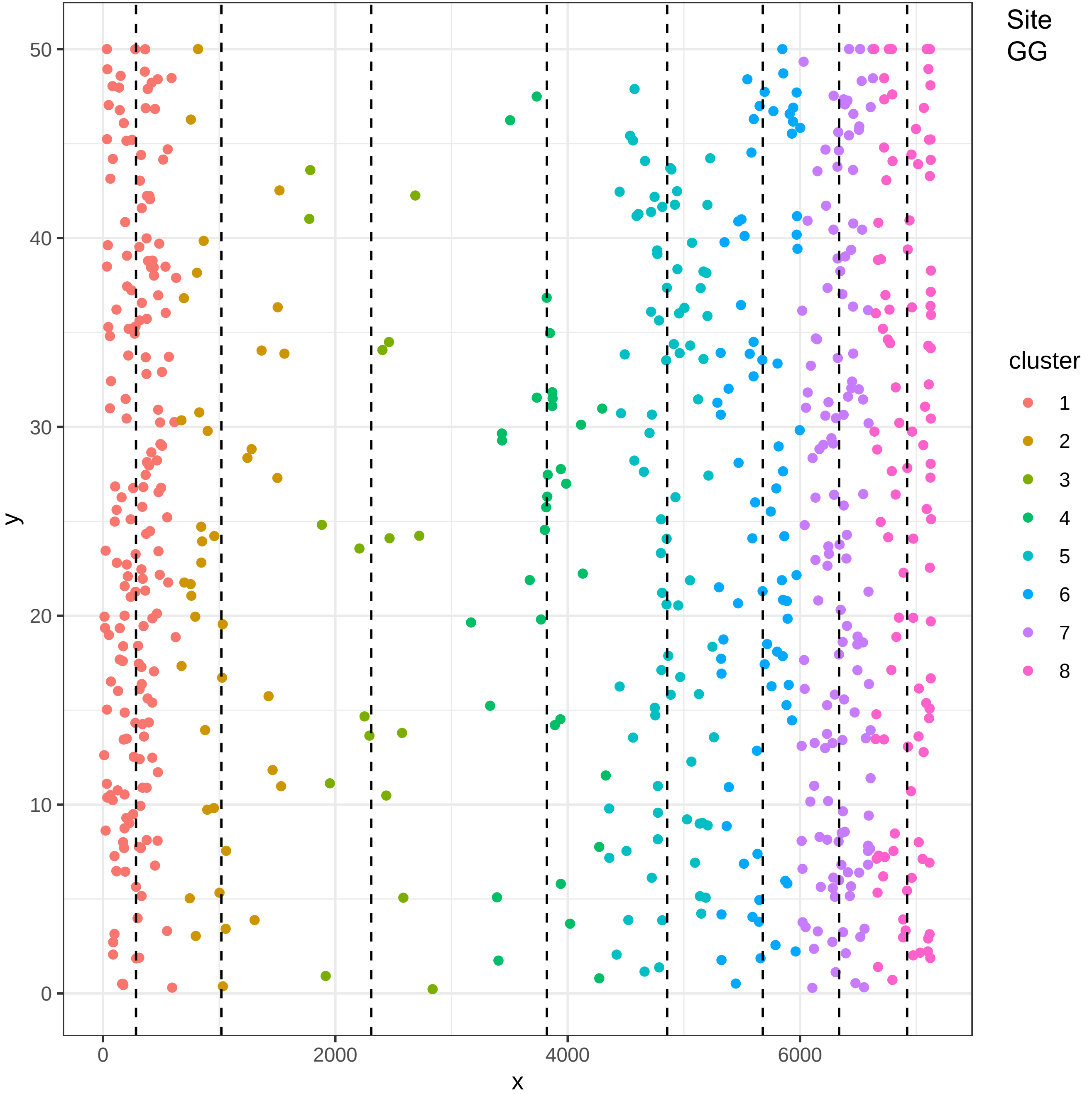

Site
GG
cluster
1
2
3
4
5
6
7
8
y
x
0
10
20
30
40
50
0
2000
4000
6000

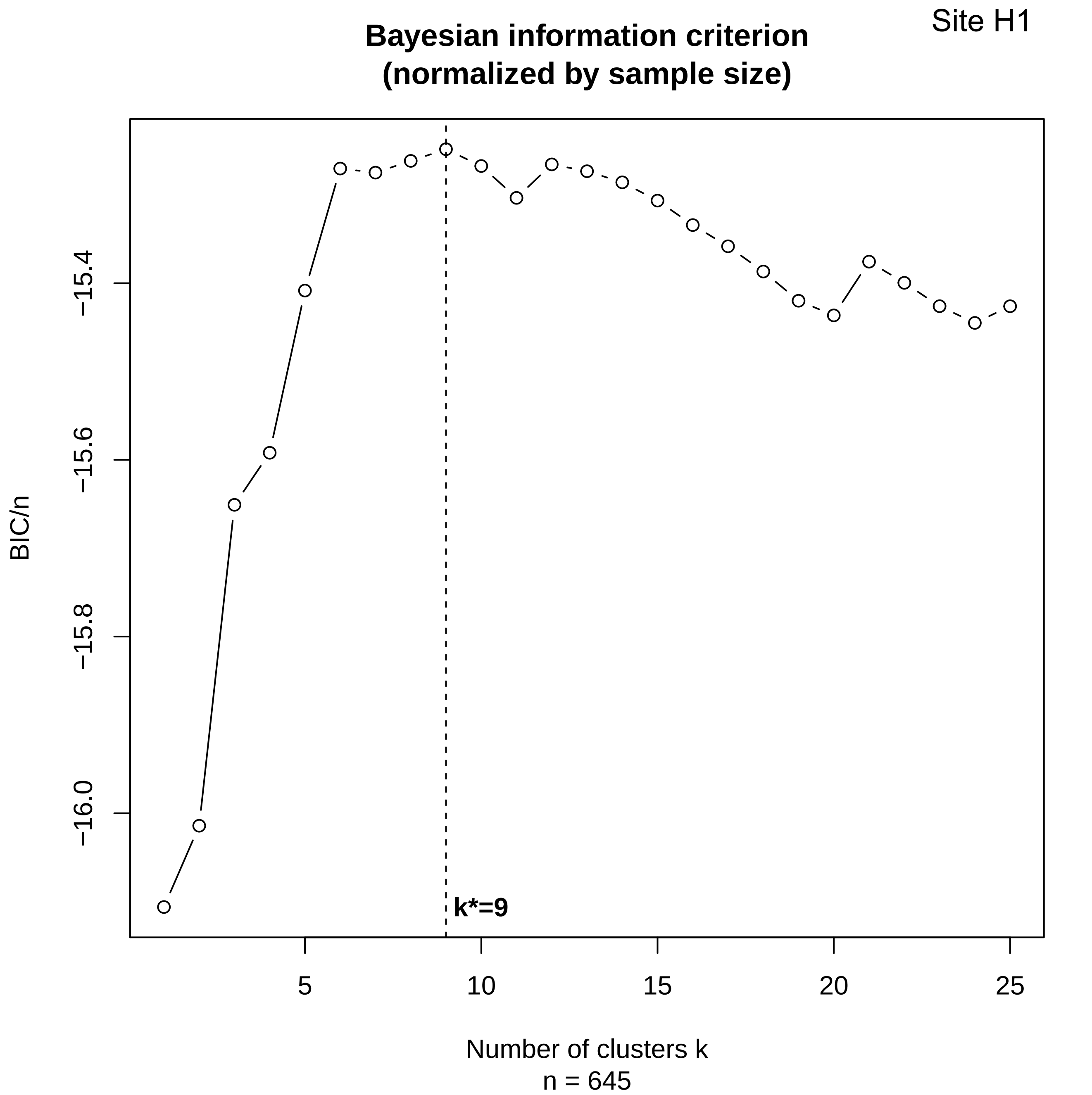
Site H1
Bayesian information criterion
(normalized by sample size)
BIC/n
−15.4
−15.6
−15.8
−16.0
k*=9
5
10
15
20
25
Number of clusters k
n = 645

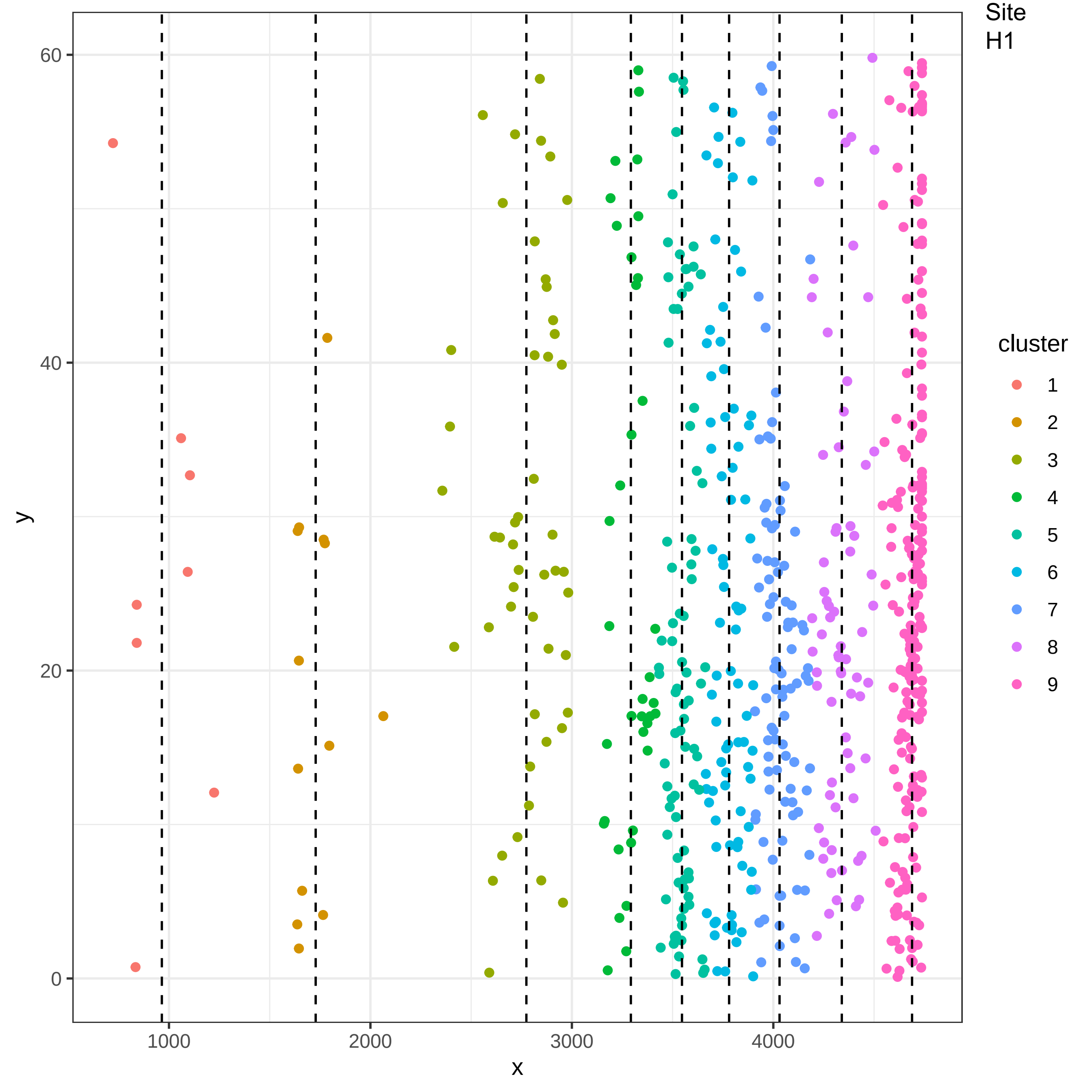

Site
H1
60
40
20
0
y
1000
2000
3000
4000
x
cluster
1
2
3
4
5
6
7
8
9

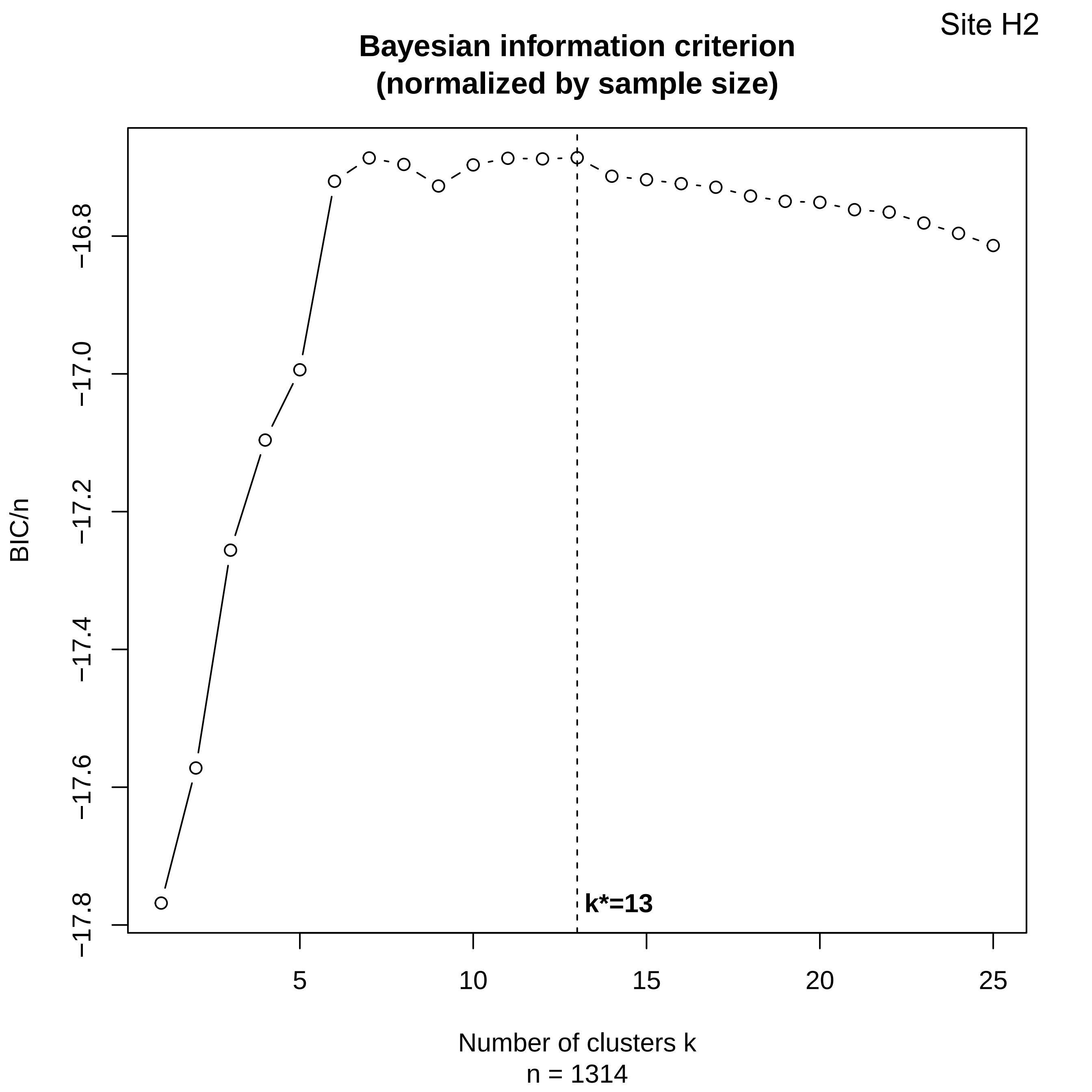
Site H2
Bayesian information criterion
(normalized by sample size)
BIC/n
−16.8
−17.0
−17.2
−17.4
−17.6
−17.8
k*=13
5
10
15
20
25
Number of clusters k
n = 1314

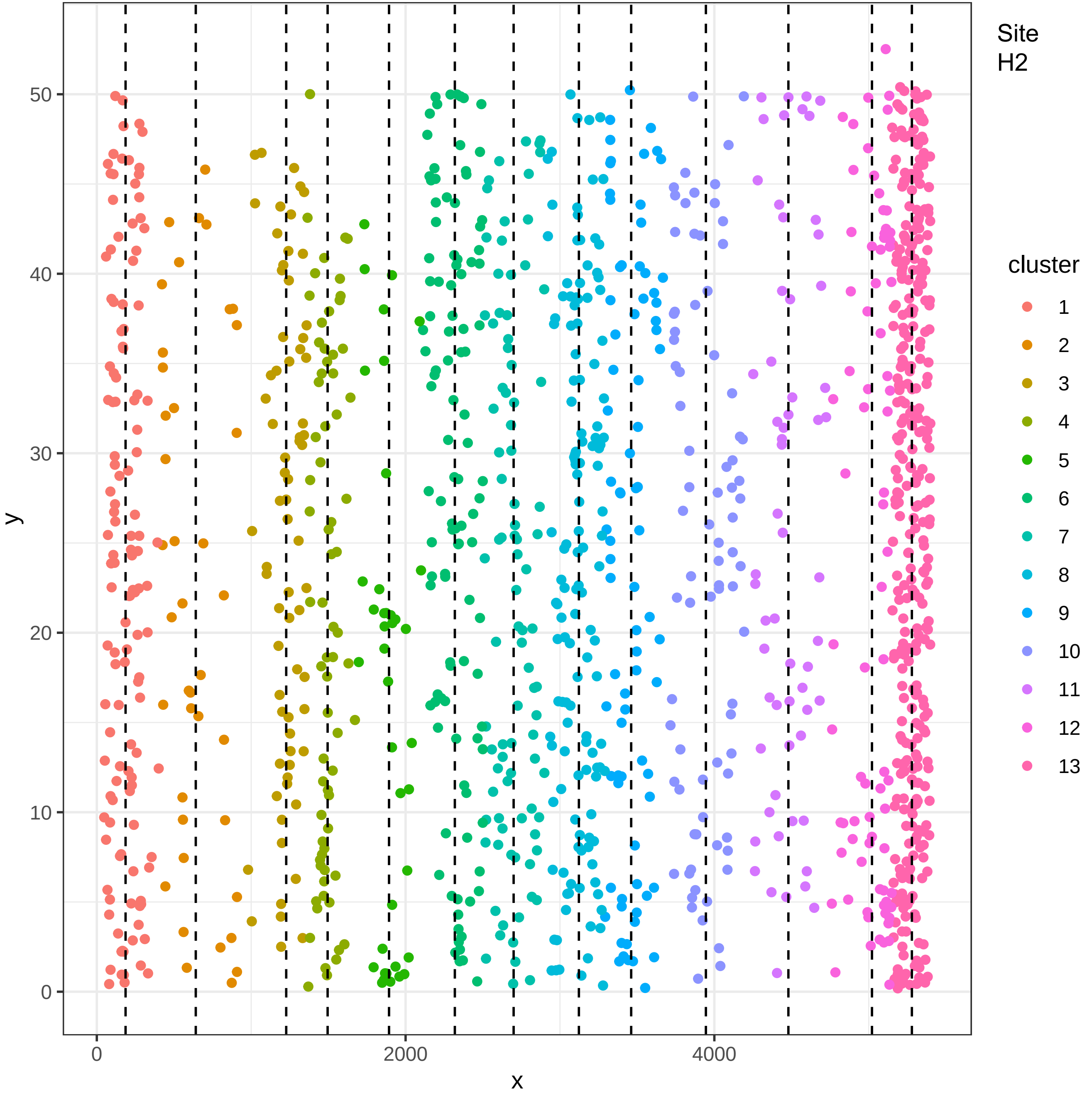
Site
H2
cluster
1
2
3
4
5
6
7
8
9
10
11
12
13
x
y
0
2000
4000
0
10
20
30
40
50

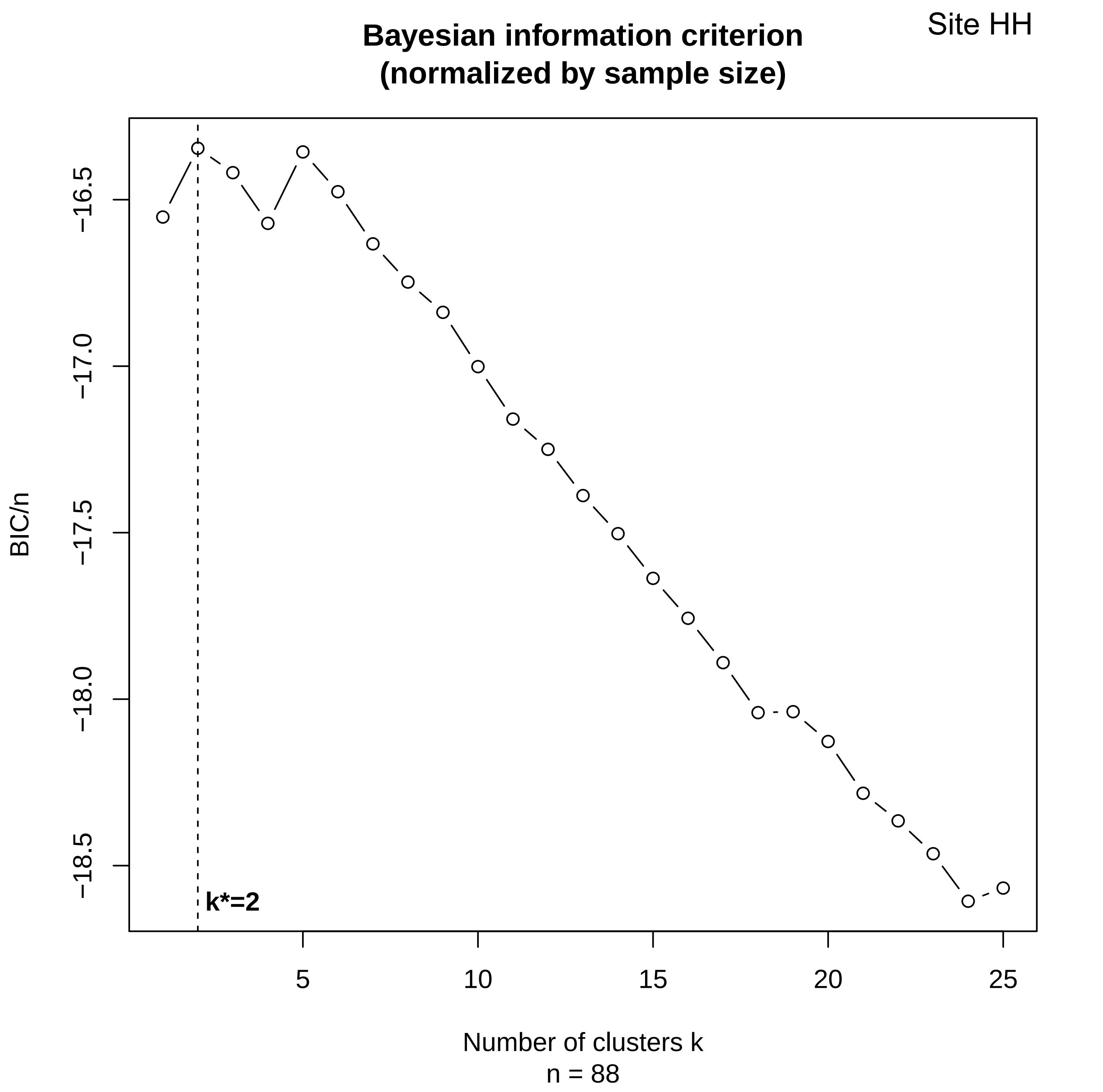
Site HH
Bayesian information criterion
(normalized by sample size)
−16.5
−17.0
−17.5
−18.0
−18.5
BIC/n
k*=2
5
10
15
20
25
Number of clusters k
n = 88

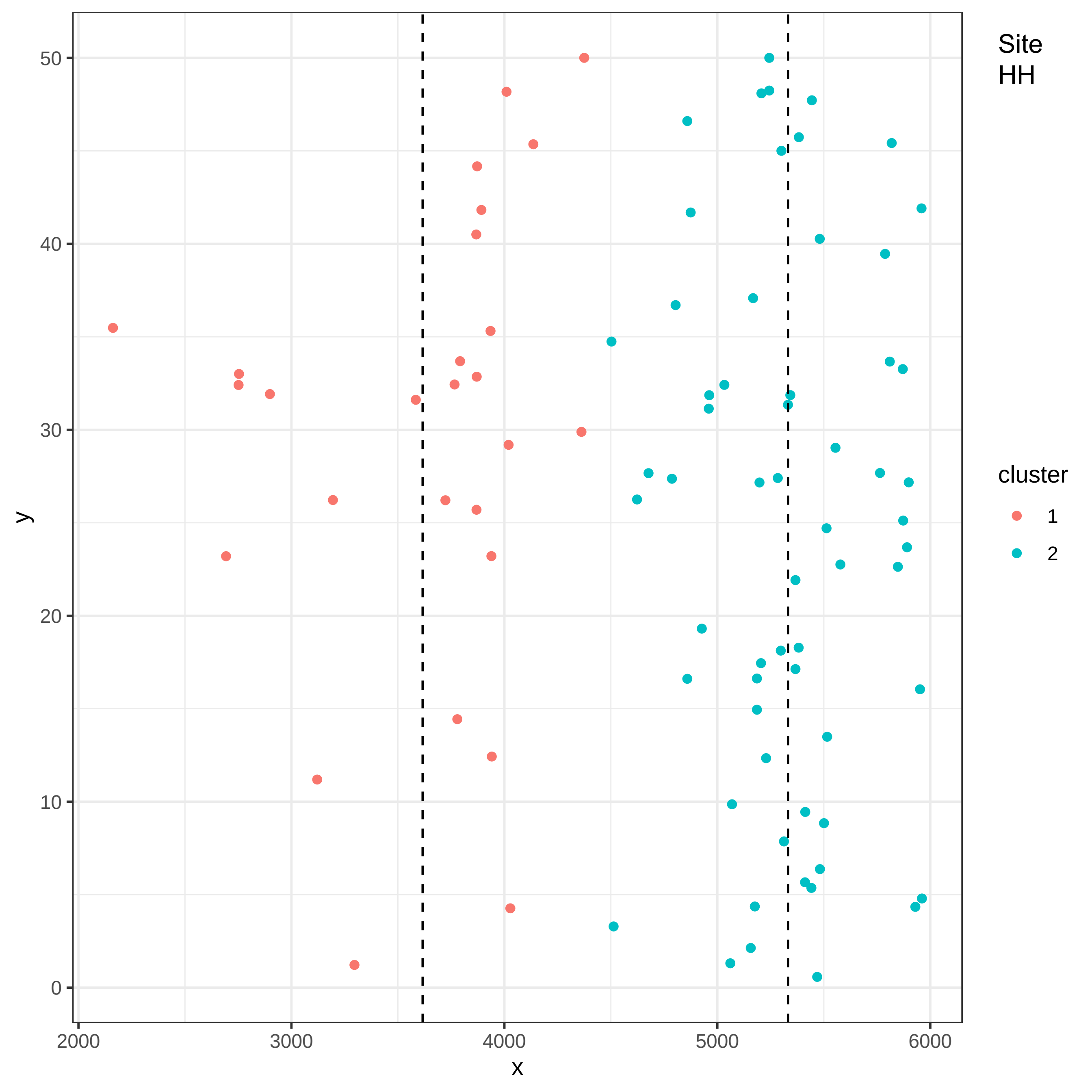
Site
HH
cluster
1
2
y
x
0
10
20
30
40
50
2000
3000
4000
5000
6000

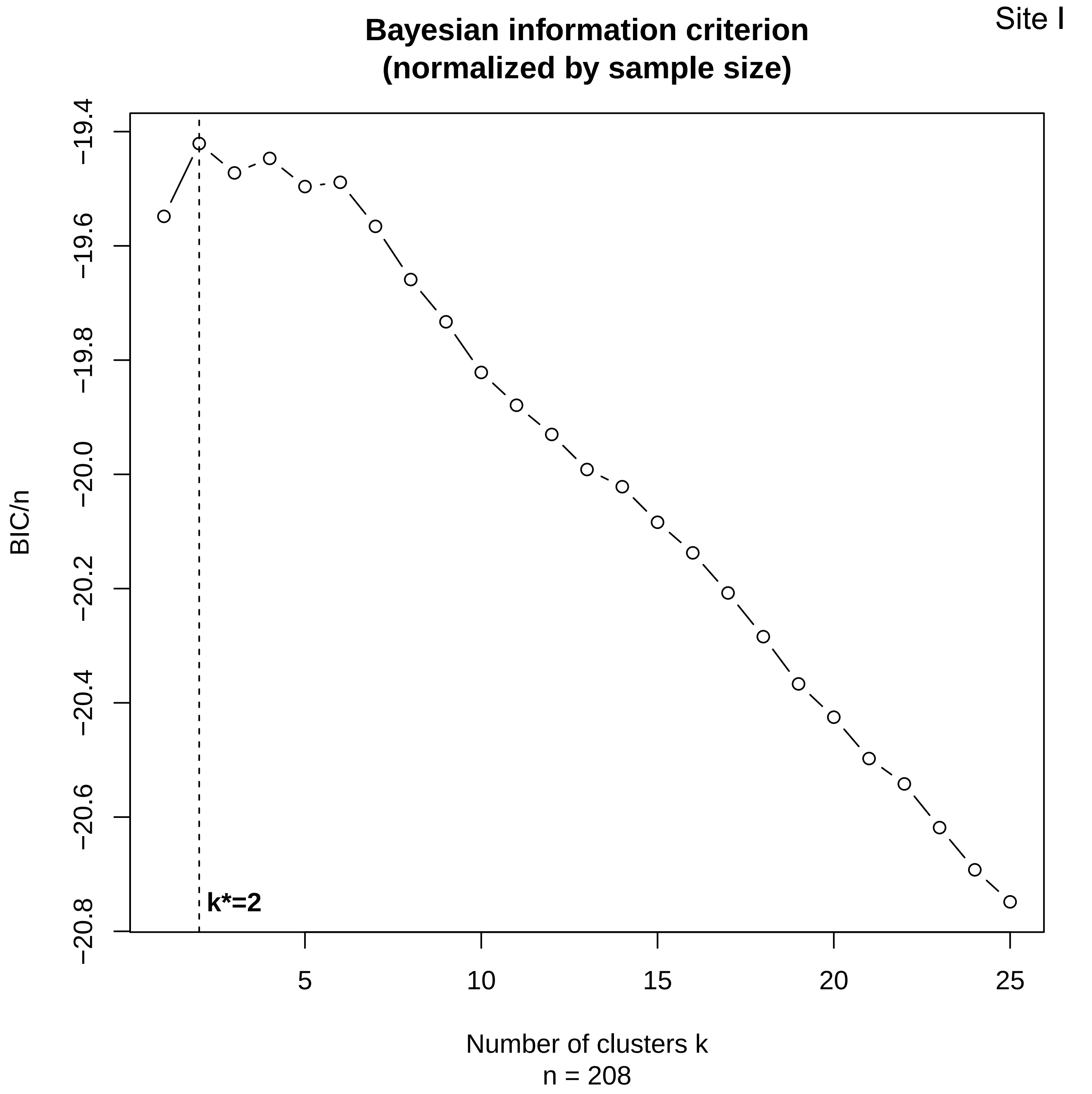

Site I
Bayesian information criterion
(normalized by sample size)
BIC/n
−19.4
−19.6
−19.8
−20.0
−20.2
−20.4
−20.6
−20.8
k*=2
5
10
15
20
25
Number of clusters k
n = 208

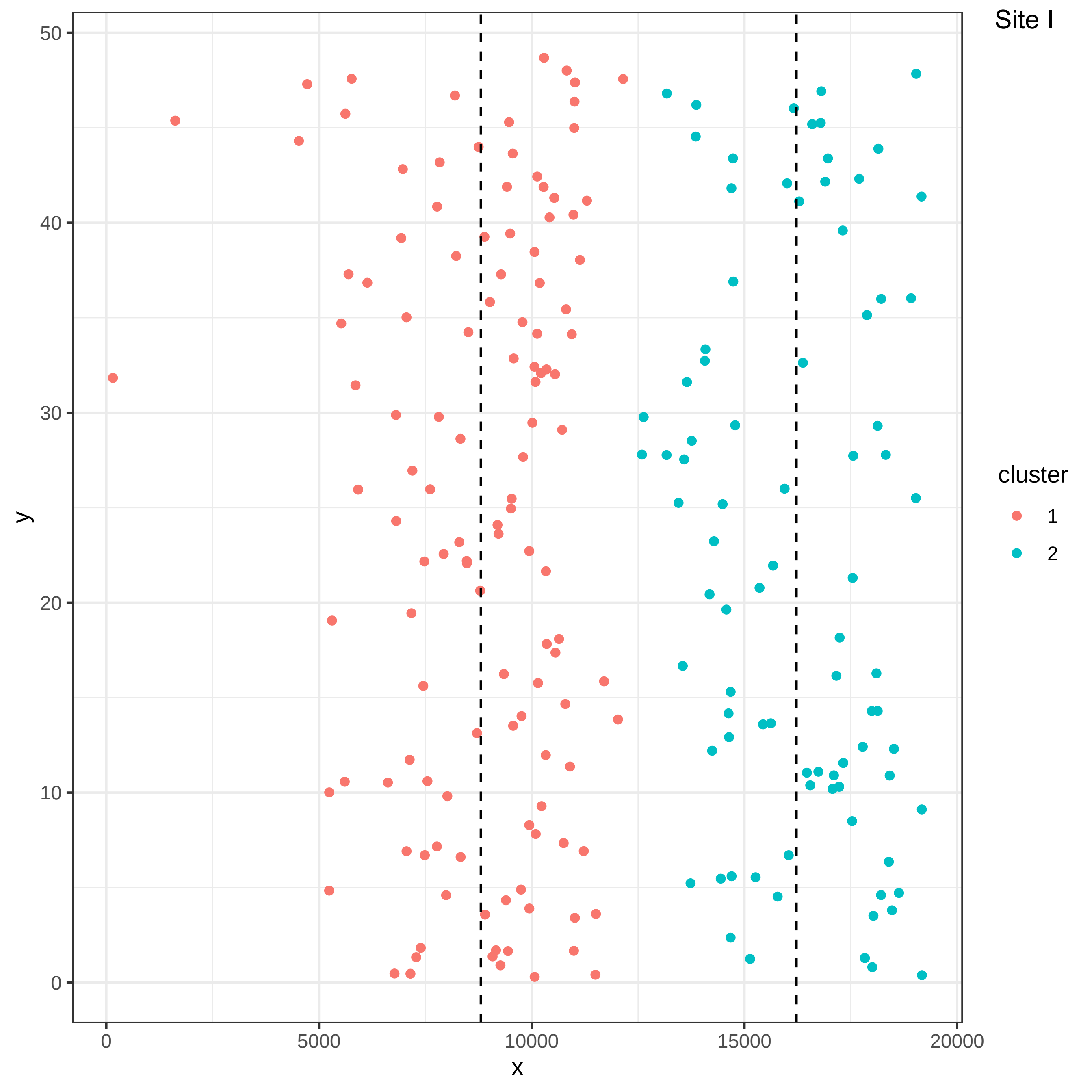

Site I
50
40
30
20
10
0
y
0
5000
10000
15000
20000
x
cluster
1
2

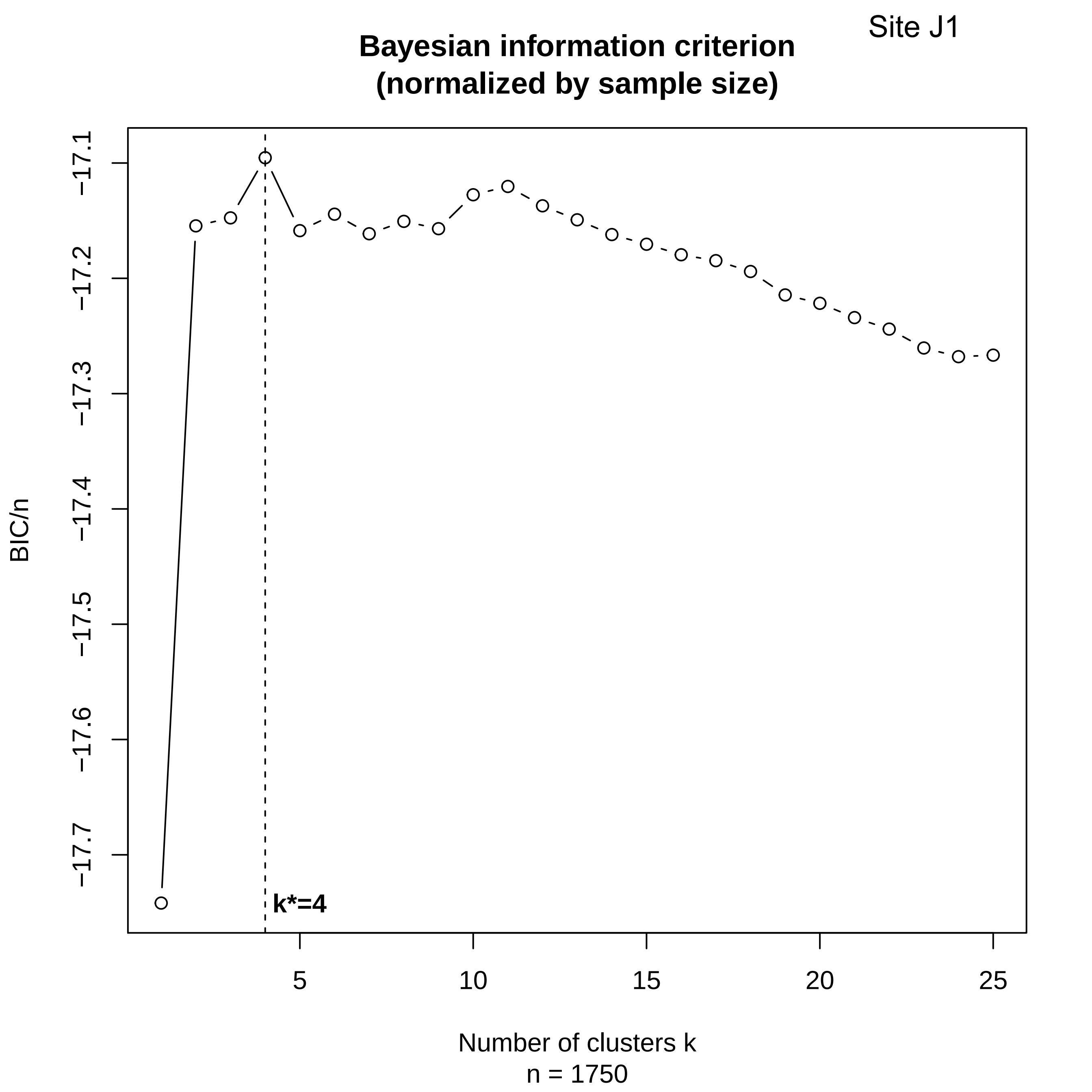
Site J1
Bayesian information criterion
(normalized by sample size)
−17.1
−17.2
−17.3
−17.4
−17.5
−17.6
−17.7
BIC/n
k*=4
5
10
15
20
25
Number of clusters k
n = 1750

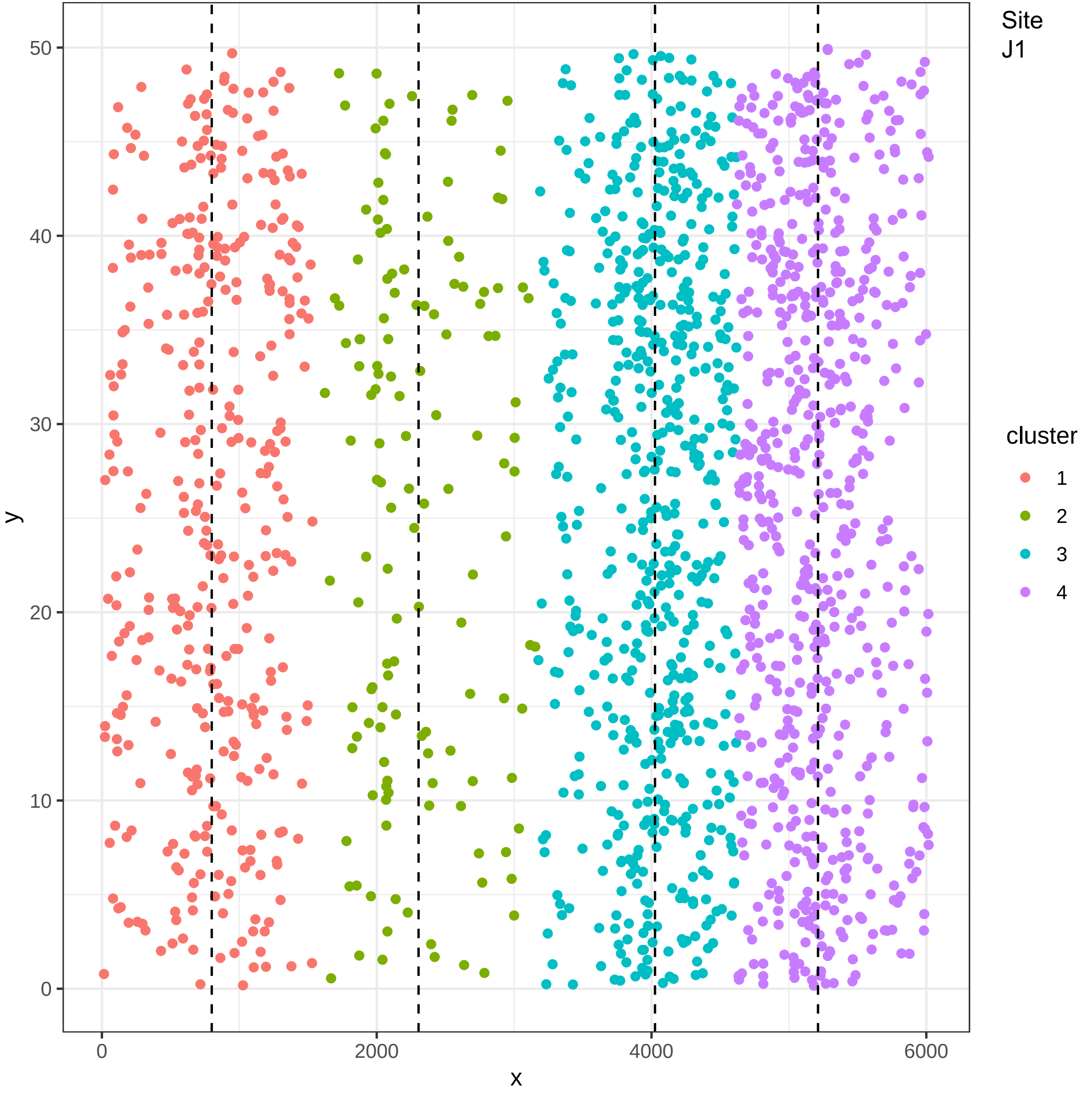

Site
J1
50
40
30
20
10
0
y
0
2000
4000
6000
x
cluster
1
2
3
4

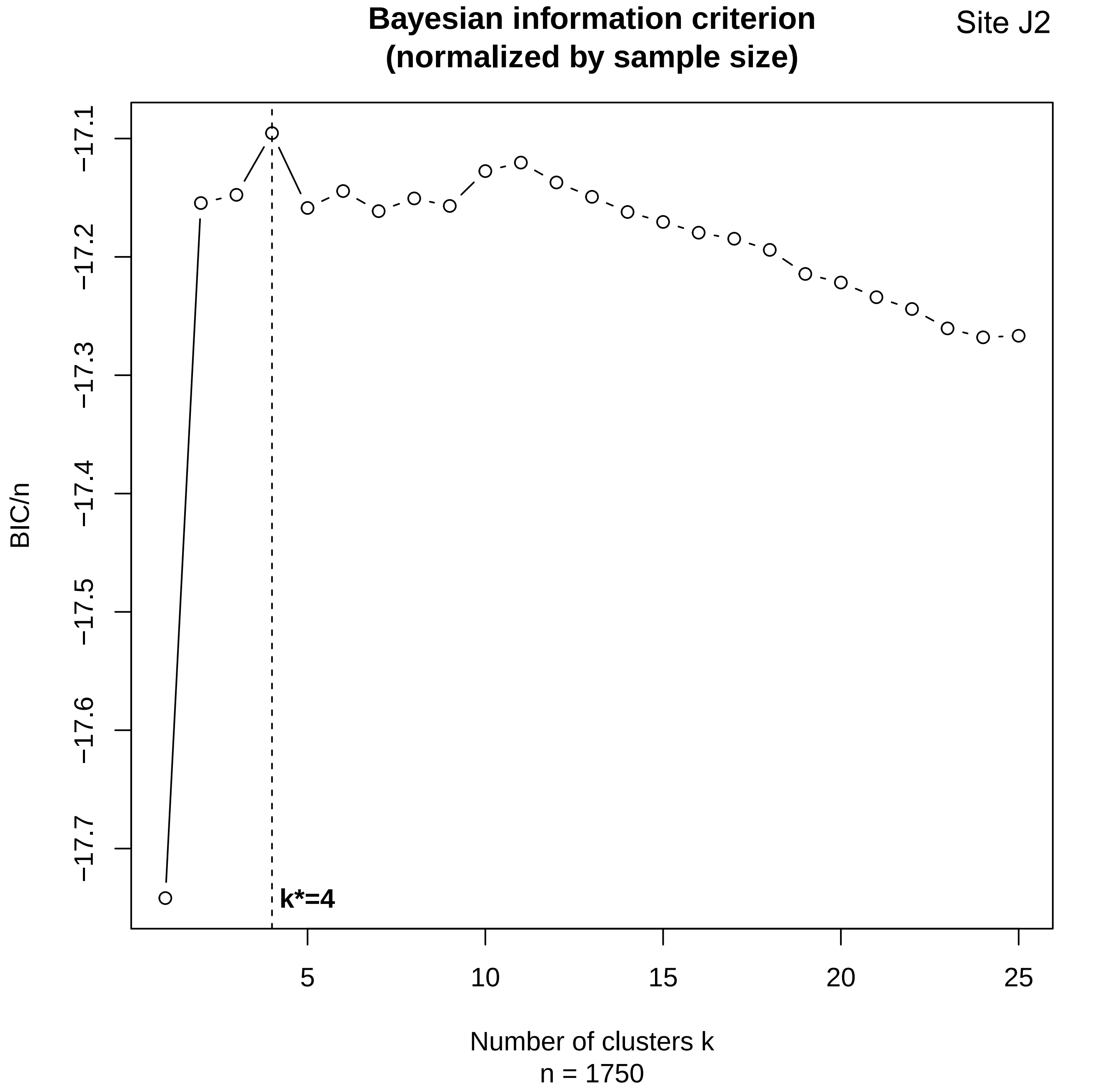

Bayesian information criterion
(normalized by sample size)
Site J2
BIC/n
−17.1
−17.2
−17.3
−17.4
−17.5
−17.6
−17.7
k*=4
5
10
15
20
25
Number of clusters k
n = 1750

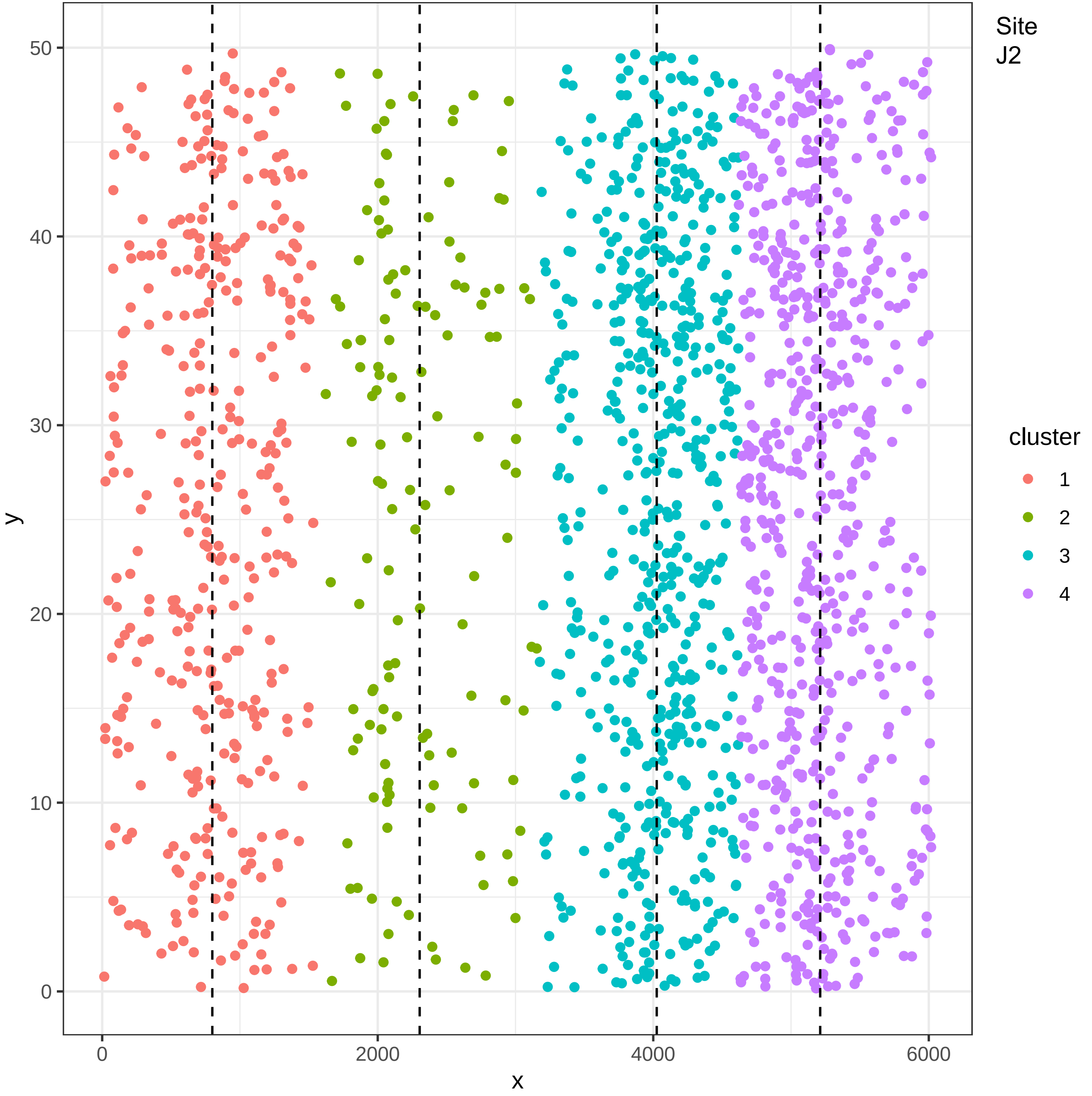

Site
J2
cluster
1
2
3
4
y
x
0
10
20
30
40
50
0
2000
4000
6000

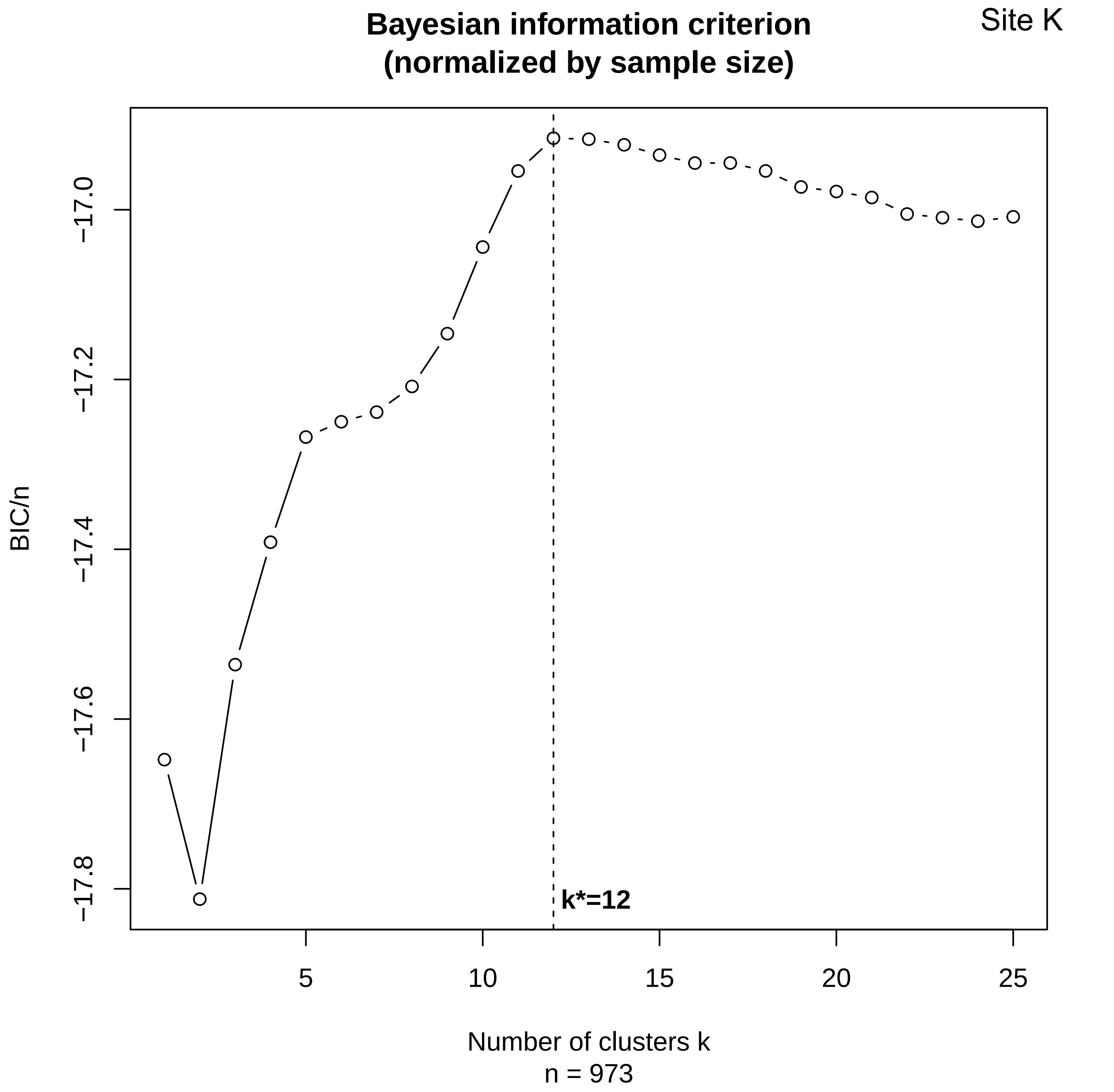
Bayesian information criterion
(normalized by sample size)
Site K
BIC/n
−17.0
−17.2
−17.4
−17.6
−17.8
k*=12
5
10
15
20
25
Number of clusters k
n = 973

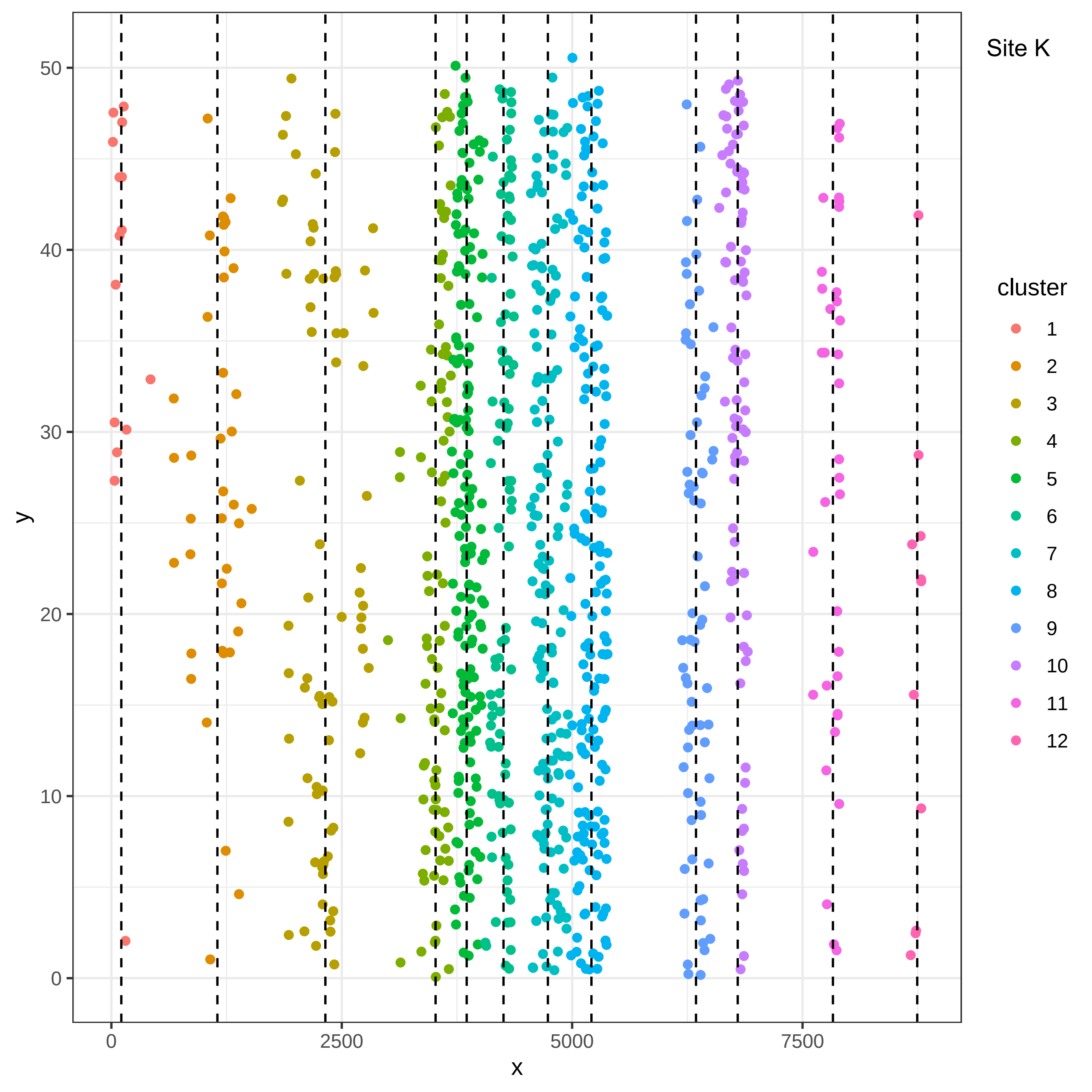

Site K
cluster
1
2
3
4
5
6
7
8
9
10
11
12
y
x
0
10
20
30
40
50
0
2500
5000
7500

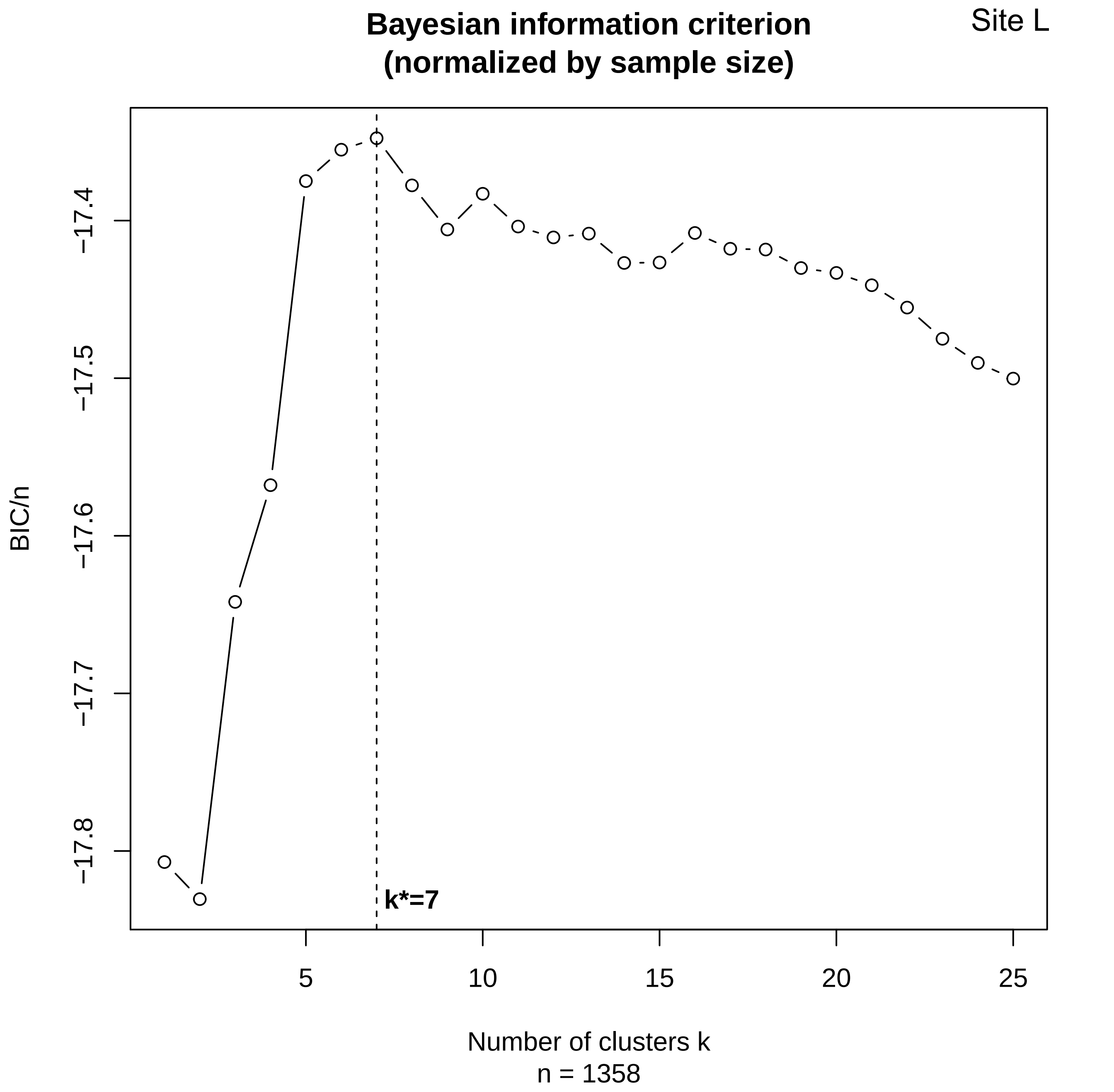
Bayesian information criterion
(normalized by sample size)
Site L
BIC/n
−17.4
−17.5
−17.6
−17.7
−17.8
k*=7
5
10
15
20
25
Number of clusters k
n = 1358

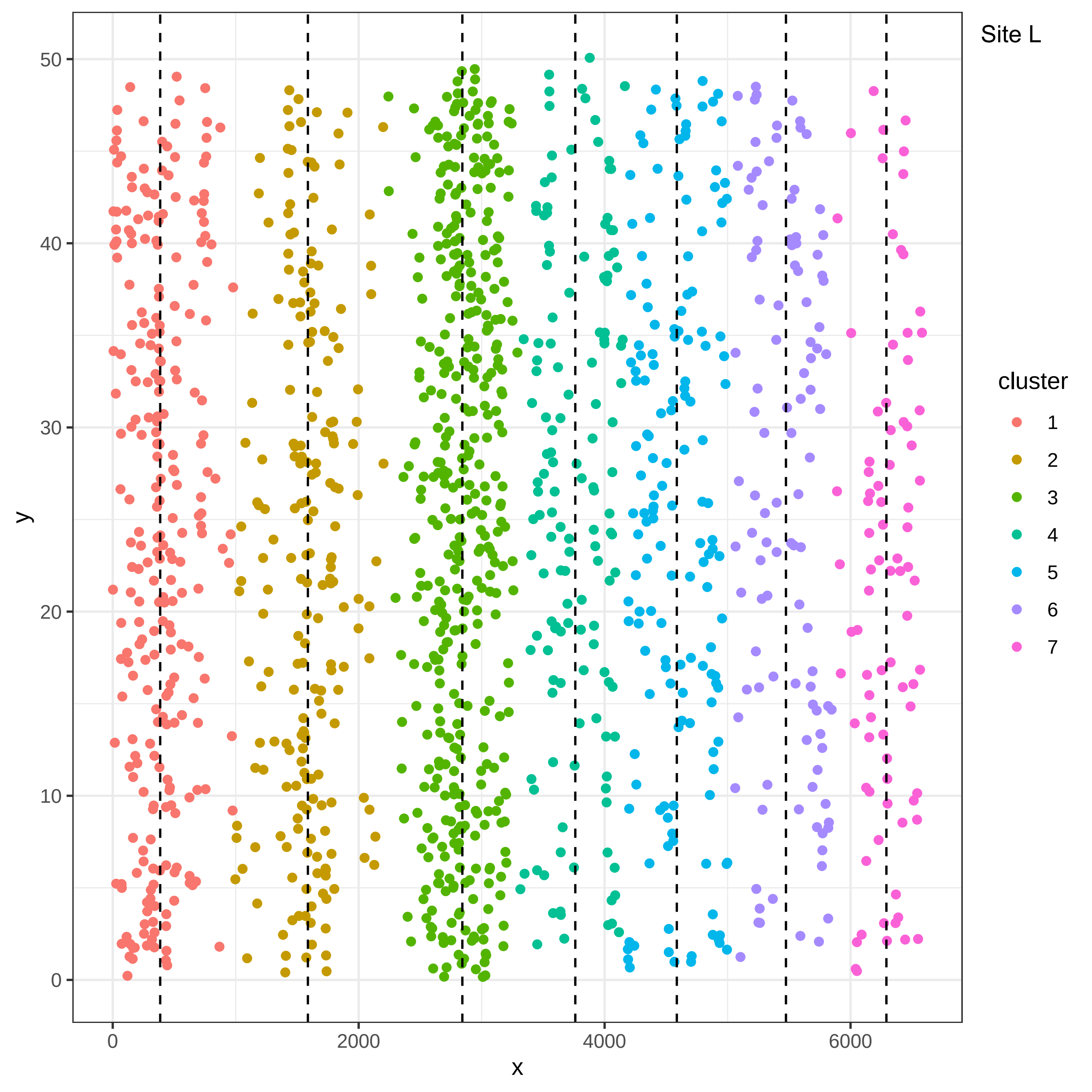

Site L
cluster
1
2
3
4
5
6
7
y
x
0
10
20
30
40
50
0
2000
4000
6000

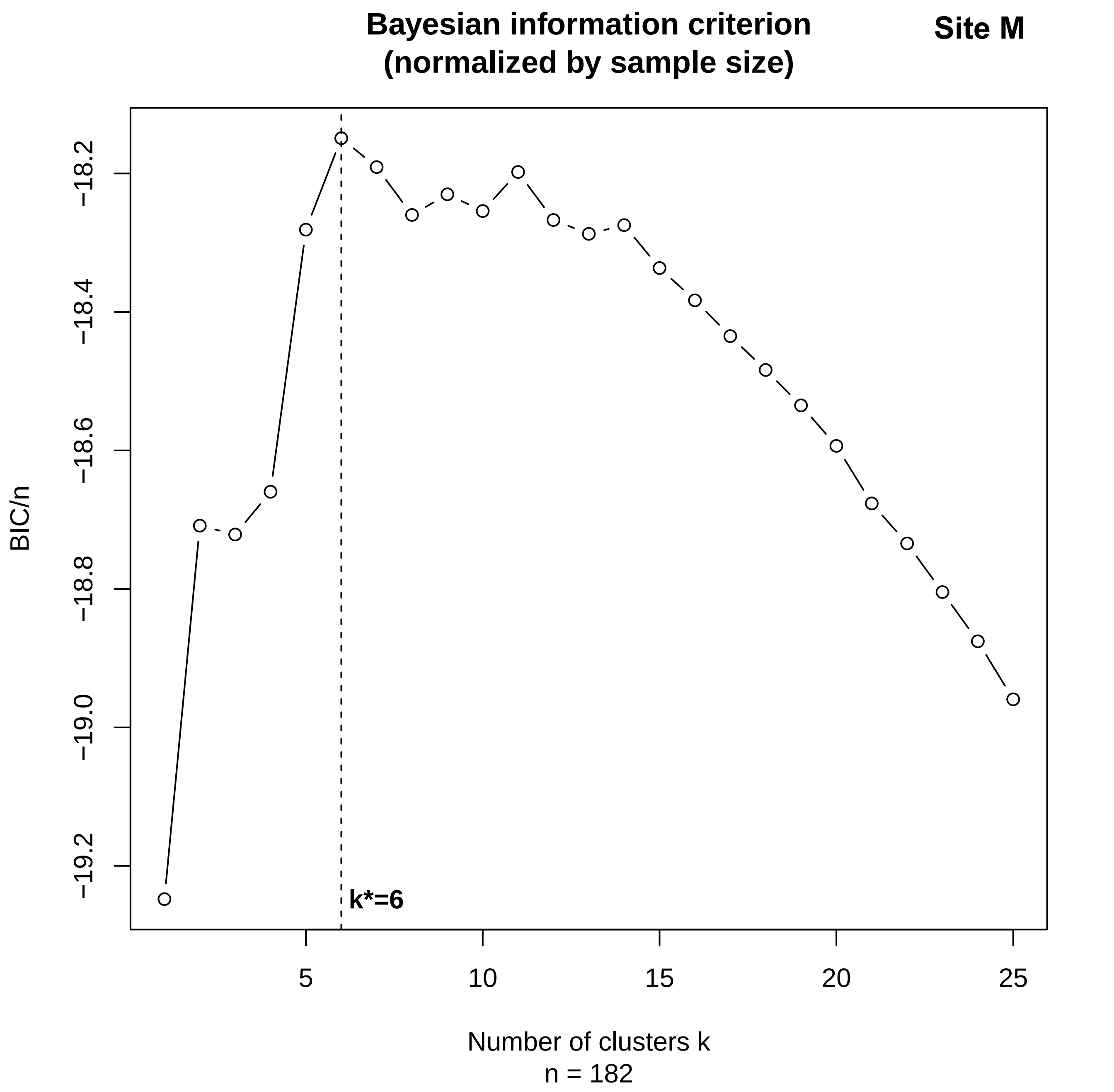

Bayesian information criterion
(normalized by sample size)
Site M
BIC/n
−18.2
−18.4
−18.6
−18.8
−19.0
−19.2
k*=6
5
10
15
20
25
Number of clusters k
n = 182

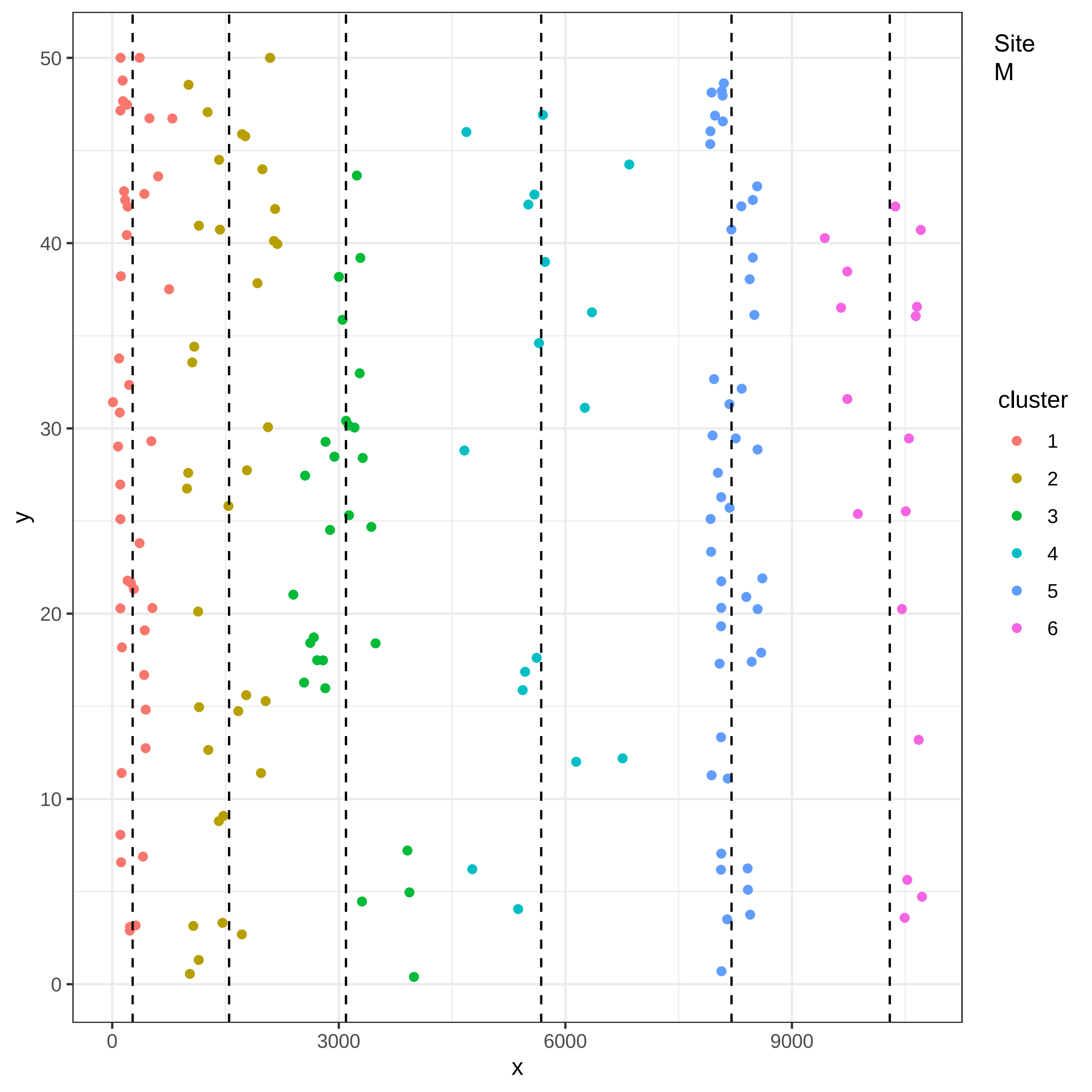

Site
M
cluster
1
2
3
4
5
6
y
x
0
10
20
30
40
50
0
3000
6000
9000

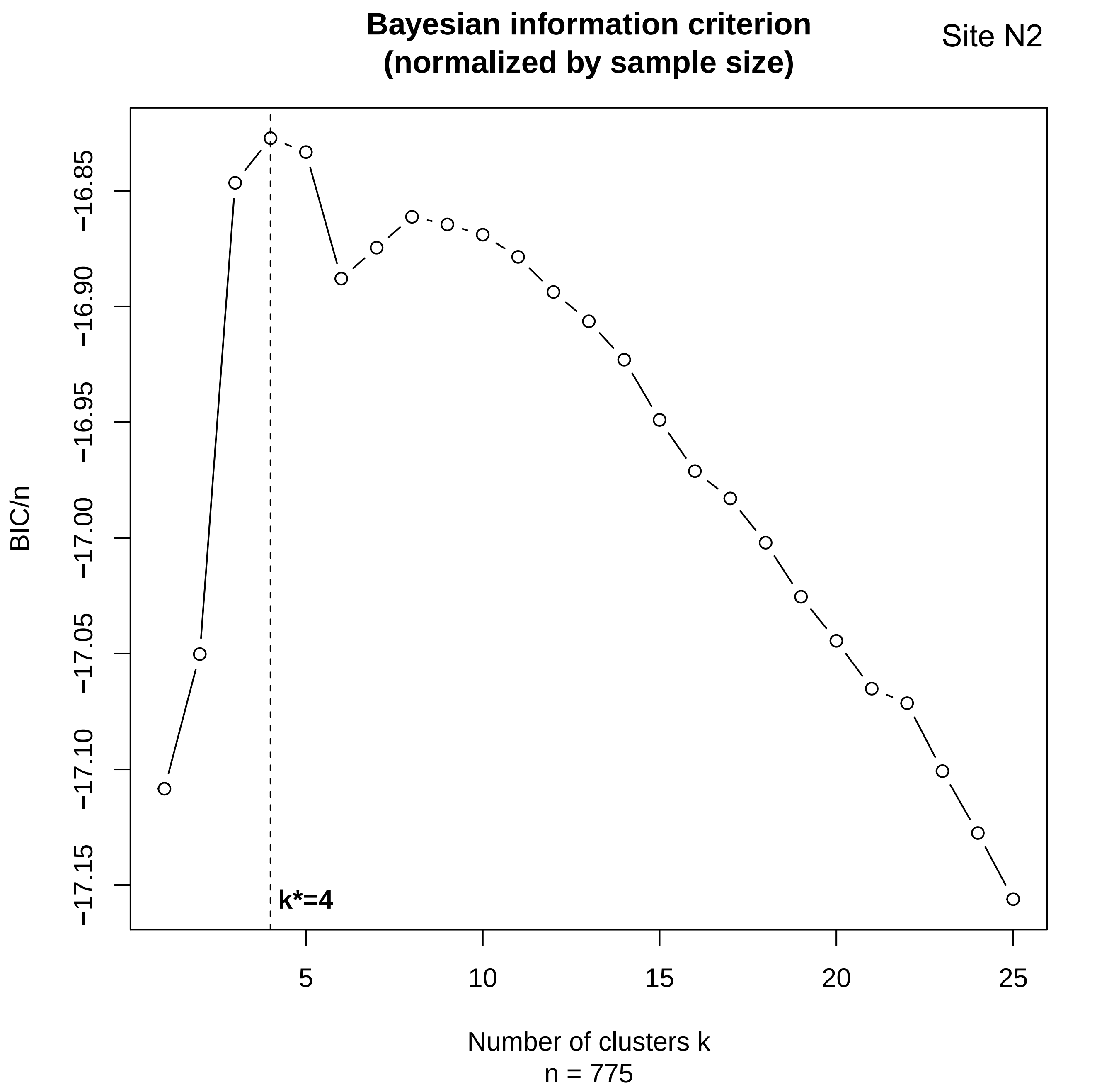

Bayesian information criterion
(normalized by sample size)
Site N2
BIC/n
−16.85
−16.90
−16.95
−17.00
−17.05
−17.10
−17.15
k*=4
5
10
15
20
25
Number of clusters k
n = 775

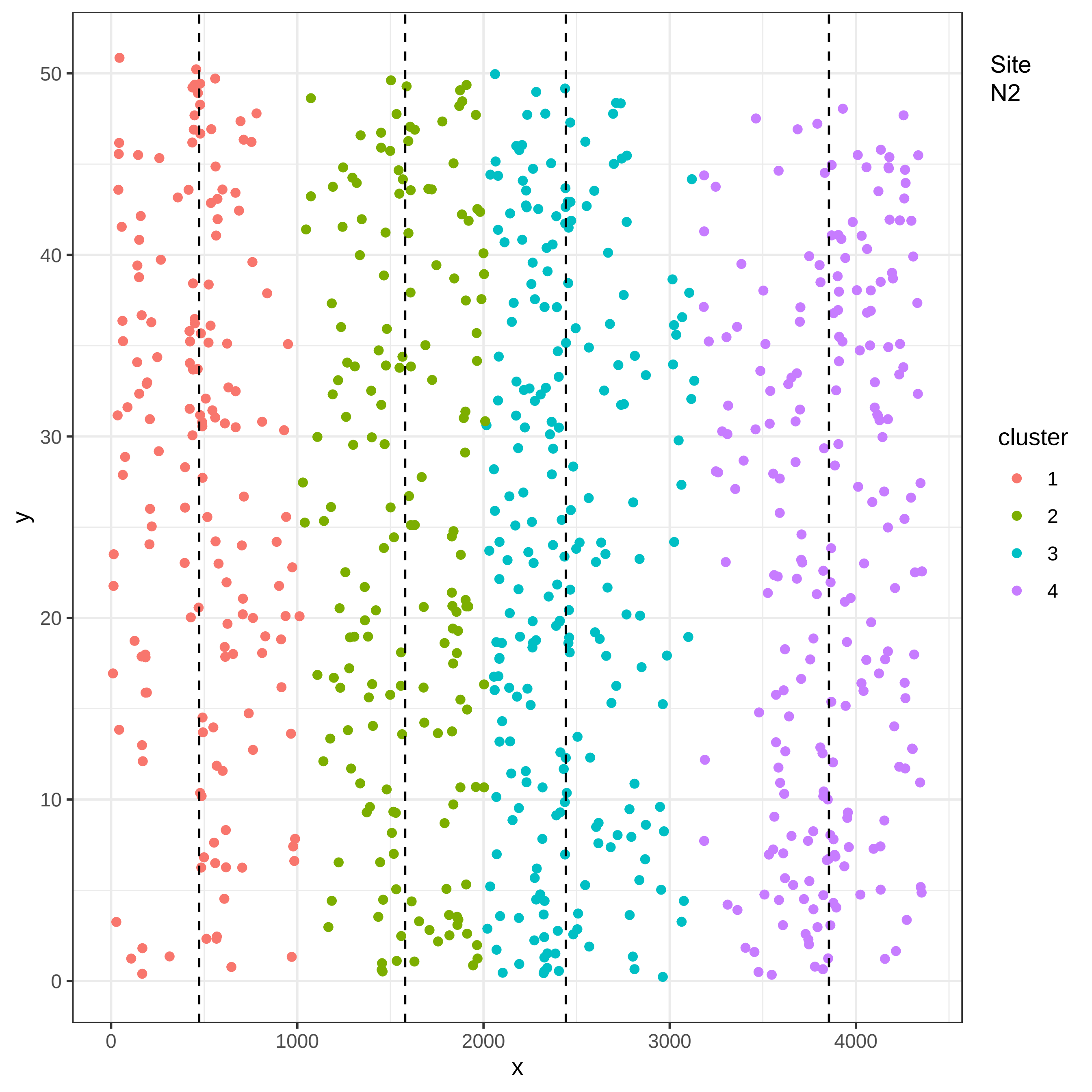

Site
N2
cluster
1
2
3
4
y
x
0
10
20
30
40
50
0
1000
2000
3000
4000

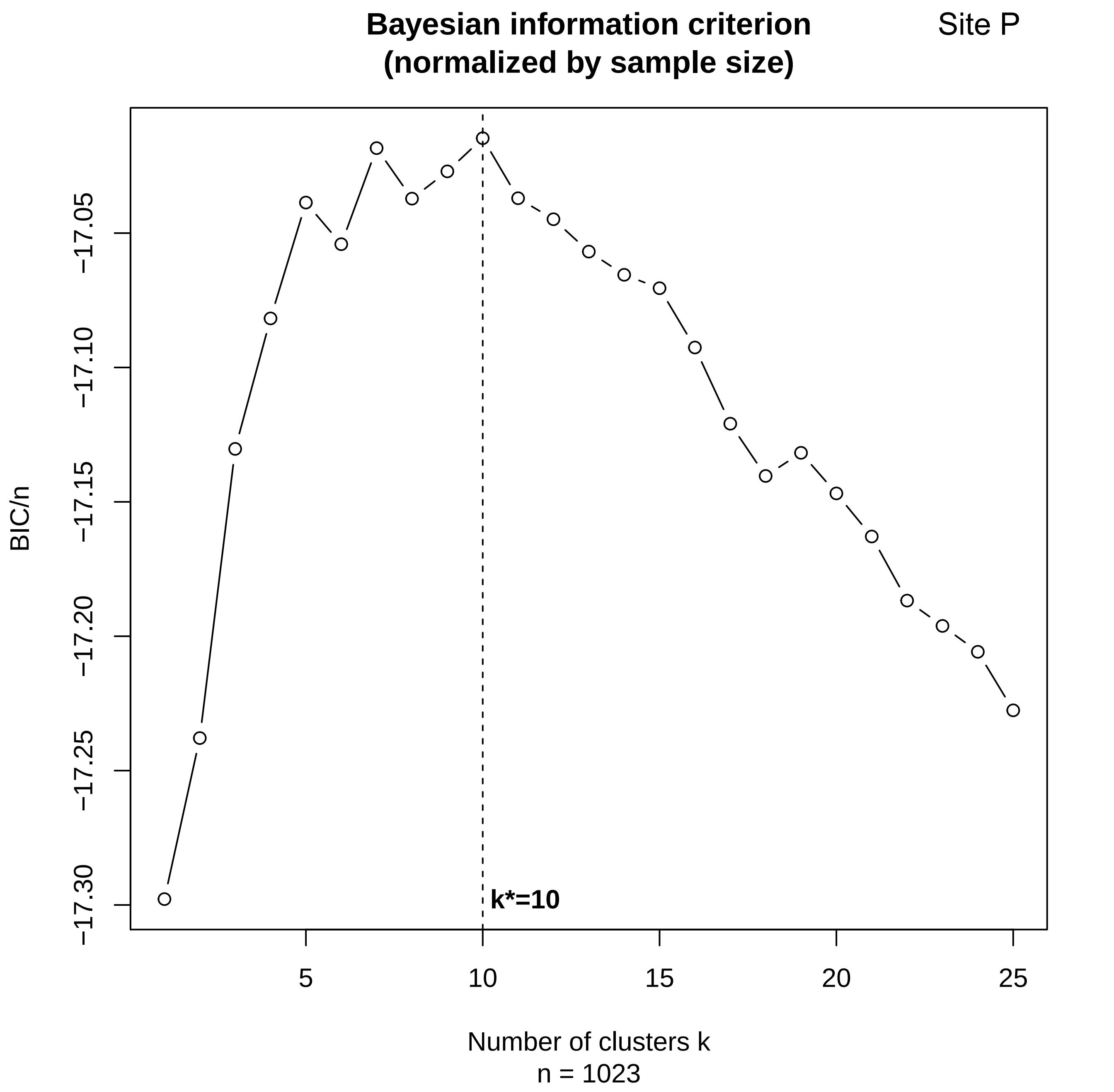

Bayesian information criterion
(normalized by sample size)
Site P
BIC/n
−17.05
−17.10
−17.15
−17.20
−17.25
−17.30
k*=10
5
10
15
20
25
Number of clusters k
n = 1023

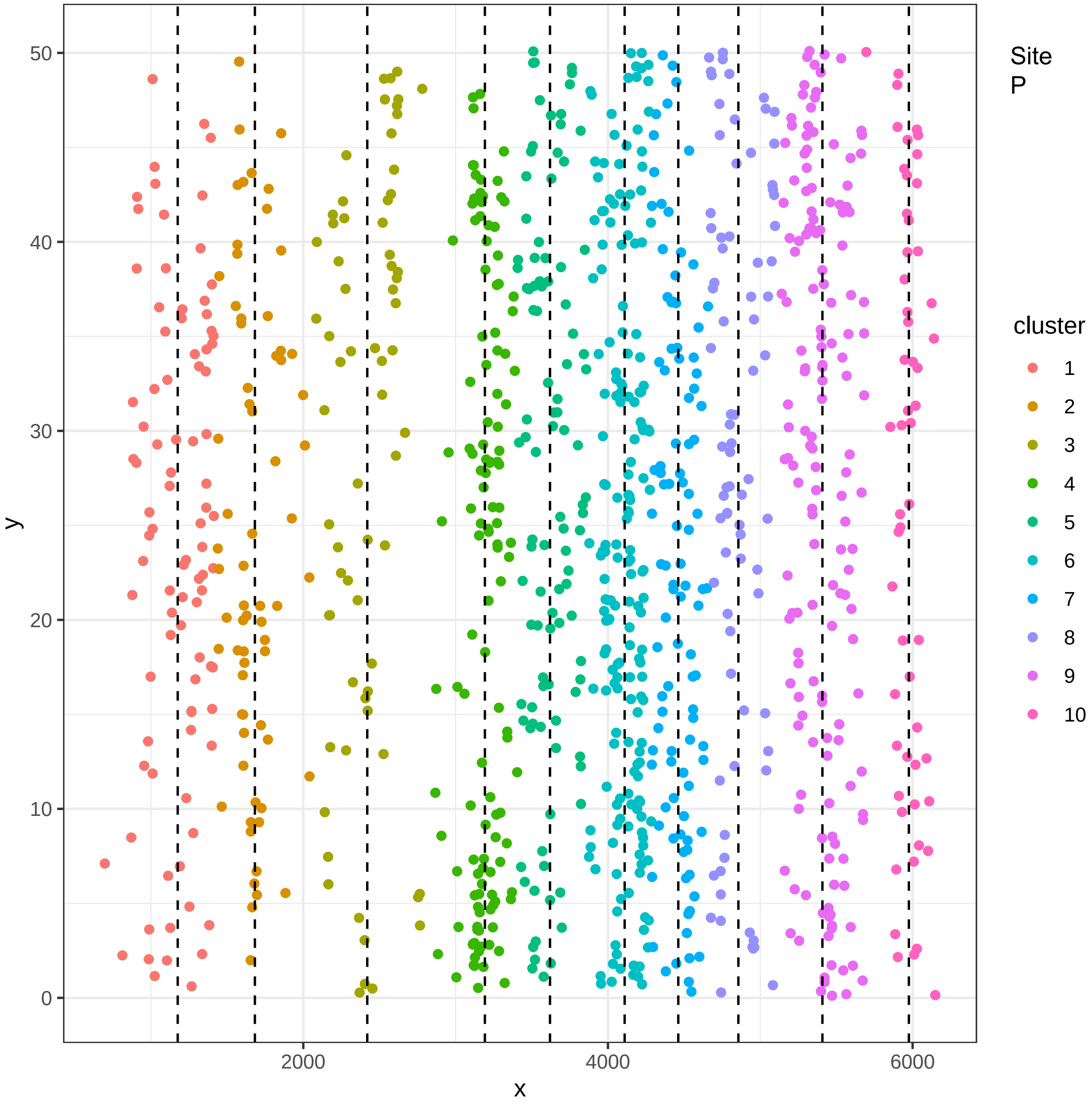

Site
P
cluster
1
2
3
4
5
6
7
8
9
10
y
x
0
10
20
30
40
50
2000
4000
6000

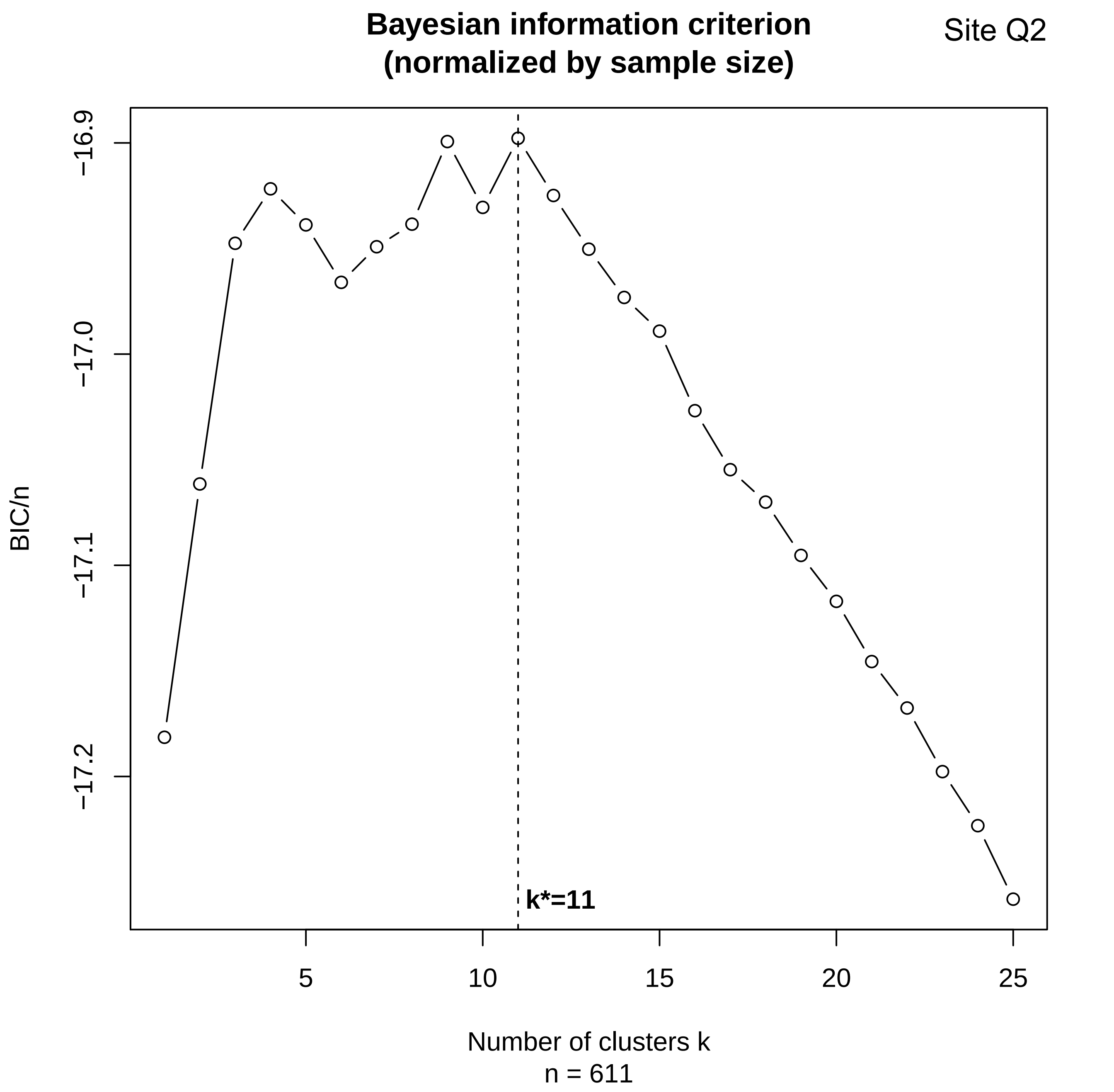

Bayesian information criterion
(normalized by sample size)
Site Q2
BIC/n
−16.9
−17.0
−17.1
−17.2
k*=11
5
10
15
20
25
Number of clusters k
n = 611

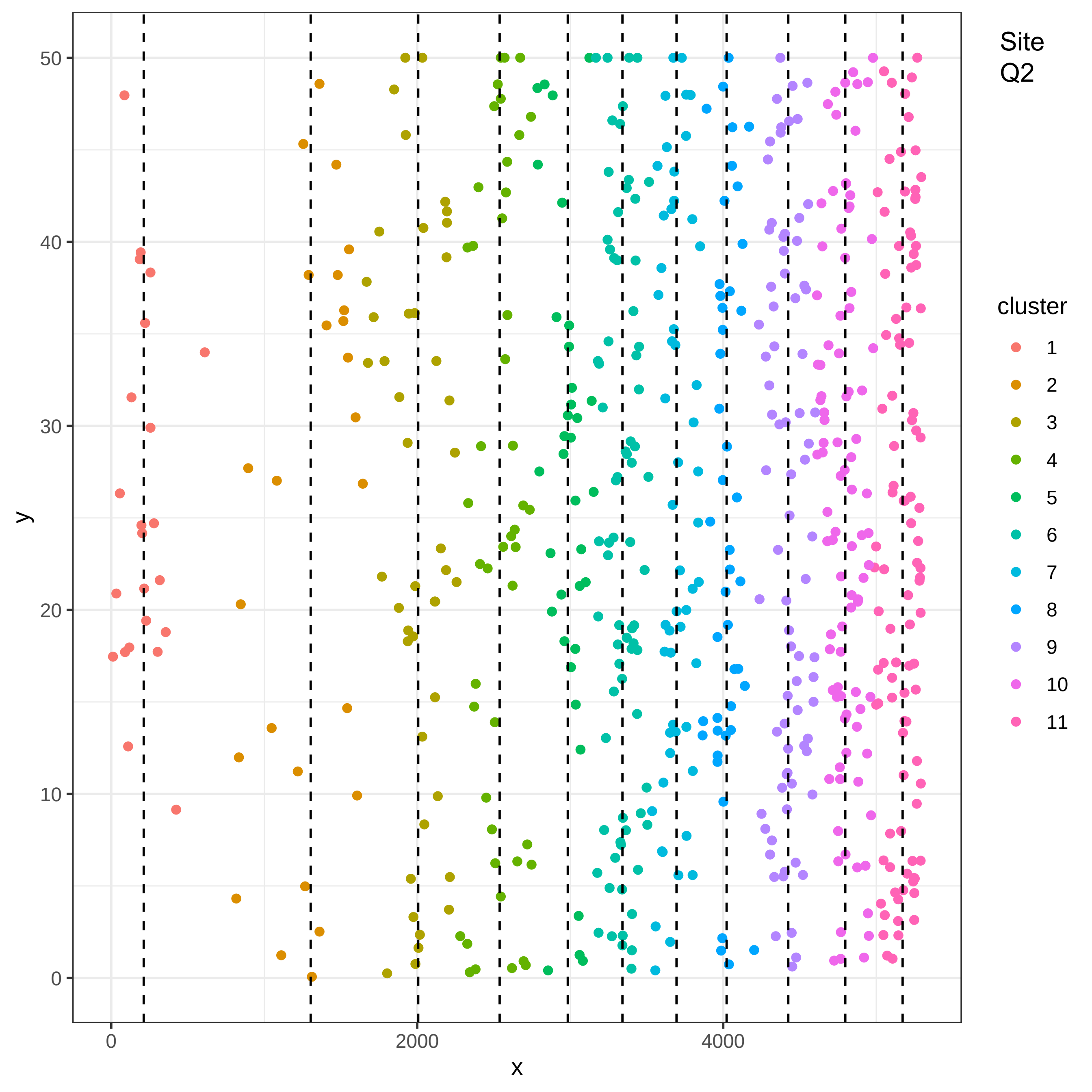

Site
Q2
cluster
1
2
3
4
5
6
7
8
9
10
11
y
x
0
10
20
30
40
50
0
2000
4000

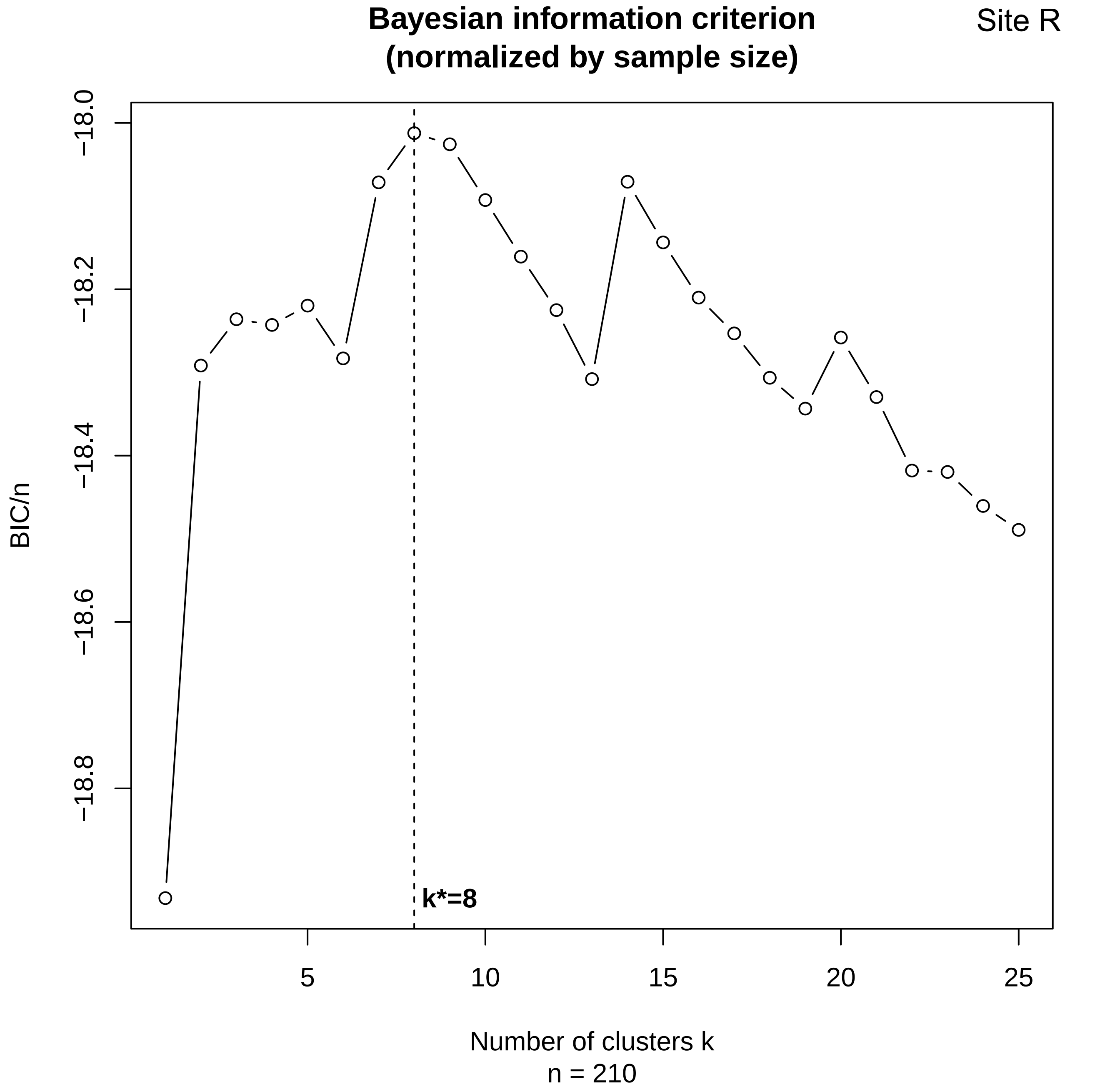
Bayesian information criterion
(normalized by sample size)
Site R
BIC/n
−18.0
−18.2
−18.4
−18.6
−18.8
k*=8
5
10
15
20
25
Number of clusters k
n = 210

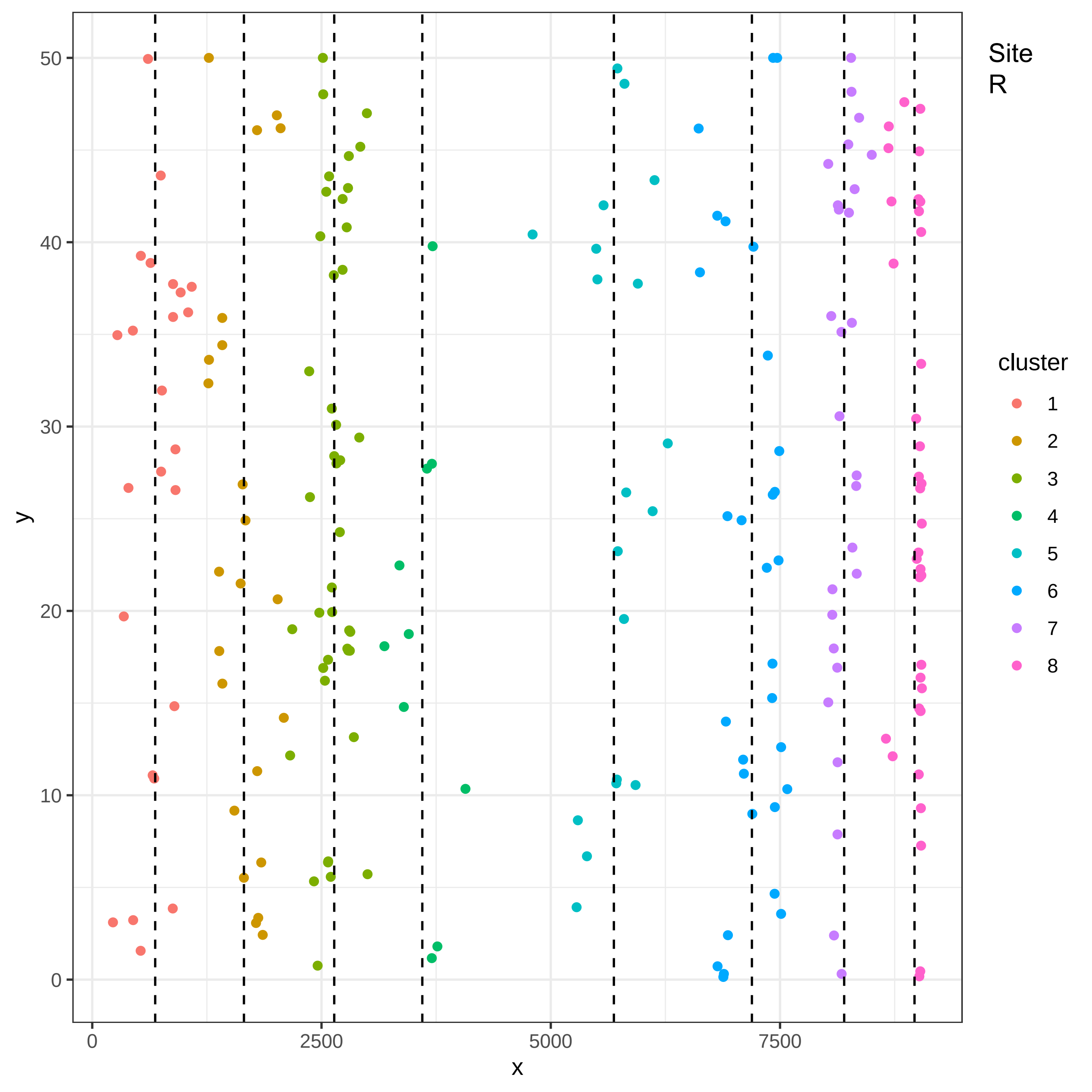
Site
R
cluster
1
2
3
4
5
6
7
8
y
x
0
10
20
30
40
50
0
2500
5000
7500

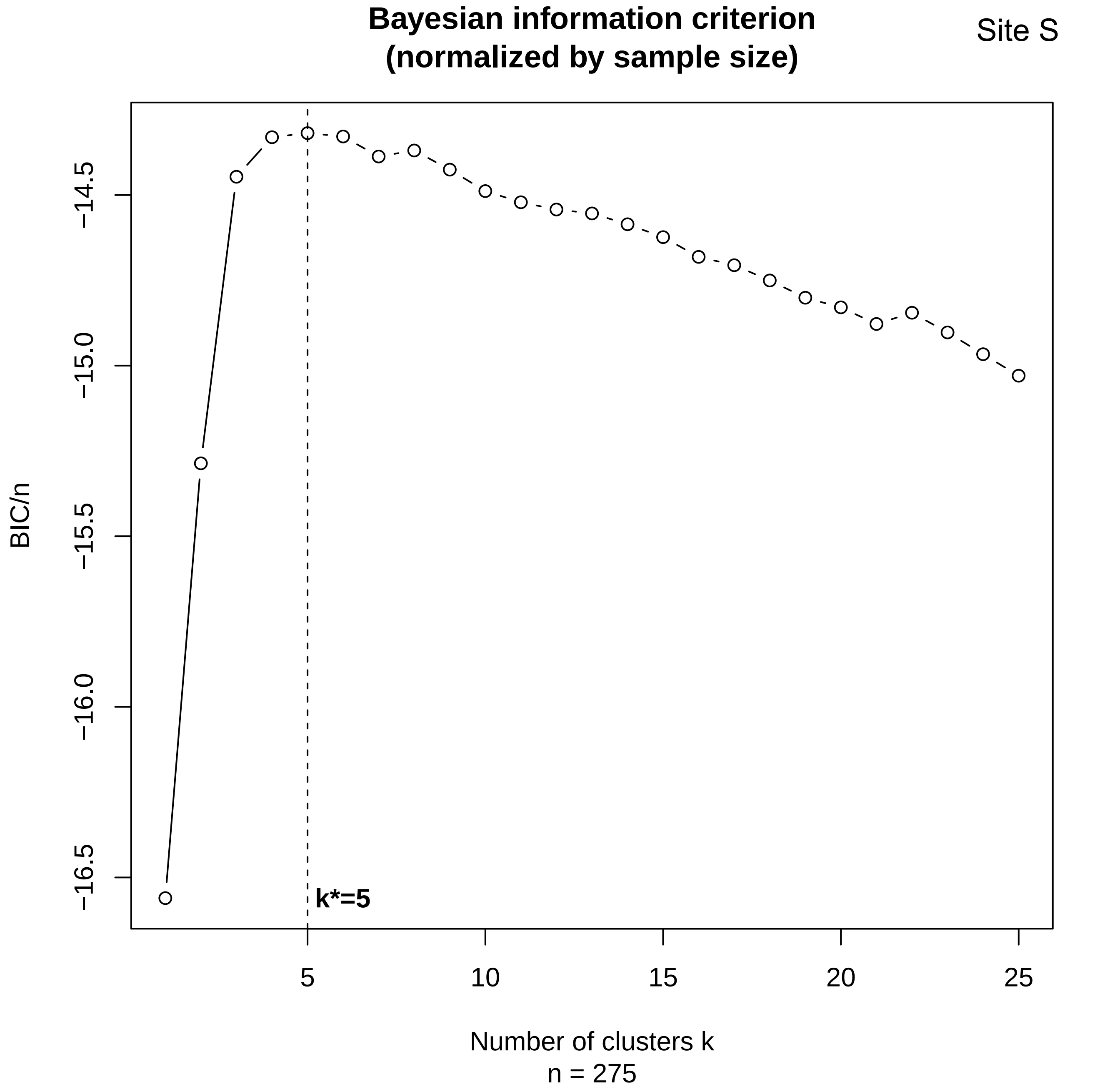

Bayesian information criterion
(normalized by sample size)
Site S
BIC/n
−14.5
−15.0
−15.5
−16.0
−16.5
k*=5
5
10
15
20
25
Number of clusters k
n = 275

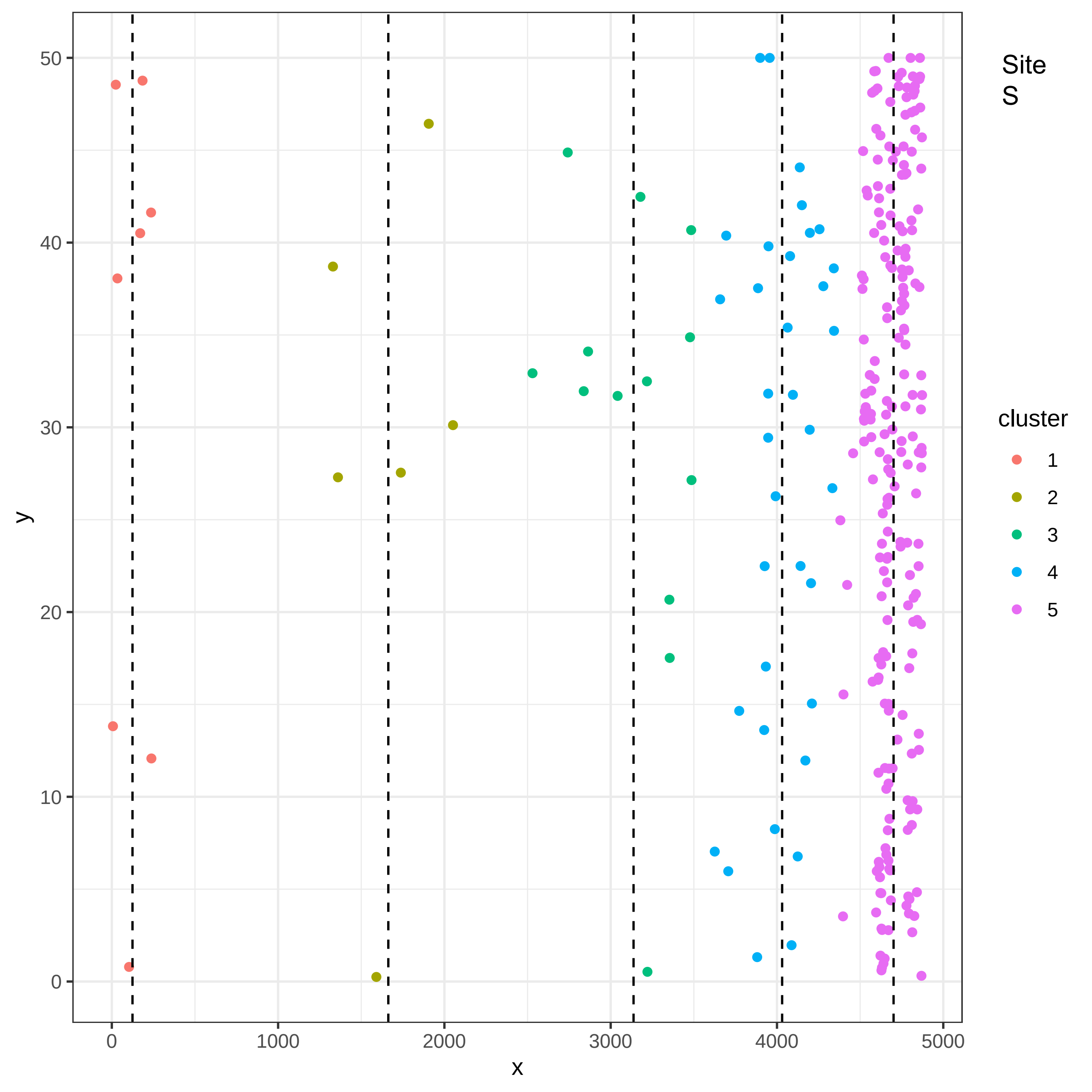
Site
S
cluster
1
2
3
4
5
y
x
0
1000
2000
3000
4000
5000
0
10
20
30
40
50

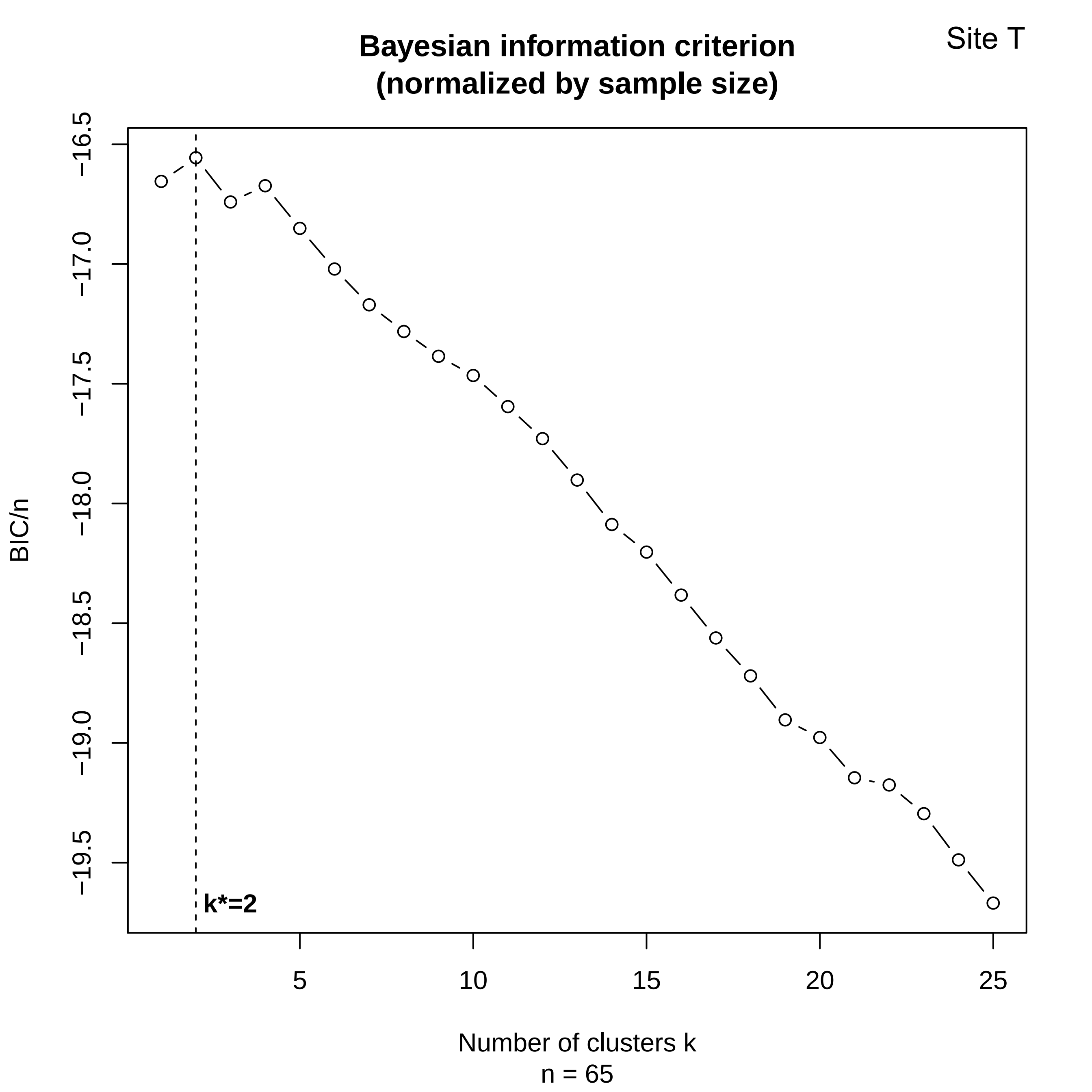
Site T
Bayesian information criterion
(normalized by sample size)
BIC/n
−16.5
−17.0
−17.5
−18.0
−18.5
−19.0
−19.5
k*=2
5
10
15
20
25
Number of clusters k
n = 65

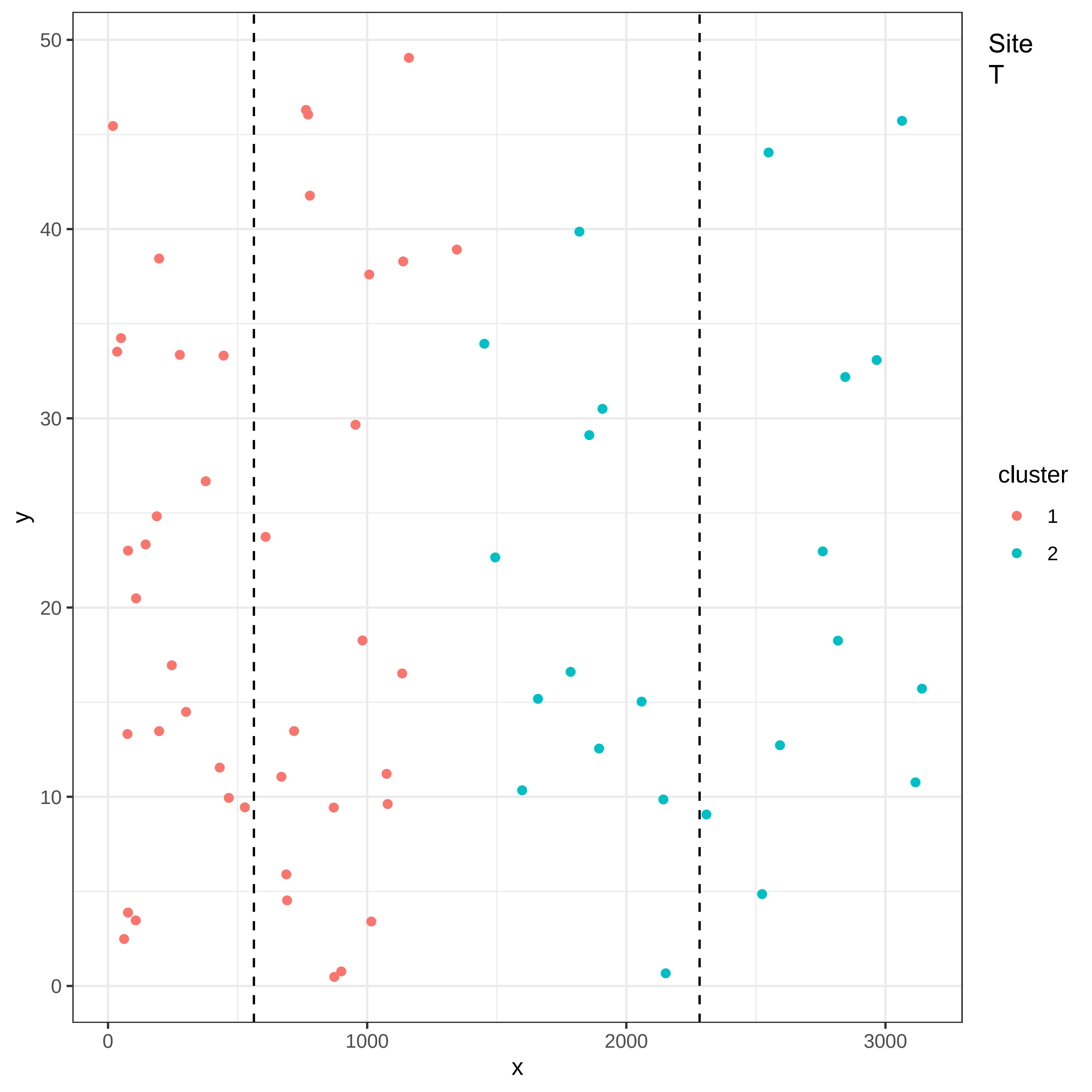

Site
T
cluster
1
2
y
x
0
10
20
30
40
50
0
1000
2000
3000

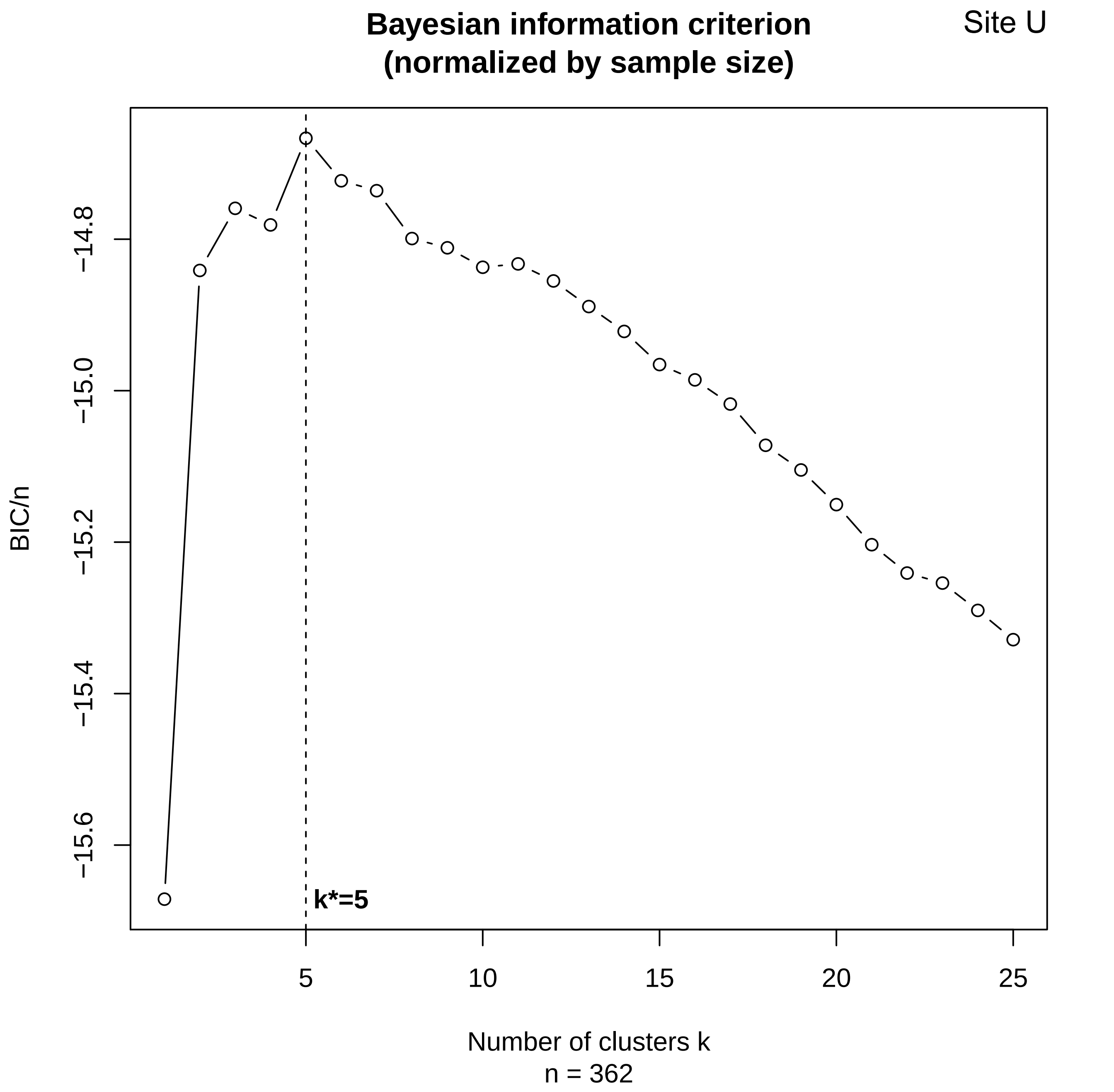
Bayesian information criterion
(normalized by sample size)
Site U
BIC/n
−14.8
−15.0
−15.2
−15.4
−15.6
k*=5
5
10
15
20
25
Number of clusters k
n = 362

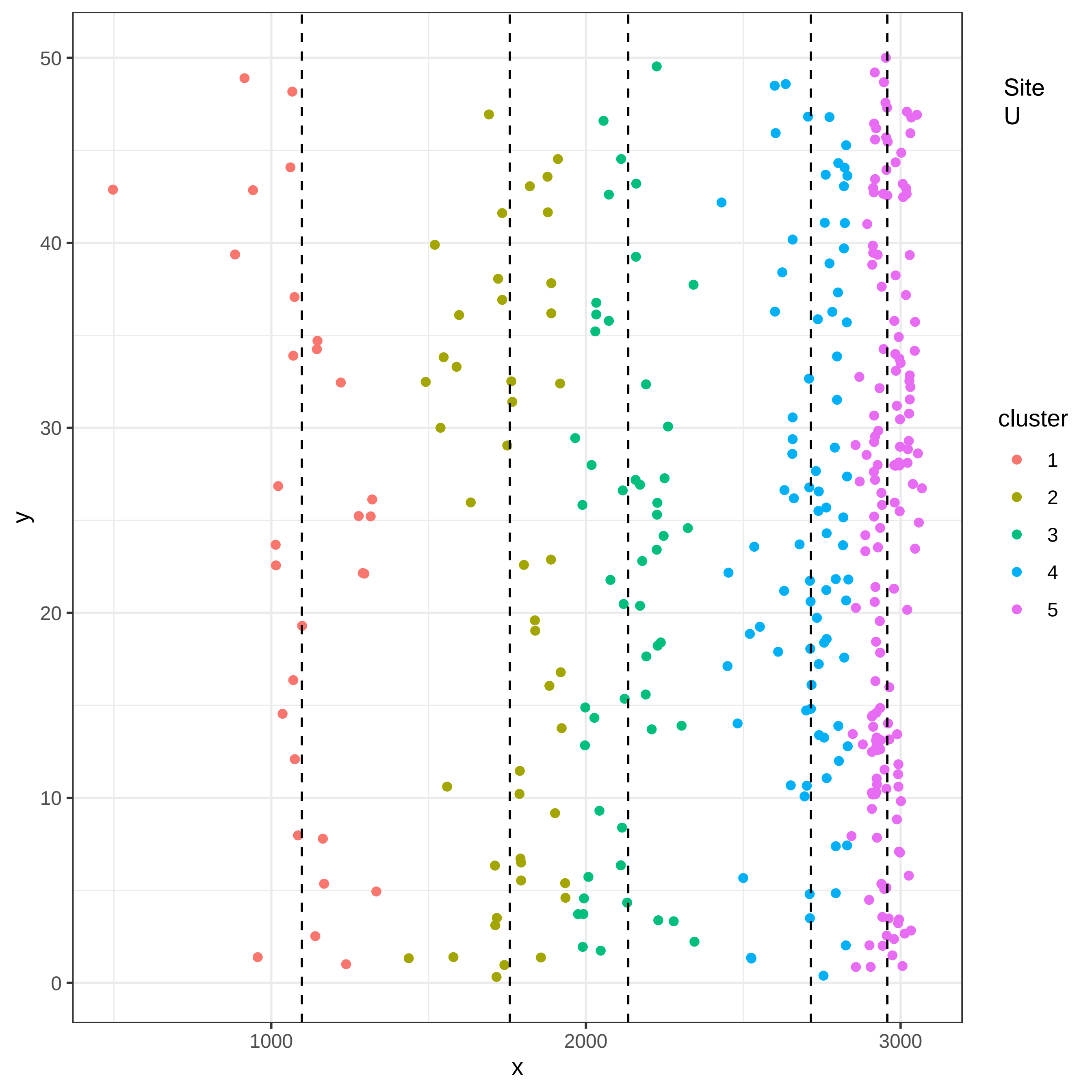

50
40
30
20
10
0
y
1000
2000
3000
x
Site
U
cluster
1
2
3
4
5

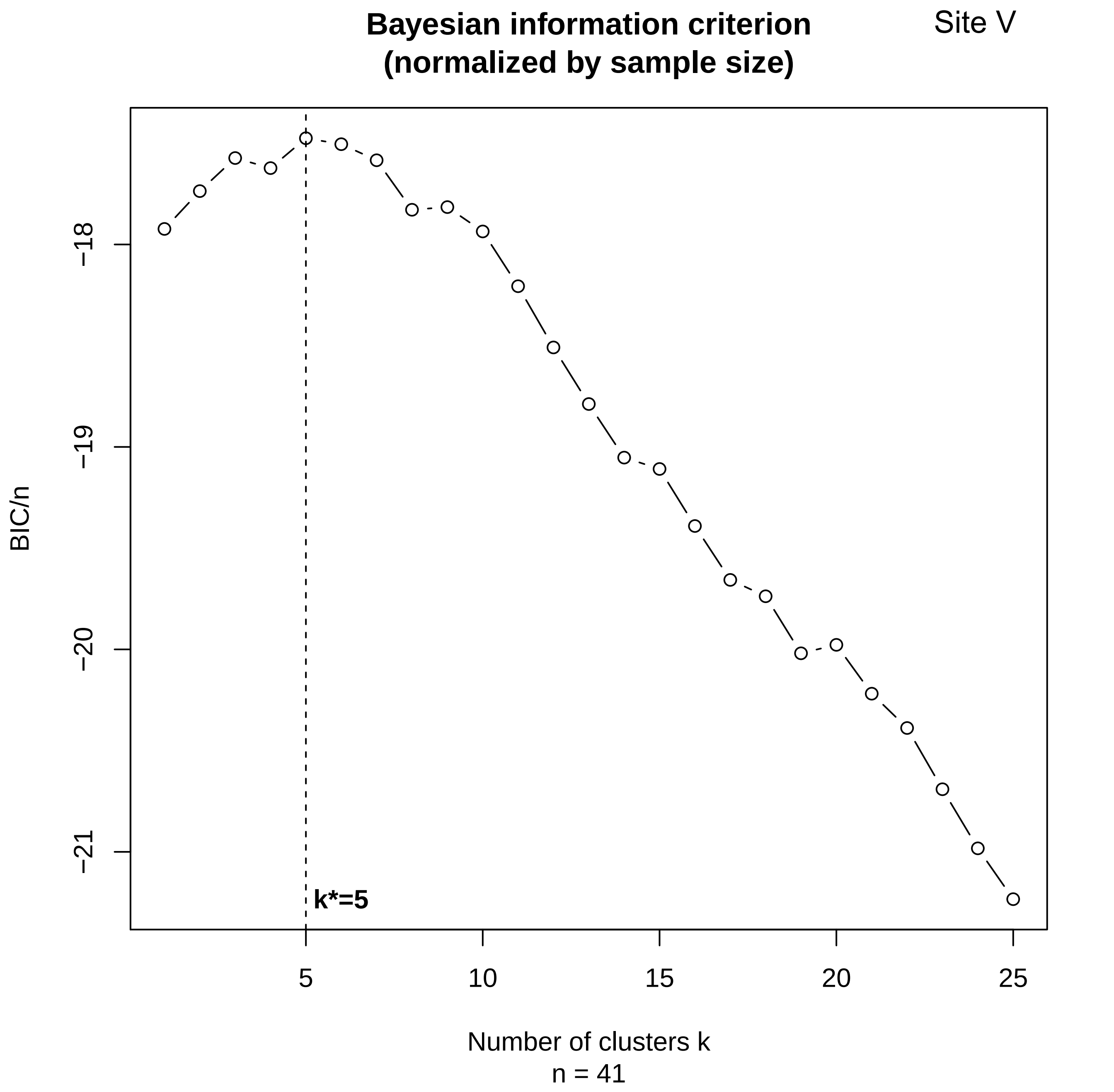

Bayesian information criterion
(normalized by sample size)
Site V
BIC/n
−18
−19
−20
−21
k*=5
5
10
15
20
25
Number of clusters k
n = 41

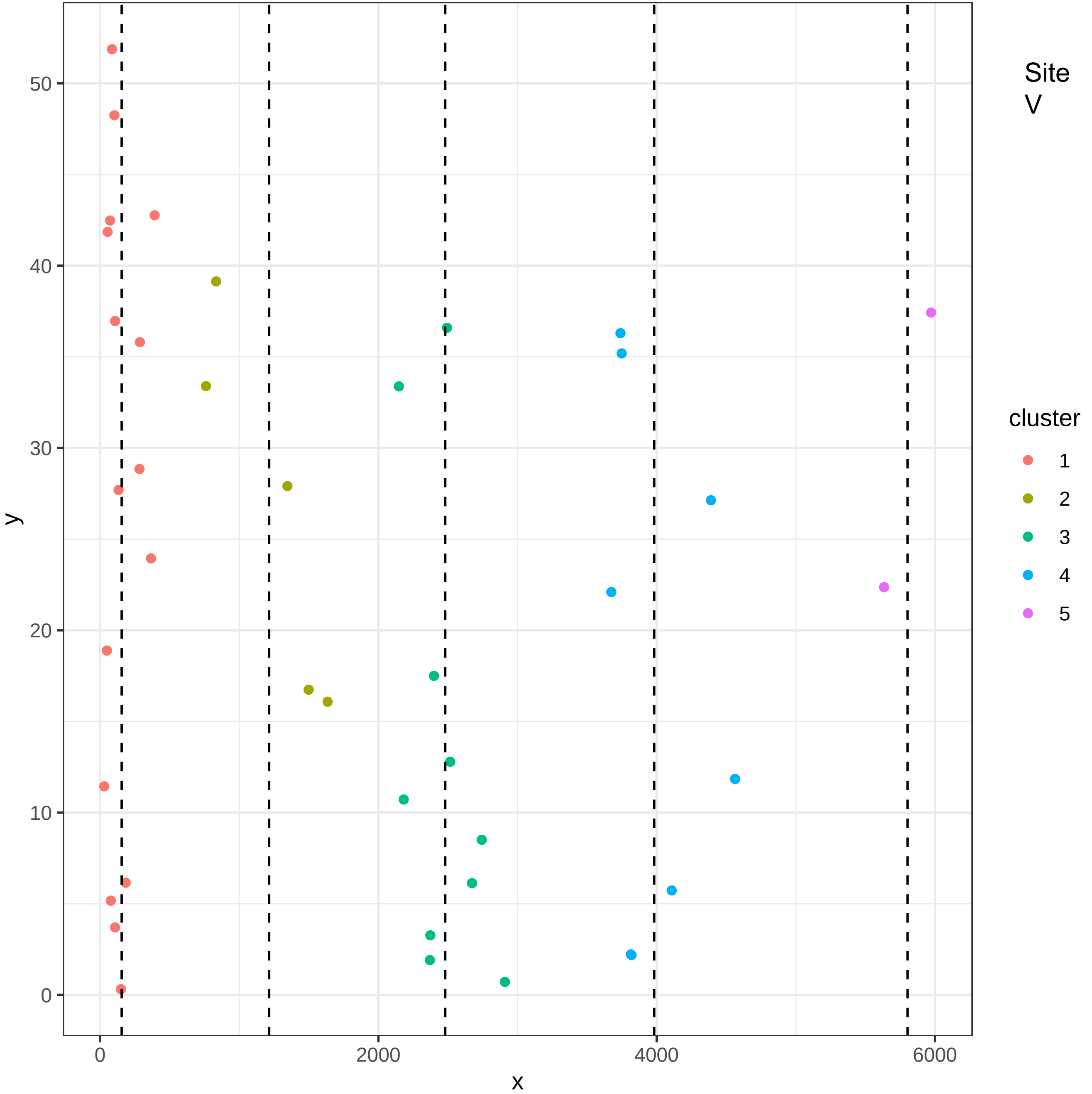
Site
V
cluster
1
2
3
4
5
y
x
0
10
20
30
40
50
0
2000
4000
6000

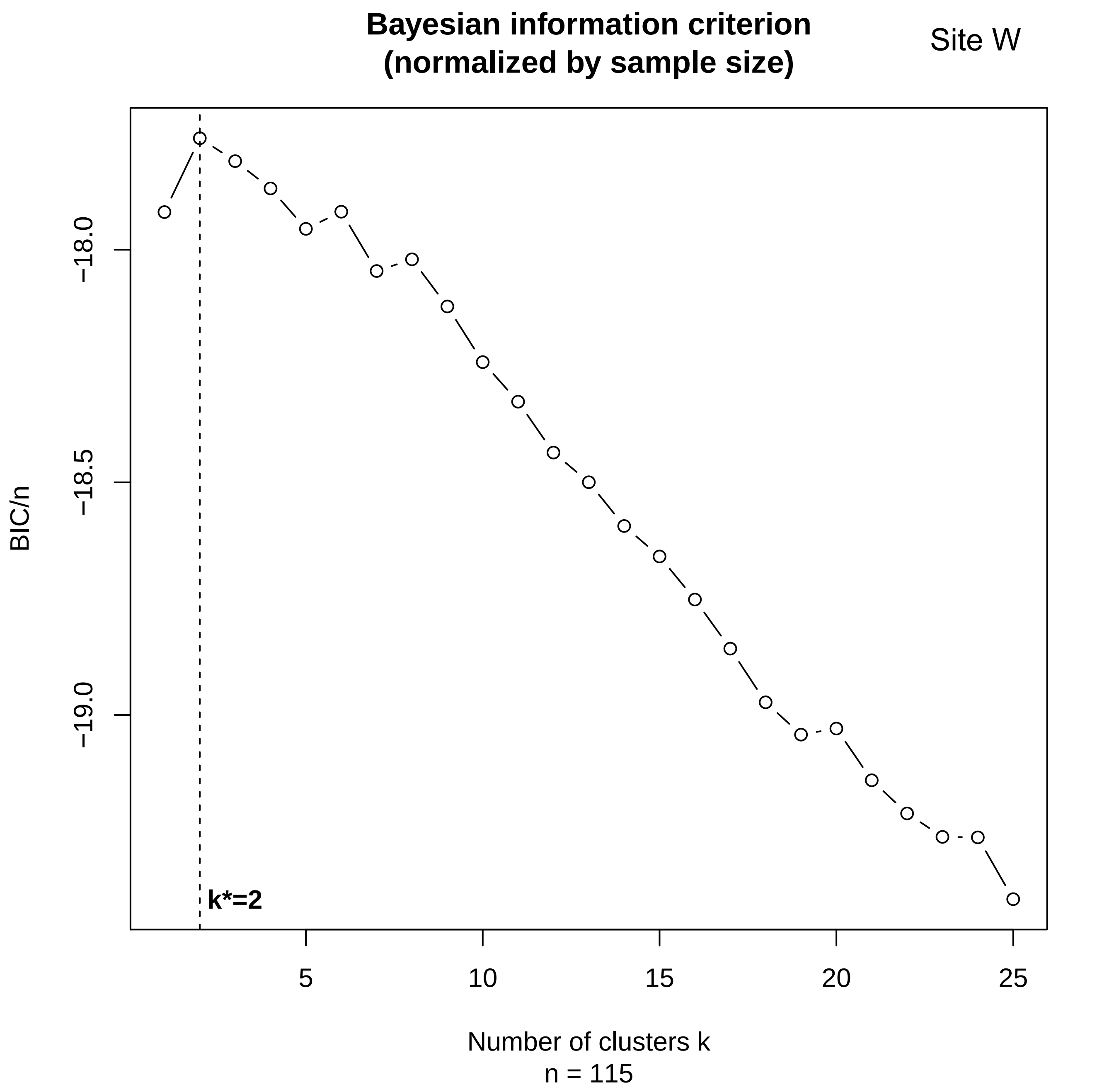

Bayesian information criterion
(normalized by sample size)
Site W
BIC/n
−18.0
−18.5
−19.0
k*=2
5
10
15
20
25
Number of clusters k
n = 115

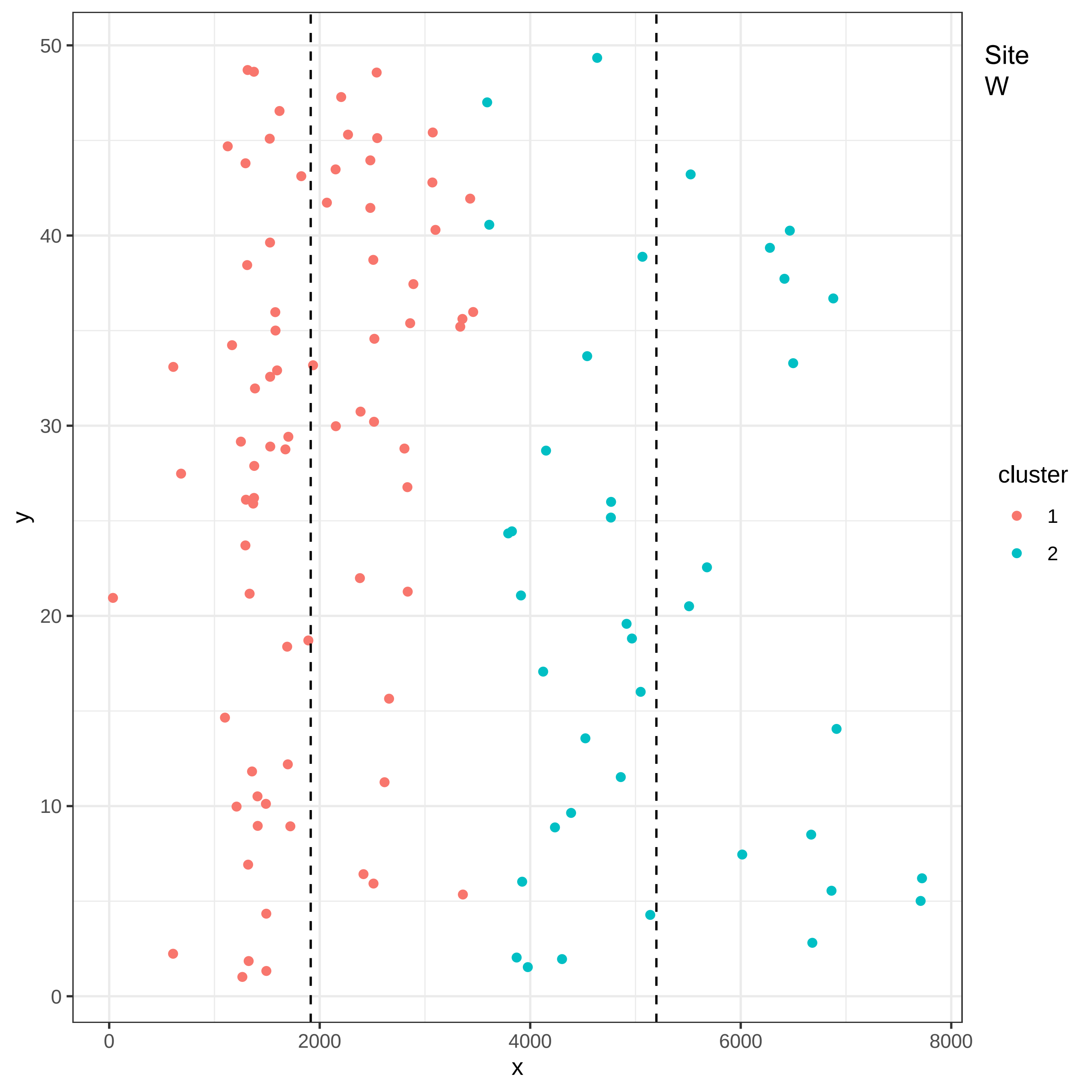

Site
W
cluster
1
2
y
x
0
10
20
30
40
50
0
2000
4000
6000
8000

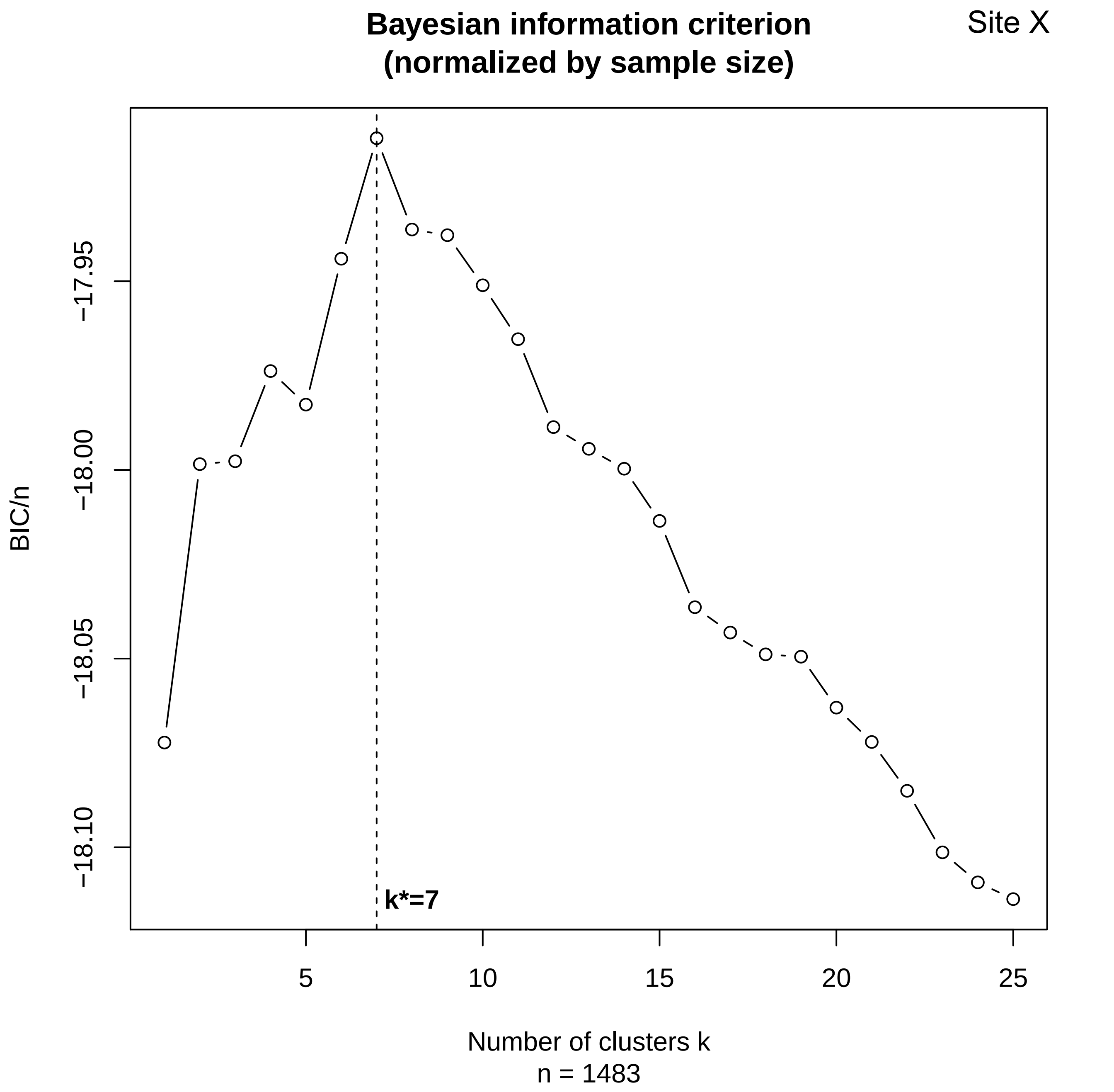

Bayesian information criterion
(normalized by sample size)
Site X
−17.95
−18.00
−18.05
−18.10
BIC/n
k*=7
5
10
15
20
25
Number of clusters k
n = 1483

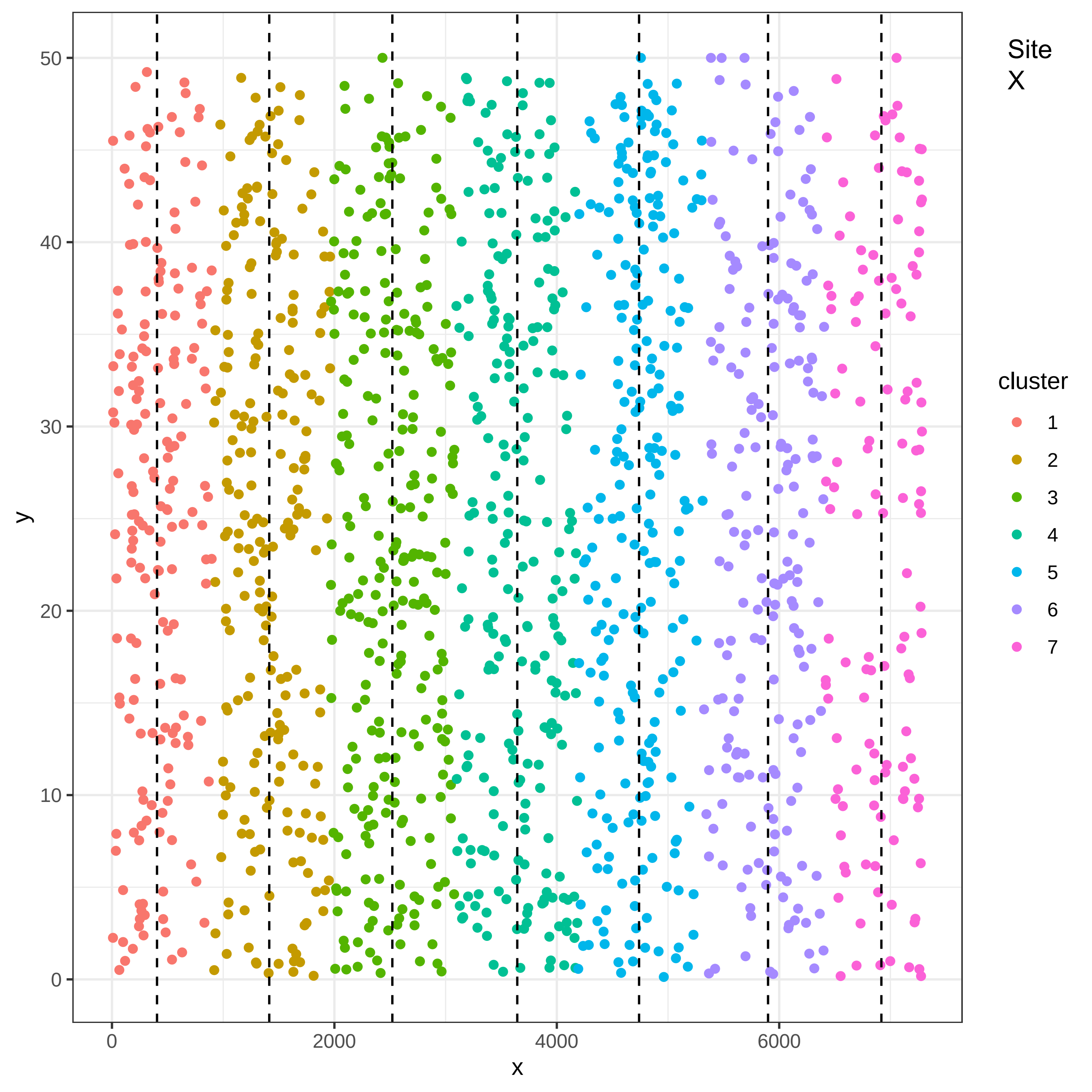
Site
X
cluster
1
2
3
4
5
6
7
y
x
0
10
20
30
40
50
0
2000
4000
6000

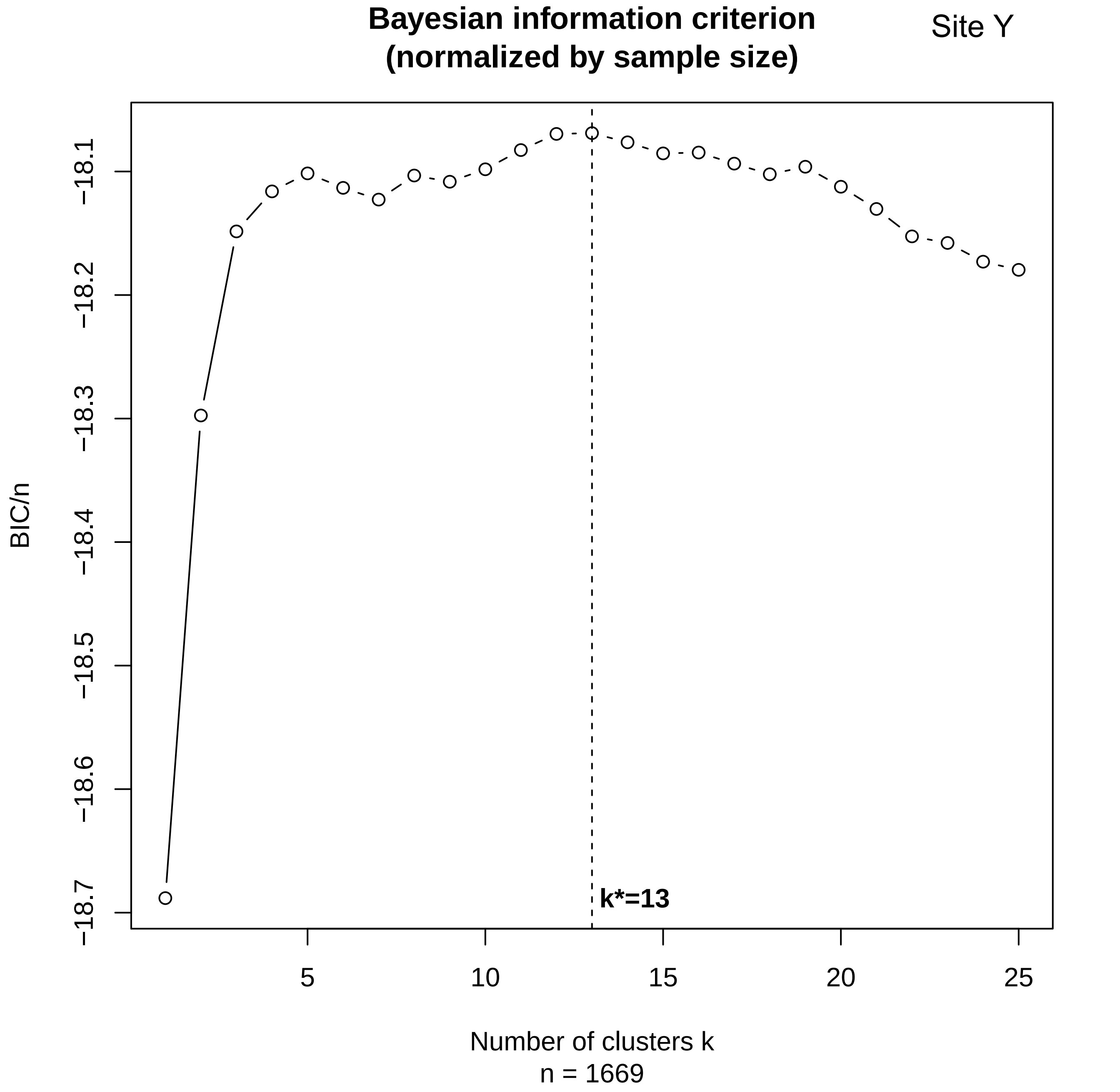
Bayesian information criterion
(normalized by sample size)
Site Y
BIC/n
−18.1
−18.2
−18.3
−18.4
−18.5
−18.6
−18.7
5
10
15
20
25
k*=13
Number of clusters k
n = 1669

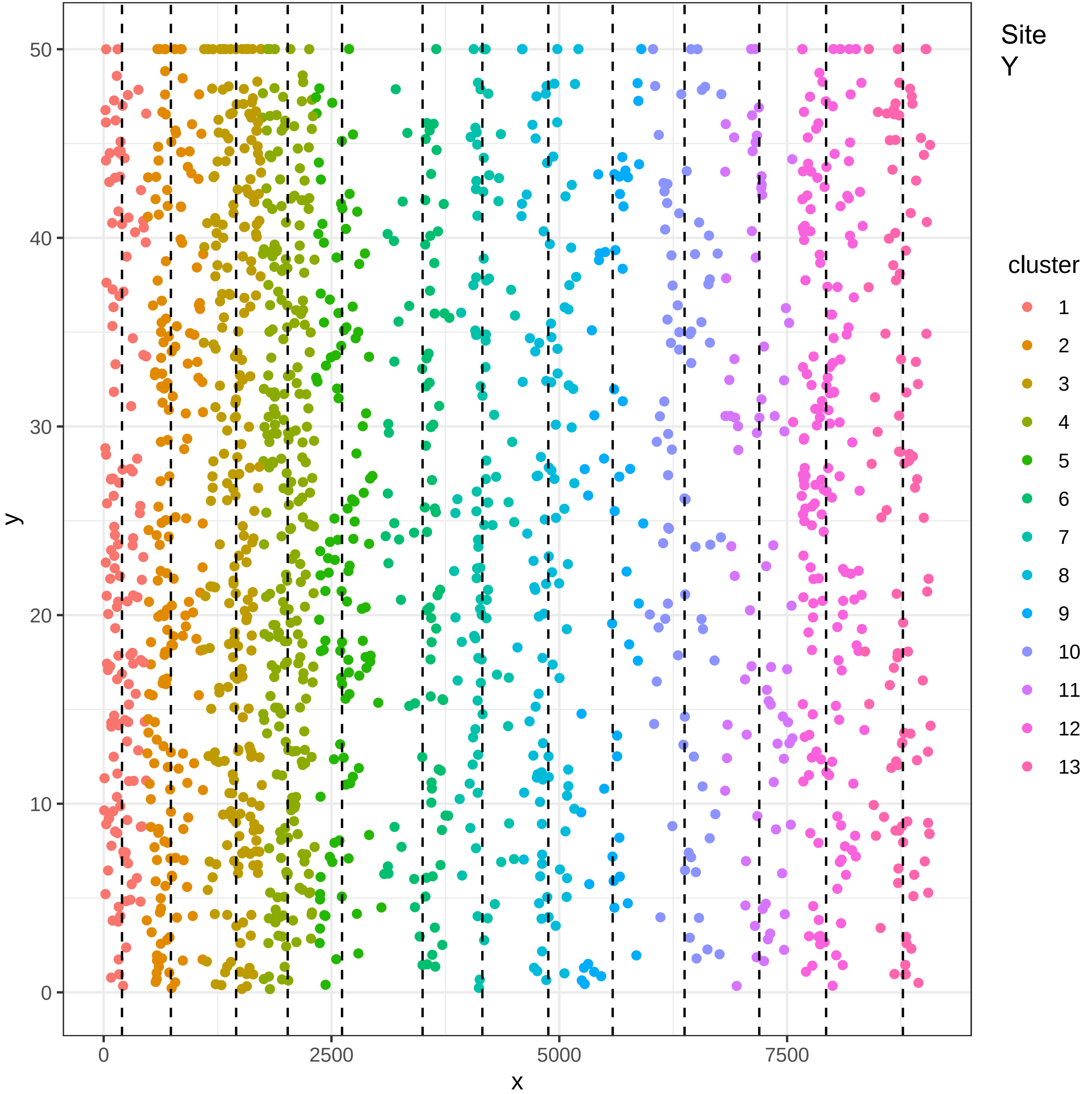

Site Y
cluster
1
2
3
4
5
6
7
8
9
10
11
12
13
y
x
0
10
20
30
40
50
0
2500
5000
7500

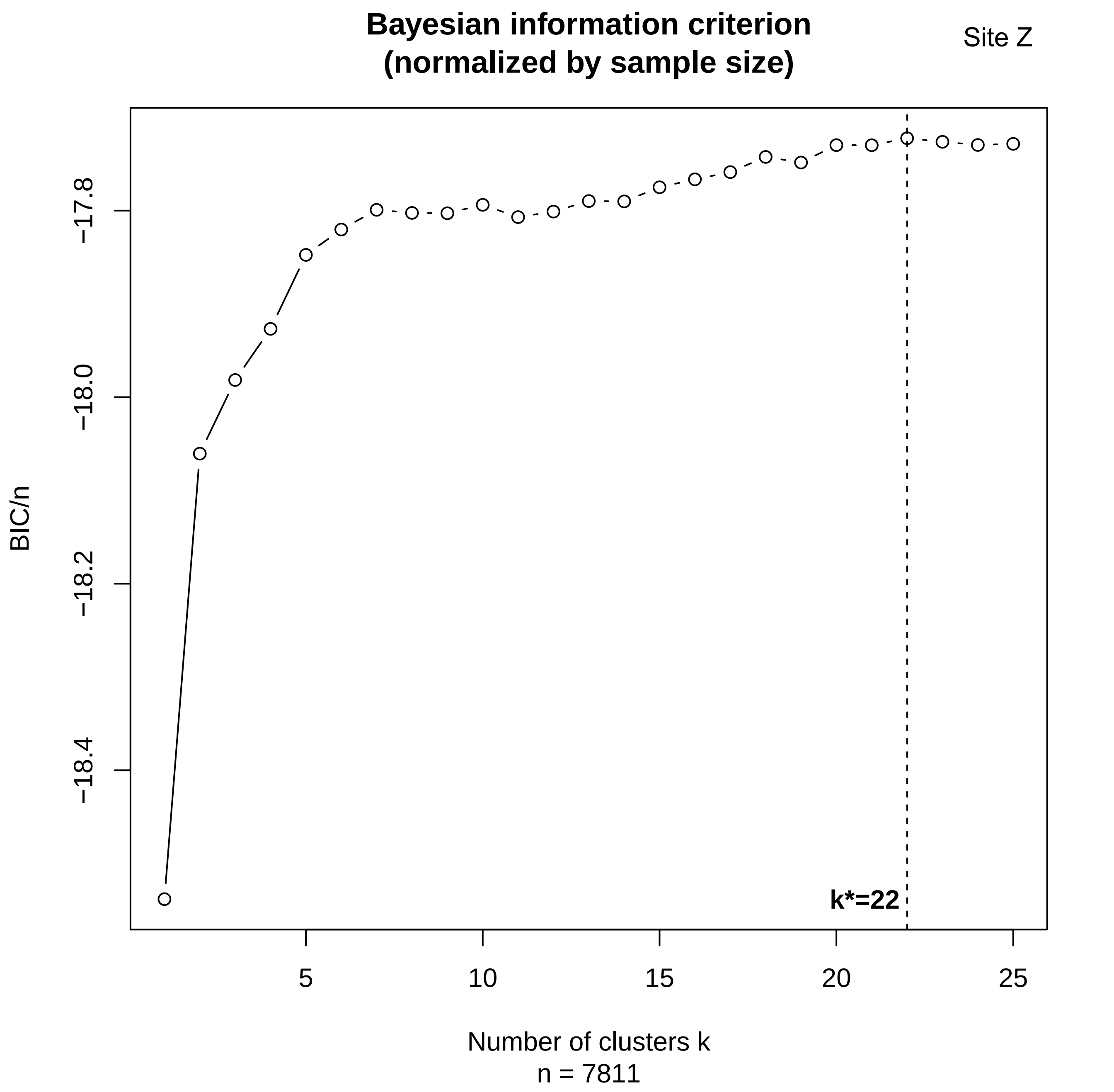
Bayesian information criterion
(normalized by sample size)
Site Z
BIC/n
−17.8
−18.0
−18.2
−18.4
k*=22
5
10
15
20
25
Number of clusters k
n = 7811

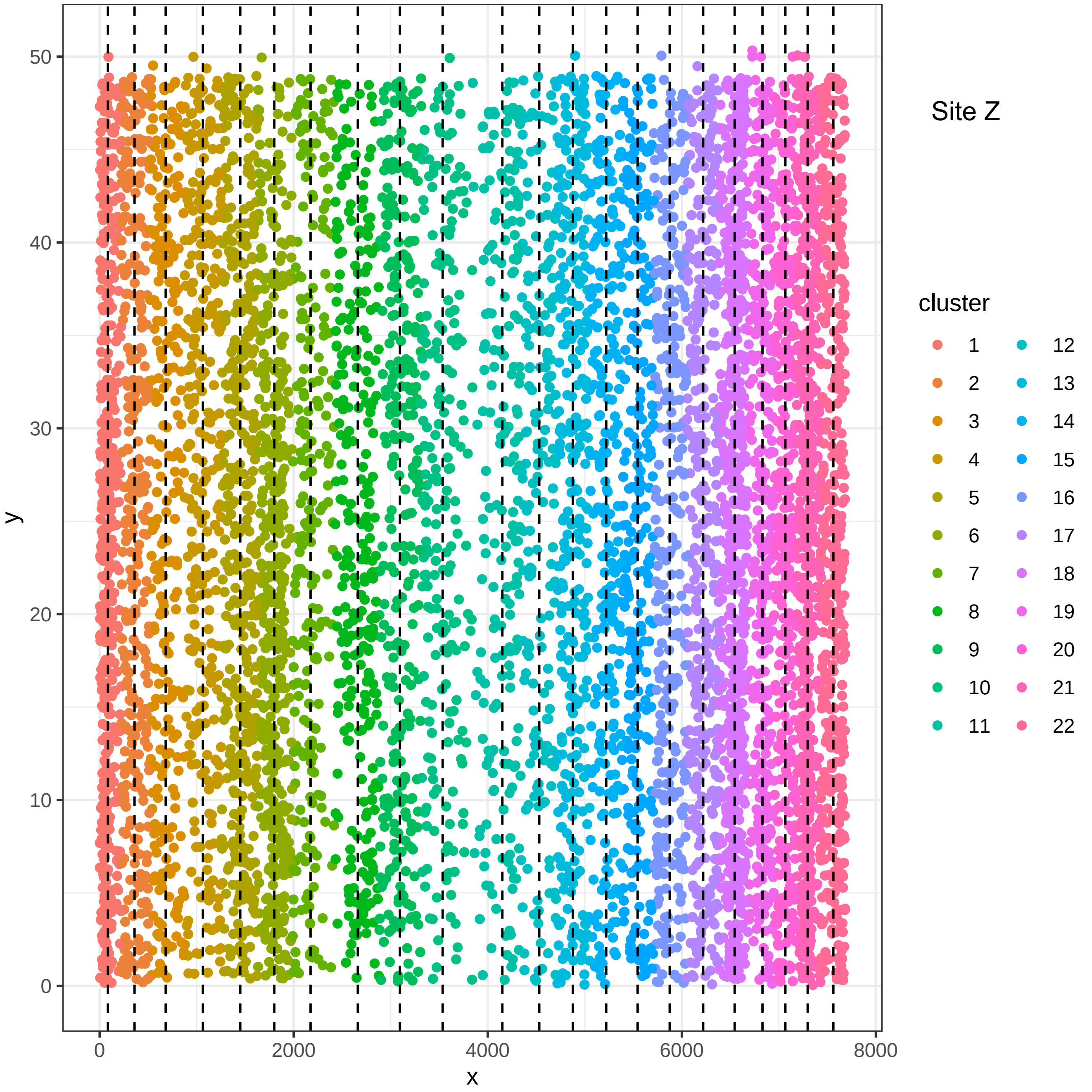

Site Z
cluster
1
2
3
4
5
6
7
8
9
10
11
12
13
14
15
16
17
18
19
20
21
22
y
x
0
10
20
30
40
50
0
2000
4000
6000
8000

Kernel density plots for all sites. Note, downslope is towards image bottom in all cases.

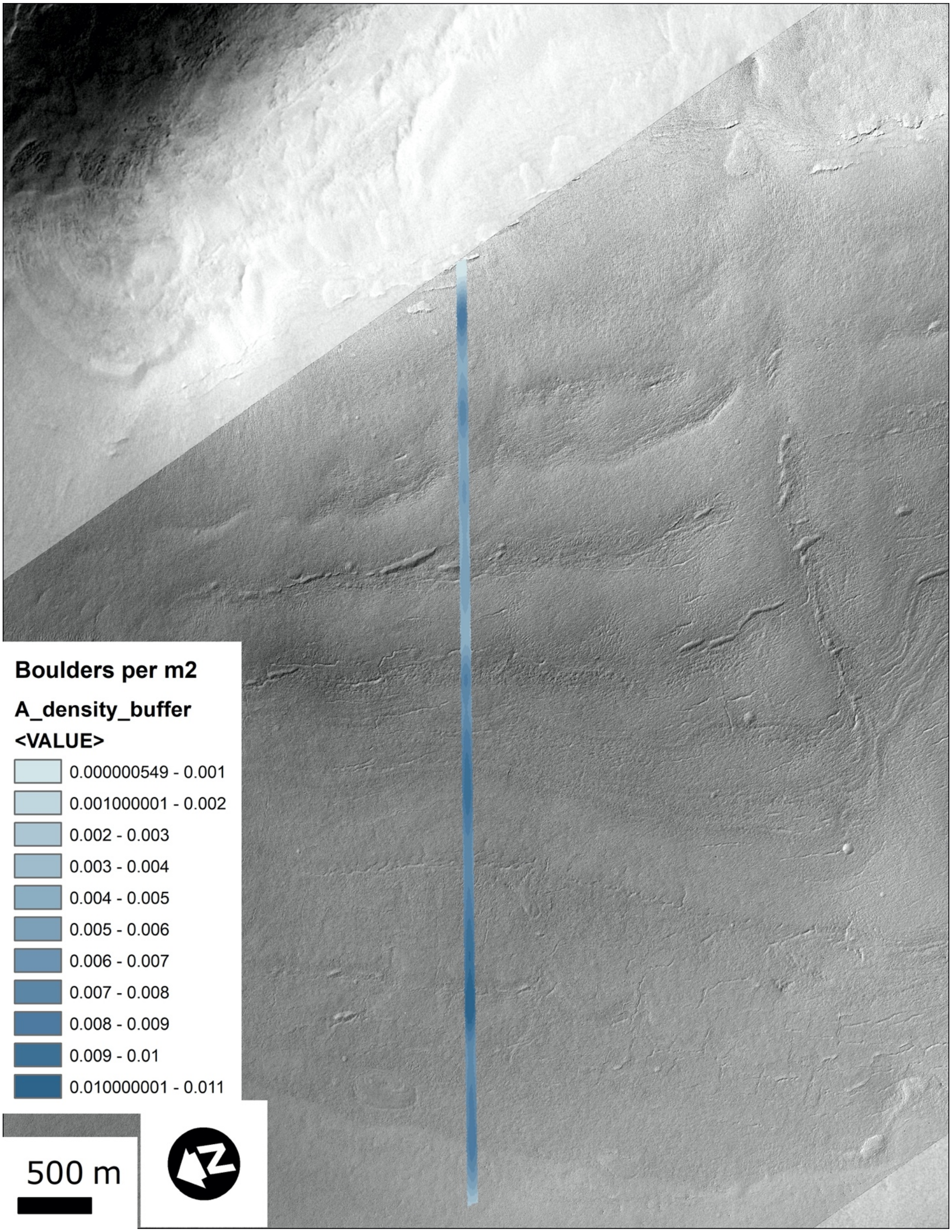
Boulders per m2
A_density_buffer
<VALUE>
0.000000549 - 0.001
0.001000001 - 0.002
0.002 - 0.003
0.003 - 0.004
0.004 - 0.005
0.005 - 0.006
0.006 - 0.007
0.007 - 0.008
0.008 - 0.009
0.009 - 0.01
0.010000001 - 0.011
500 m
N
Site A.

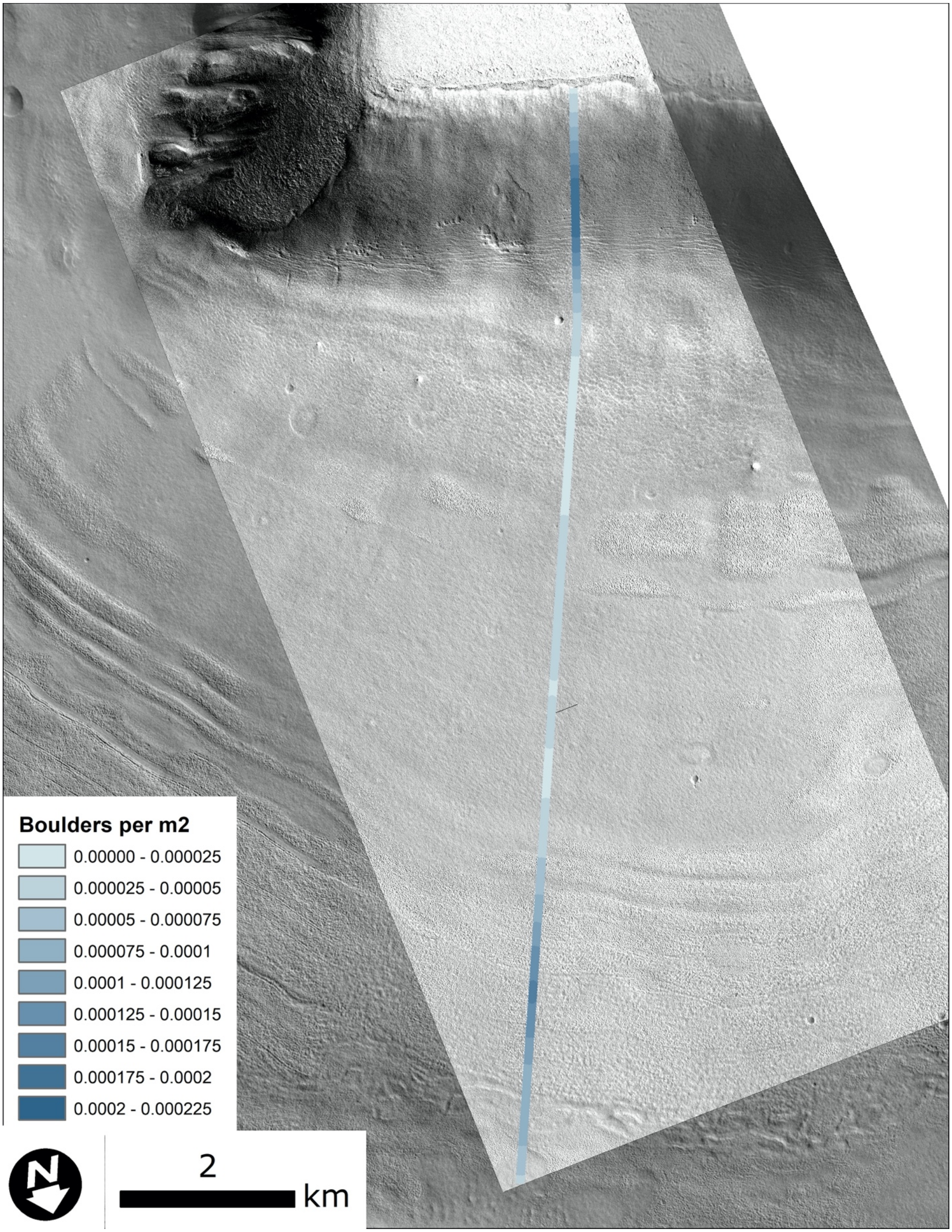
Boulders per m2
0.00000 - 0.000025
0.000025 - 0.00005
0.00005 - 0.000075
0.000075 - 0.0001
0.0001 - 0.000125
0.000125 - 0.00015
0.00015 - 0.000175
0.000175 - 0.0002
0.0002 - 0.000225
2
km
Site AA.

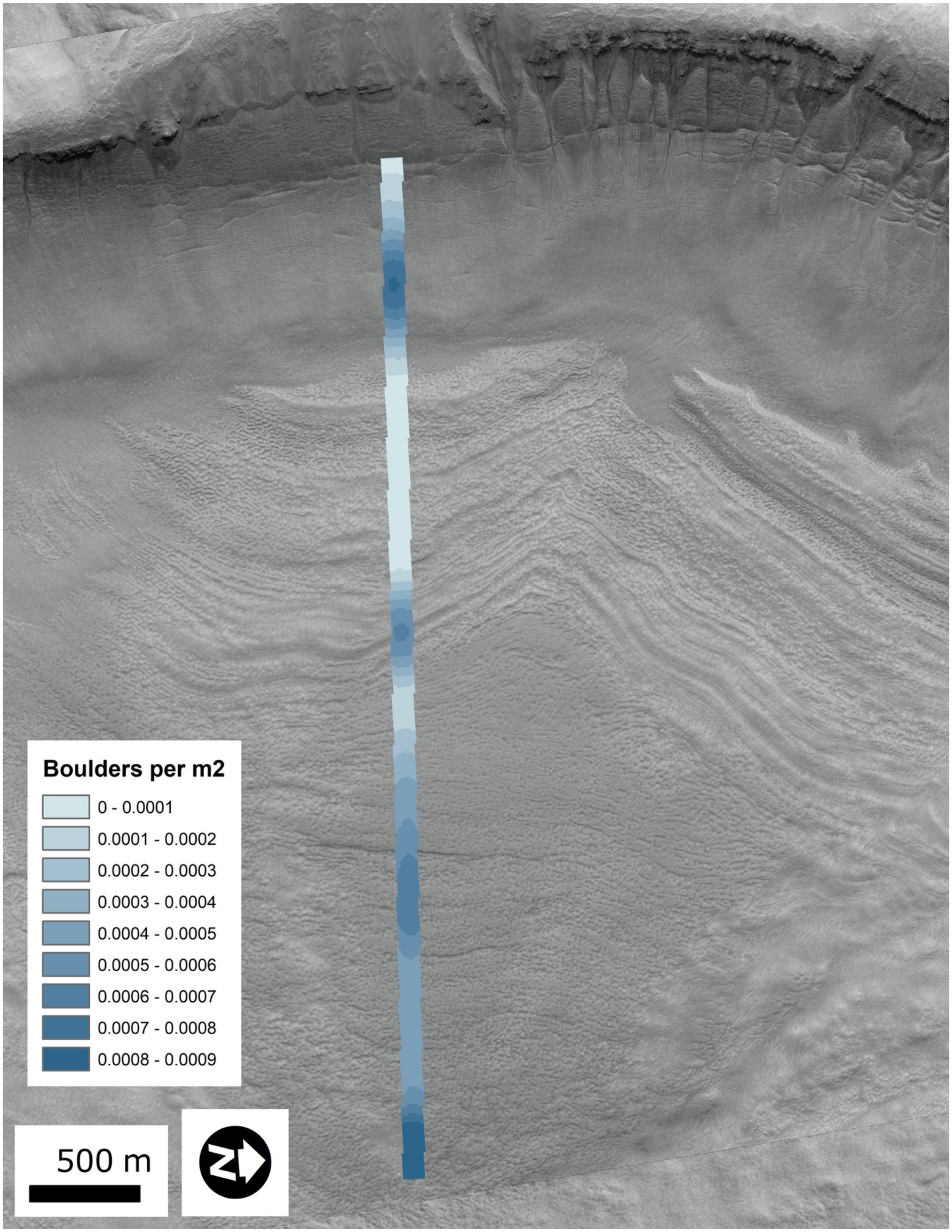
Boulders per m2
0 - 0.0001
0.0001 - 0.0002
0.0002 - 0.0003
0.0003 - 0.0004
0.0004 - 0.0005
0.0005 - 0.0006
0.0006 - 0.0007
0.0007 - 0.0008
0.0008 - 0.0009
500 m
N
Site B.

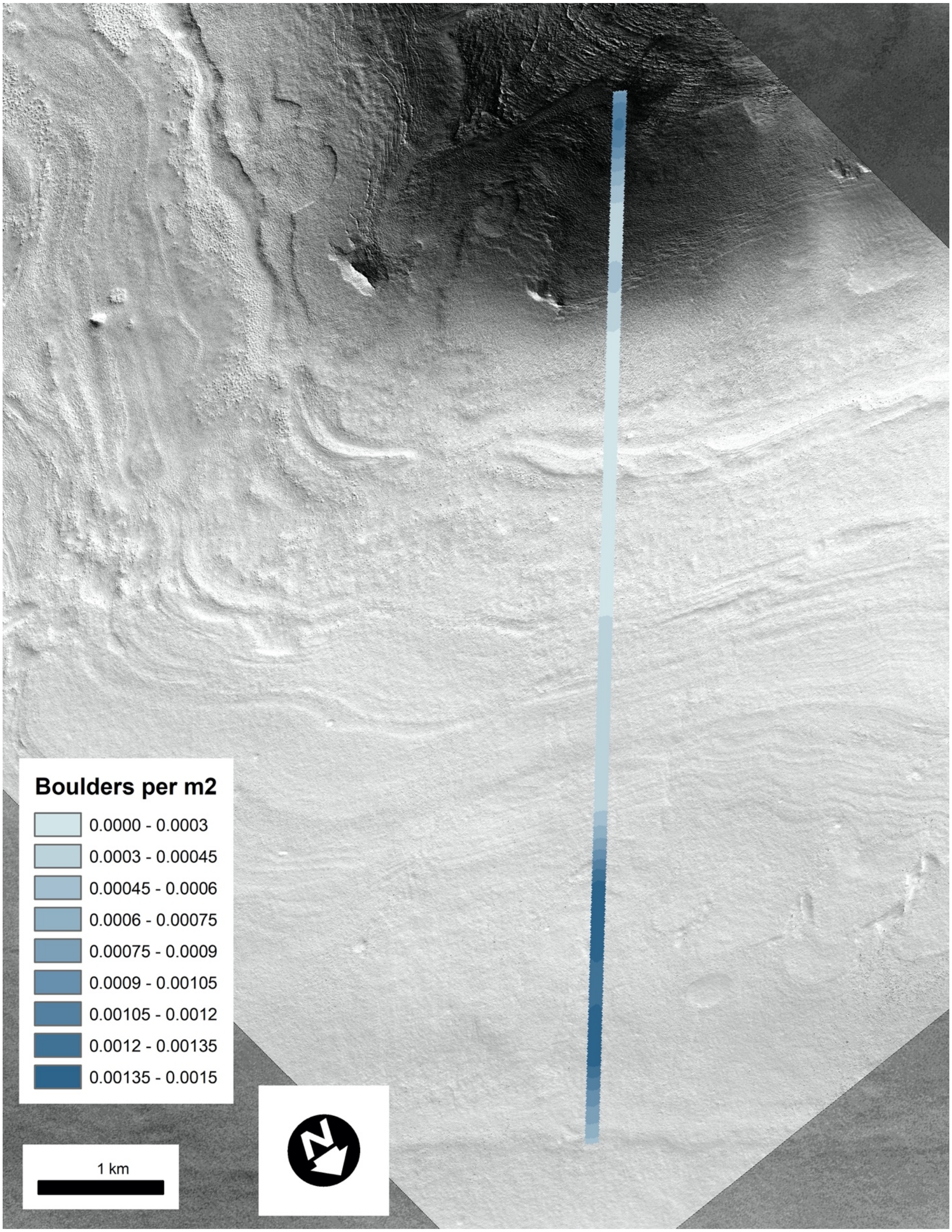
Boulders per m2
0.0000 - 0.0003
0.0003 - 0.00045
0.00045 - 0.0006
0.0006 - 0.00075
0.00075 - 0.0009
0.0009 - 0.00105
0.00105 - 0.0012
0.0012 - 0.00135
0.00135 - 0.0015
1 km
N
Site BB.

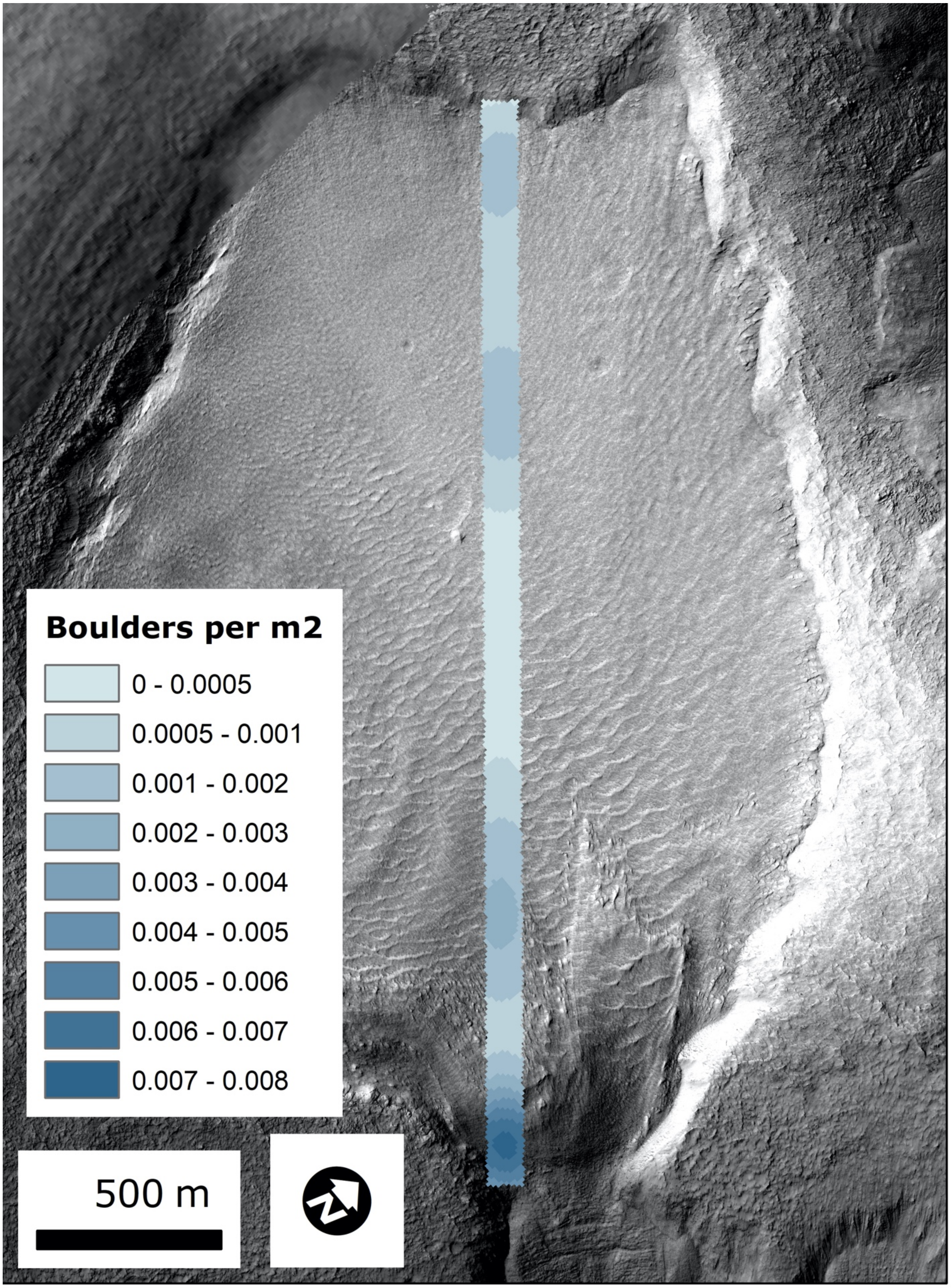
Boulders per m2
0 - 0.0005
0.0005 - 0.001
0.001 - 0.002
0.002 - 0.003
0.003 - 0.004
0.004 - 0.005
0.005 - 0.006
0.006 - 0.007
0.007 - 0.008
500 m
N

Site C.

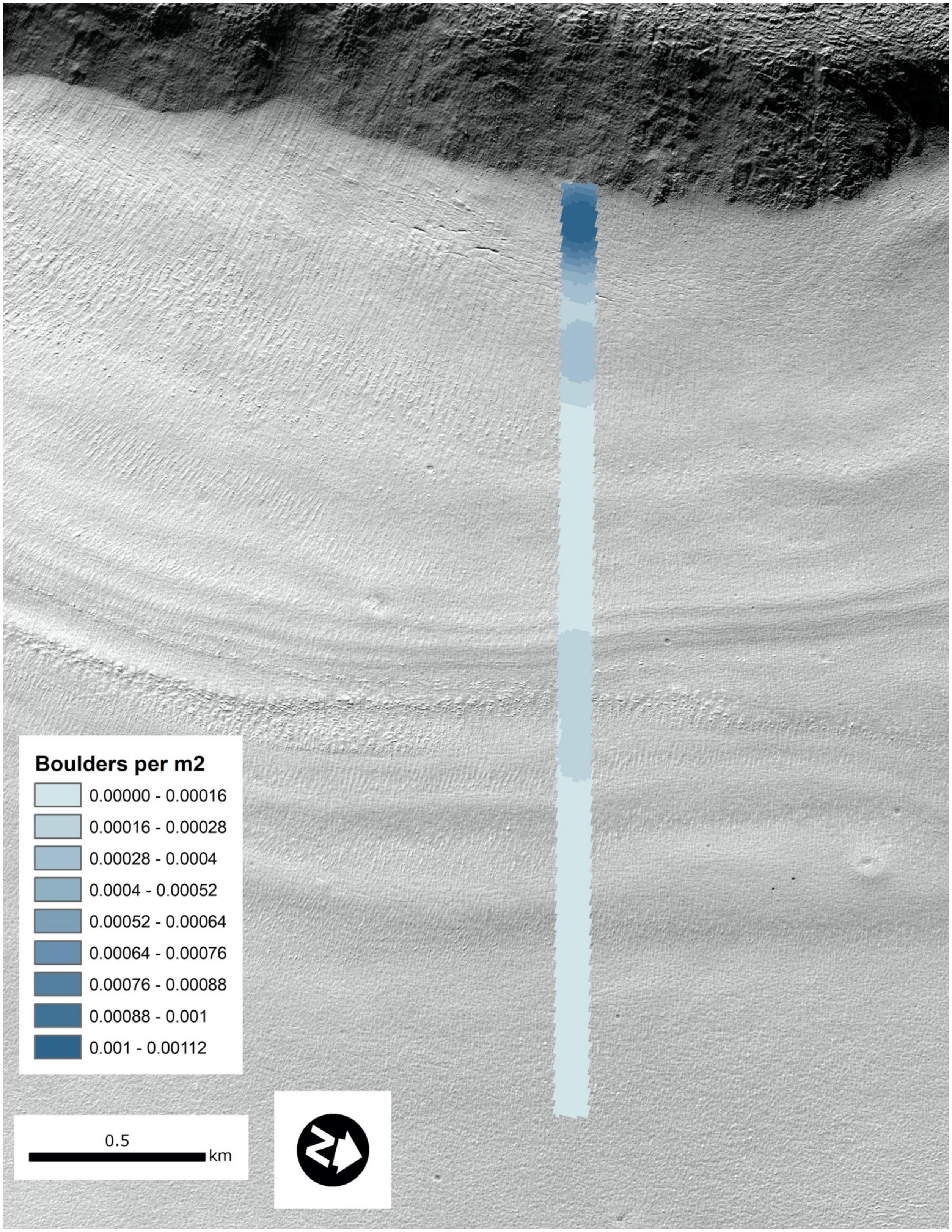

Boulders per m2
0.00000 - 0.00016
0.00016 - 0.00028
0.00028 - 0.0004
0.0004 - 0.00052
0.00052 - 0.00064
0.00064 - 0.00076
0.00076 - 0.00088
0.00088 - 0.001
0.001 - 0.00112
0.5
km
N

Site CC1.

Site CC1.

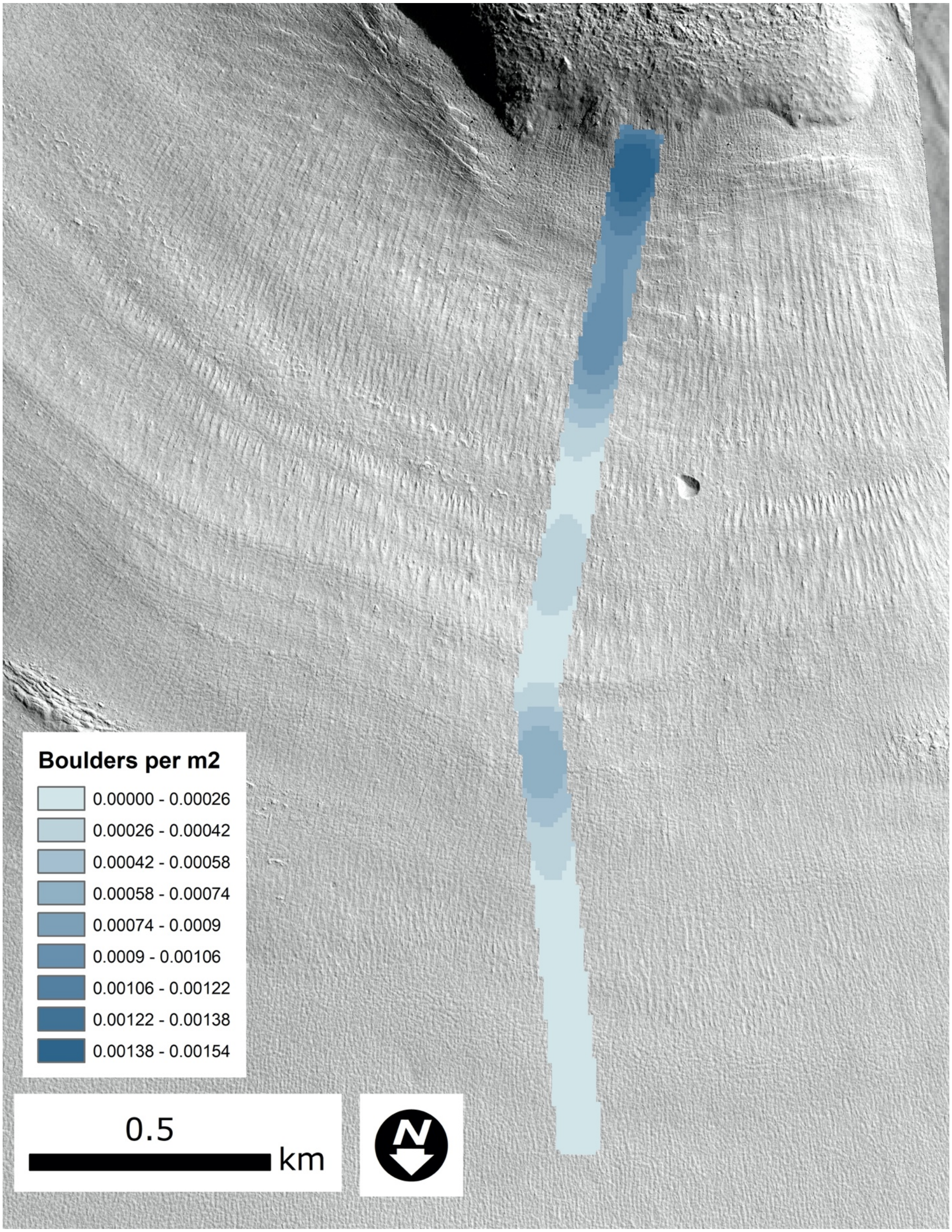

Site CC2.

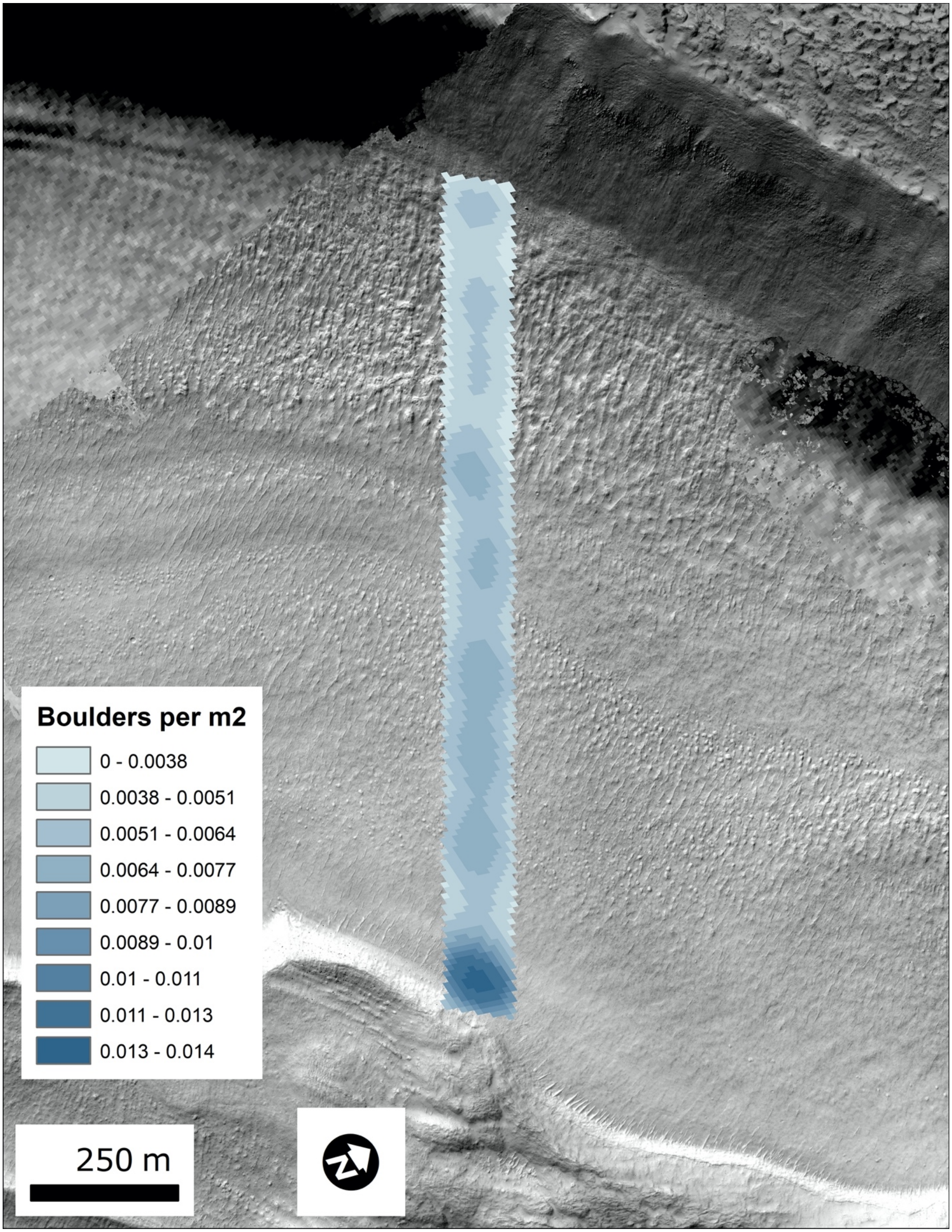

Boulders per m2
0 - 0.0038
0.0038 - 0.0051
0.0051 - 0.0064
0.0064 - 0.0077
0.0077 - 0.0089
0.0089 - 0.01
0.01 - 0.011
0.011 - 0.013
0.013 - 0.014
250 m

Site D1.

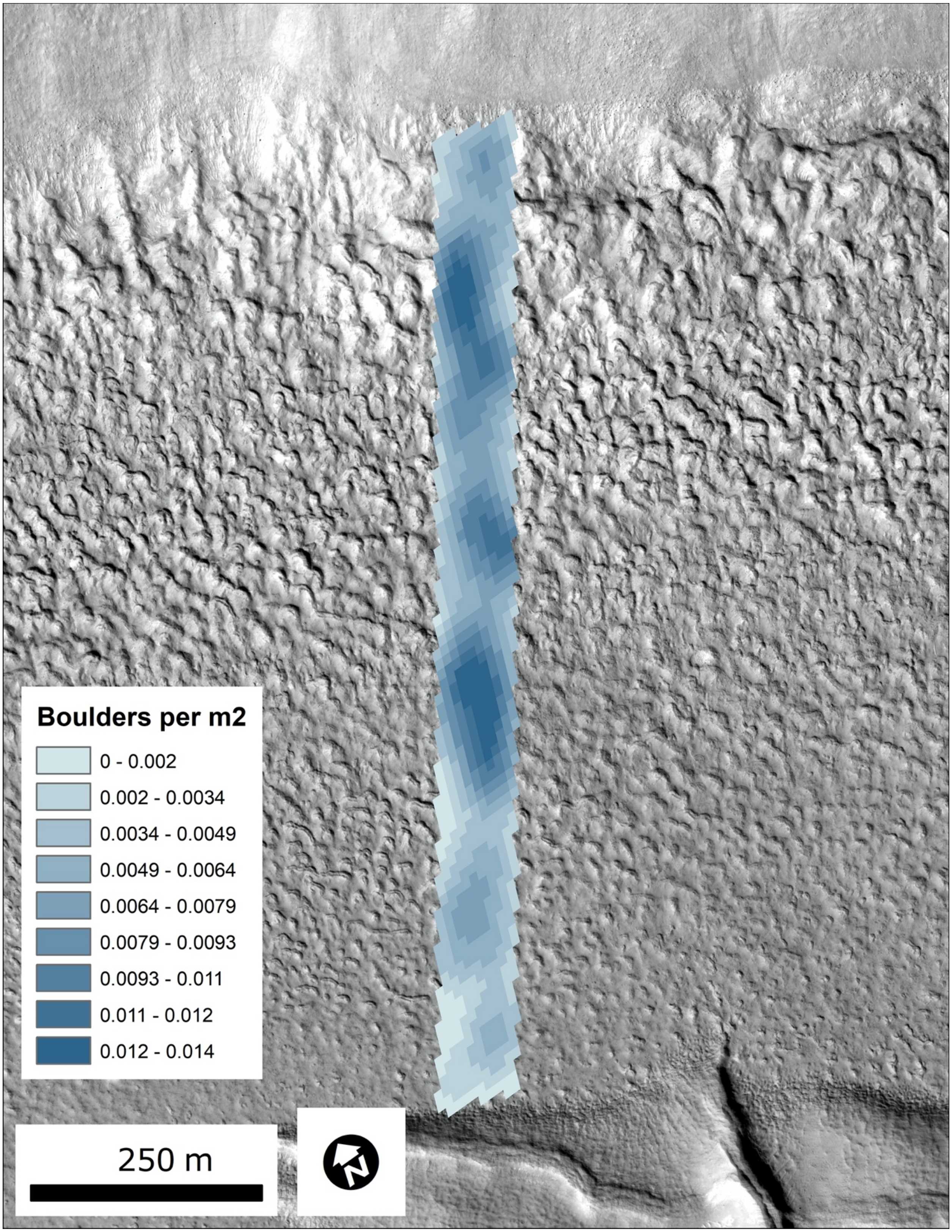
Boulders per m2
0 - 0.002
0.002 - 0.0034
0.0034 - 0.0049
0.0049 - 0.0064
0.0064 - 0.0079
0.0079 - 0.0093
0.0093 - 0.011
0.011 - 0.012
0.012 - 0.014
250 m
Site D2

Site D3

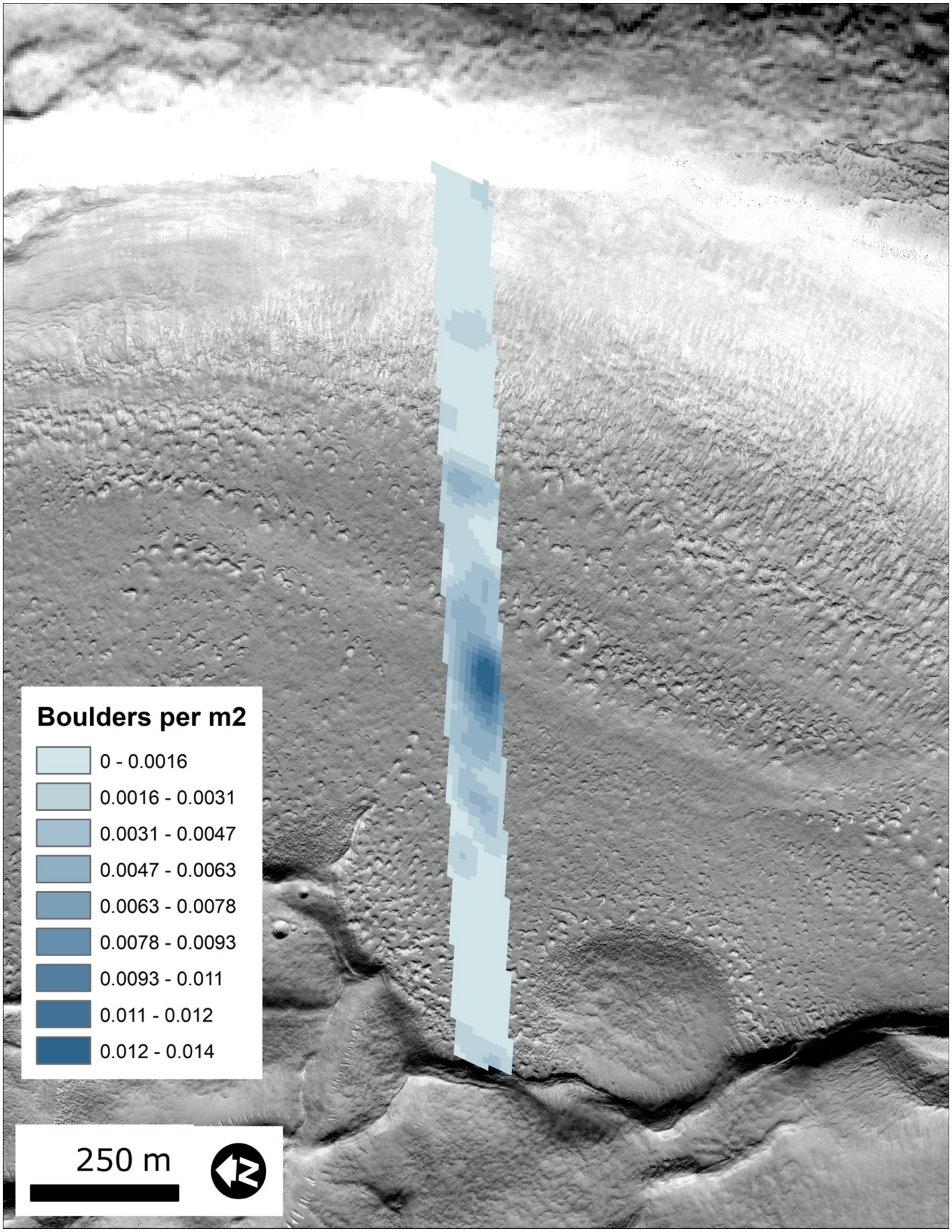

Site D3

Site D4.

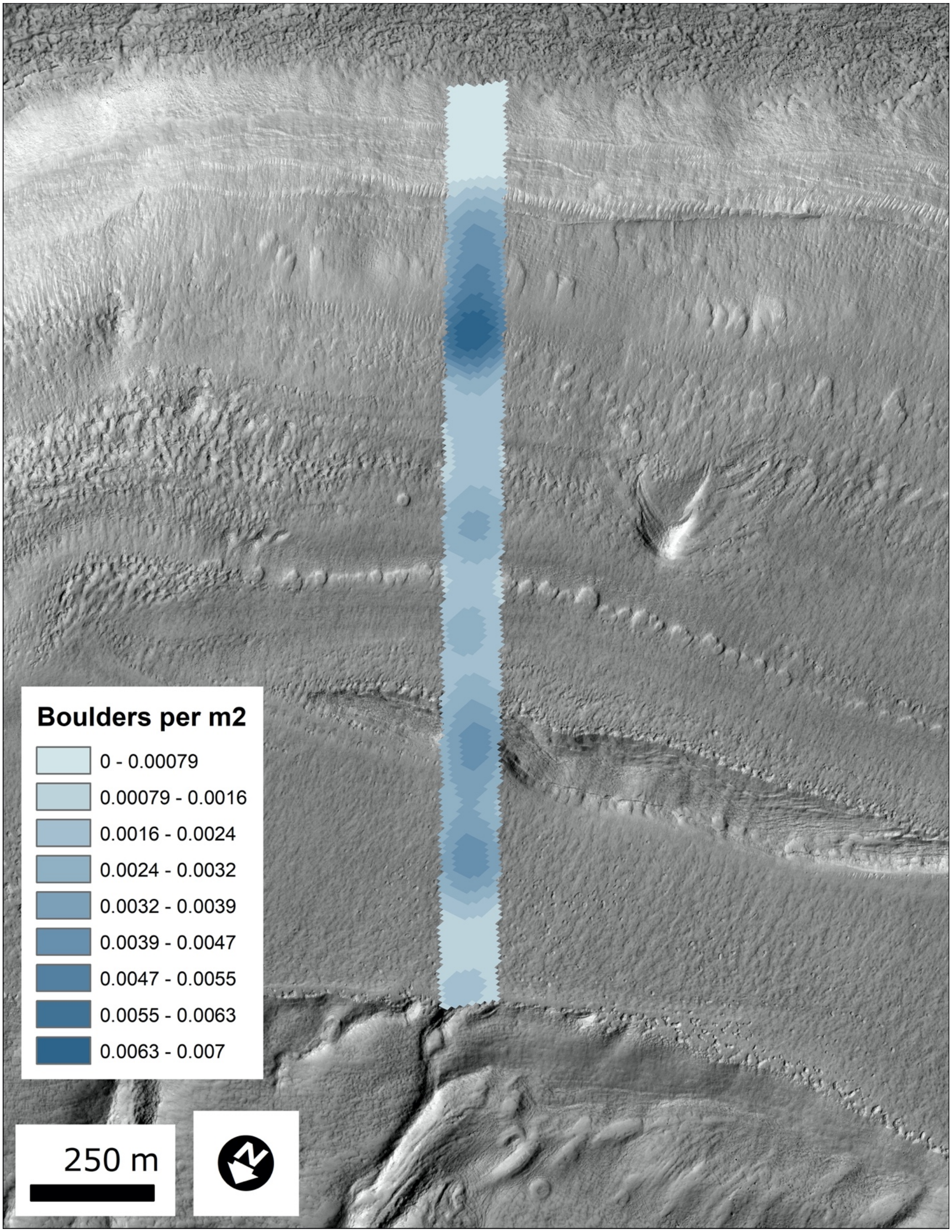

Site D4.

Site D5.

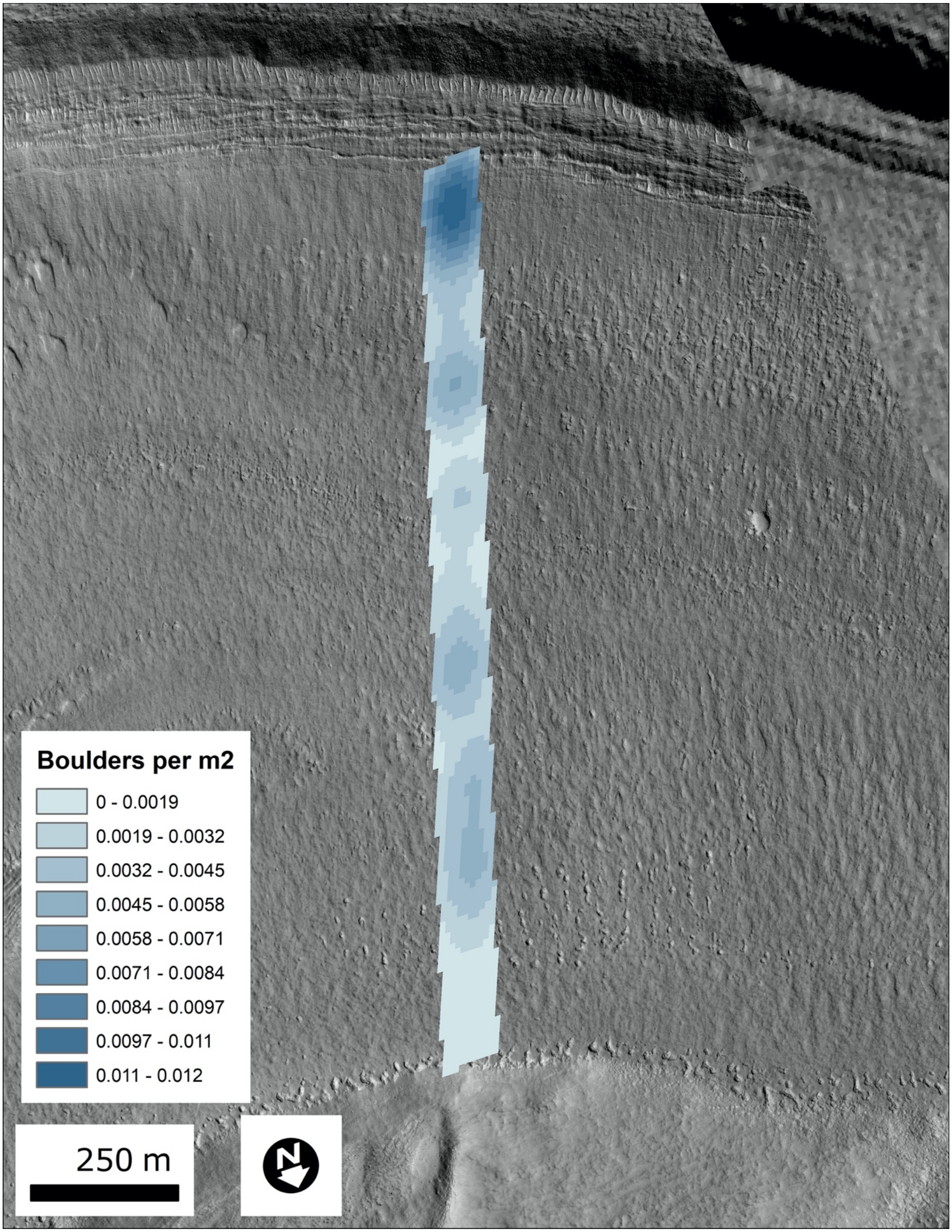

Site D5.

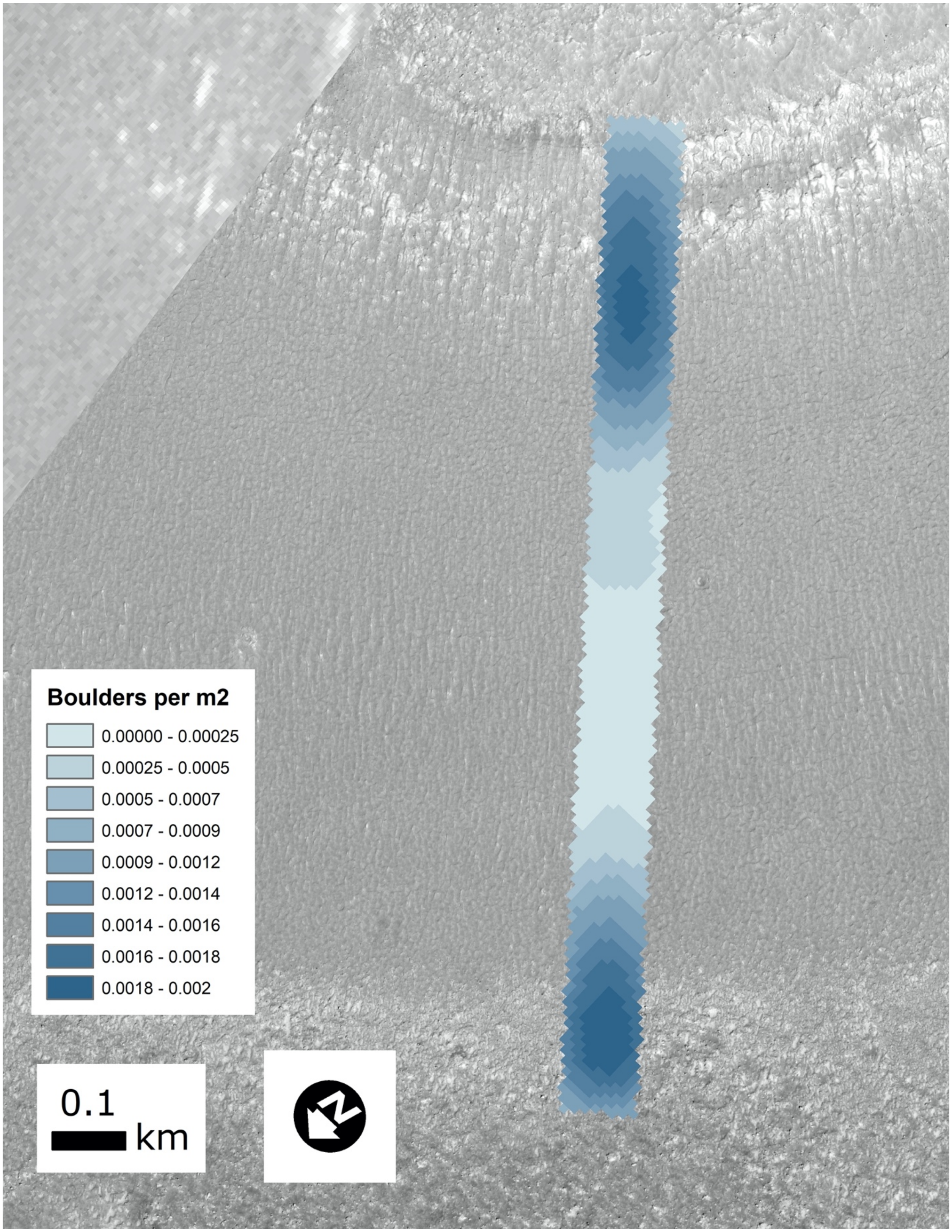
Boulders per m2
0.00000 - 0.00025
0.00025 - 0.0005
0.0005 - 0.0007
0.0007 - 0.0009
0.0009 - 0.0012
0.0012 - 0.0014
0.0014 - 0.0016
0.0016 - 0.0018
0.0018 - 0.002
0.1
km
N
Site DD1.

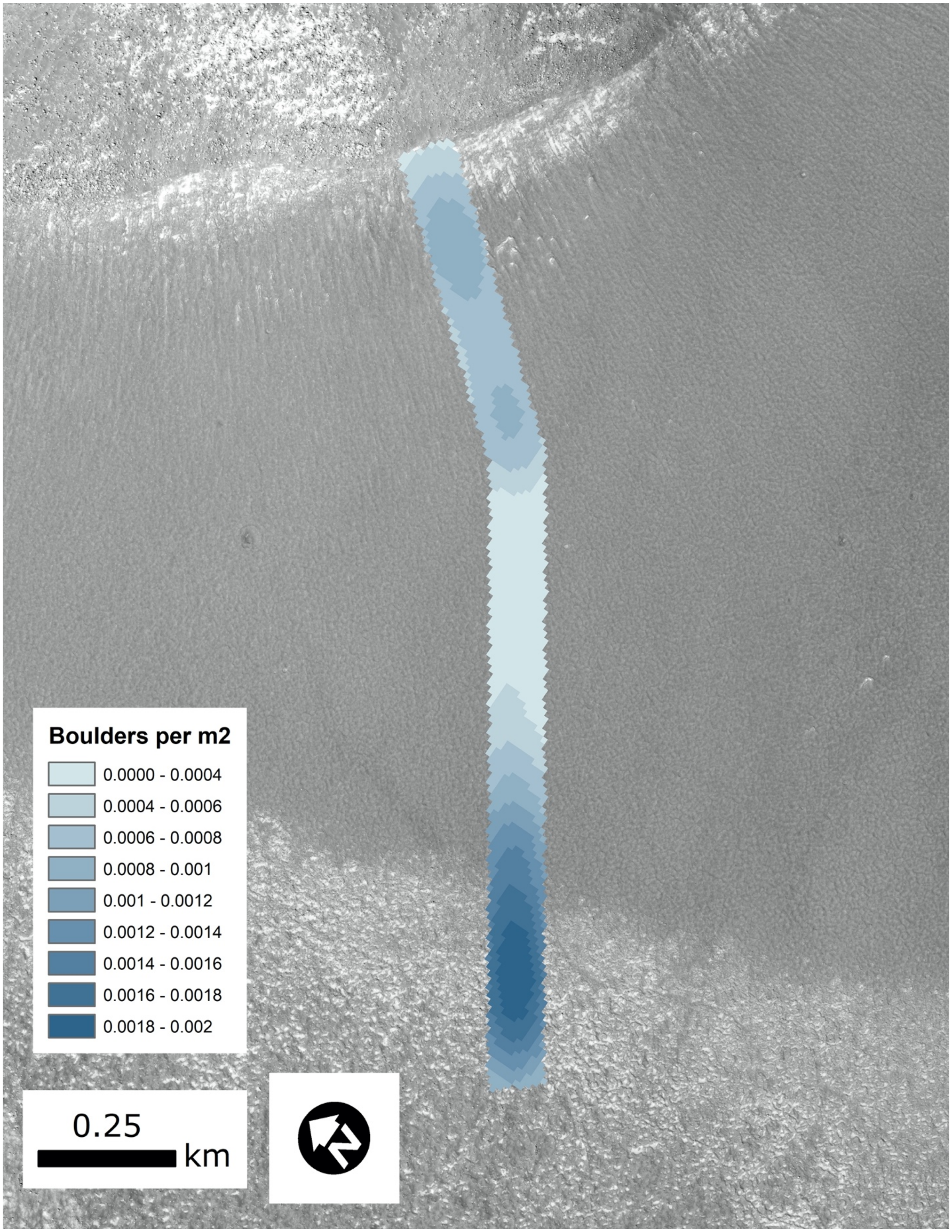
Boulders per m2
0.0000 - 0.0004
0.0004 - 0.0006
0.0006 - 0.0008
0.0008 - 0.001
0.001 - 0.0012
0.0012 - 0.0014
0.0014 - 0.0016
0.0016 - 0.0018
0.0018 - 0.002
0.25
km
Site DD2.

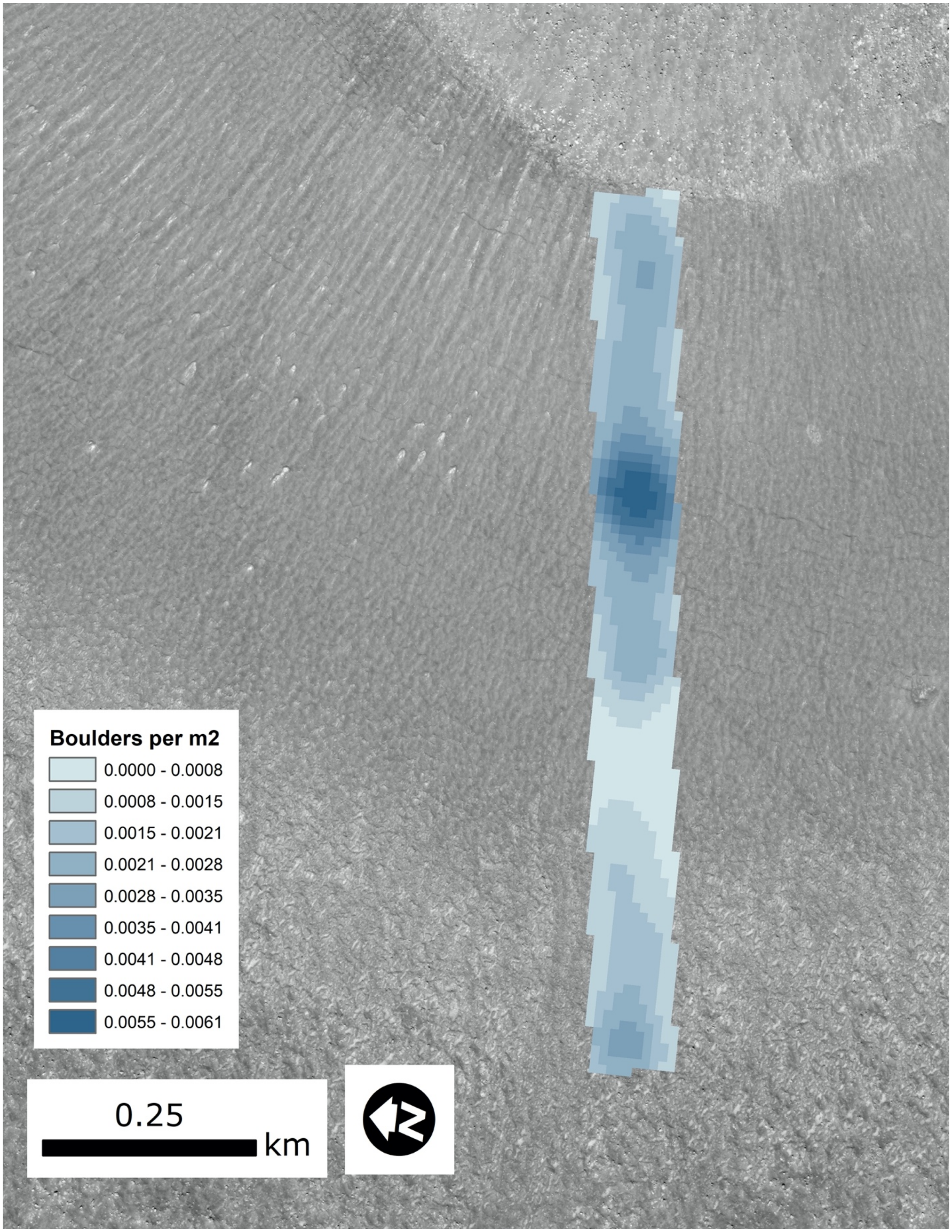
Boulders per m2
0.0000 - 0.0008
0.0008 - 0.0015
0.0015 - 0.0021
0.0021 - 0.0028
0.0028 - 0.0035
0.0035 - 0.0041
0.0041 - 0.0048
0.0048 - 0.0055
0.0055 - 0.0061
0.25
km
N
Site DD3.

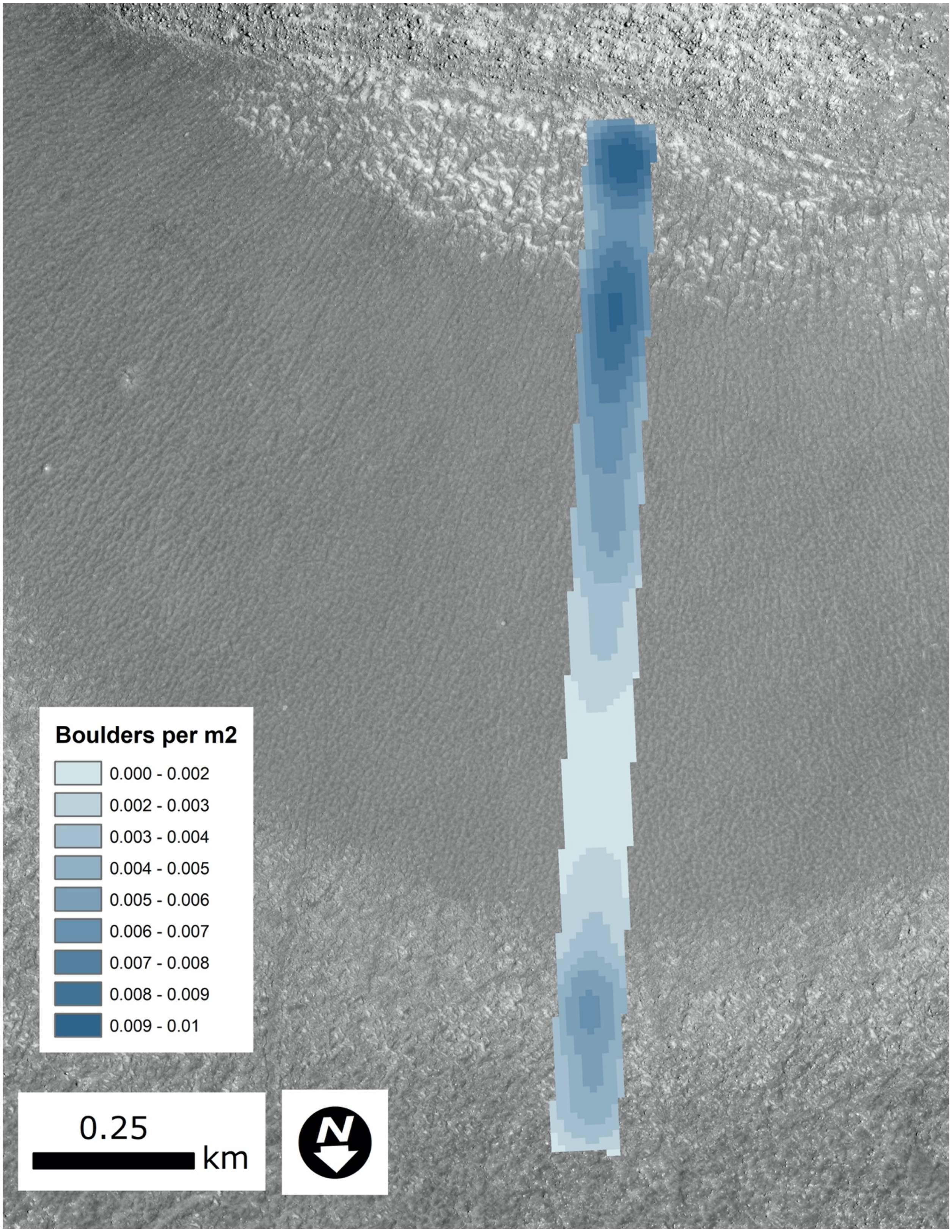
Boulders per m2
0.000 - 0.002
0.002 - 0.003
0.003 - 0.004
0.004 - 0.005
0.005 - 0.006
0.006 - 0.007
0.007 - 0.008
0.008 - 0.009
0.009 - 0.01
0.25
km
N
Site DD4.

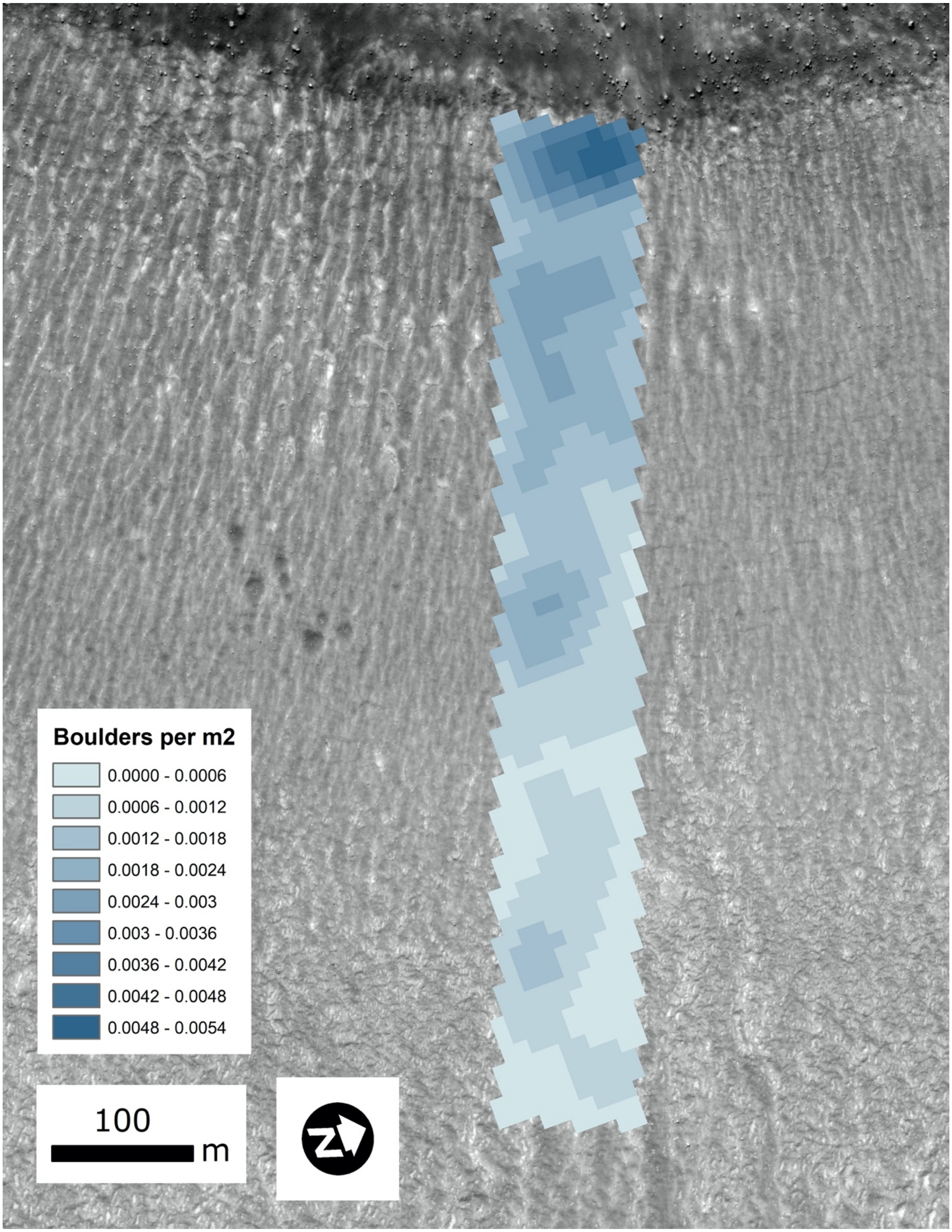
Boulders per m2
0.0000 - 0.0006
0.0006 - 0.0012
0.0012 - 0.0018
0.0018 - 0.0024
0.0024 - 0.003
0.003 - 0.0036
0.0036 - 0.0042
0.0042 - 0.0048
0.0048 - 0.0054
100
m

Site DD5.

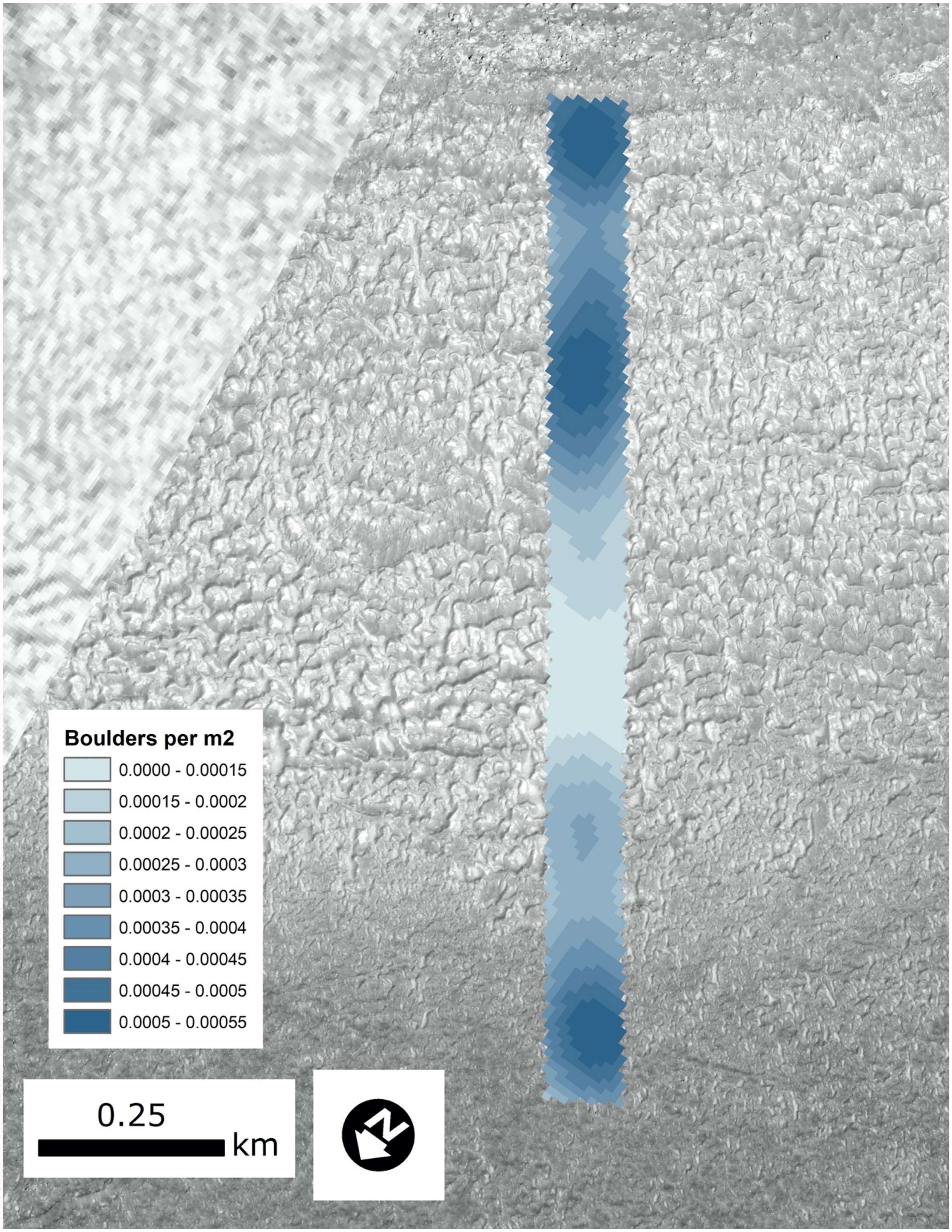

Boulders per m2
0.0000 - 0.00015
0.00015 - 0.0002
0.0002 - 0.00025
0.00025 - 0.0003
0.0003 - 0.00035
0.00035 - 0.0004
0.0004 - 0.00045
0.00045 - 0.0005
0.0005 - 0.00055
0.25
km

Site DD6.

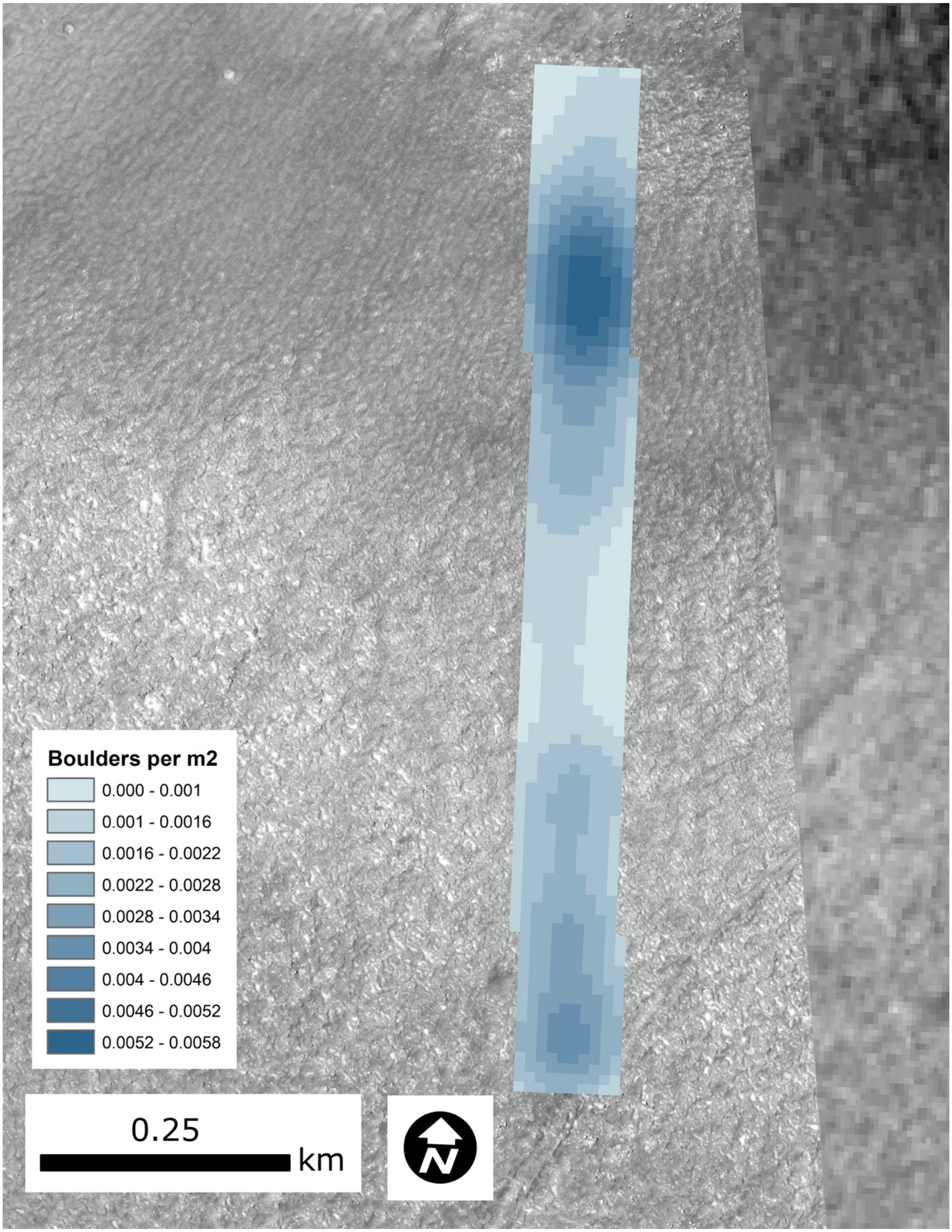

Boulders per m2
0.000 - 0.001
0.001 - 0.0016
0.0016 - 0.0022
0.0022 - 0.0028
0.0028 - 0.0034
0.0034 - 0.004
0.004 - 0.0046
0.0046 - 0.0052
0.0052 - 0.0058
0.25
km
N

Site DD7.

Site E

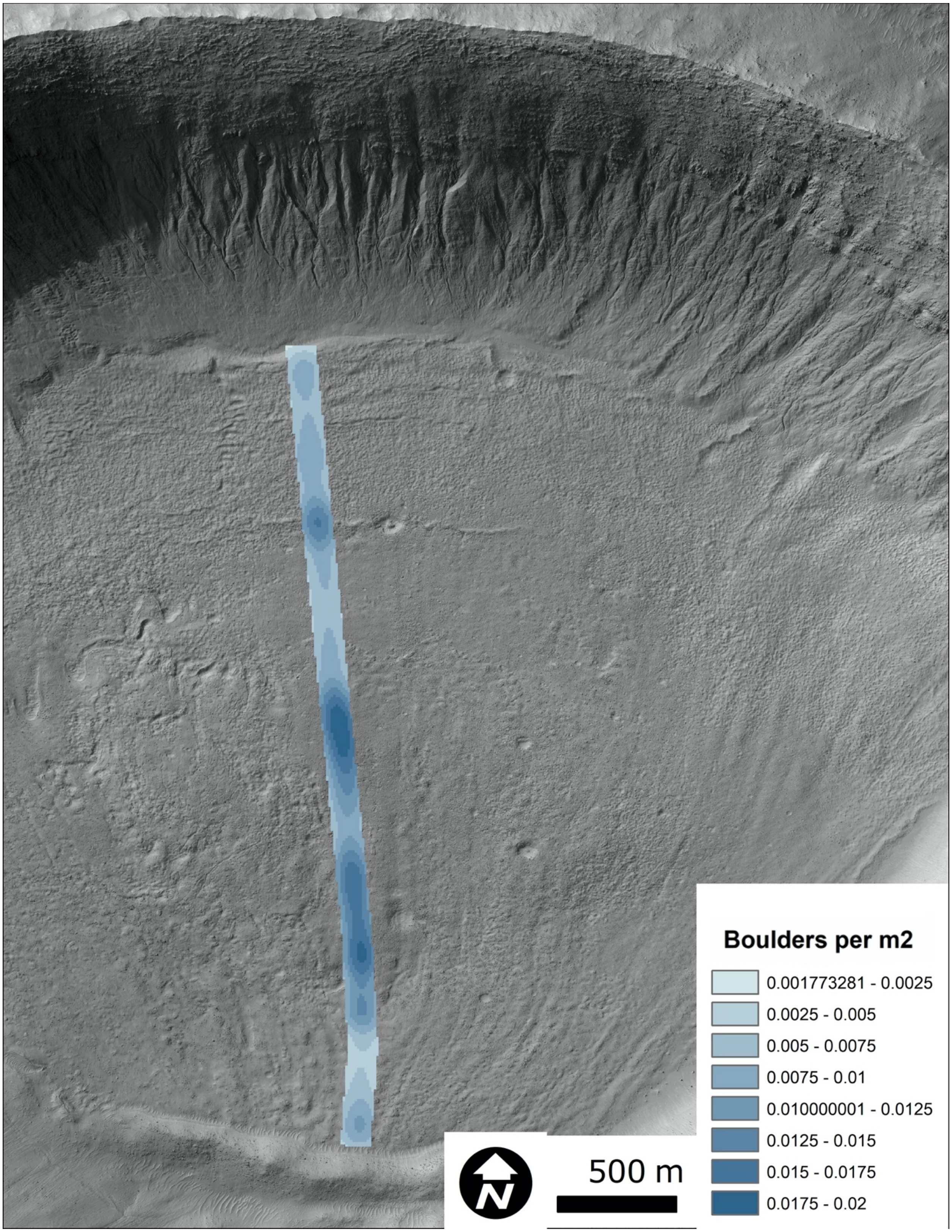

Boulders per m2
0.001773281 - 0.0025
0.0025 - 0.005
0.005 - 0.0075
0.0075 - 0.01
0.010000001 - 0.0125
0.0125 - 0.015
0.015 - 0.0175
0.0175 - 0.02
500 m
N

Site E

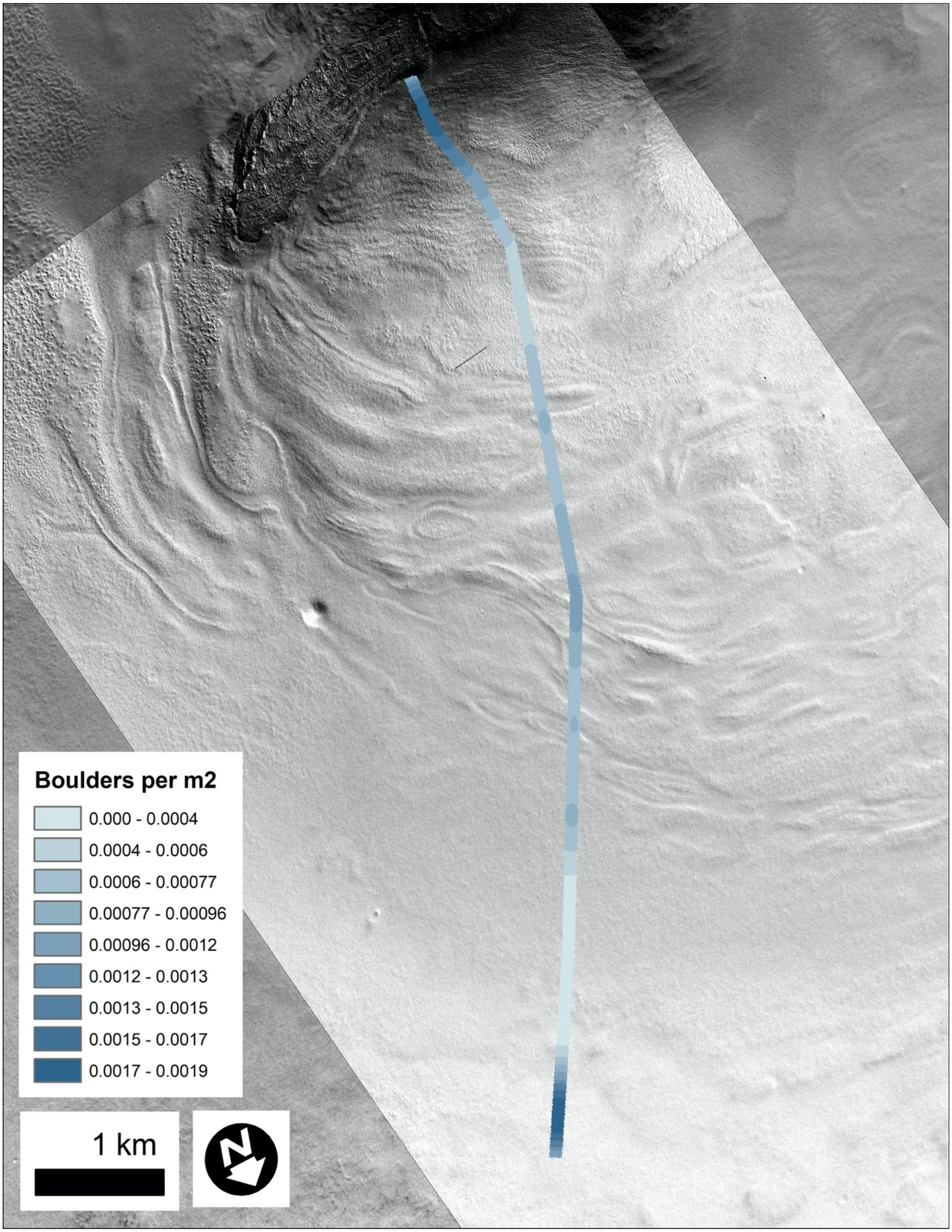
Boulders per m2
0.000 - 0.0004
0.0004 - 0.0006
0.0006 - 0.00077
0.00077 - 0.00096
0.00096 - 0.0012
0.0012 - 0.0013
0.0013 - 0.0015
0.0015 - 0.0017
0.0017 - 0.0019
1 km
N
Site EE

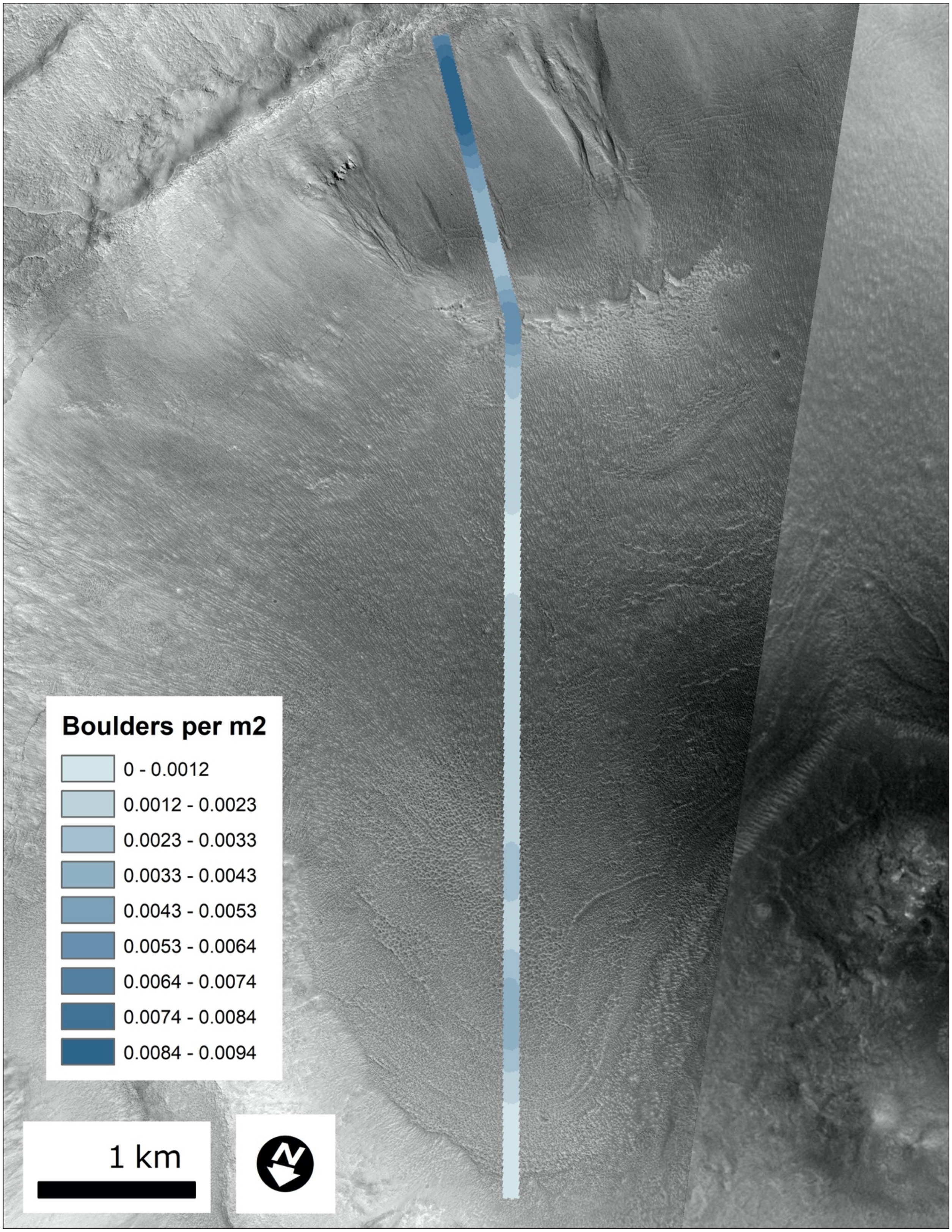
Boulders per m2
0 - 0.0012
0.0012 - 0.0023
0.0023 - 0.0033
0.0033 - 0.0043
0.0043 - 0.0053
0.0053 - 0.0064
0.0064 - 0.0074
0.0074 - 0.0084
0.0084 - 0.0094
1 km
N

Site F.

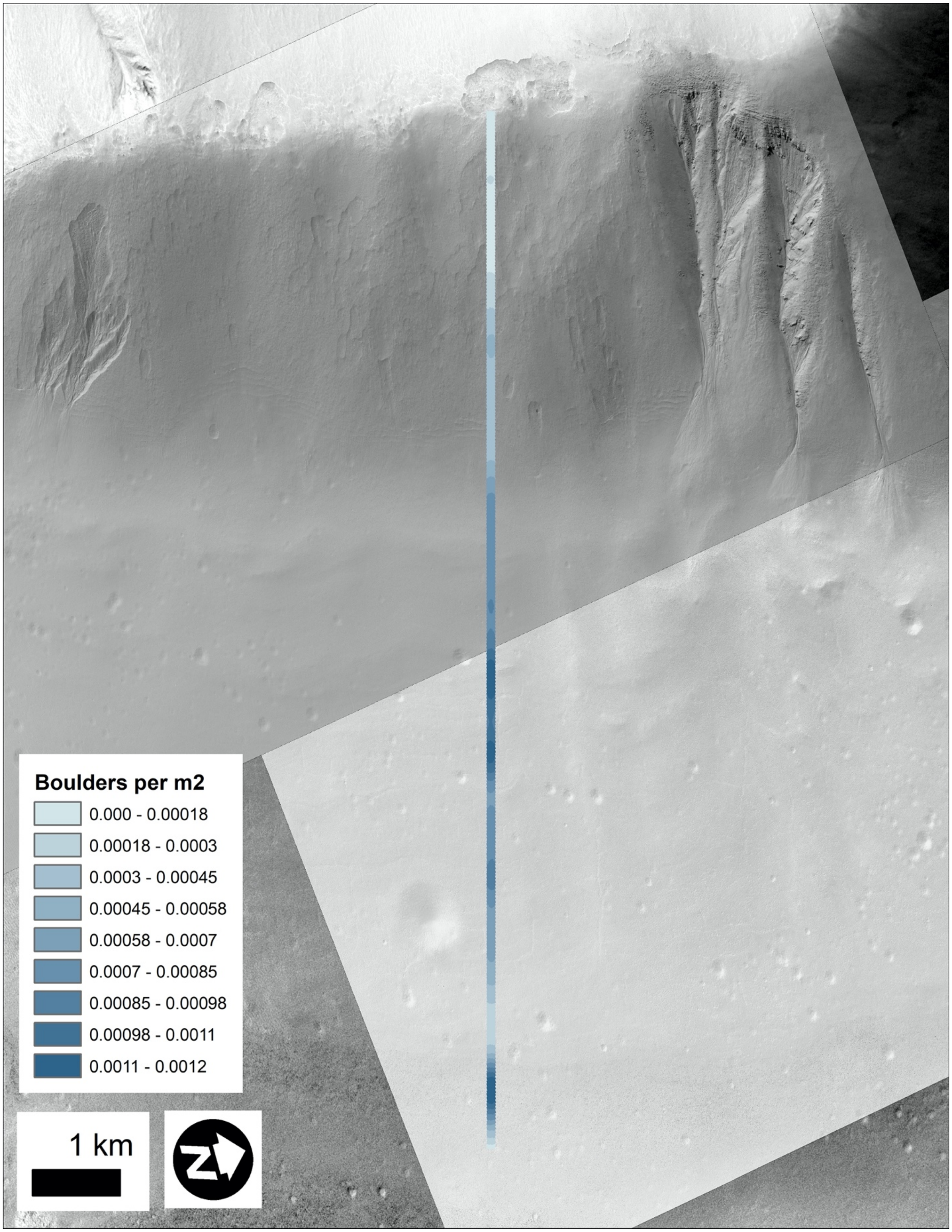

Boulders per m2
0.000 - 0.00018
0.00018 - 0.0003
0.0003 - 0.00045
0.00045 - 0.00058
0.00058 - 0.0007
0.0007 - 0.00085
0.00085 - 0.00098
0.00098 - 0.0011
0.0011 - 0.0012
1 km

Site FF

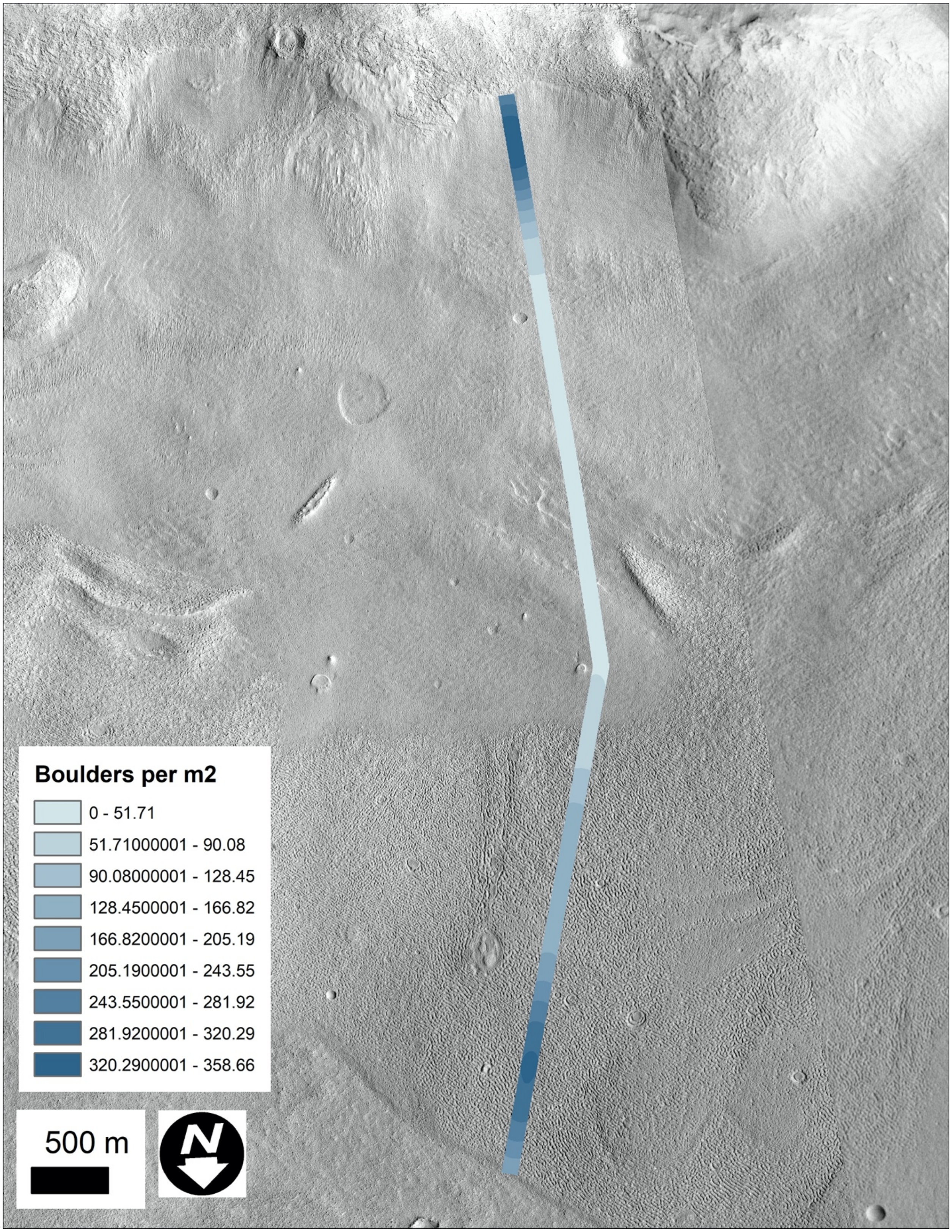
Boulders per m2
0 - 51.71
51.71000001 - 90.08
90.08000001 - 128.45
128.4500001 - 166.82
166.8200001 - 205.19
205.1900001 - 243.55
243.5500001 - 281.92
281.9200001 - 320.29
320.2900001 - 358.66
500 m
N
Site GG.

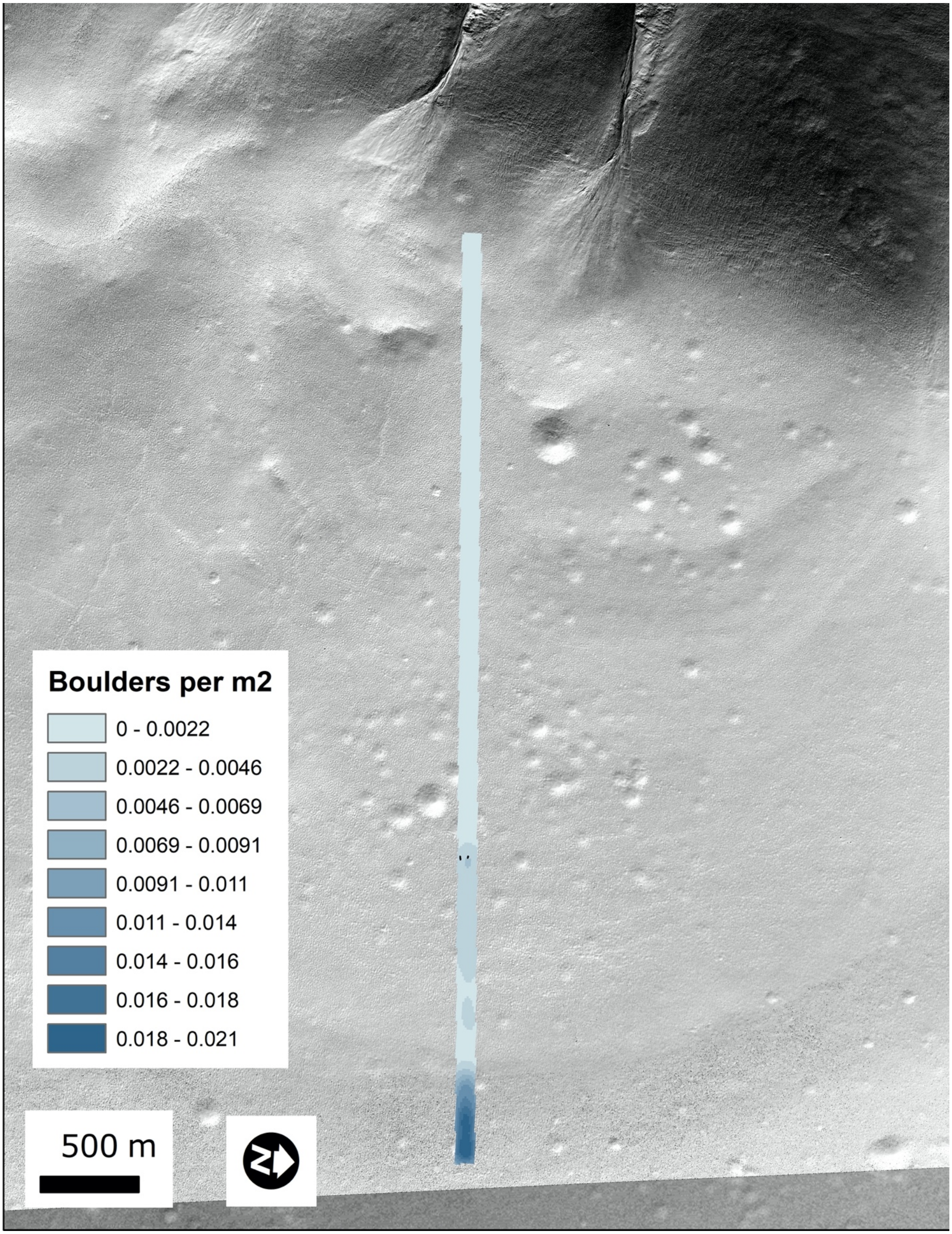

Boulders per m2
0 - 0.0022
0.0022 - 0.0046
0.0046 - 0.0069
0.0069 - 0.0091
0.0091 - 0.011
0.011 - 0.014
0.014 - 0.016
0.016 - 0.018
0.018 - 0.021
500 m
N

Site H1.

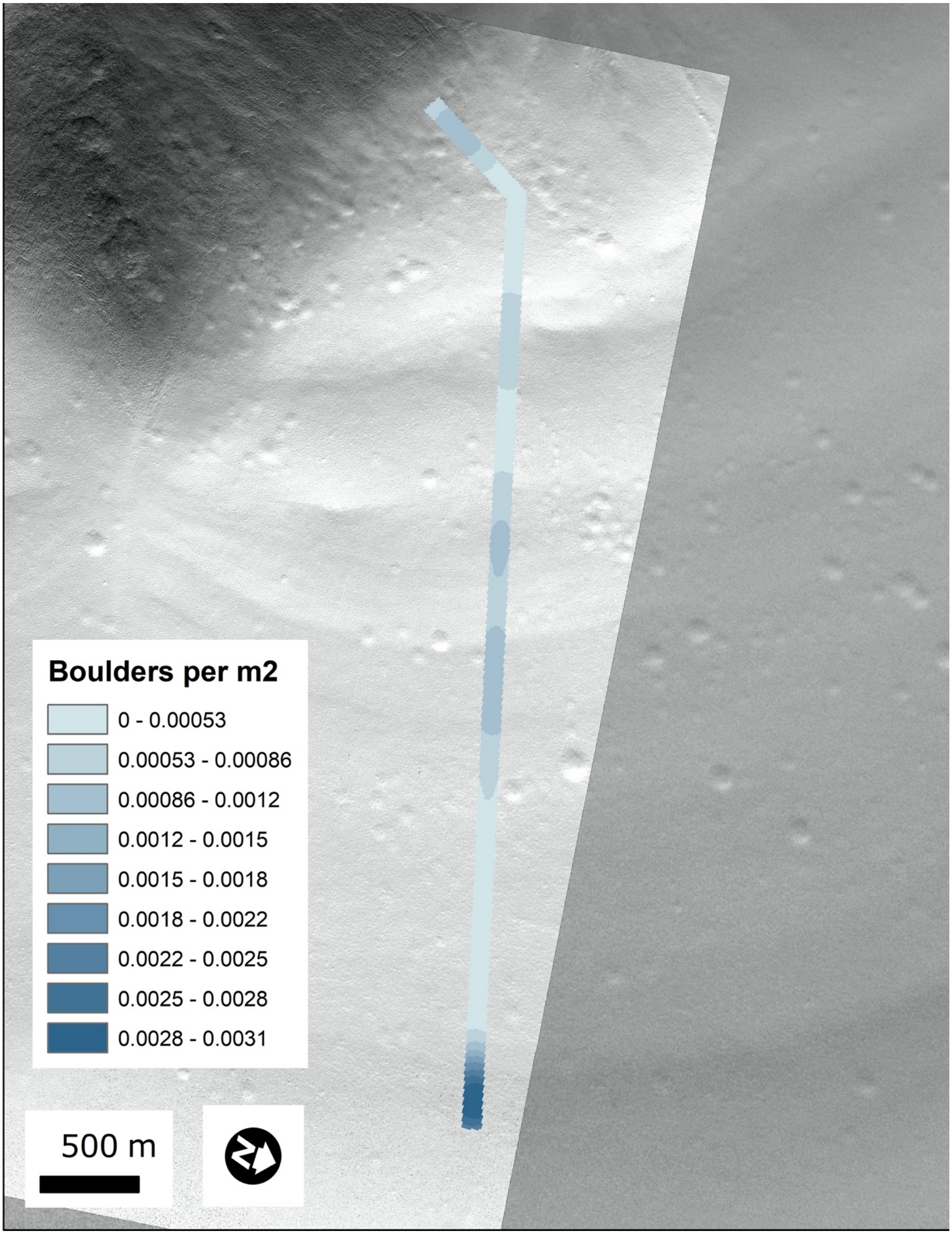

Boulders per m2
0 - 0.00053
0.00053 - 0.00086
0.00086 - 0.0012
0.0012 - 0.0015
0.0015 - 0.0018
0.0018 - 0.0022
0.0022 - 0.0025
0.0025 - 0.0028
0.0028 - 0.0031
500 m

Site H2.

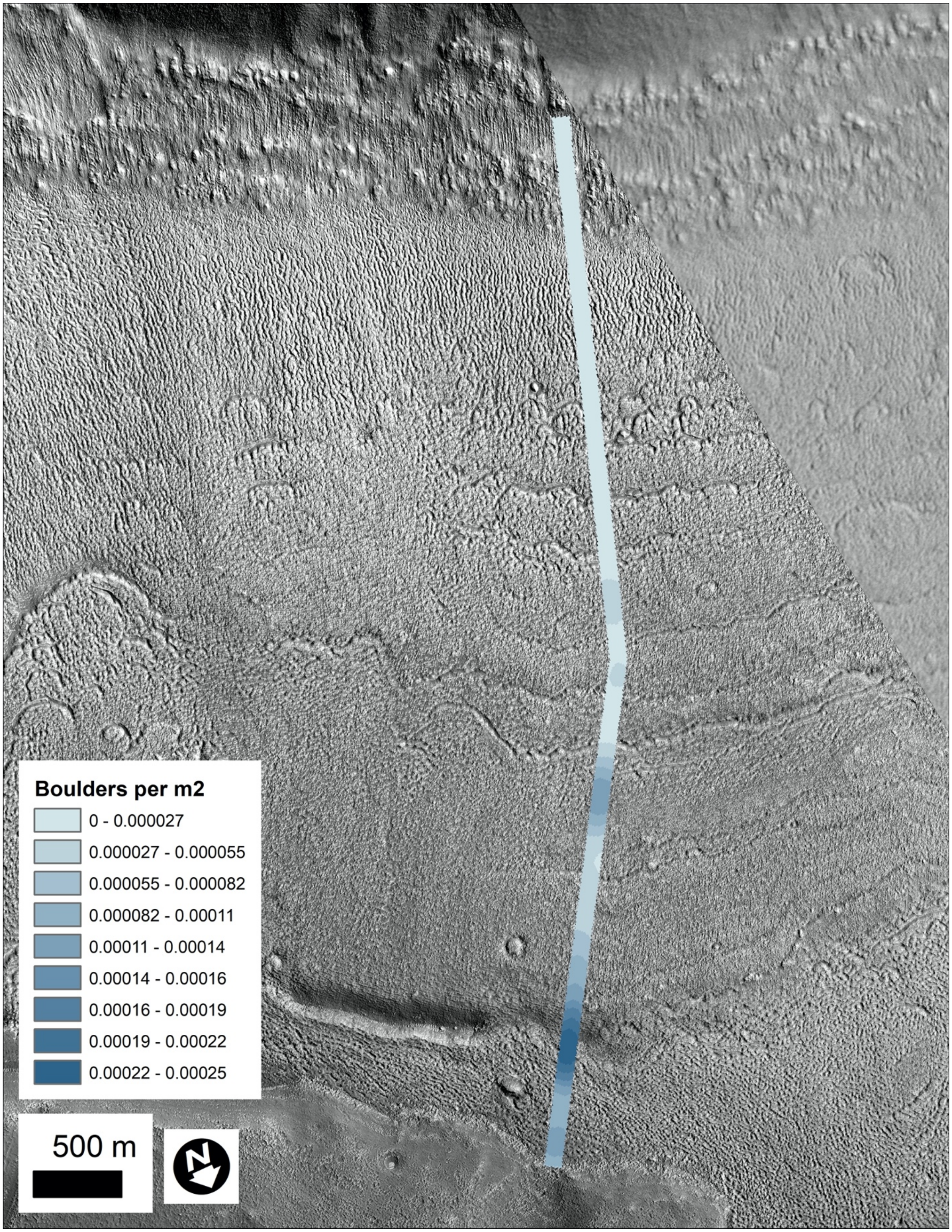
Boulders per m2
0 - 0.000027
0.000027 - 0.000055
0.000055 - 0.000082
0.000082 - 0.00011
0.00011 - 0.00014
0.00014 - 0.00016
0.00016 - 0.00019
0.00019 - 0.00022
0.00022 - 0.00025
500 m
N
Site HH.

Site J1.

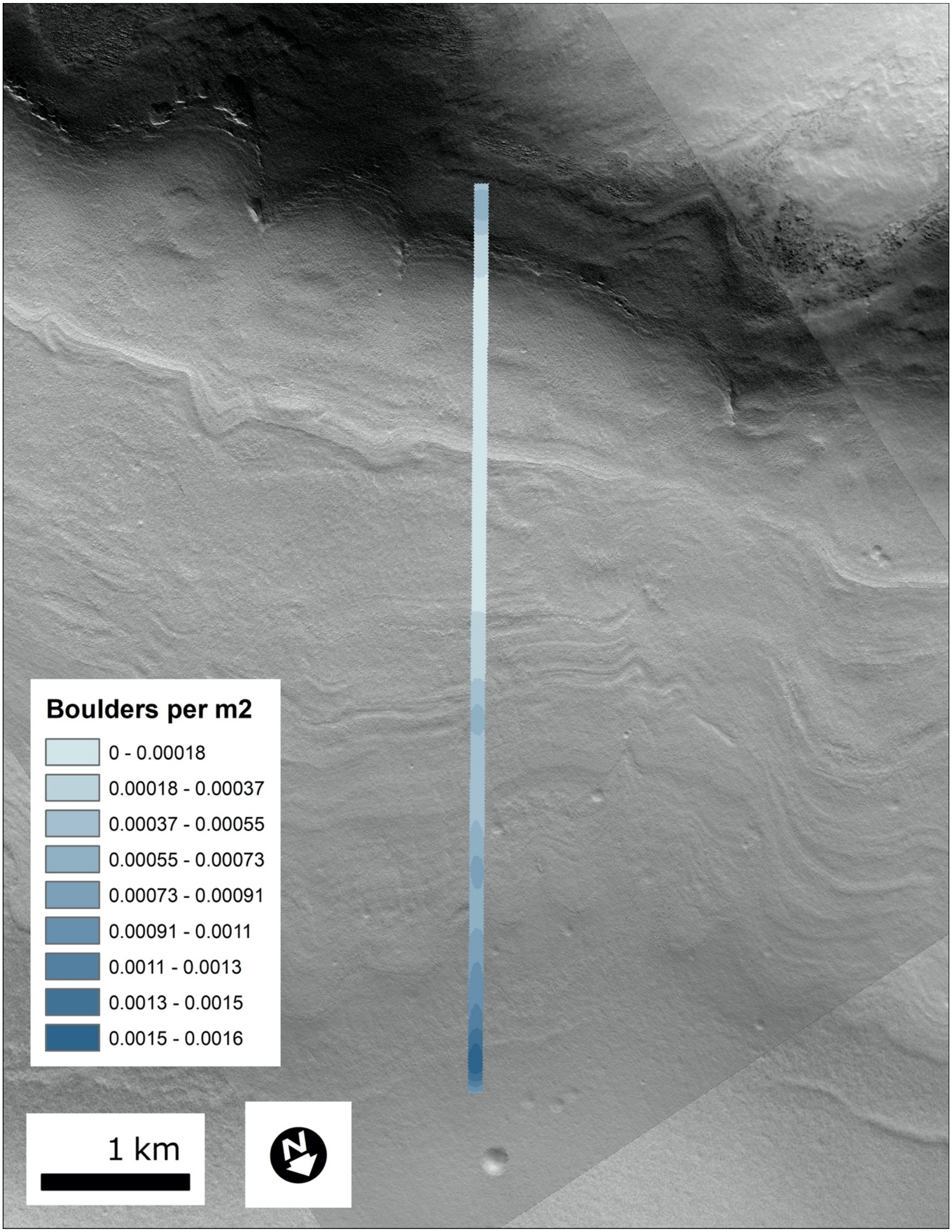

Site J1.

Site J2.

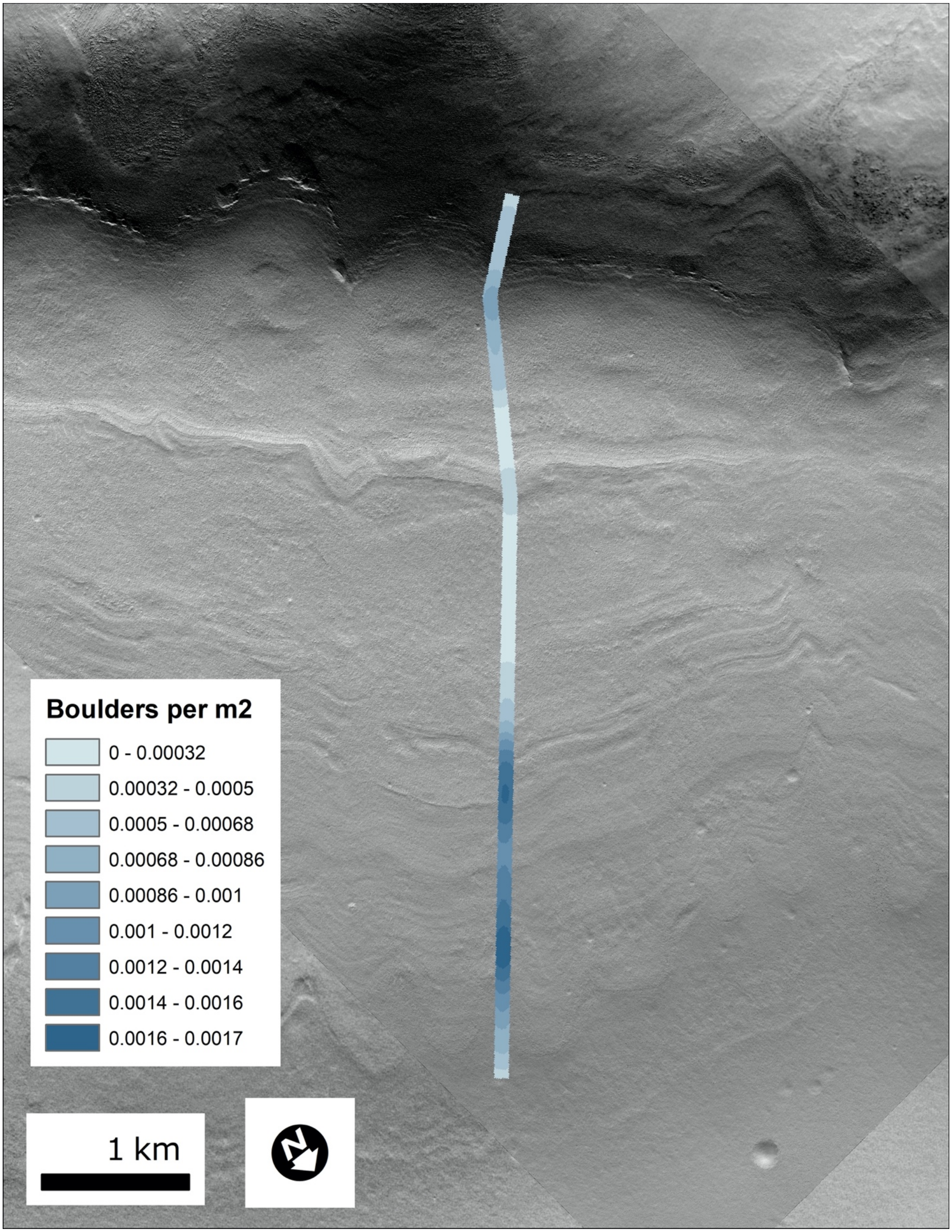

Site J2.

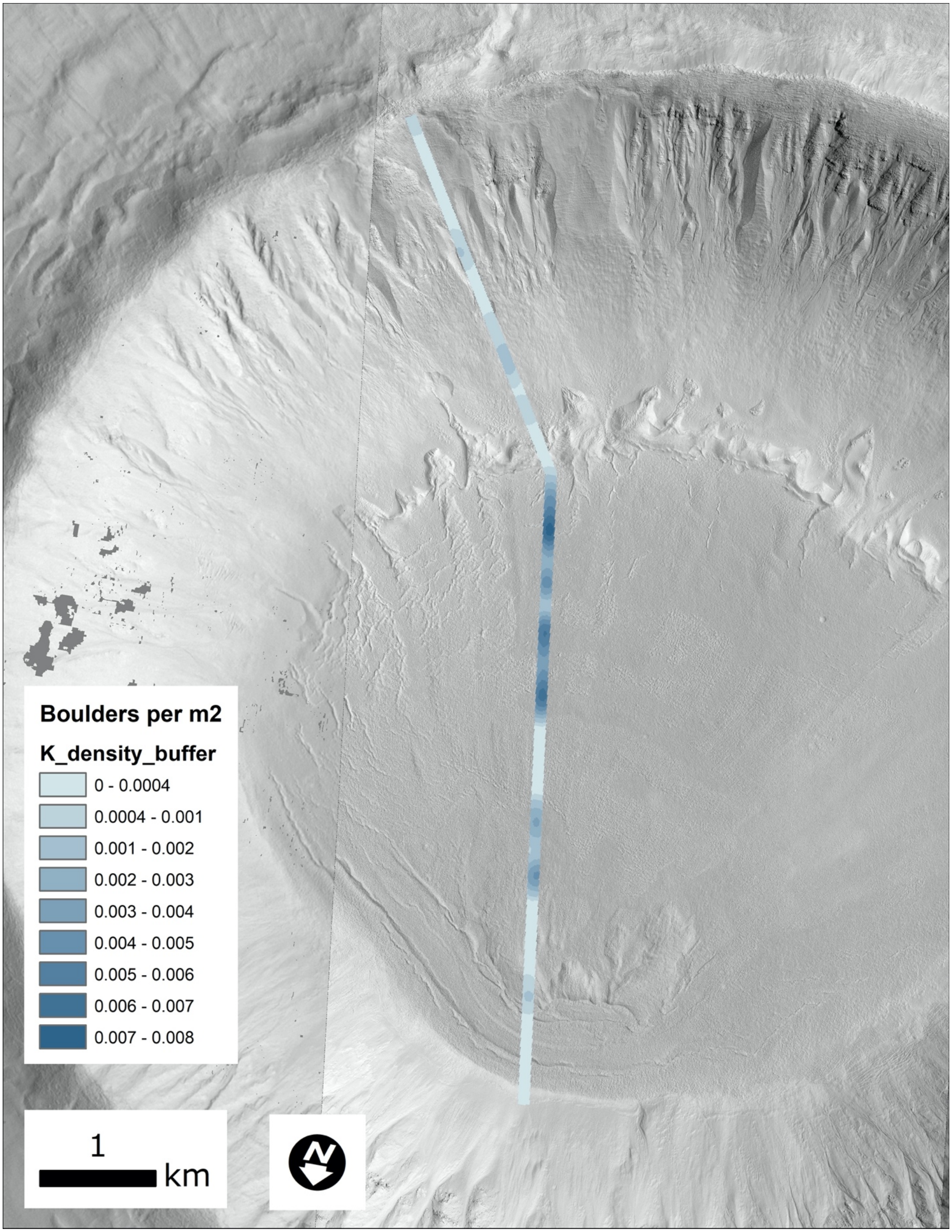

Boulders per m2
K_density_buffer
0 - 0.0004
0.0004 - 0.001
0.001 - 0.002
0.002 - 0.003
0.003 - 0.004
0.004 - 0.005
0.005 - 0.006
0.006 - 0.007
0.007 - 0.008
1
km
N

Site K.

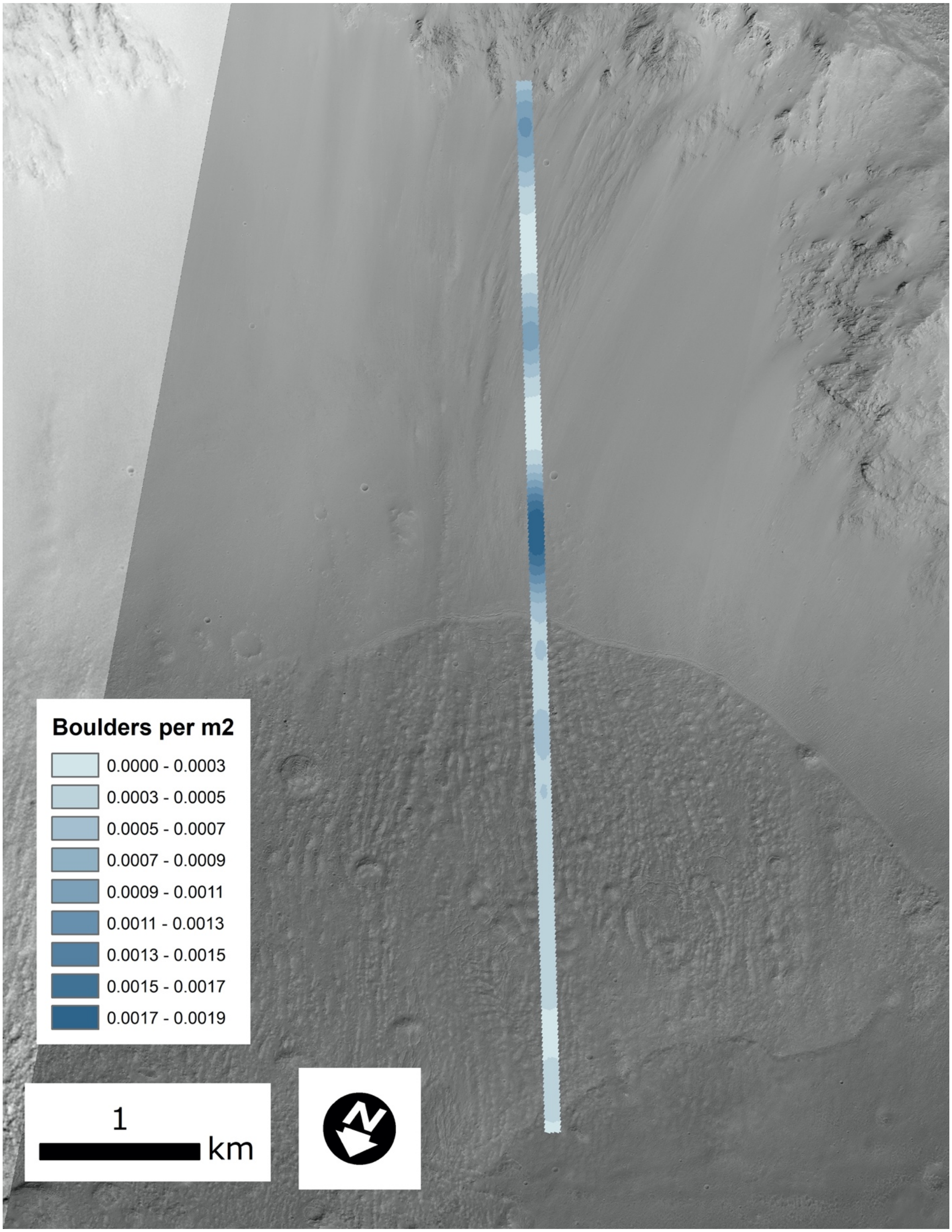
Boulders per m2
0.0000 - 0.0003
0.0003 - 0.0005
0.0005 - 0.0007
0.0007 - 0.0009
0.0009 - 0.0011
0.0011 - 0.0013
0.0013 - 0.0015
0.0015 - 0.0017
0.0017 - 0.0019
1
km
N
Site L.

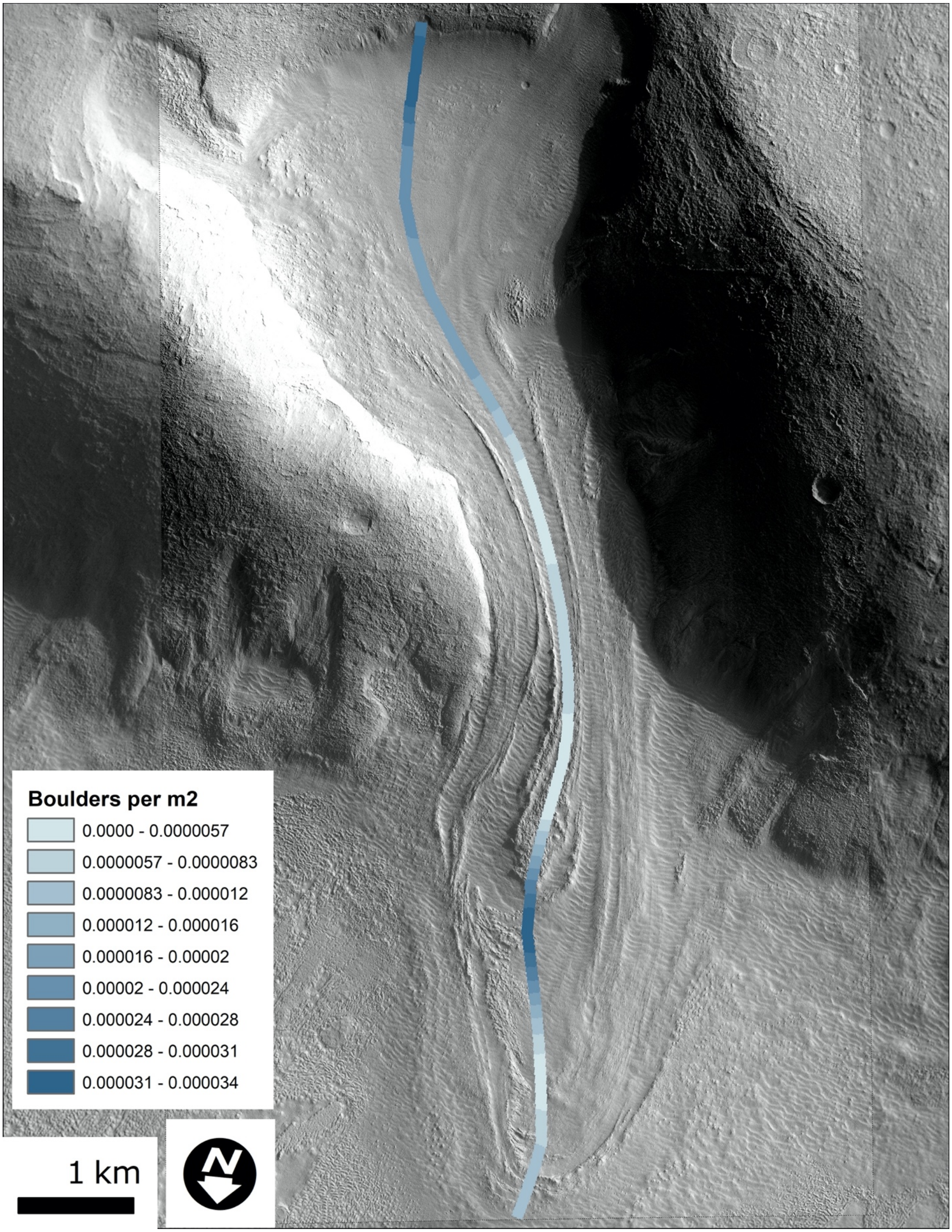
Boulders per m2
0.0000 - 0.0000057
0.0000057 - 0.0000083
0.0000083 - 0.000012
0.000012 - 0.000016
0.000016 - 0.00002
0.00002 - 0.000024
0.000024 - 0.000028
0.000028 - 0.000031
0.000031 - 0.000034
1 km
N

Site M.

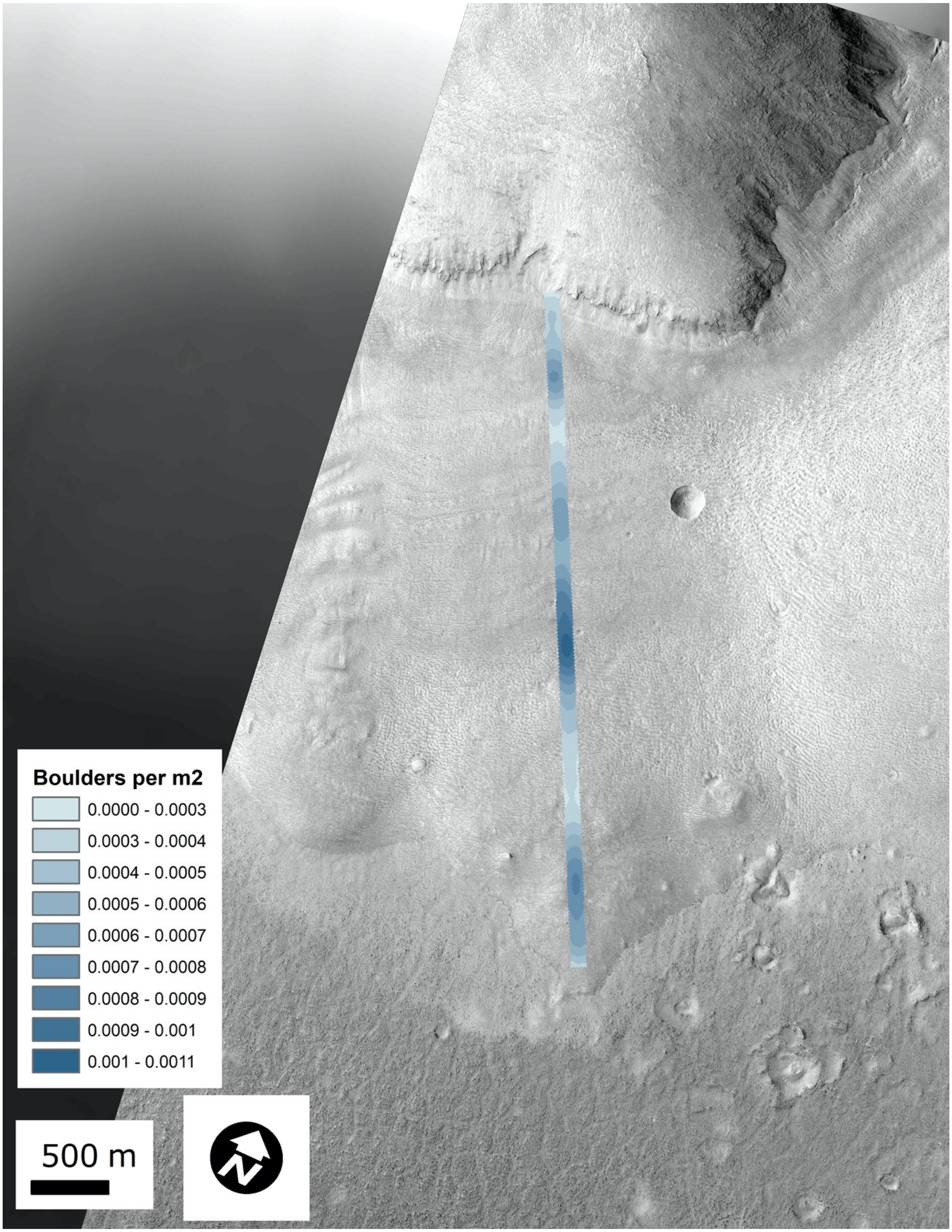
Boulders per m2
0.0000 - 0.0003
0.0003 - 0.0004
0.0004 - 0.0005
0.0005 - 0.0006
0.0006 - 0.0007
0.0007 - 0.0008
0.0008 - 0.0009
0.0009 - 0.001
0.001 - 0.0011
500 m
N
Site N.

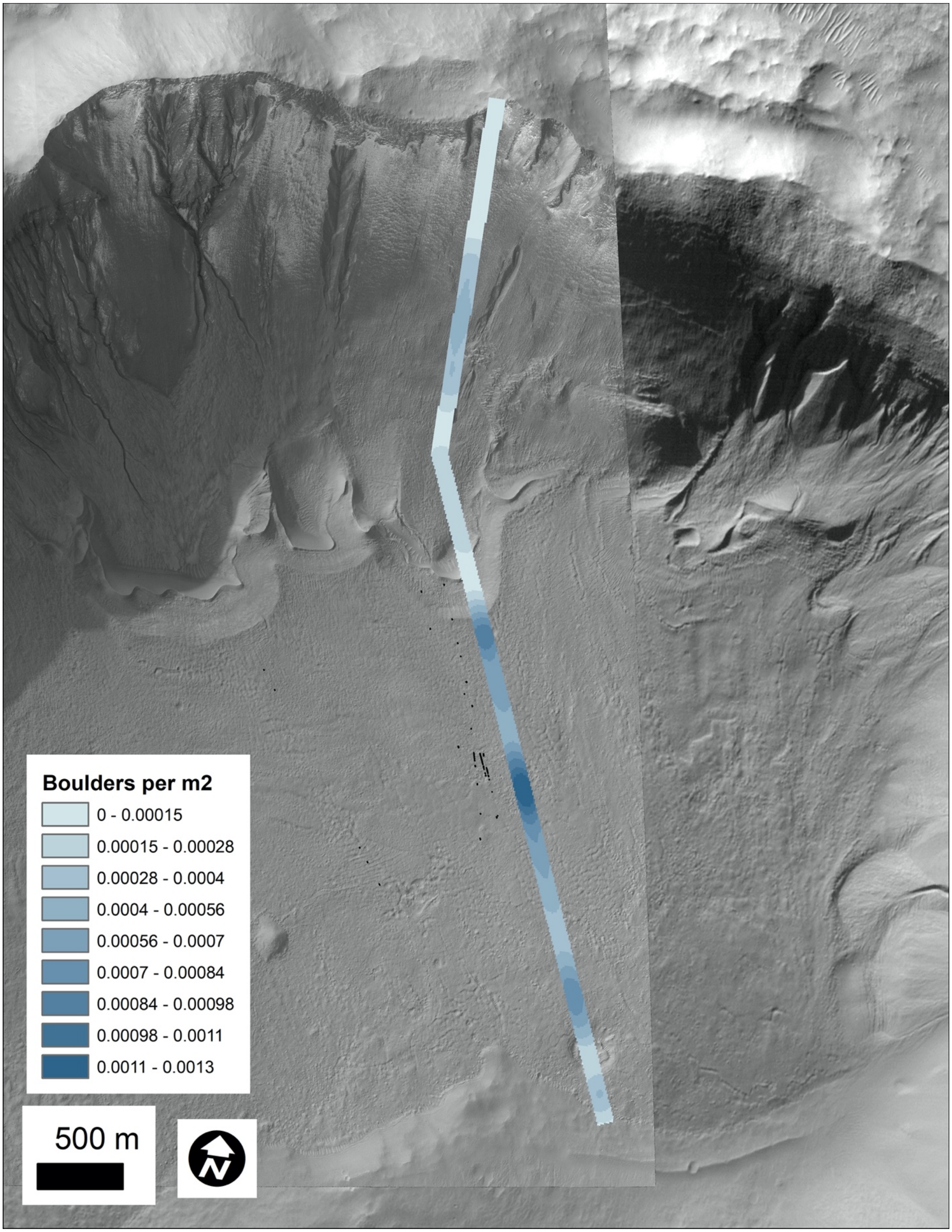

Boulders per m2
0 - 0.00015
0.00015 - 0.00028
0.00028 - 0.0004
0.0004 - 0.00056
0.00056 - 0.0007
0.0007 - 0.00084
0.00084 - 0.00098
0.00098 - 0.0011
0.0011 - 0.0013
500 m
N

Site P.

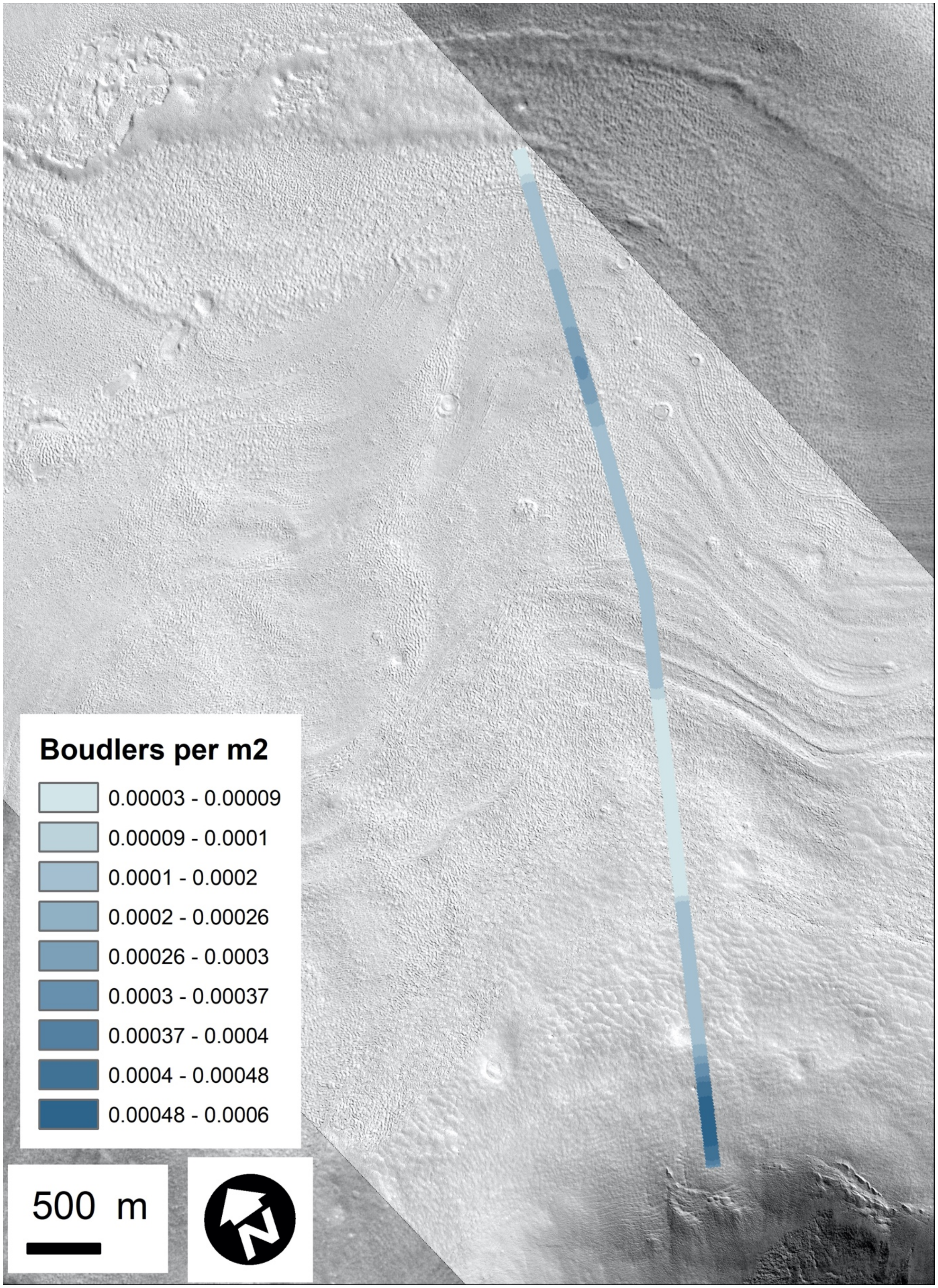

Boudlers per m2
0.00003 - 0.00009
0.00009 - 0.0001
0.0001 - 0.0002
0.0002 - 0.00026
0.00026 - 0.0003
0.0003 - 0.00037
0.00037 - 0.0004
0.0004 - 0.00048
0.00048 - 0.0006
500 m
N

Site Q.

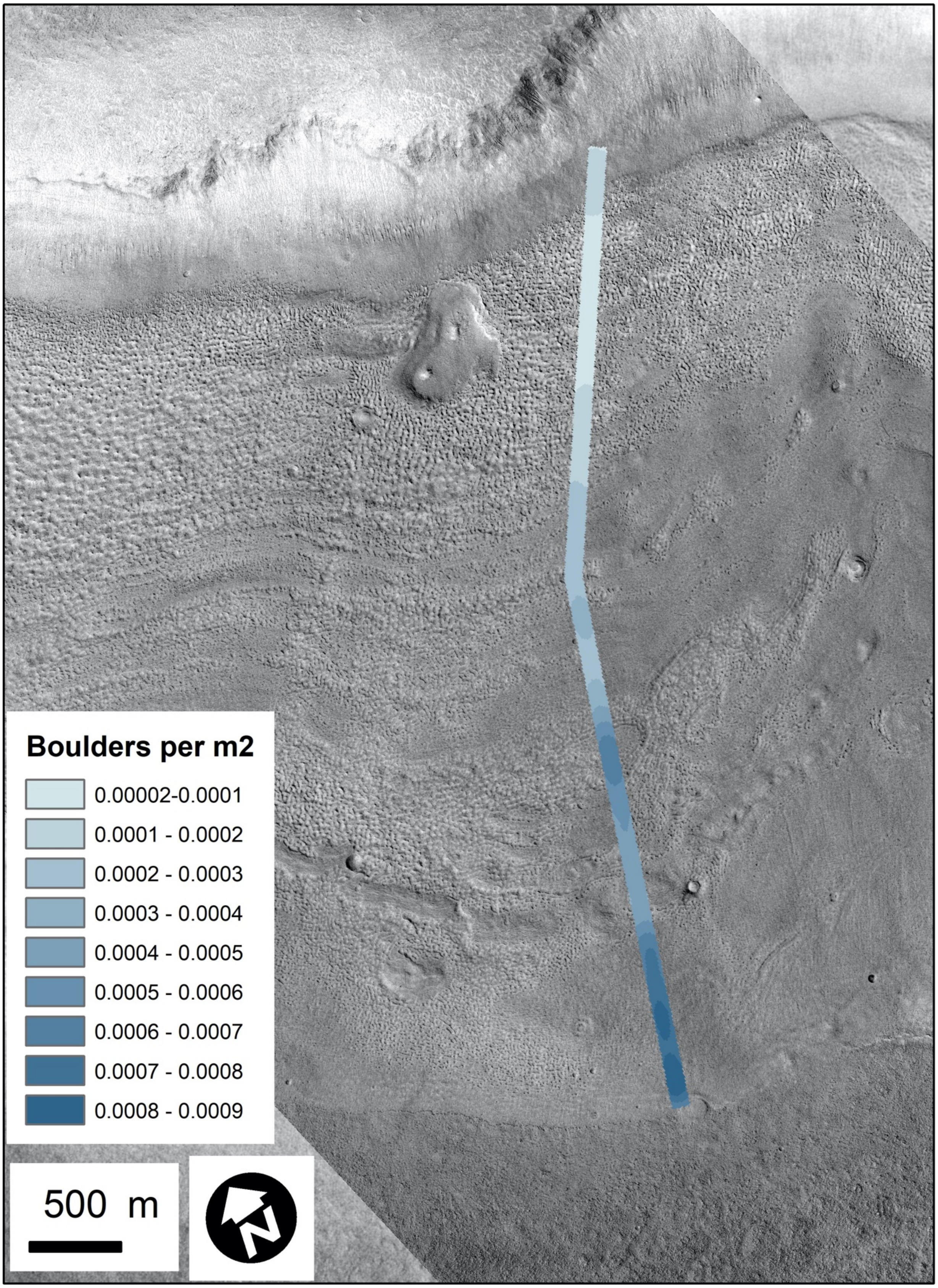
Boulders per m2
0.00002-0.0001
0.0001 - 0.0002
0.0002 - 0.0003
0.0003 - 0.0004
0.0004 - 0.0005
0.0005 - 0.0006
0.0006 - 0.0007
0.0007 - 0.0008
0.0008 - 0.0009
500 m
N
Site Q2.

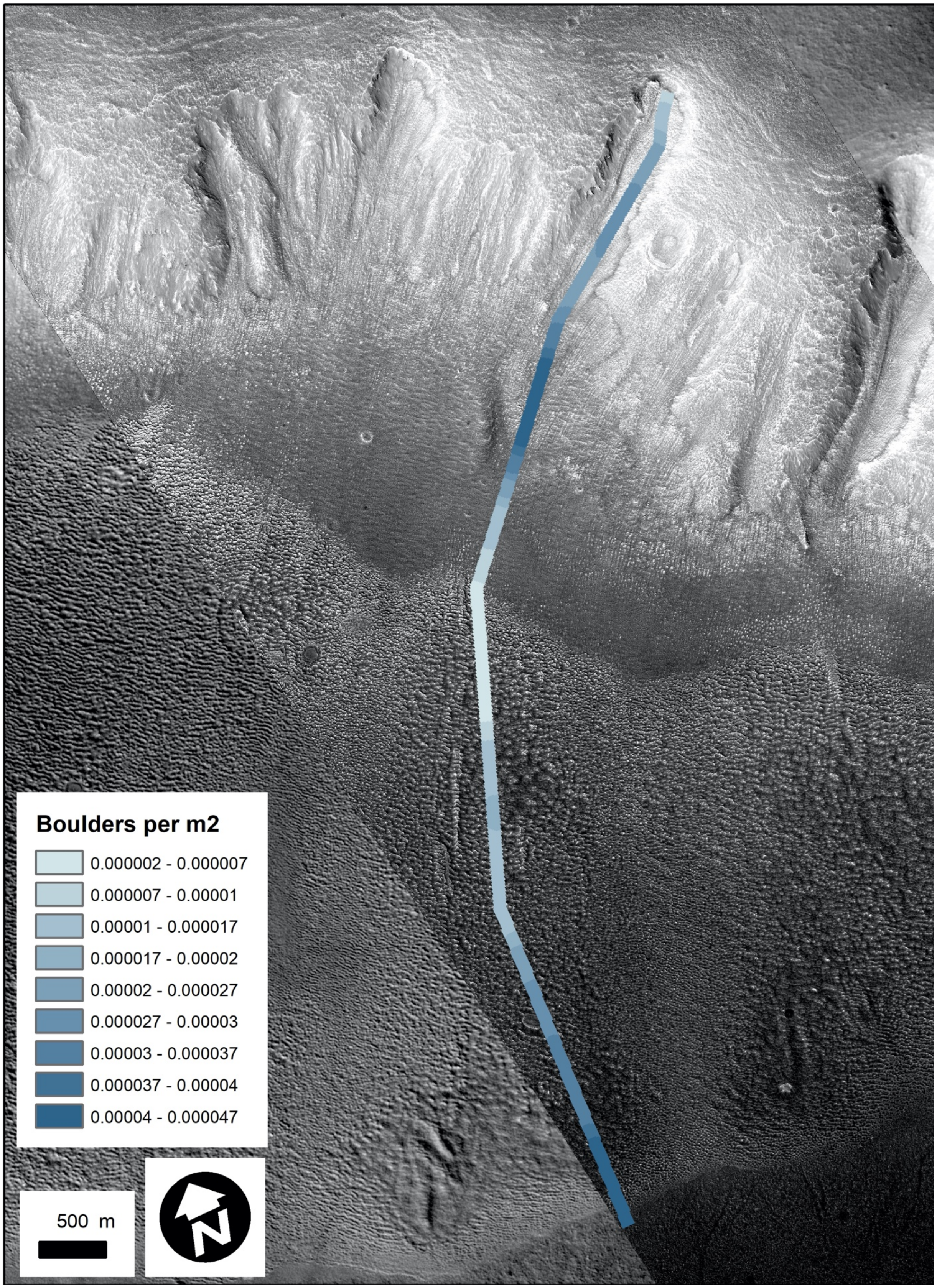
Boulders per m2
0.000002 - 0.000007
0.000007 - 0.00001
0.00001 - 0.000017
0.000017 - 0.00002
0.00002 - 0.000027
0.000027 - 0.00003
0.00003 - 0.000037
0.000037 - 0.00004
0.00004 - 0.000047
500 m
N
Site R.

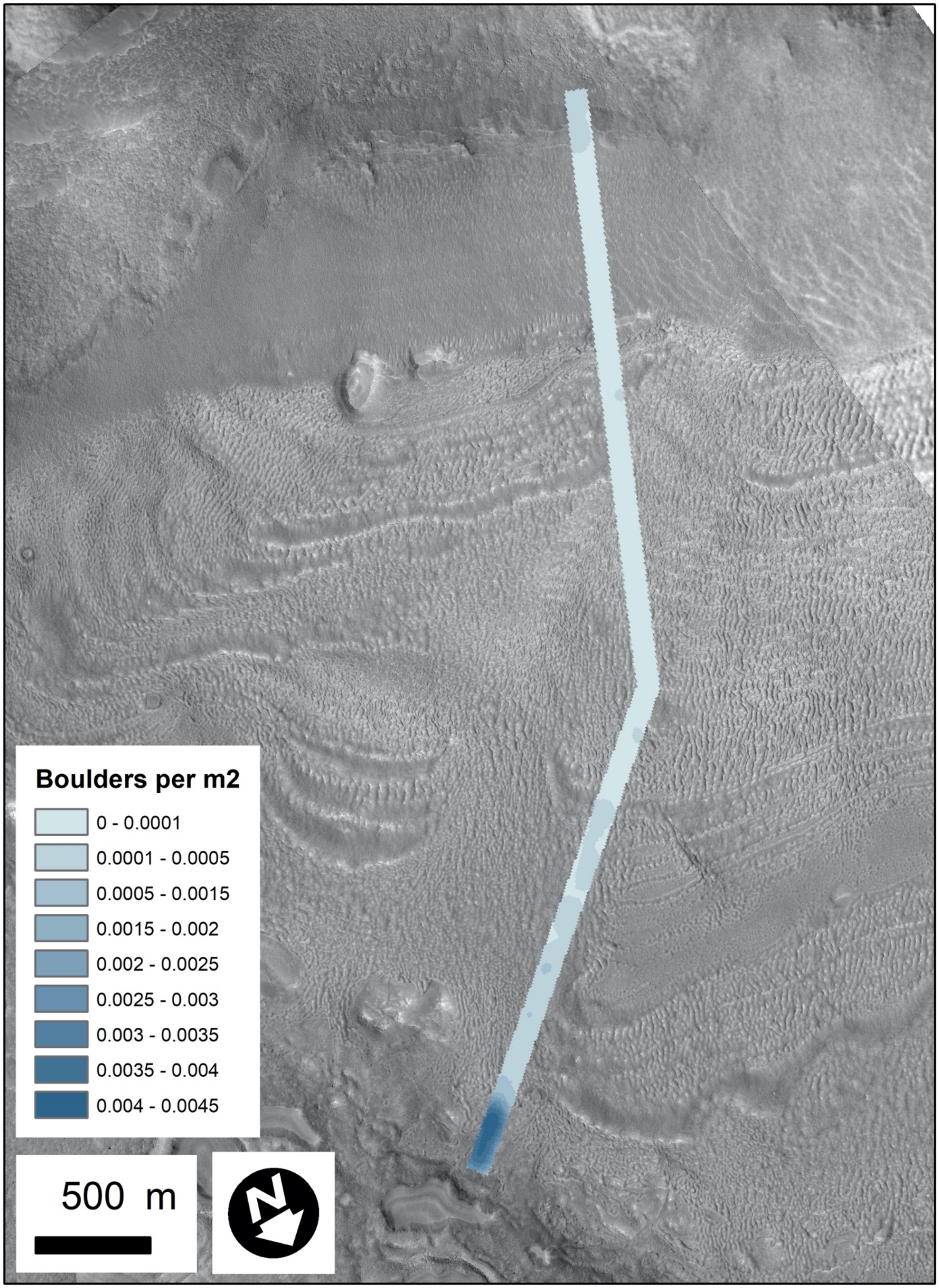

Boulders per m2
0 - 0.0001
0.0001 - 0.0005
0.0005 - 0.0015
0.0015 - 0.002
0.002 - 0.0025
0.0025 - 0.003
0.003 - 0.0035
0.0035 - 0.004
0.004 - 0.0045
500 m
N

Site S.

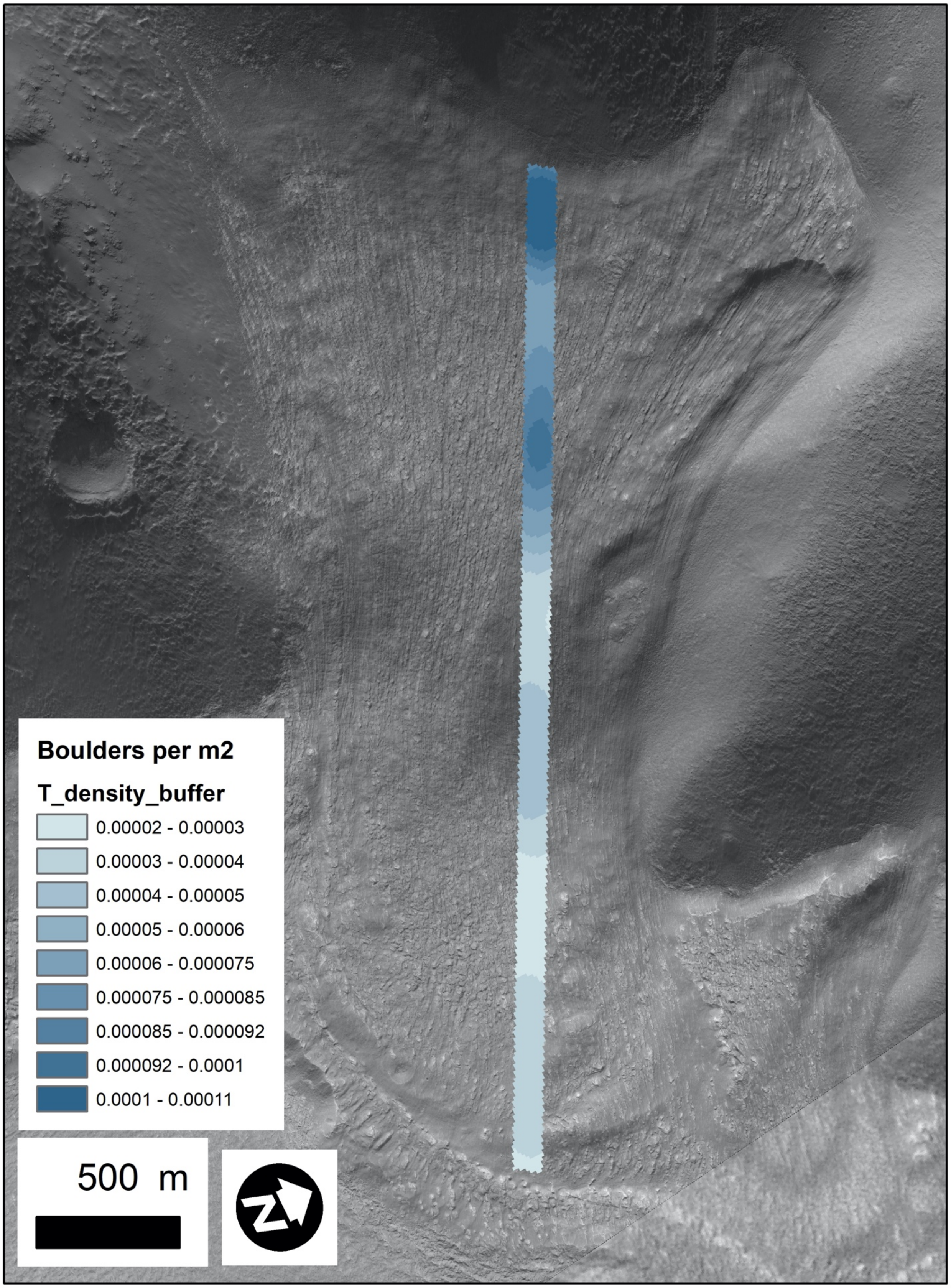
Boulders per m2
T_density_buffer
0.00002 - 0.00003
0.00003 - 0.00004
0.00004 - 0.00005
0.00005 - 0.00006
0.00006 - 0.000075
0.000075 - 0.000085
0.000085 - 0.000092
0.000092 - 0.0001
0.0001 - 0.00011
500 m

Site T.

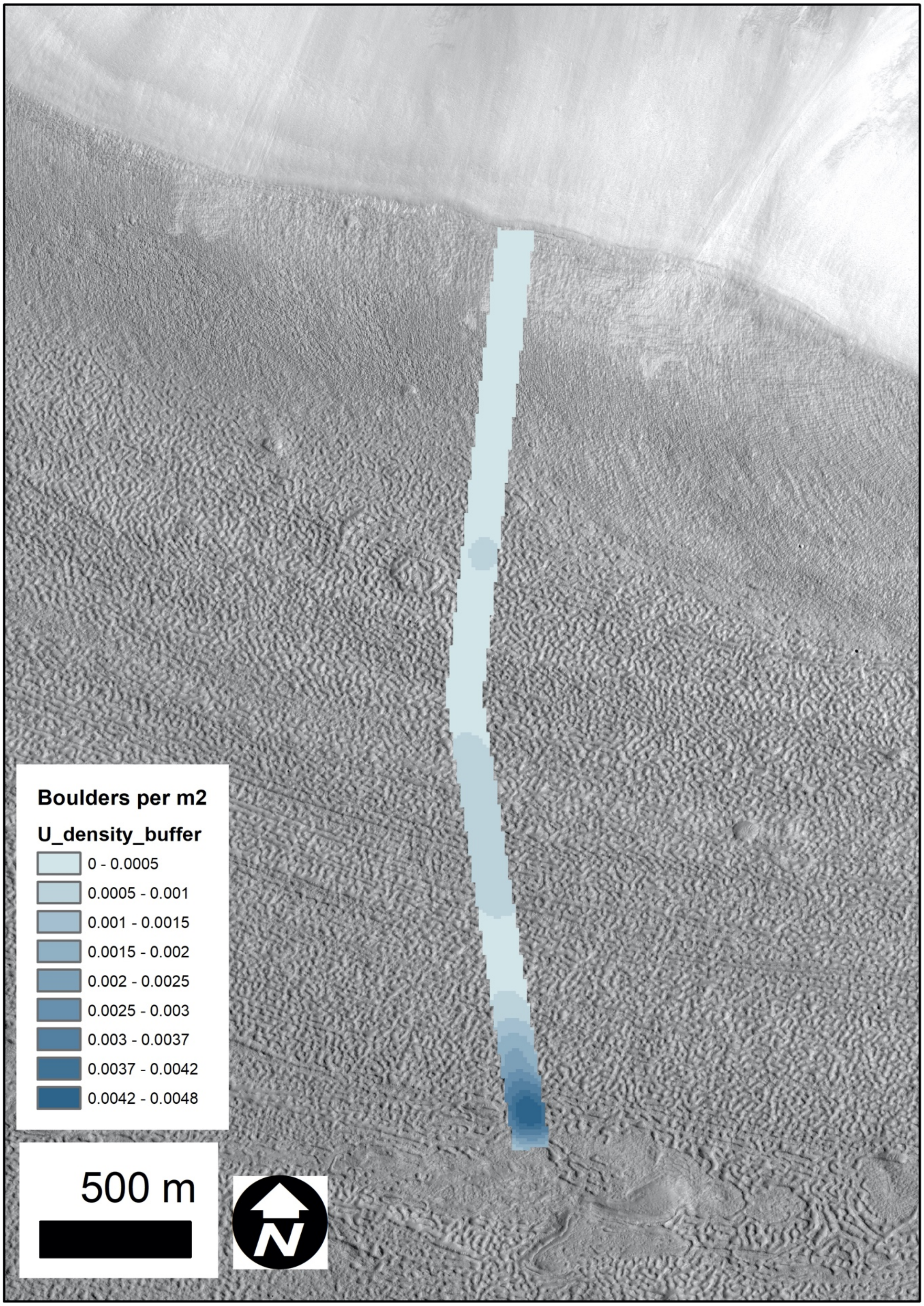
Boulders per m2
U_density_buffer
0 - 0.0005
0.0005 - 0.001
0.001 - 0.0015
0.0015 - 0.002
0.002 - 0.0025
0.0025 - 0.003
0.003 - 0.0037
0.0037 - 0.0042
0.0042 - 0.0048
500 m
N

Site U.

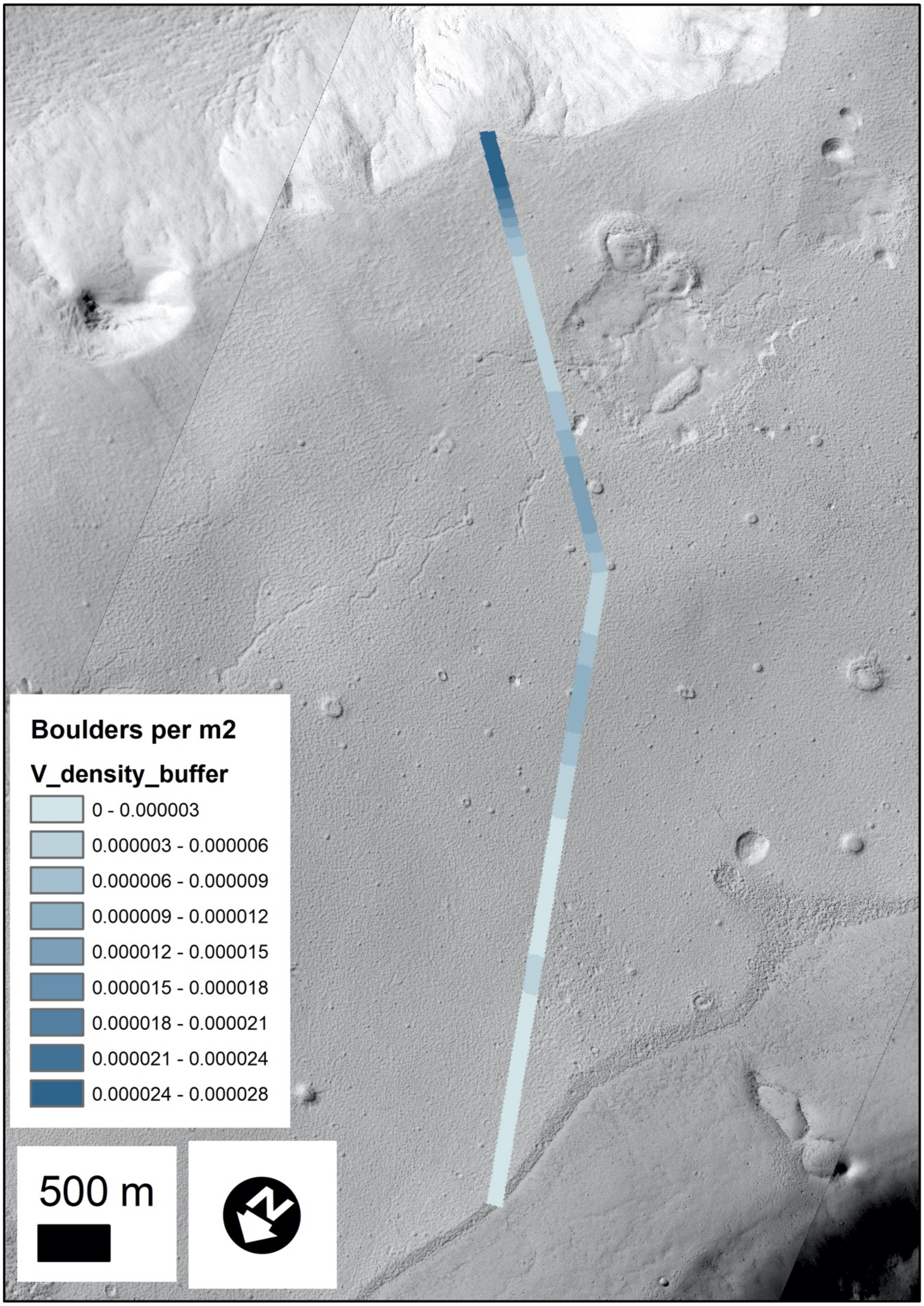

Boulders per m2
V_density_buffer
0 - 0.000003
0.000003 - 0.000006
0.000006 - 0.000009
0.000009 - 0.000012
0.000012 - 0.000015
0.000015 - 0.000018
0.000018 - 0.000021
0.000021 - 0.000024
0.000024 - 0.000028
500 m
N

Site V.

Site W.

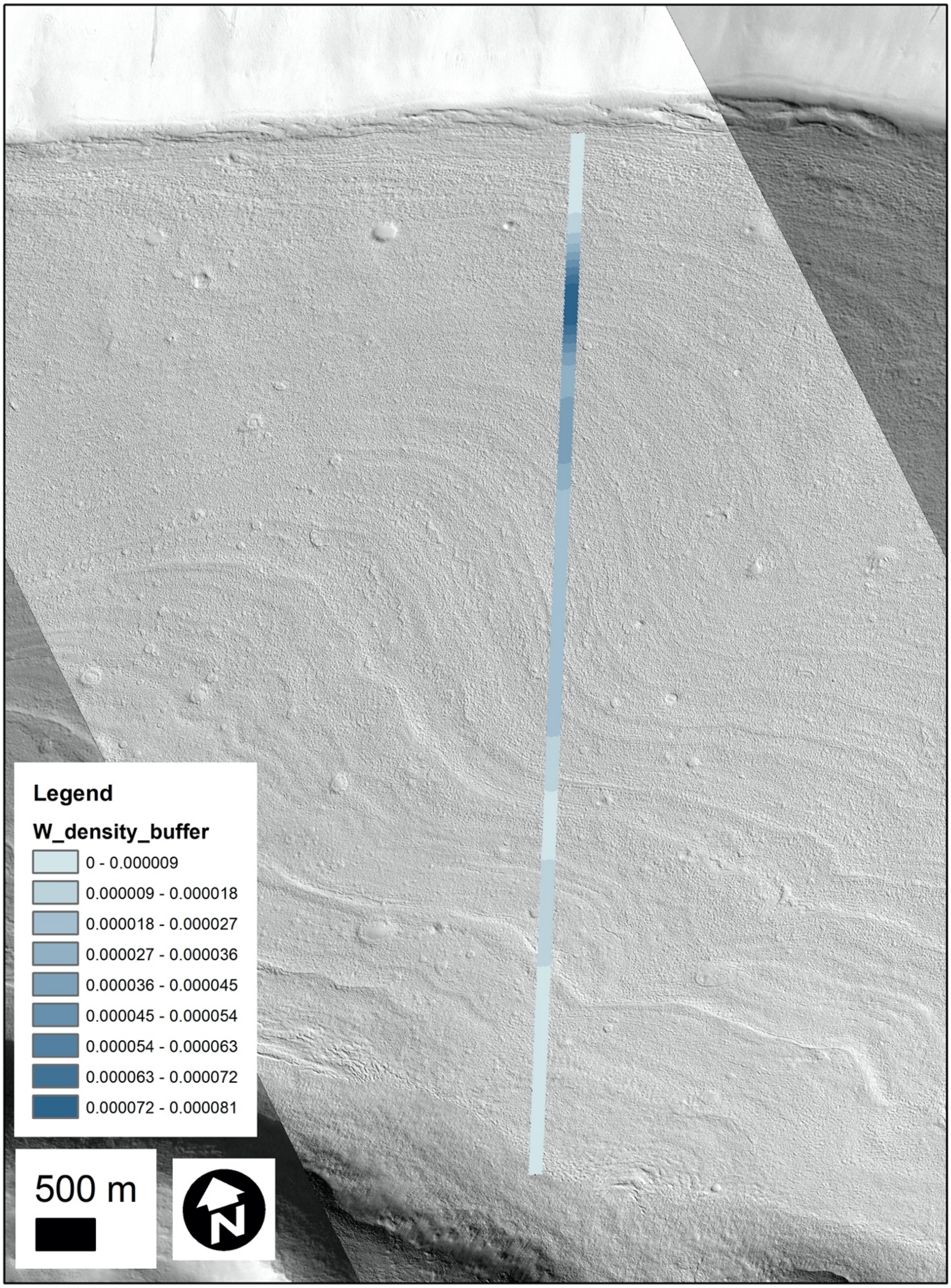

Legend
W_density_buffer
0 - 0.000009
0.000009 - 0.000018
0.000018 - 0.000027
0.000027 - 0.000036
0.000036 - 0.000045
0.000045 - 0.000054
0.000054 - 0.000063
0.000063 - 0.000072
0.000072 - 0.000081
500 m
N

Site W.

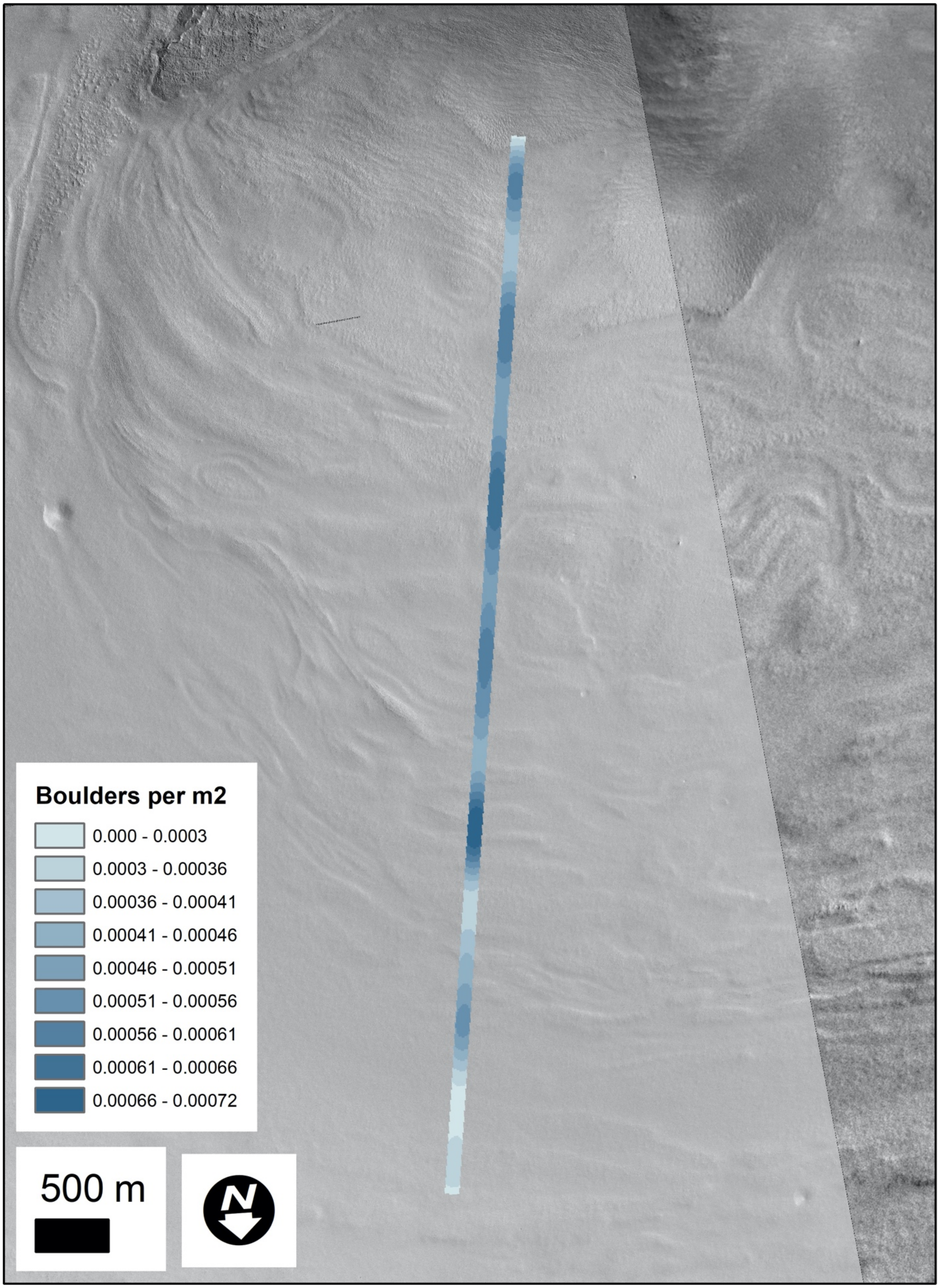
Boulders per m2
0.000 - 0.0003
0.0003 - 0.00036
0.00036 - 0.00041
0.00041 - 0.00046
0.00046 - 0.00051
0.00051 - 0.00056
0.00056 - 0.00061
0.00061 - 0.00066
0.00066 - 0.00072
500 m
N

Site X.

Site Y.

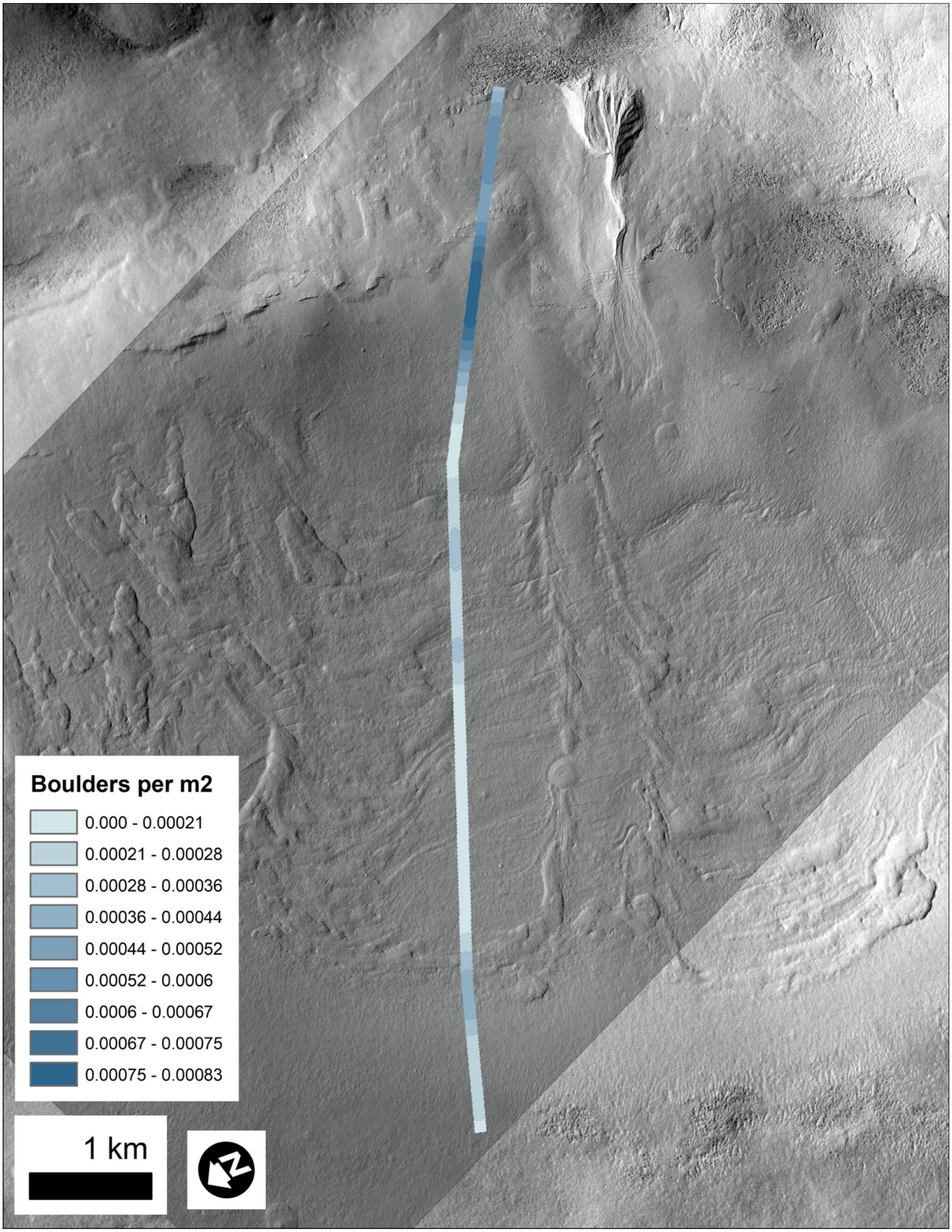

Boulders per m2
0.000 - 0.00021
0.00021 - 0.00028
0.00028 - 0.00036
0.00036 - 0.00044
0.00044 - 0.00052
0.00052 - 0.0006
0.0006 - 0.00067
0.00067 - 0.00075
0.00075 - 0.00083
1 km
N

Site Y.

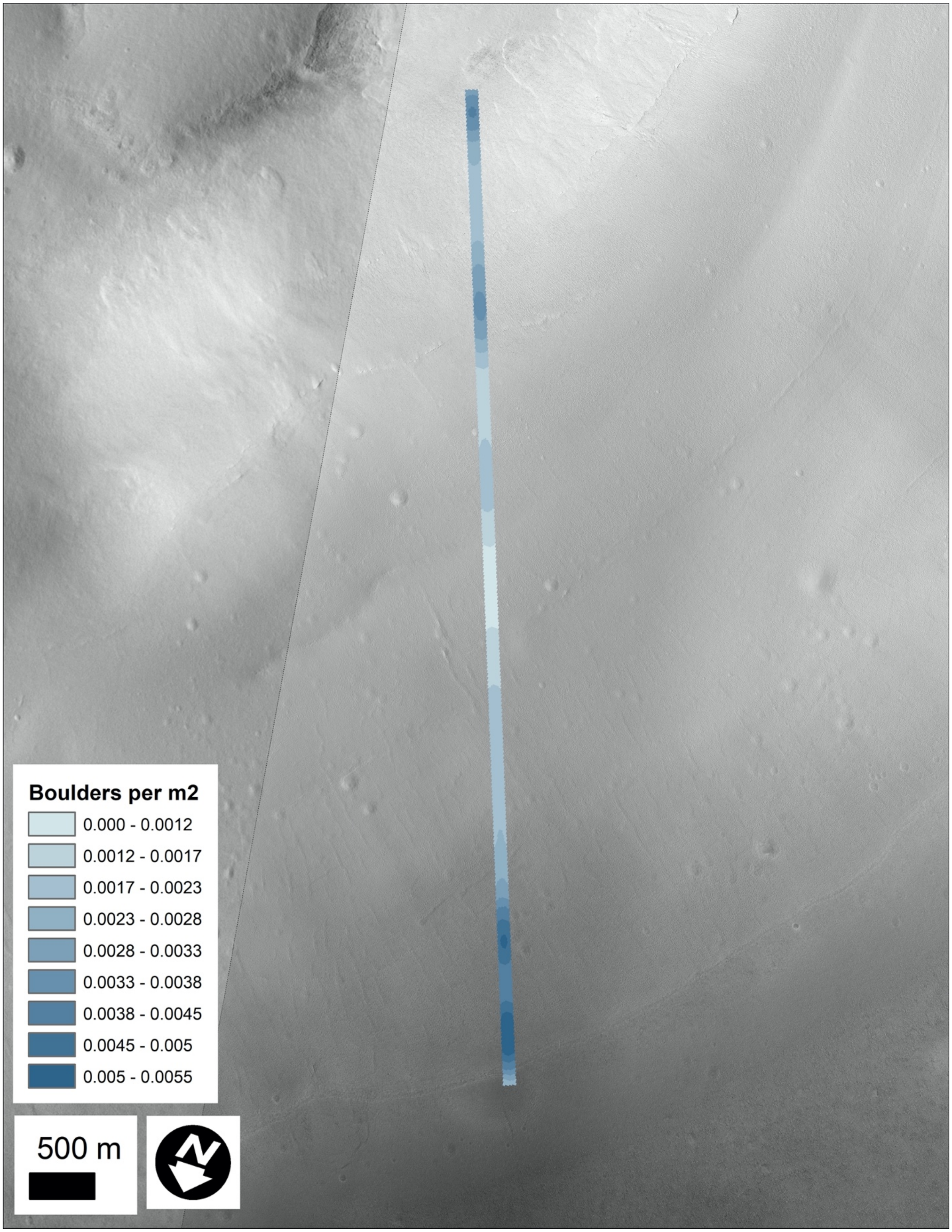
Boulders per m2
0.000 - 0.0012
0.0012 - 0.0017
0.0017 - 0.0023
0.0023 - 0.0028
0.0028 - 0.0033
0.0033 - 0.0038
0.0038 - 0.0045
0.0045 - 0.005
0.005 - 0.0055
500 m
N

Site Z.

LDA Site Boulder Density Plots

Plots showing number of boulders per 1% of distance down-LDA/glacier.

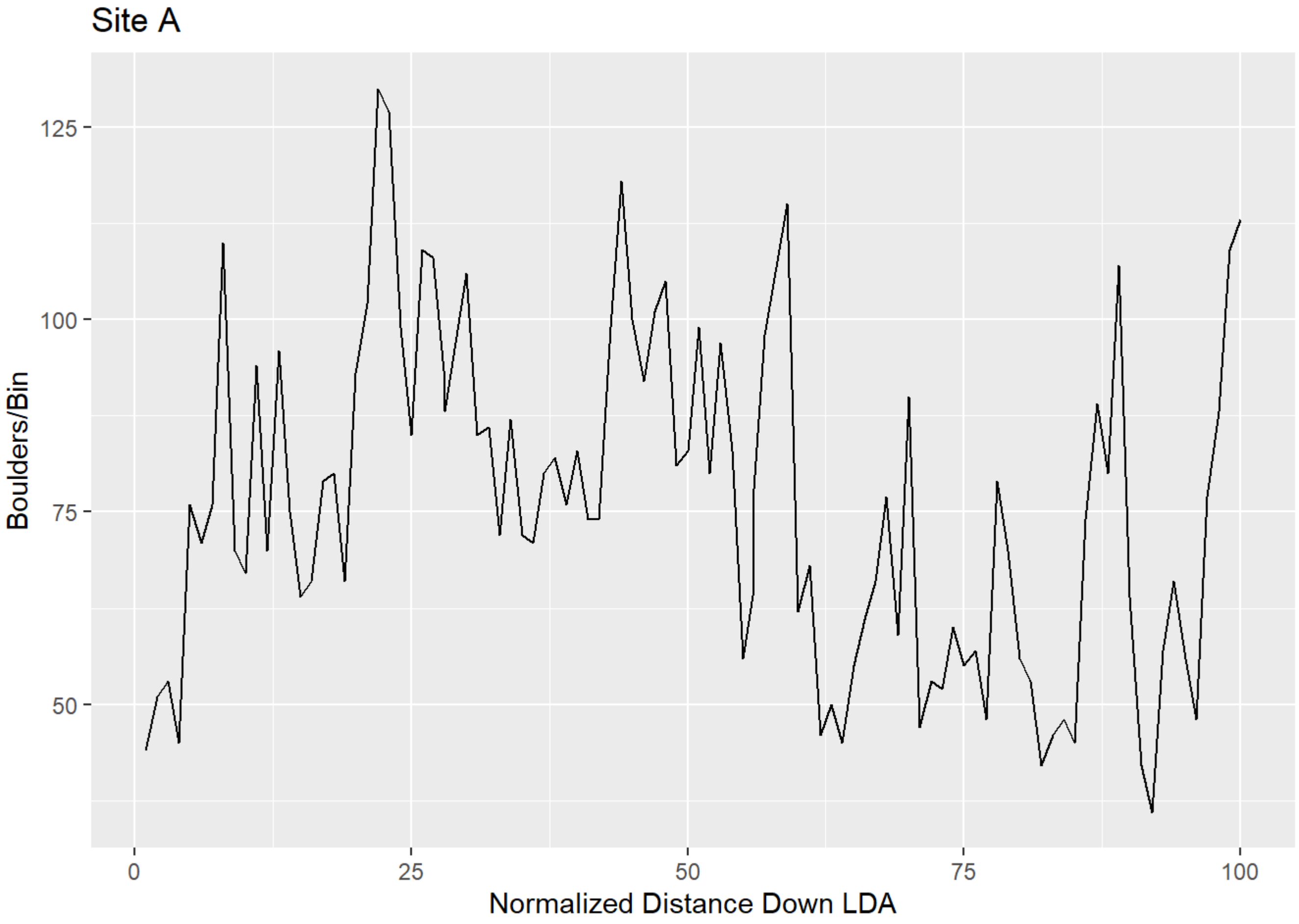

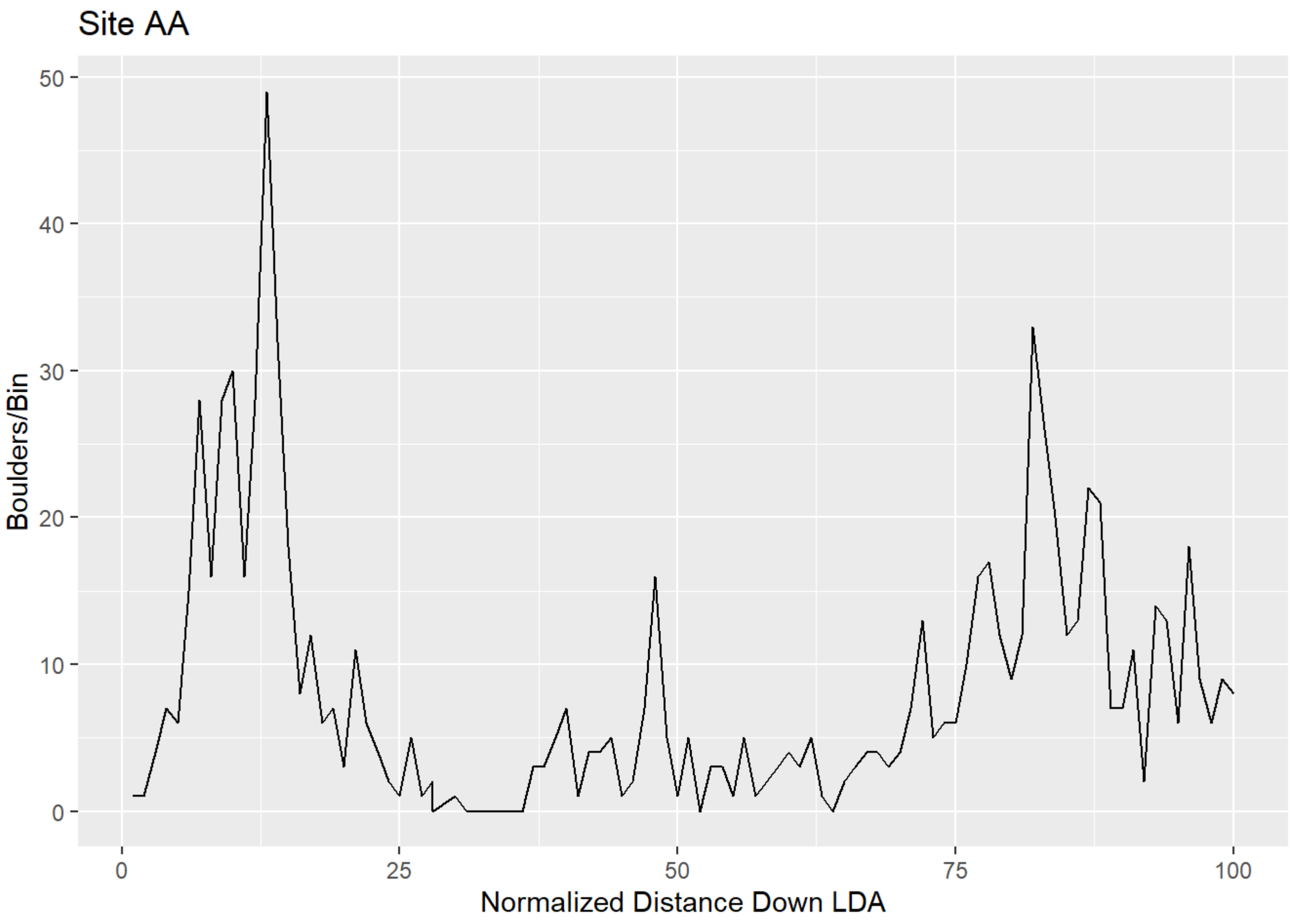
Site AA
Boulders/Bin
0
10
20
30
40
50
0
25
50
75
100
Normalized Distance Down LDA

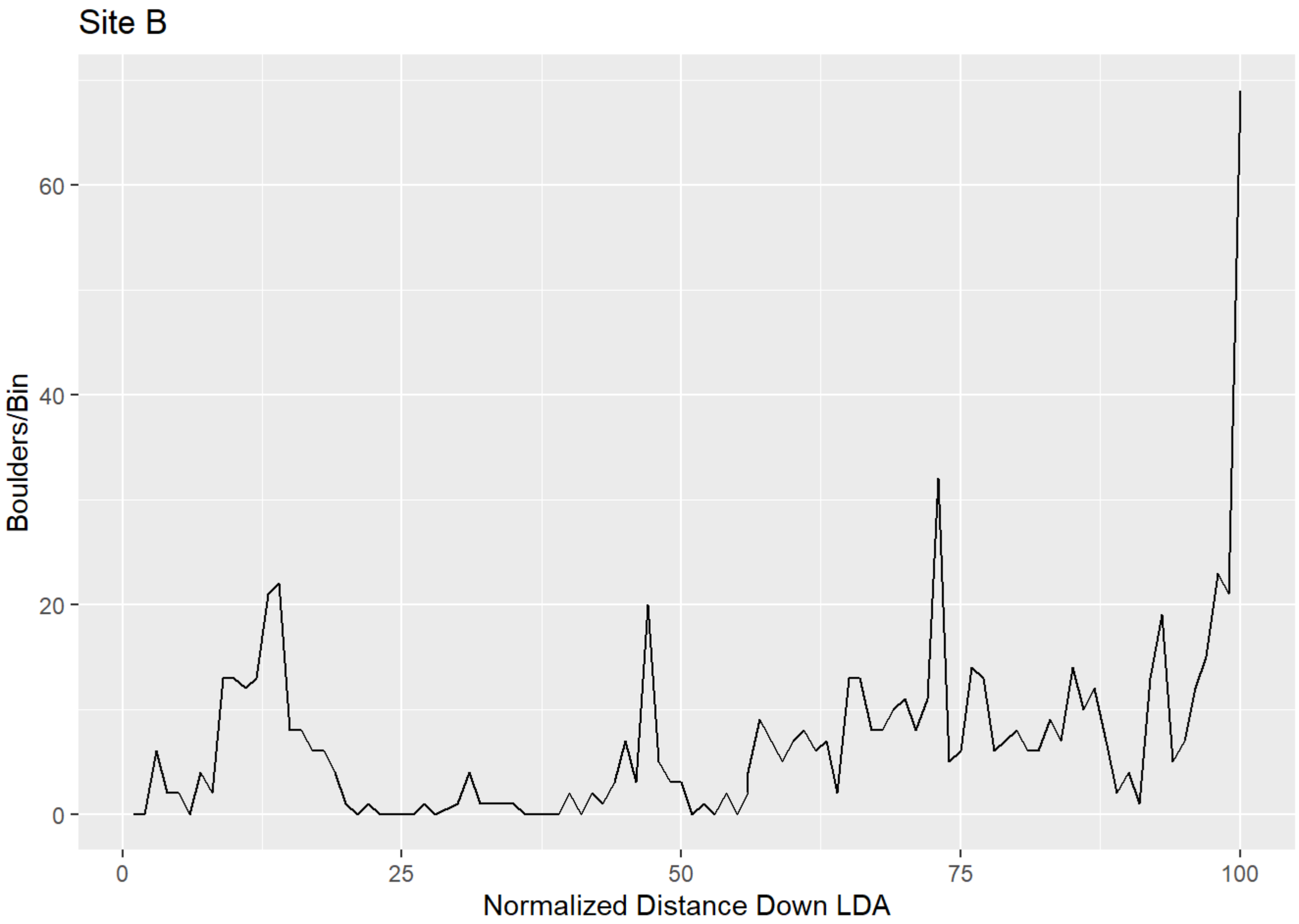
Site B
Boulders/Bin
0
20
40
60
0
25
50
75
100
Normalized Distance Down LDA

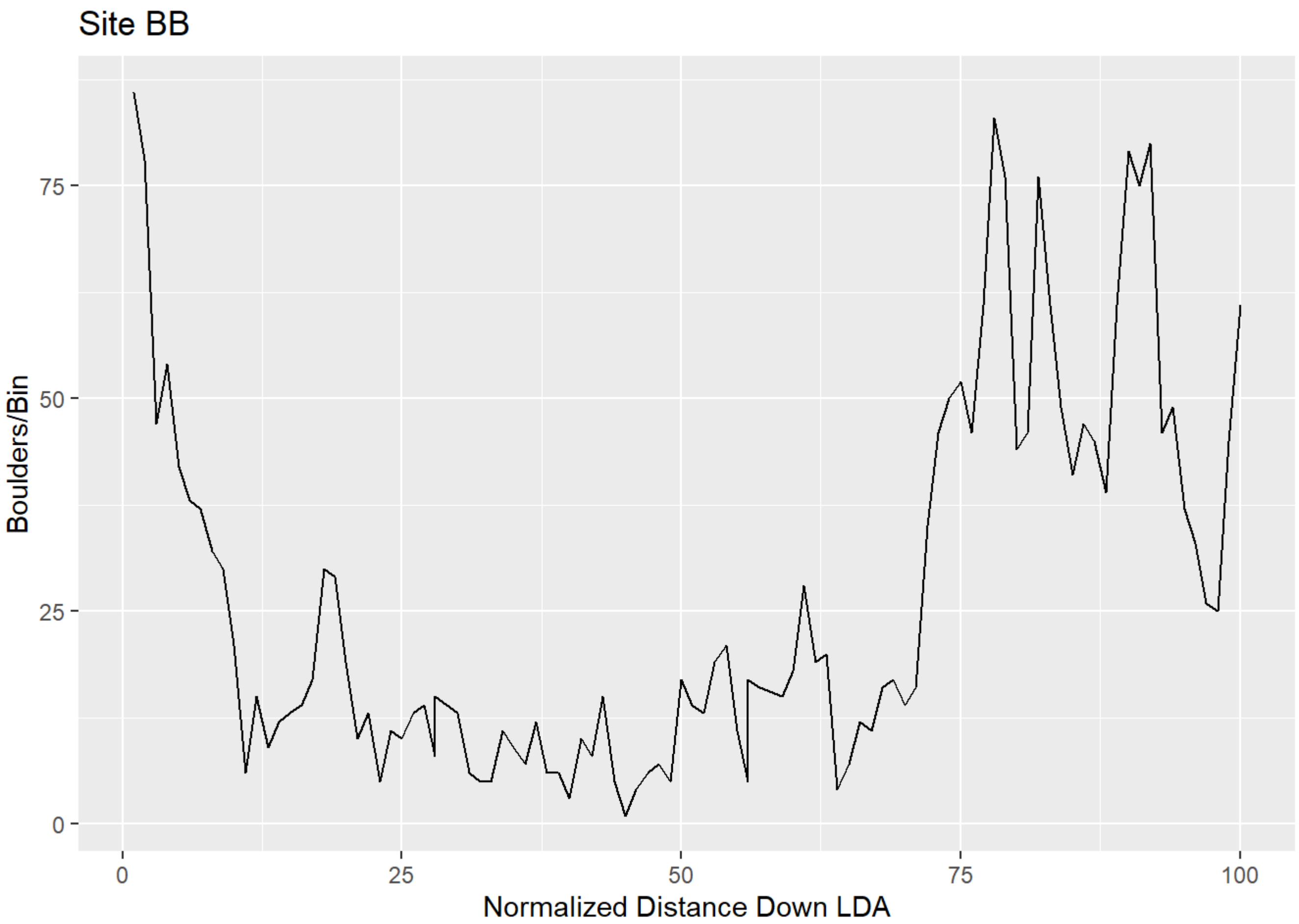
Site BB
Boulders/Bin
75
50
25
0
0
25
50
75
100
Normalized Distance Down LDA

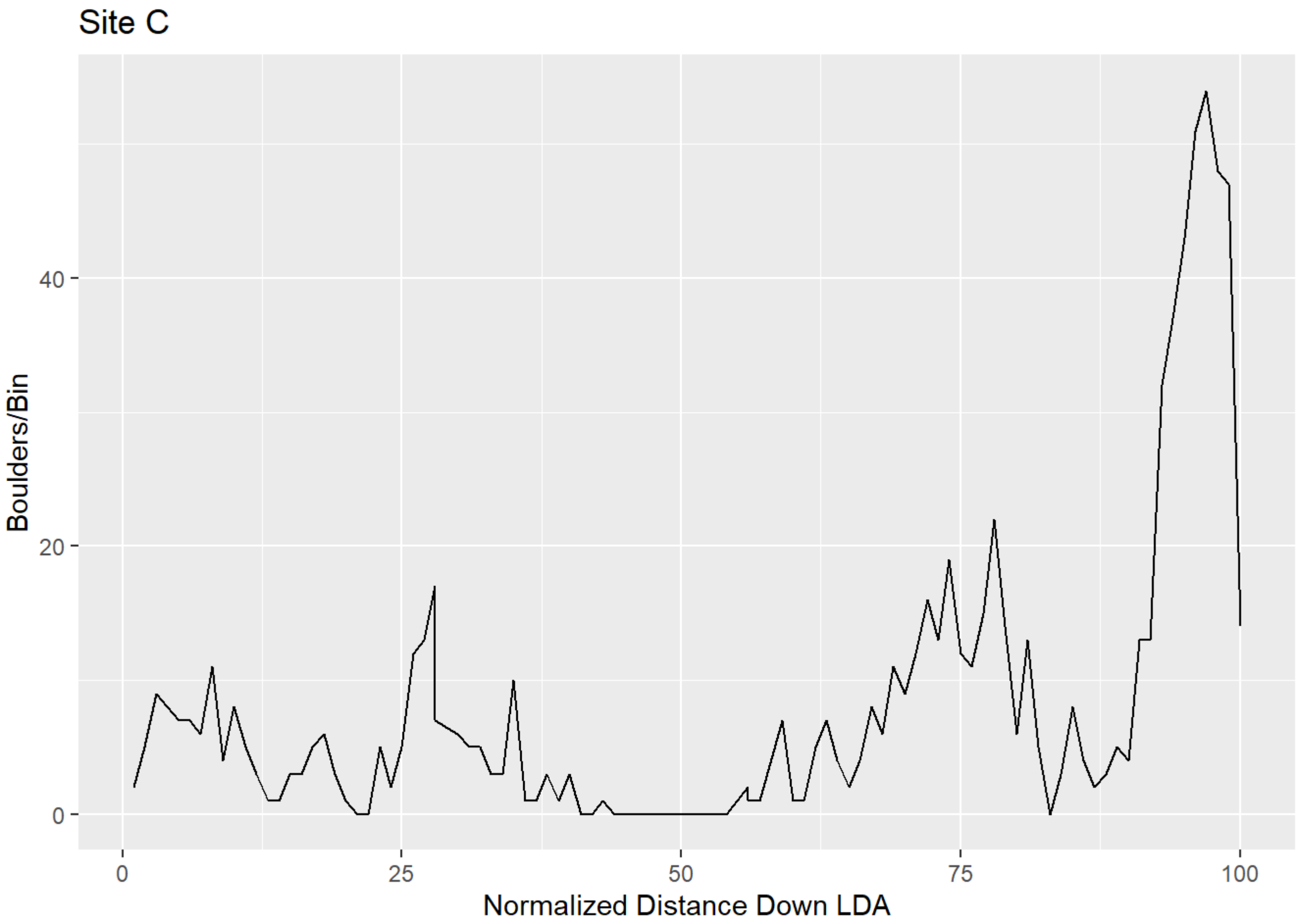
Site C
Boulders/Bin
40
20
0
0
25
50
75
100
Normalized Distance Down LDA

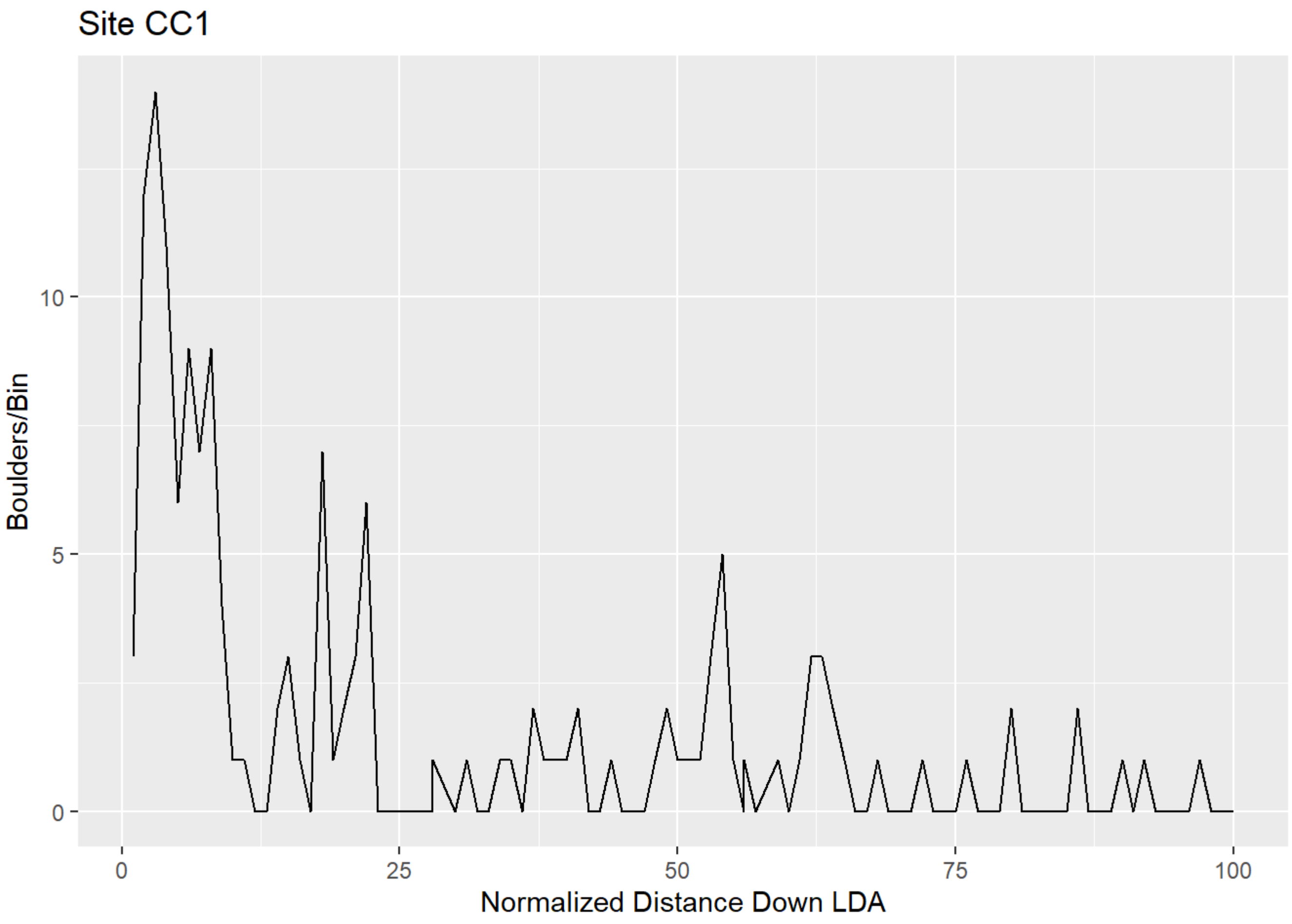

Site CC1
Boulders/Bin
0
5
10
0
25
50
75
100
Normalized Distance Down LDA

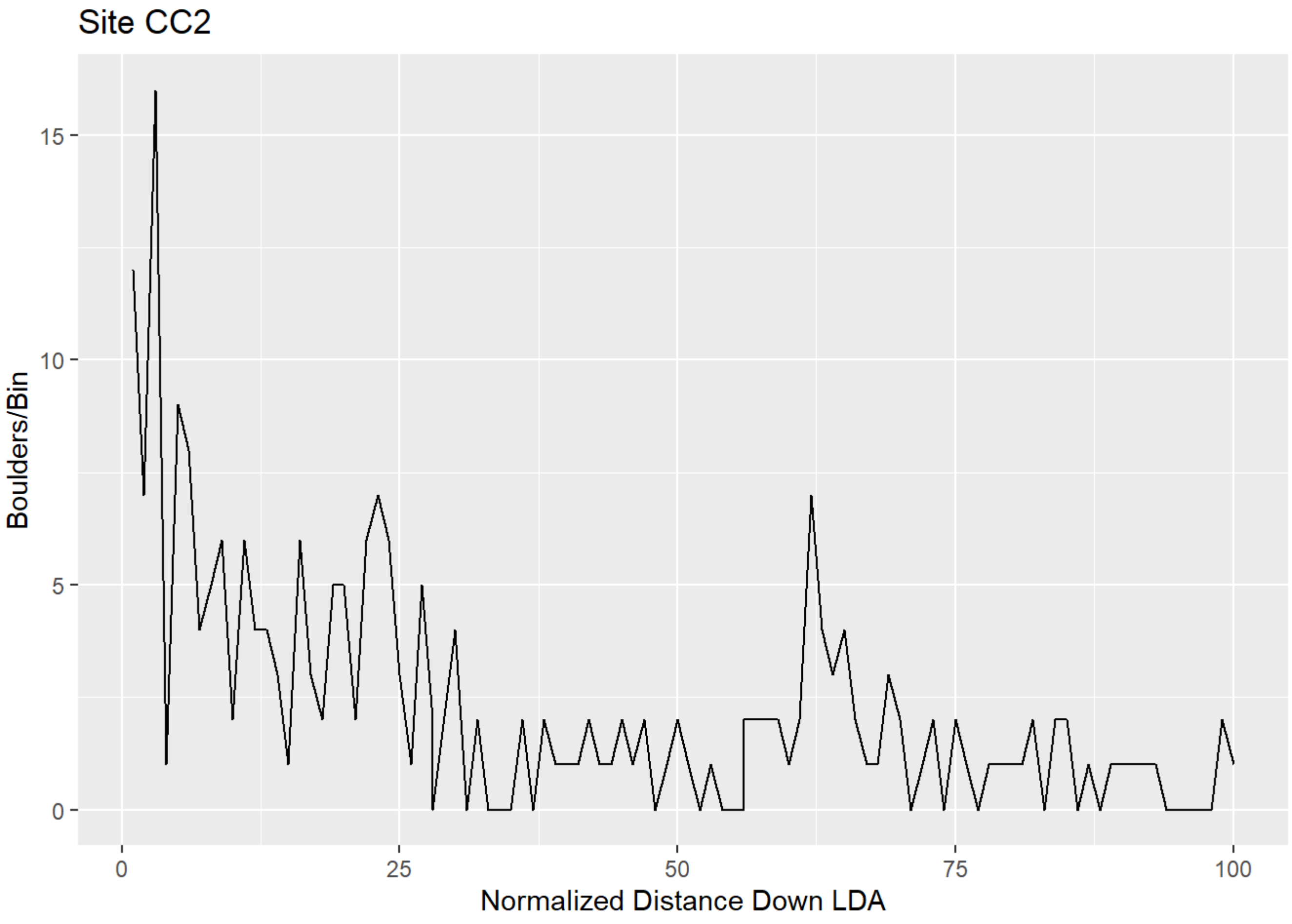

Site CC2
Boulders/Bin
0
5
10
15
0
25
50
75
100
Normalized Distance Down LDA

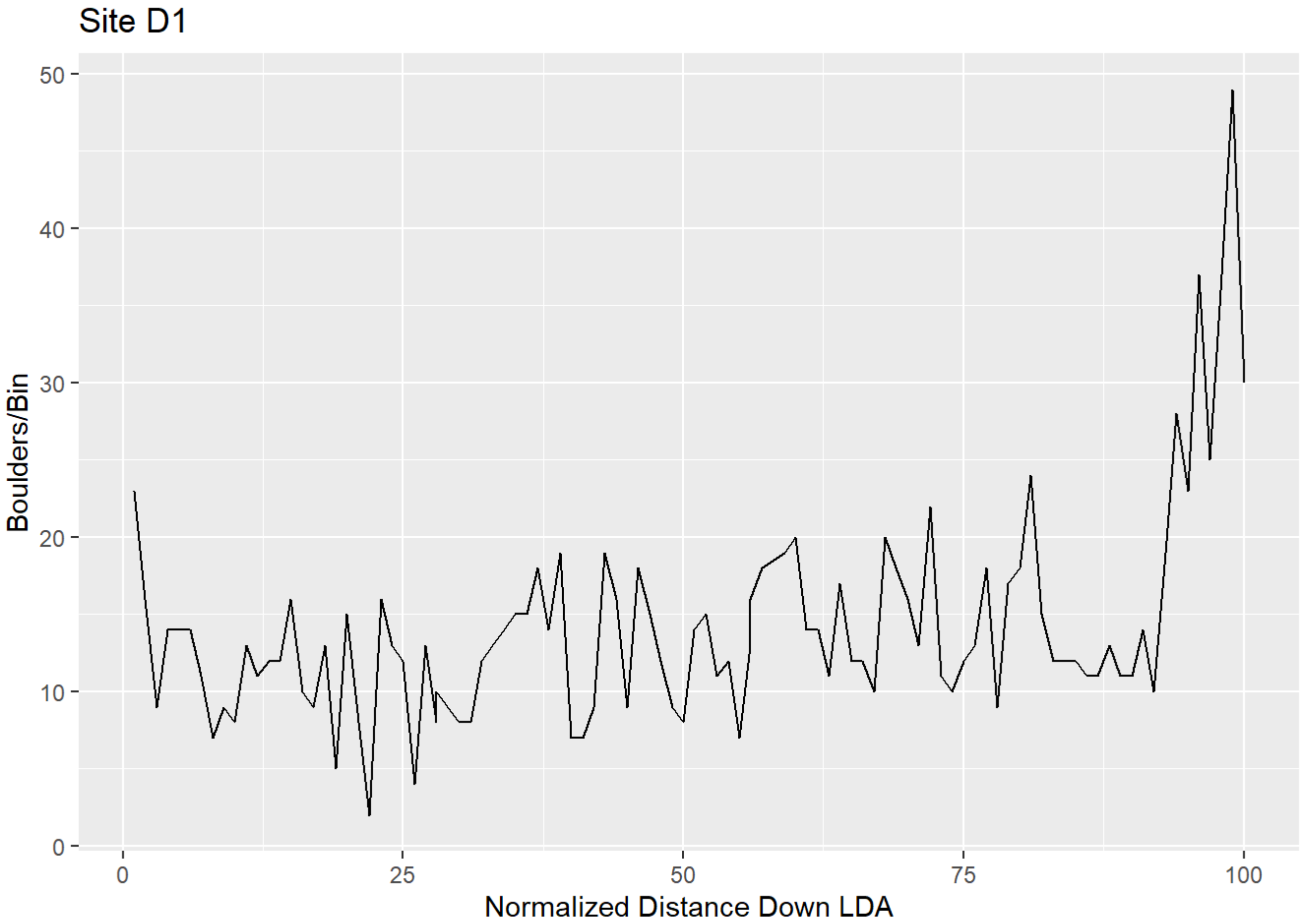
Site D1
Boulders/Bin
0
10
20
30
40
50
0
25
50
75
100
Normalized Distance Down LDA

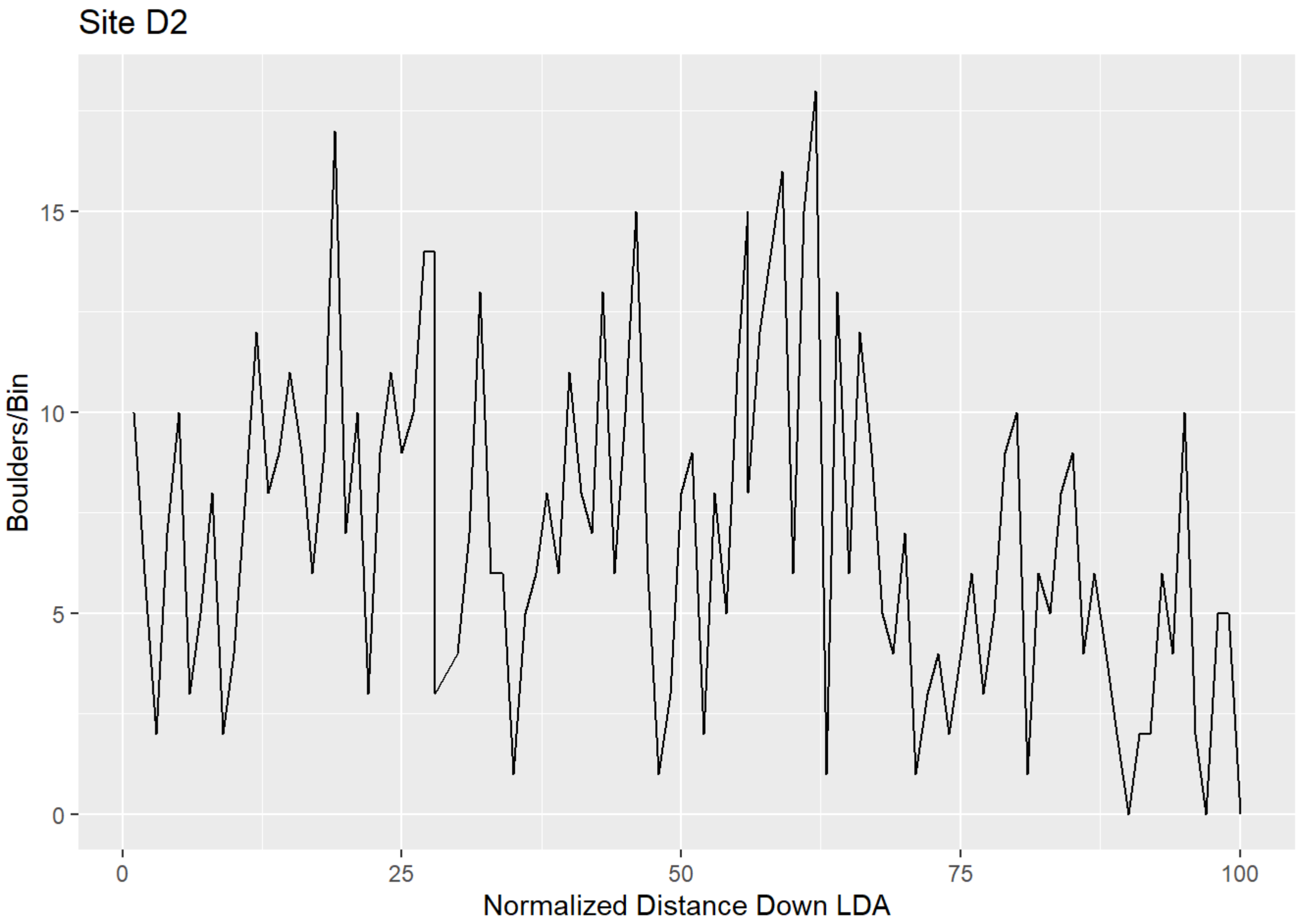
Site D2
Boulders/Bin
0
5
10
15
0
25
50
75
100
Normalized Distance Down LDA

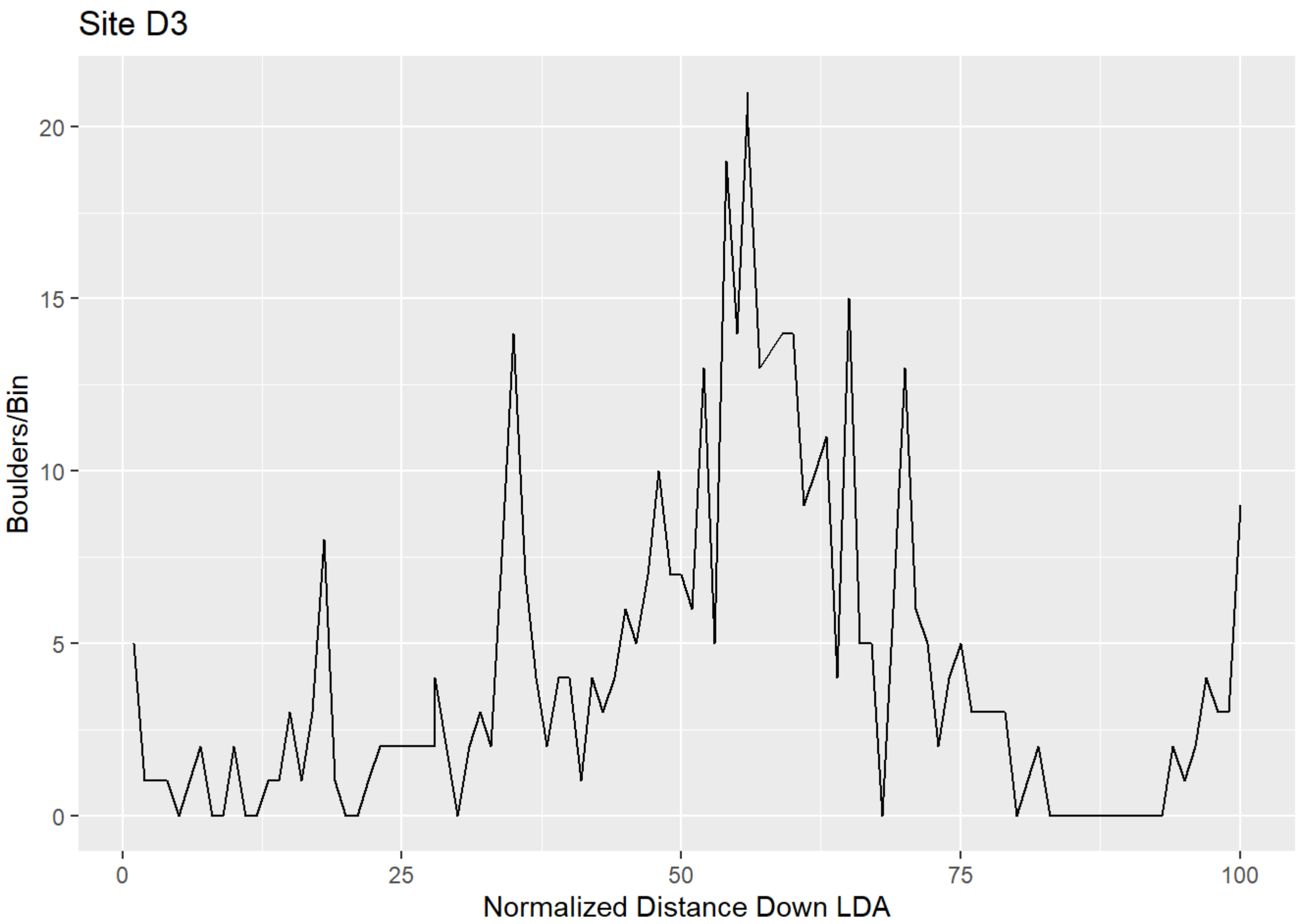

Site D3
Boulders/Bin
Normalized Distance Down LDA
0
5
10
15
20
0
25
50
75
100

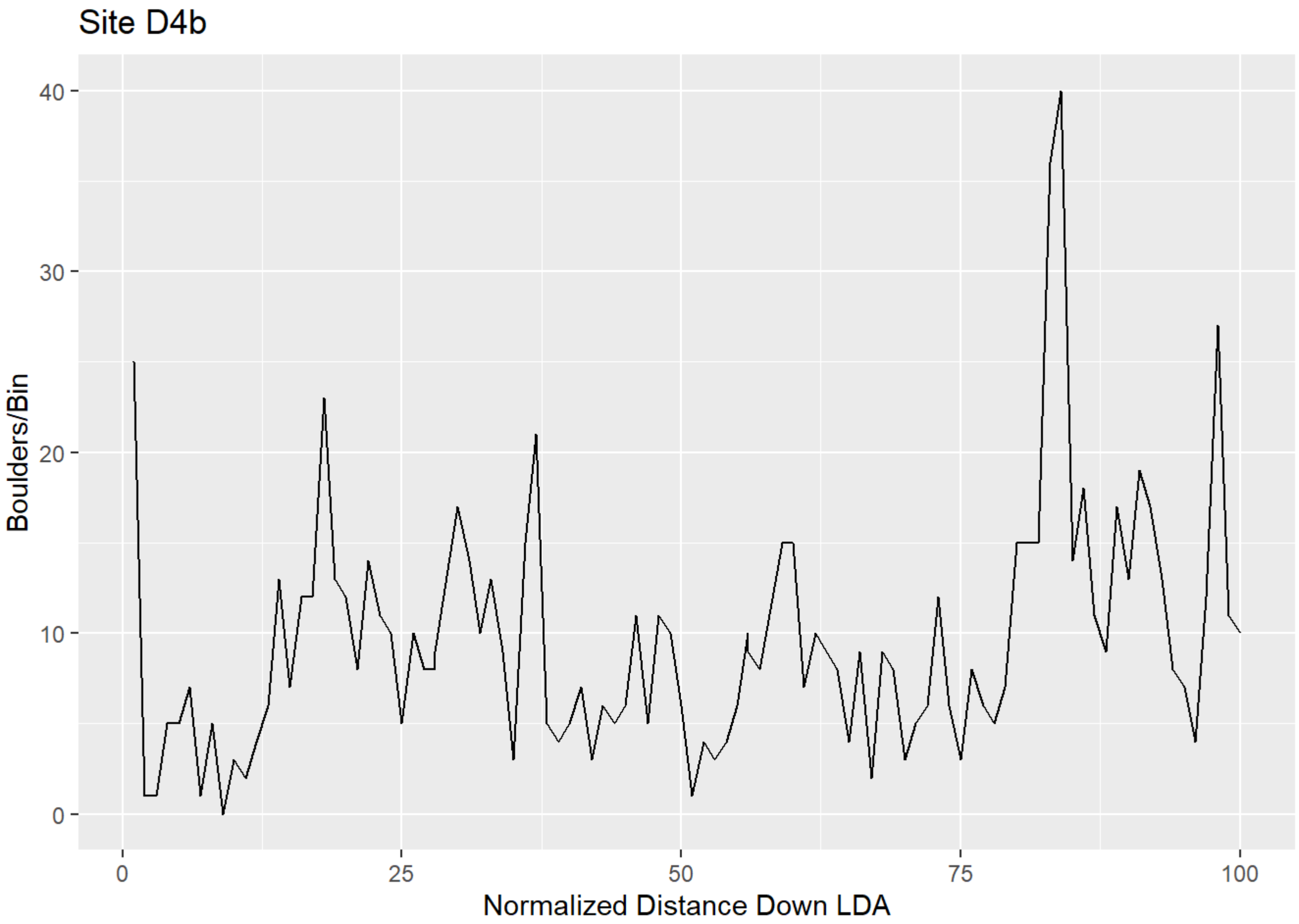

Site D4b
Boulders/Bin
Normalized Distance Down LDA
0
10
20
30
40
0
25
50
75
100

Site D5

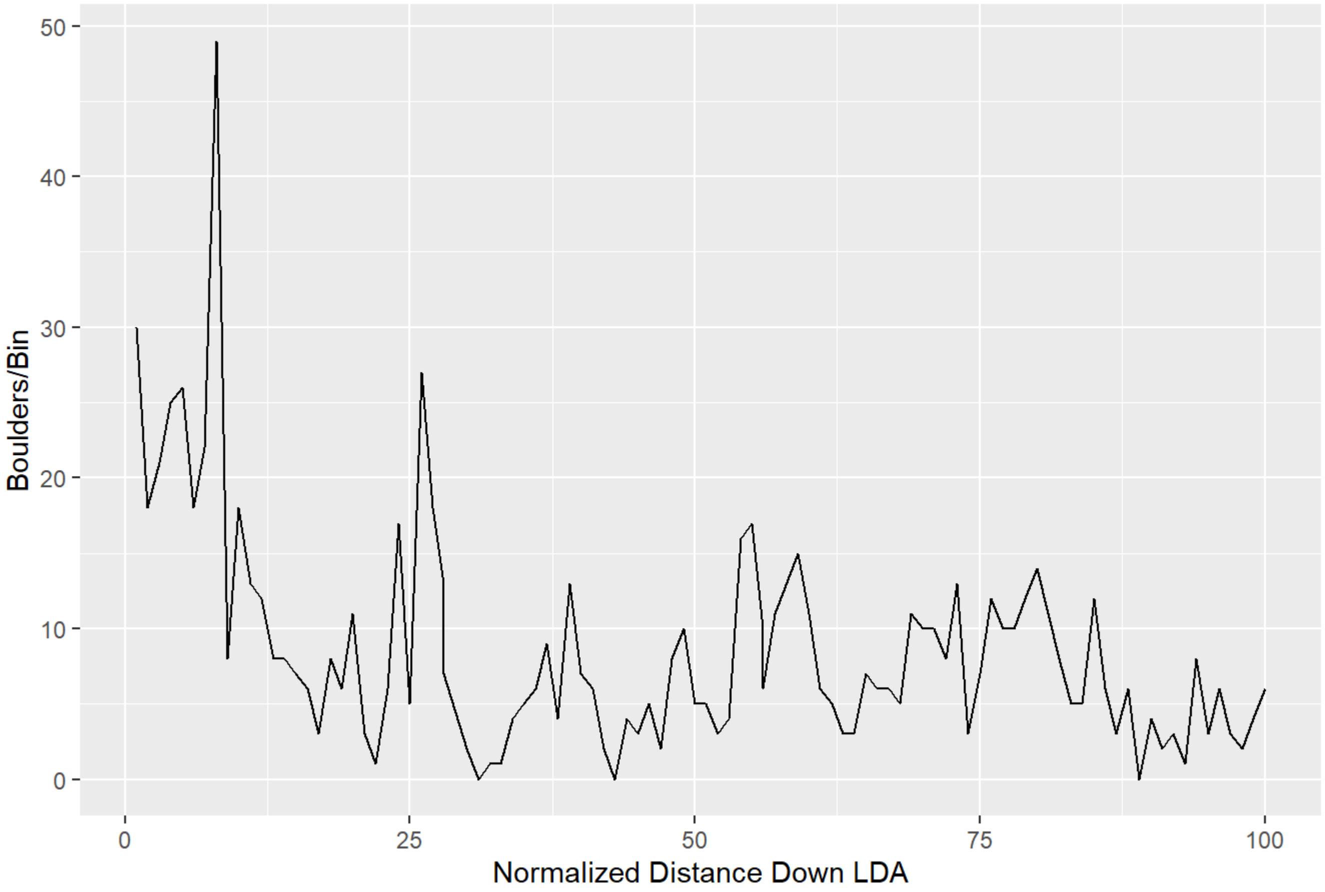

Site DD1

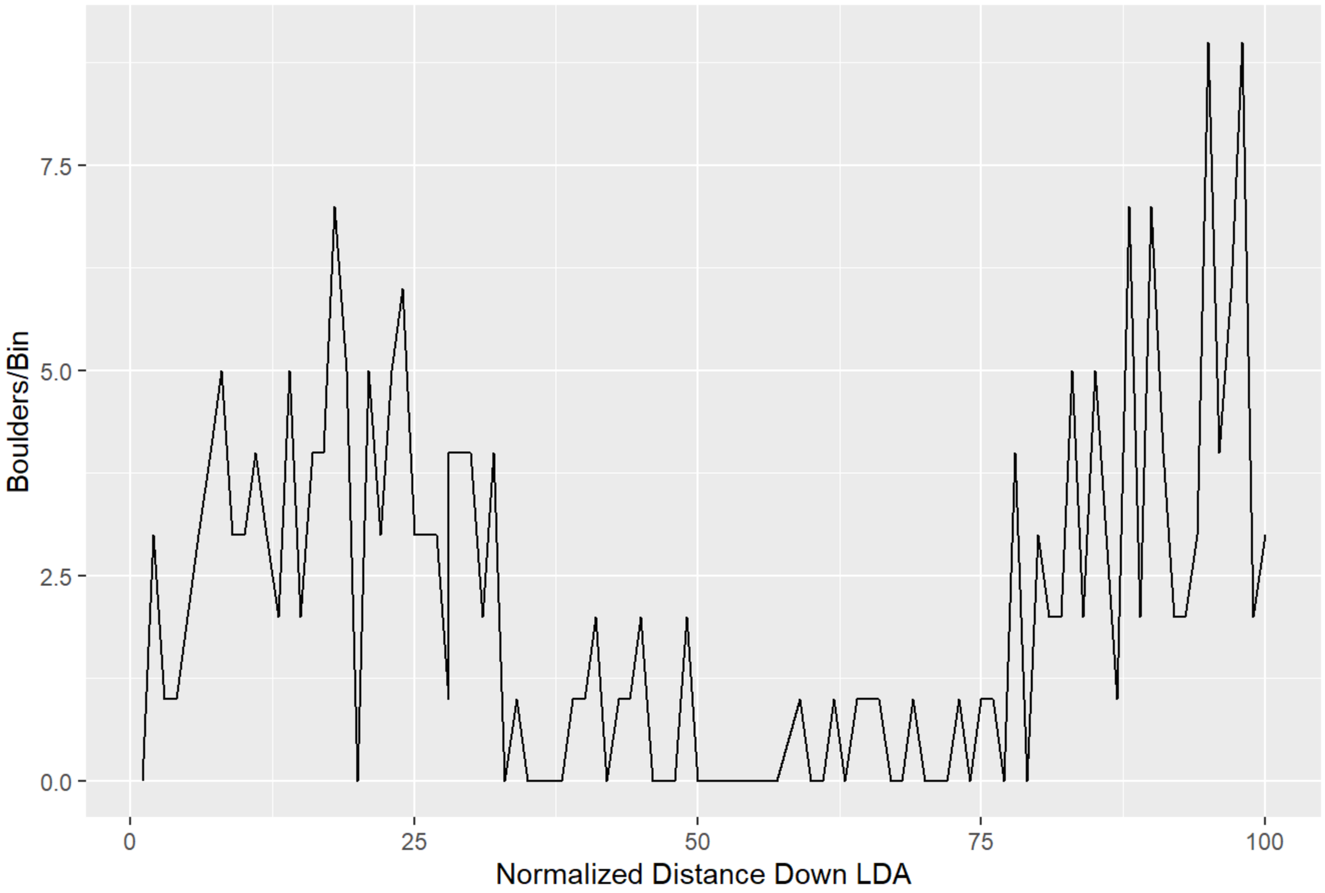

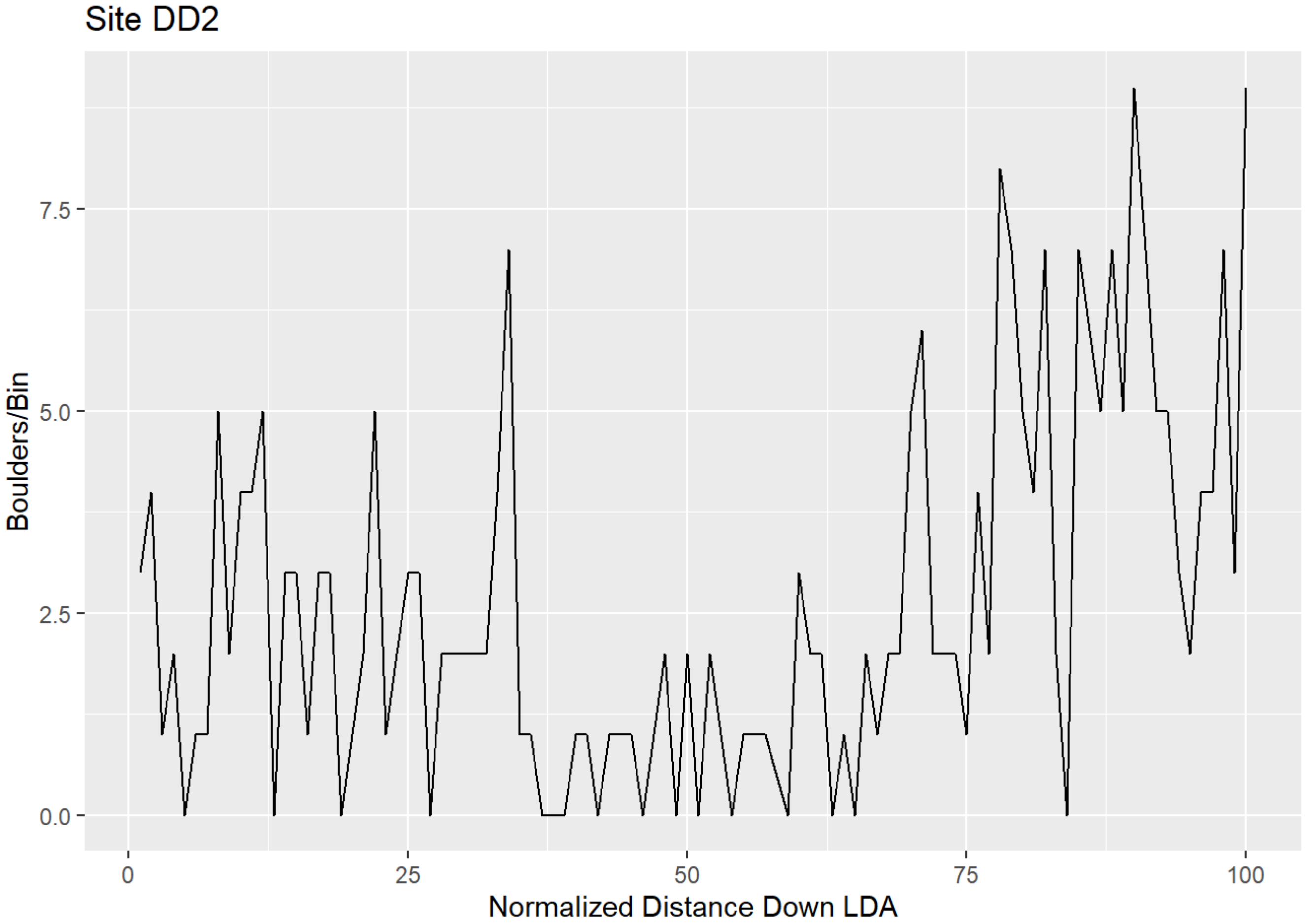
Site DD2
Boulders/Bin
0.0
2.5
5.0
7.5
0
25
50
75
100
Normalized Distance Down LDA

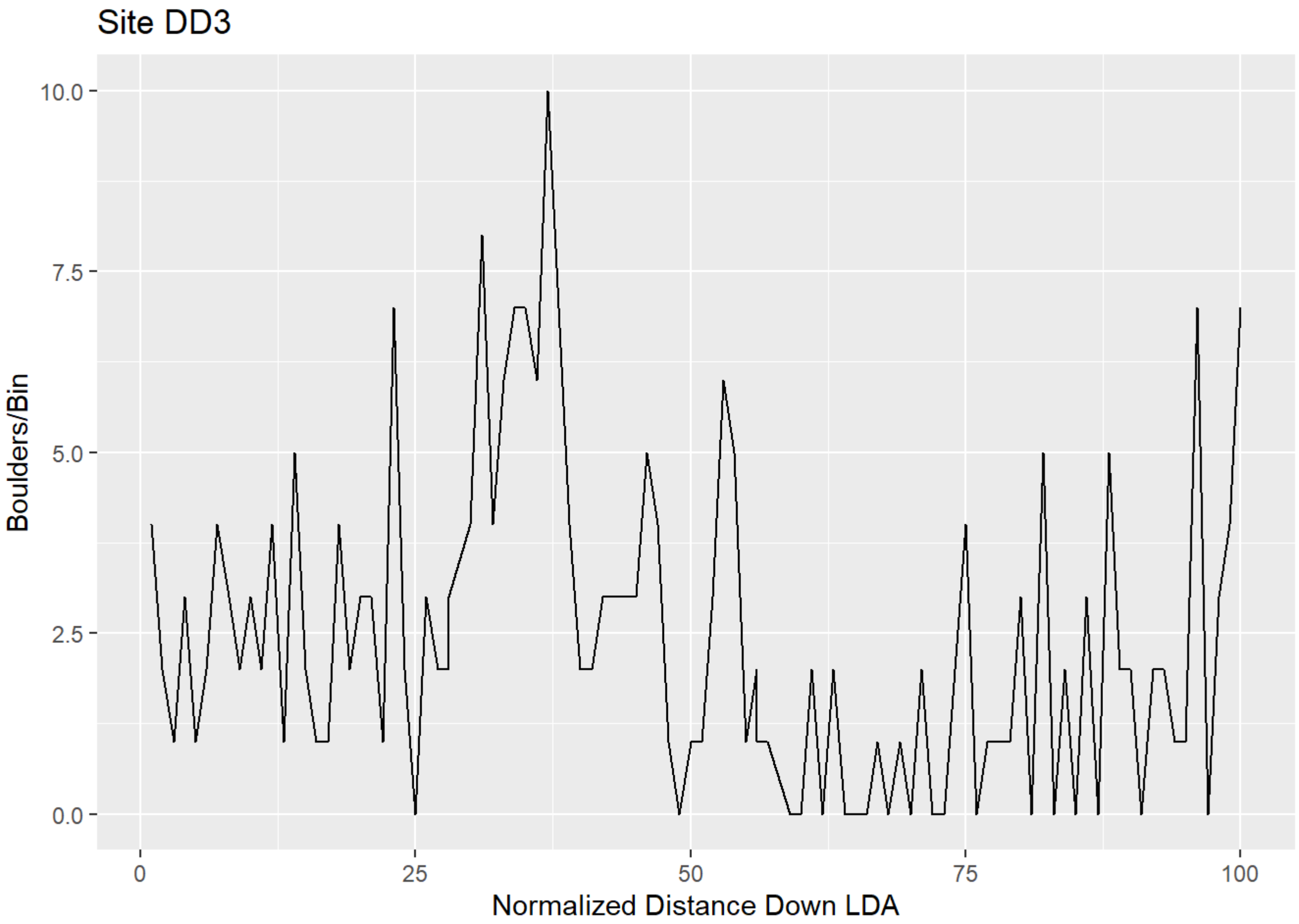
Site DD3
Boulders/Bin
0.0
2.5
5.0
7.5
10.0
0
25
50
75
100
Normalized Distance Down LDA

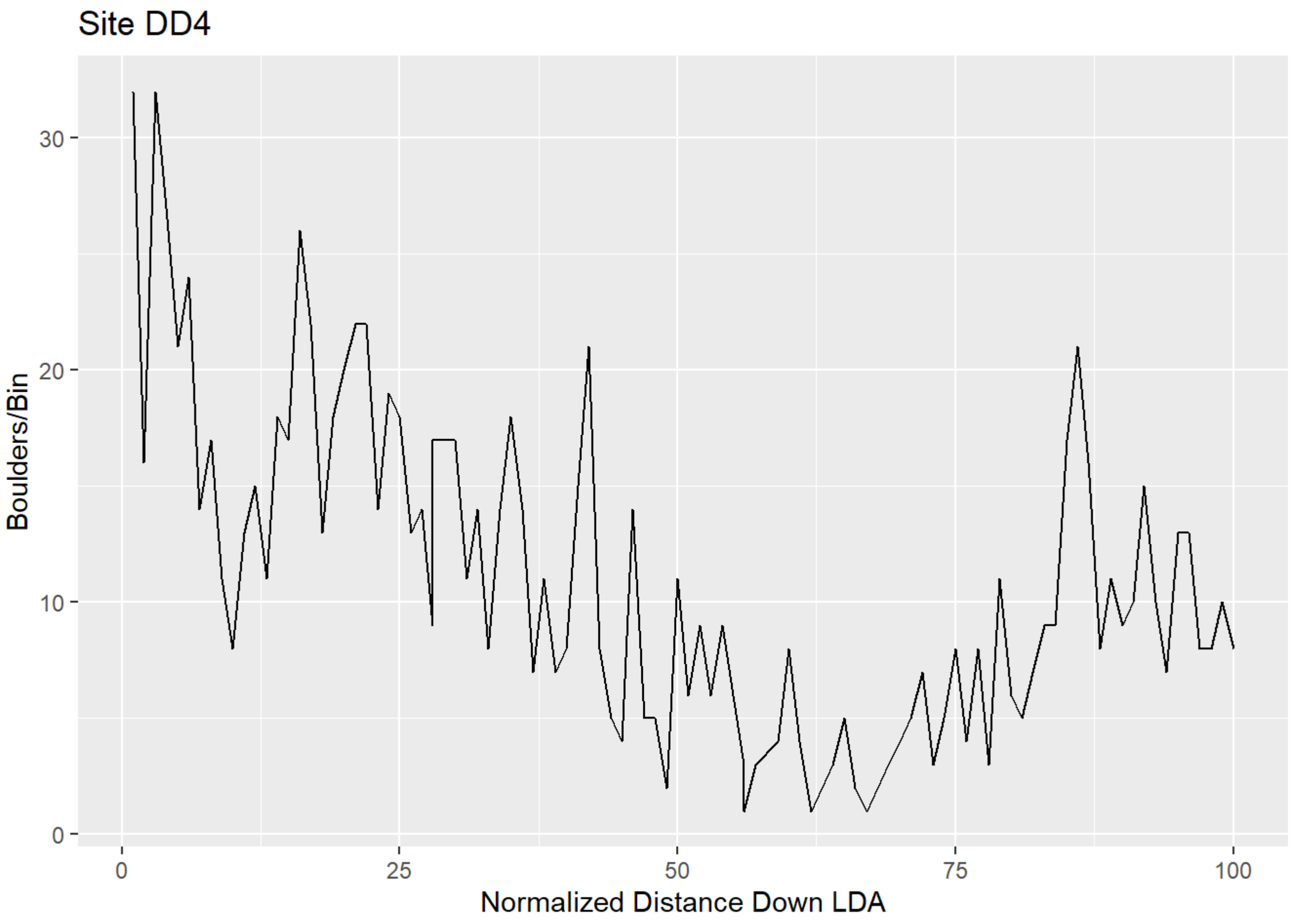
Site DD4
Boulders/Bin
30
20
10
0
0
25
50
75
100
Normalized Distance Down LDA

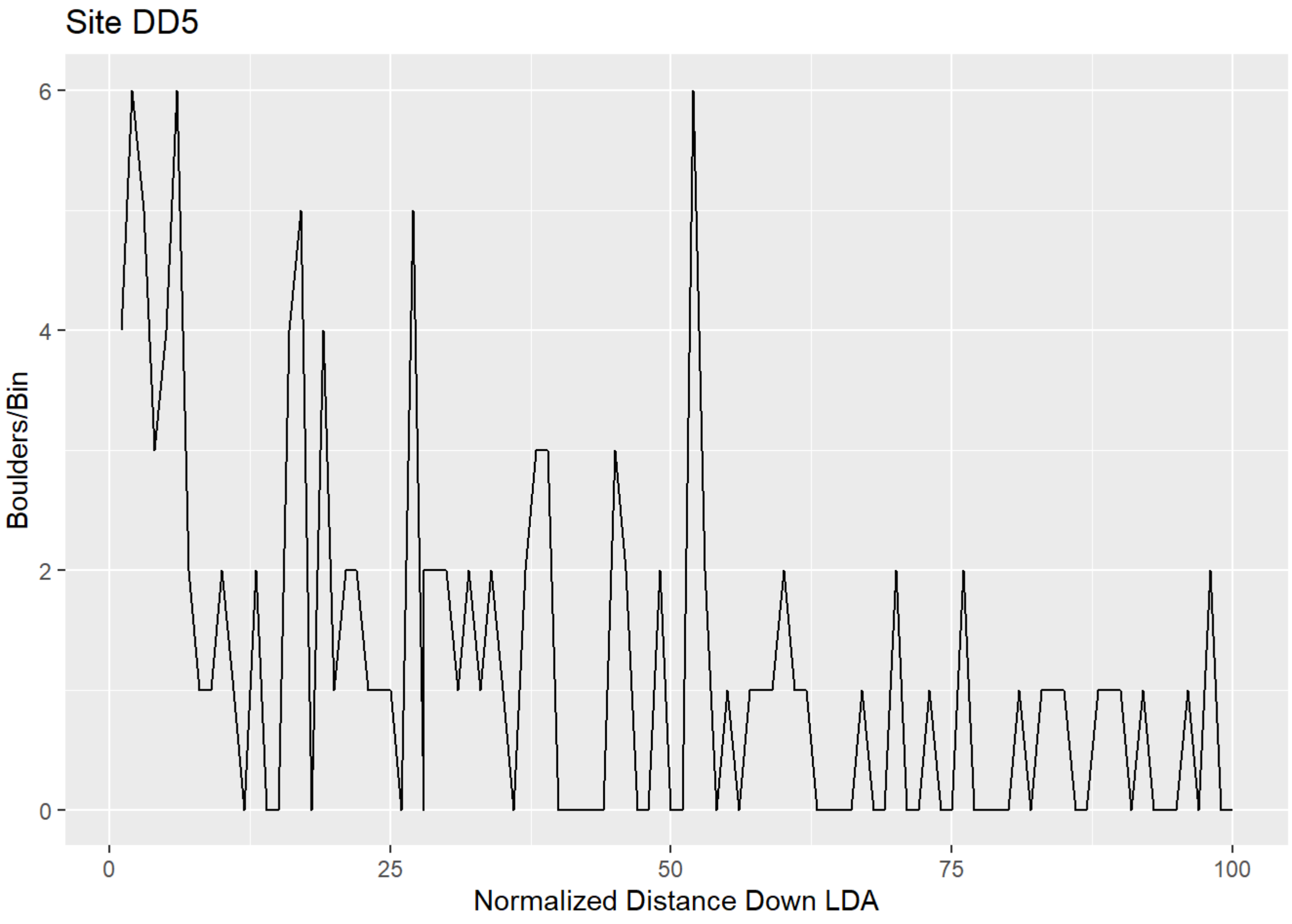
Site DD5
Boulders/Bin
6
4
2
0
0
25
50
75
100
Normalized Distance Down LDA

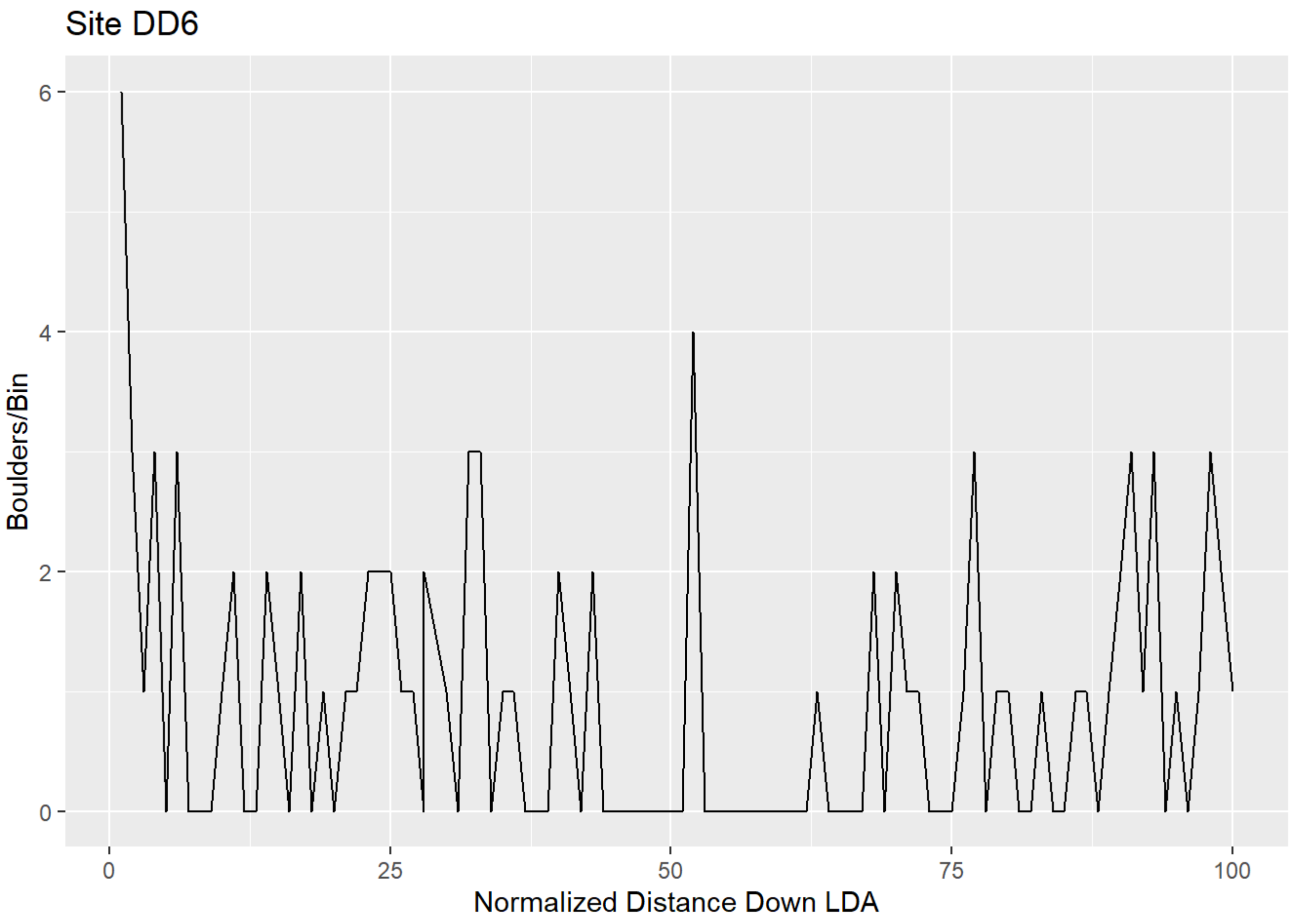

Site DD6
Boulders/Bin
0
2
4
6
0
25
50
75
100
Normalized Distance Down LDA

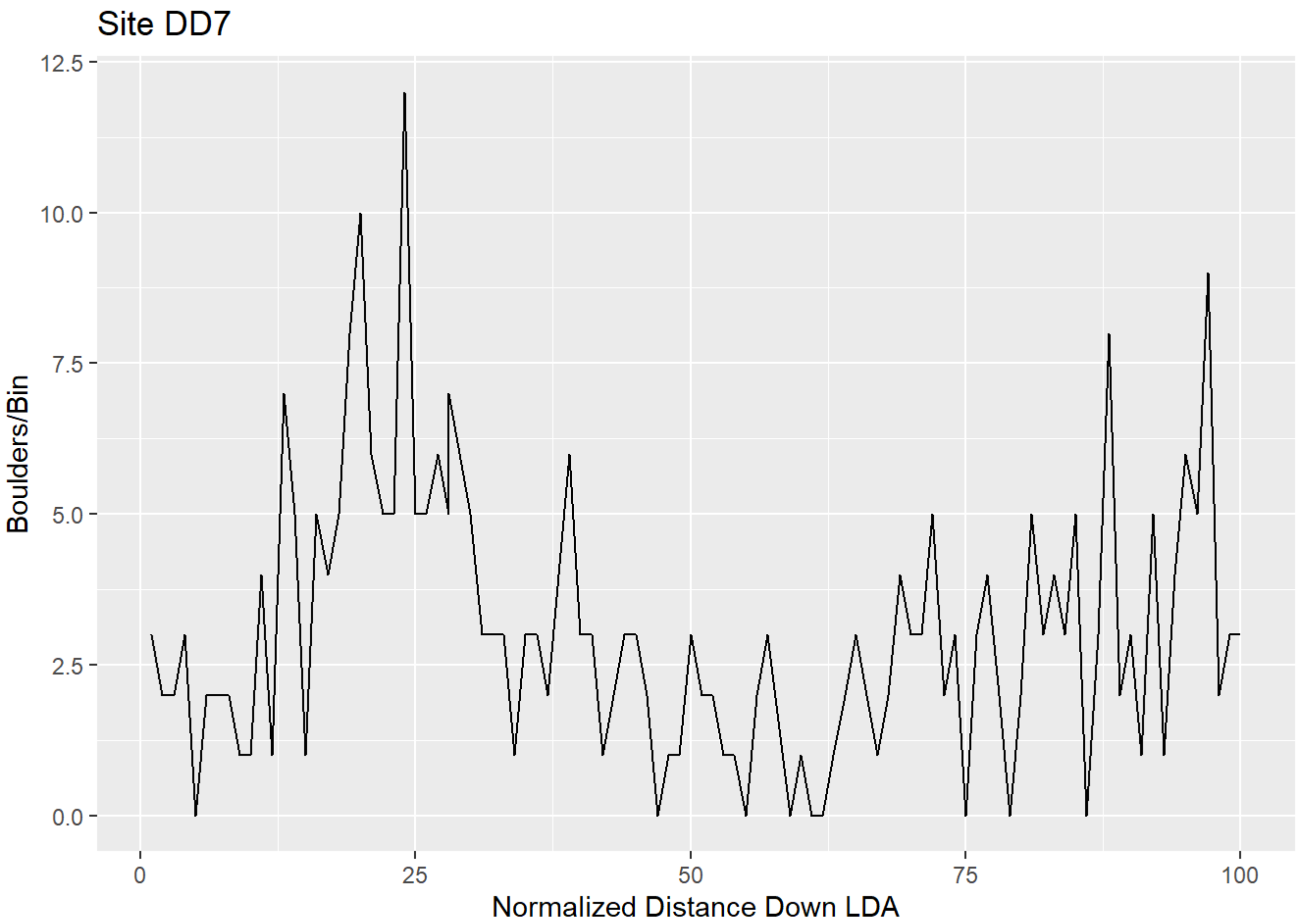
Site DD7
12.5
10.0
7.5
5.0
2.5
0.0
Boulders/Bin
0
25
50
75
100
Normalized Distance Down LDA

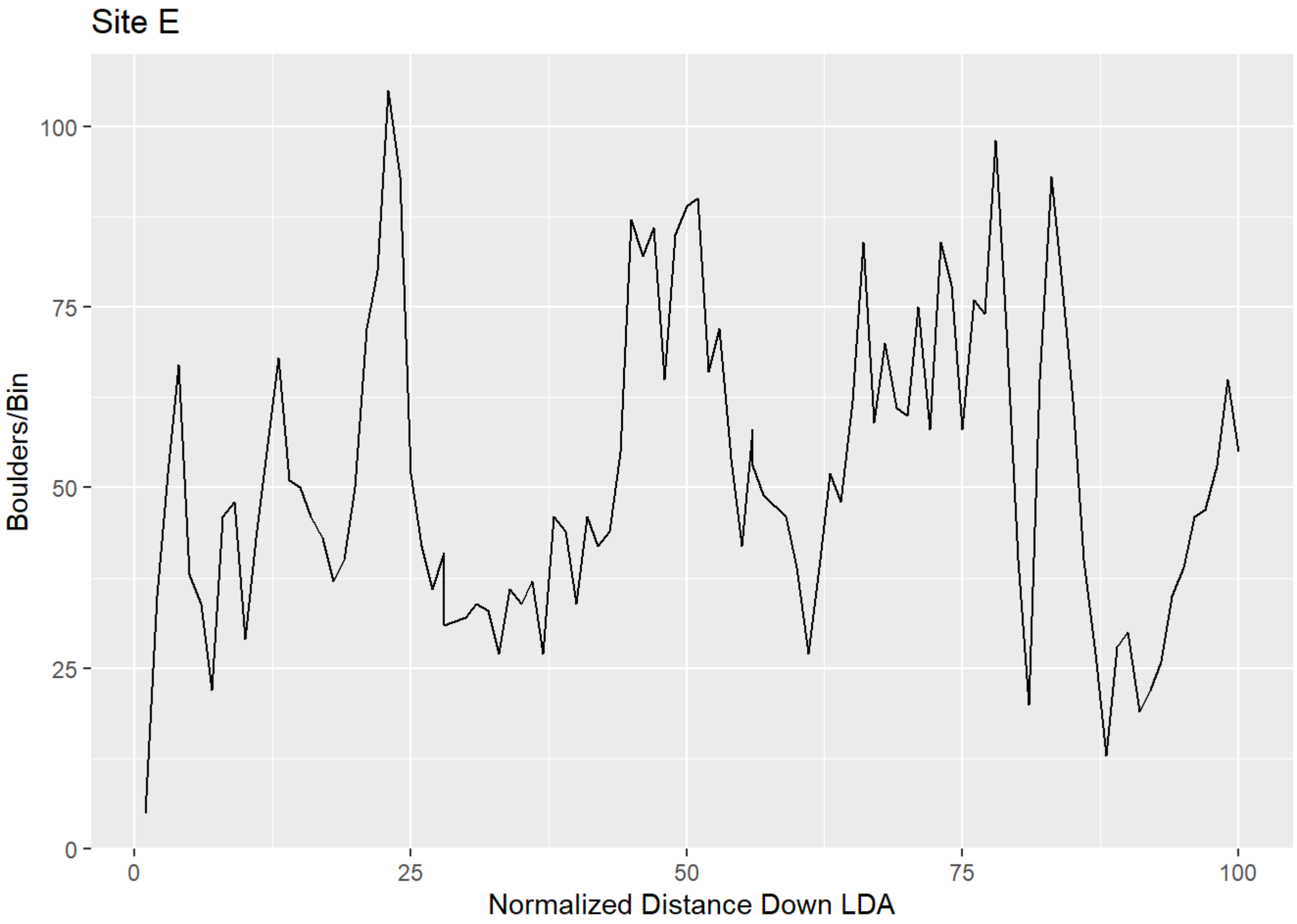
Site E
100
75
50
25
0
Boulders/Bin
0
25
50
75
100
Normalized Distance Down LDA

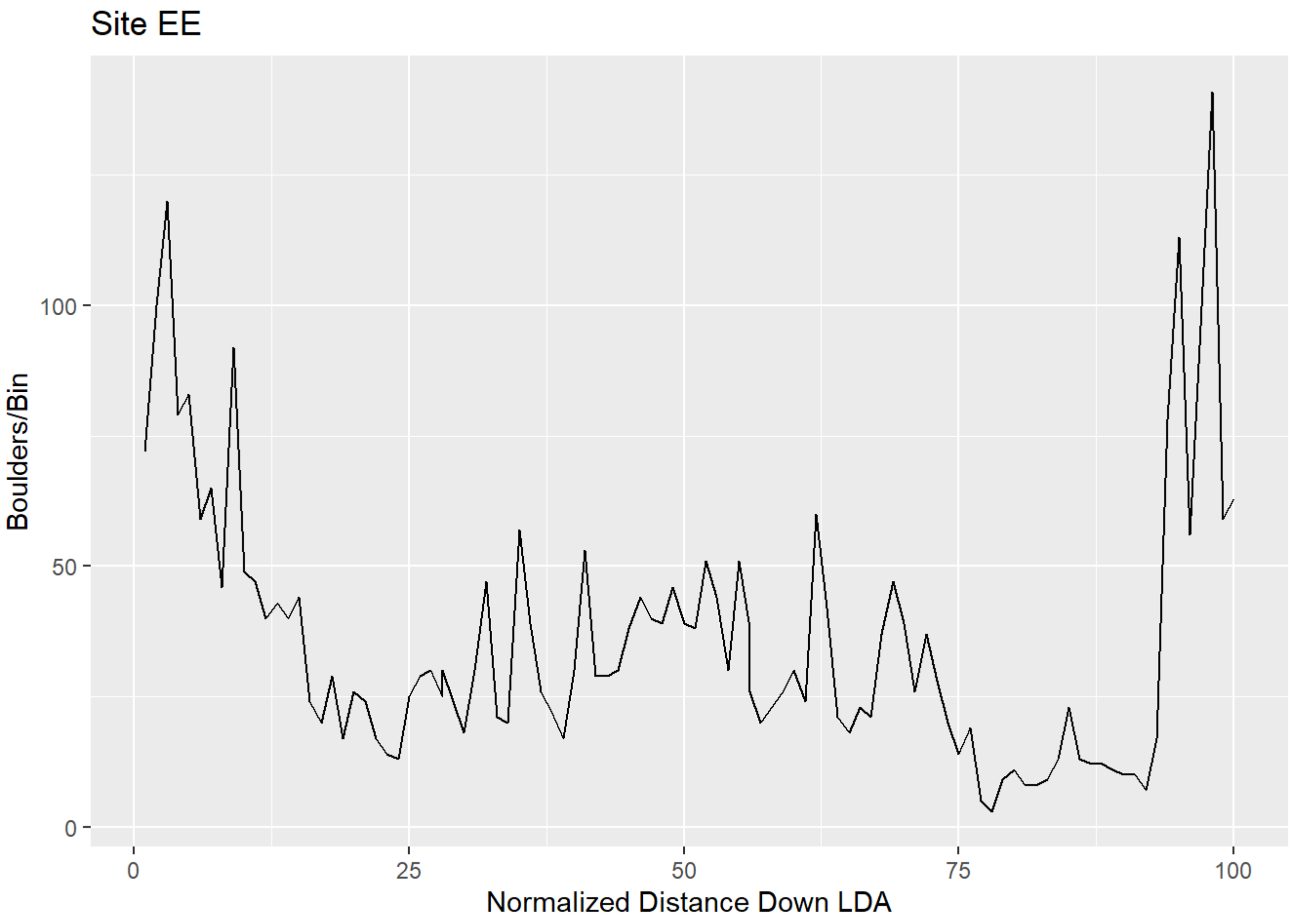
Site EE
100
50
0
Boulders/Bin
0
25
50
75
100
Normalized Distance Down LDA

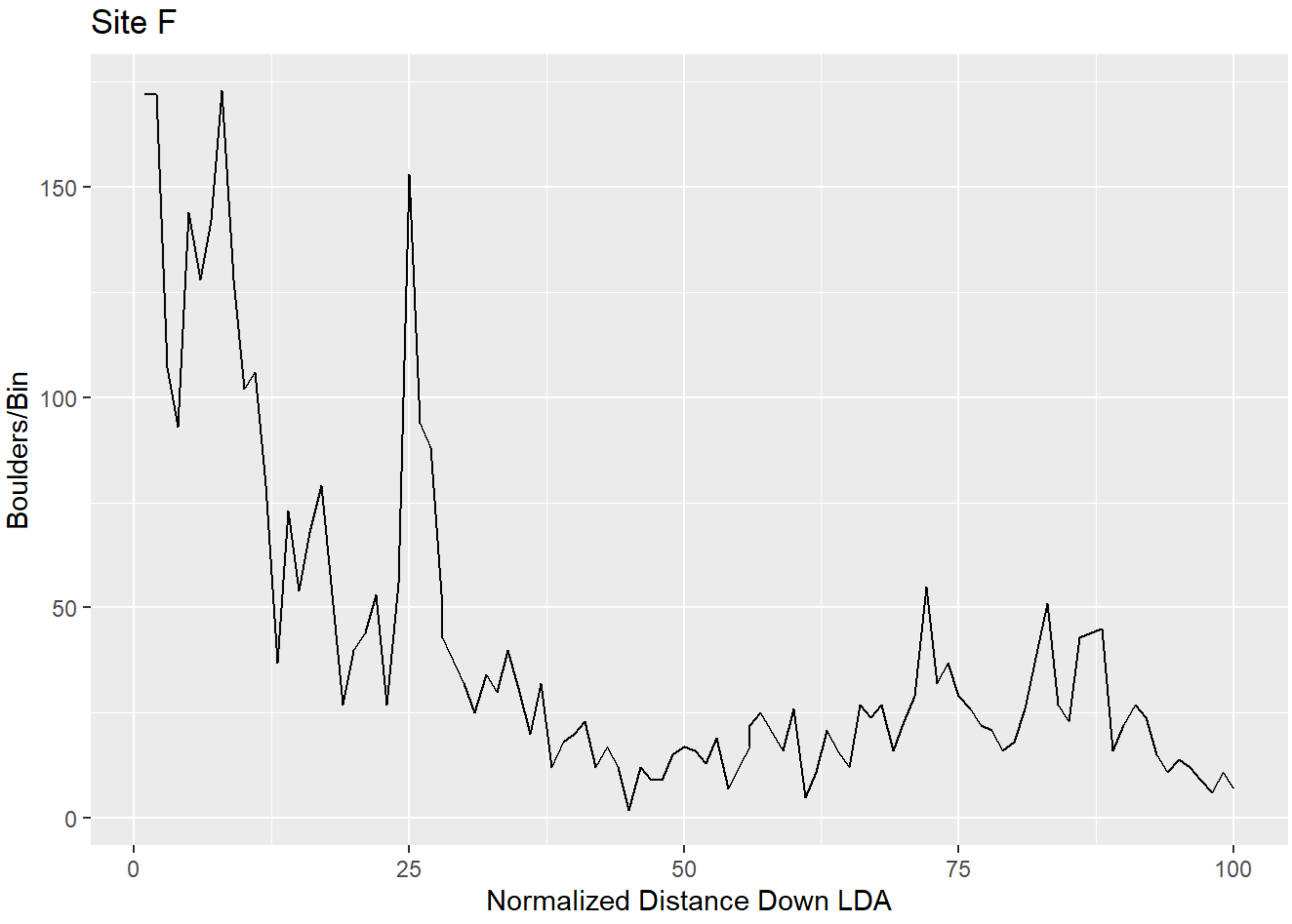
Site F
150
100
50
0
Boulders/Bin
0
25
50
75
100
Normalized Distance Down LDA

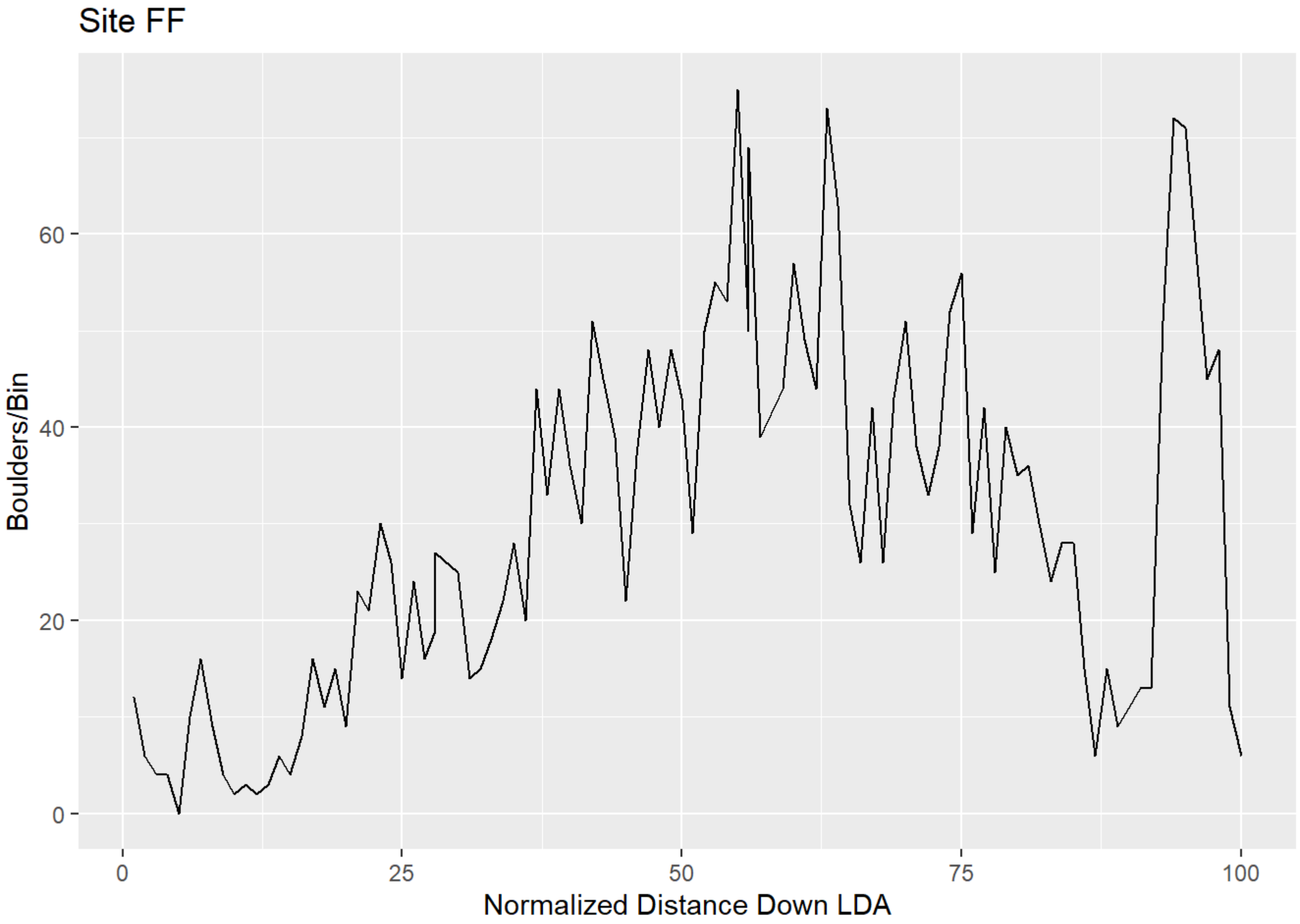
Site FF
Boulders/Bin
0
20
40
60
0
25
50
75
100
Normalized Distance Down LDA

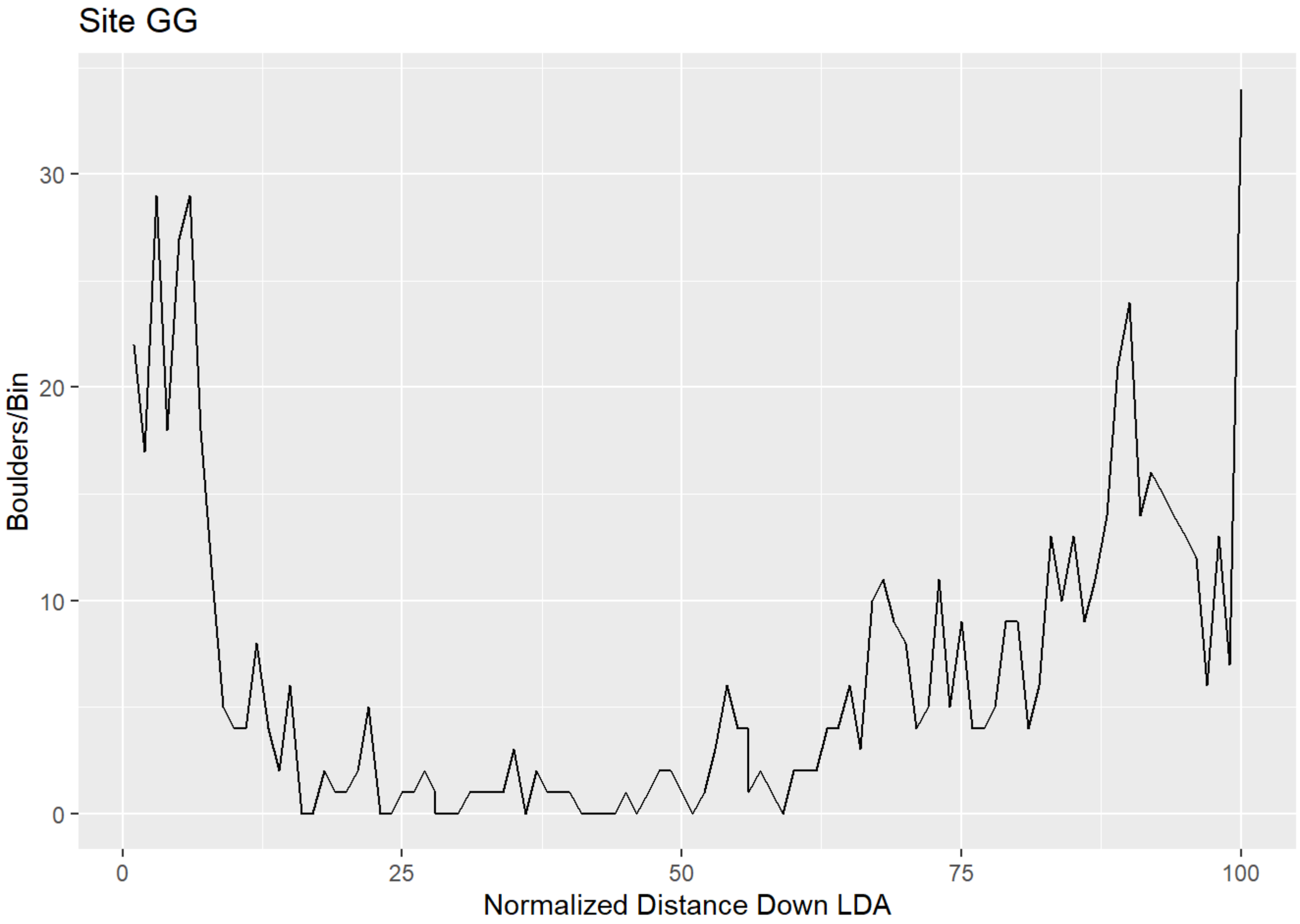
Site GG
Boulders/Bin
0
10
20
30
0
25
50
75
100
Normalized Distance Down LDA

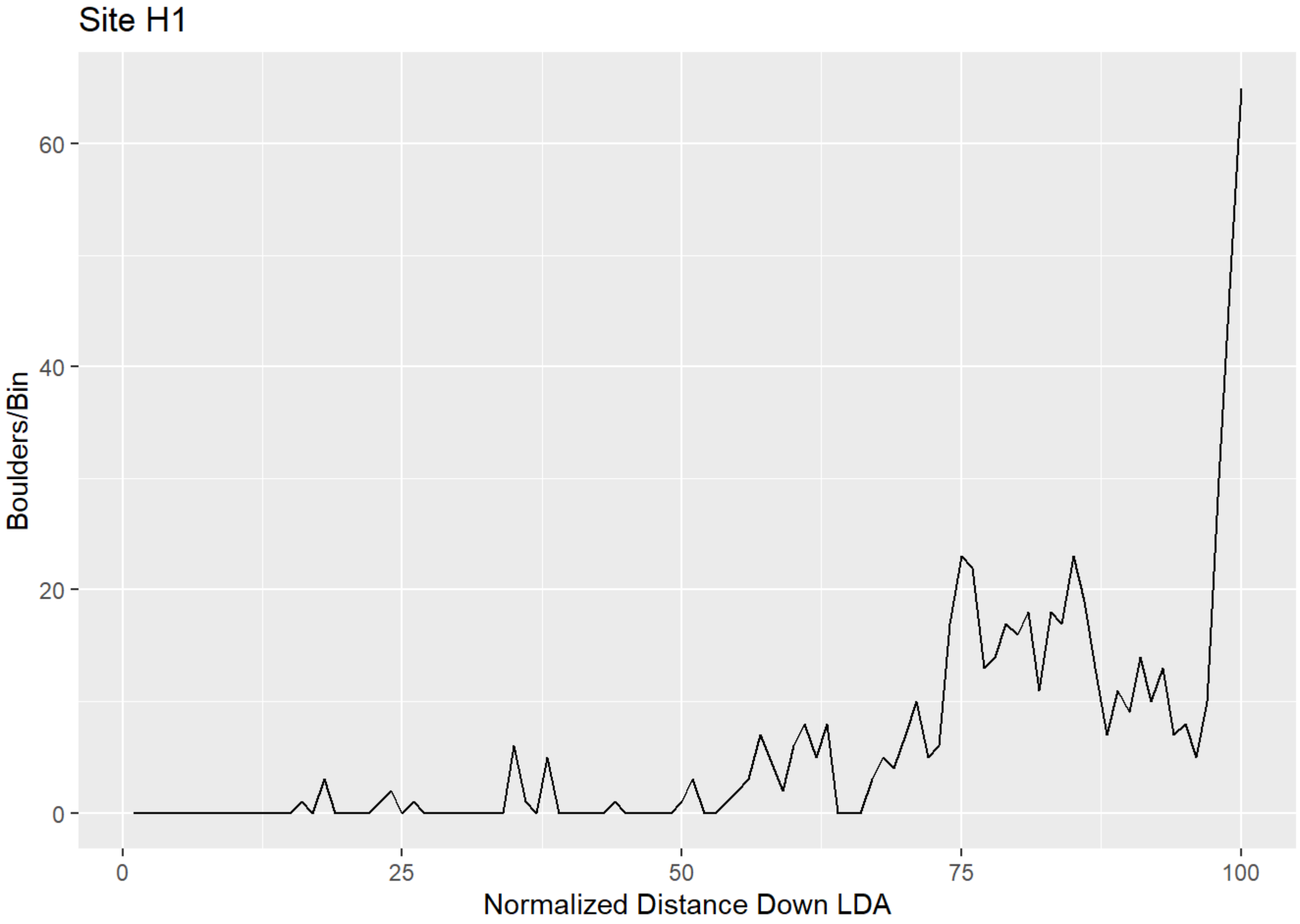
Site H1
Boulders/Bin
0
20
40
60
0
25
50
75
100
Normalized Distance Down LDA

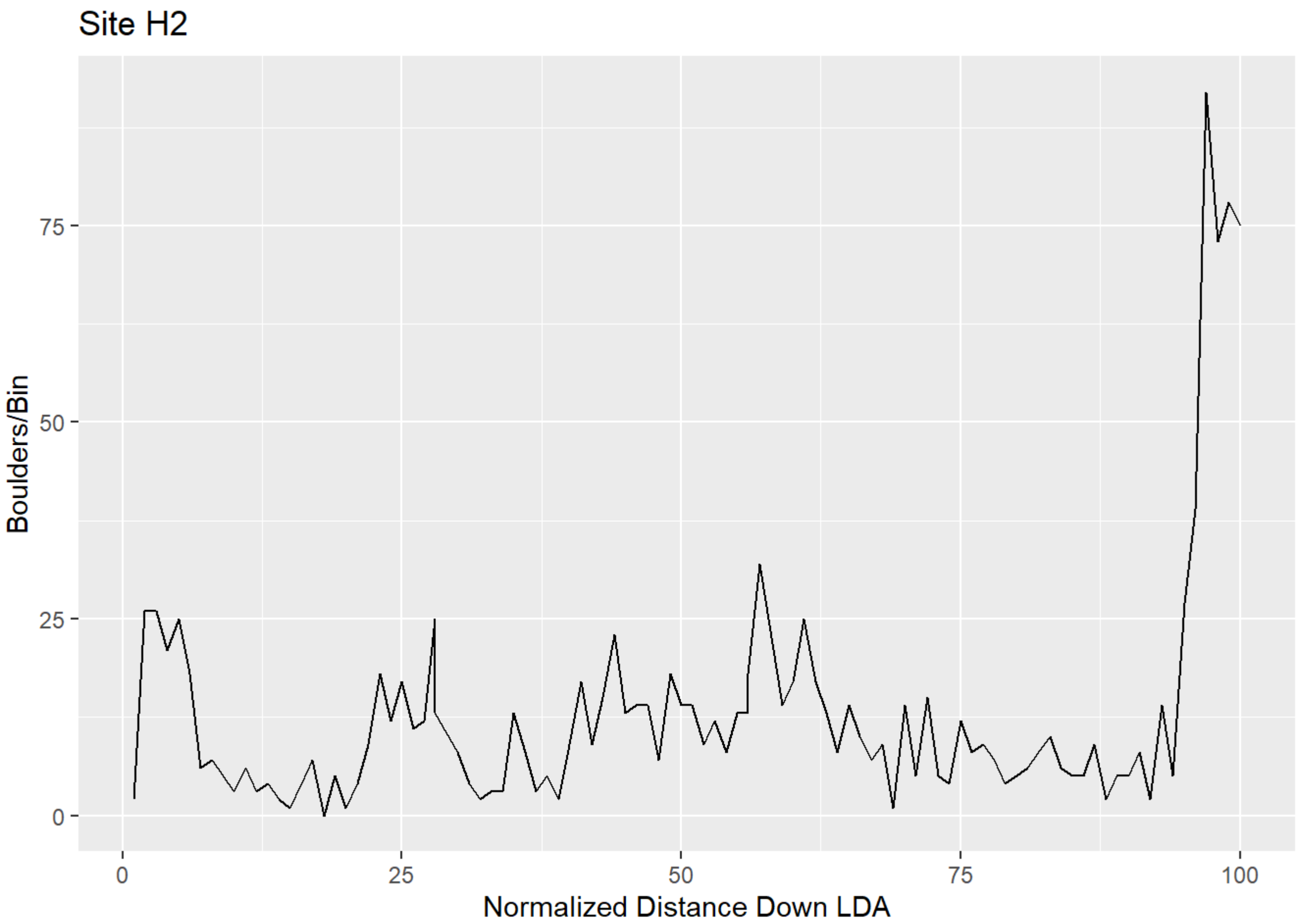
Site H2
Boulders/Bin
0
25
50
75
0
25
50
75
100
Normalized Distance Down LDA

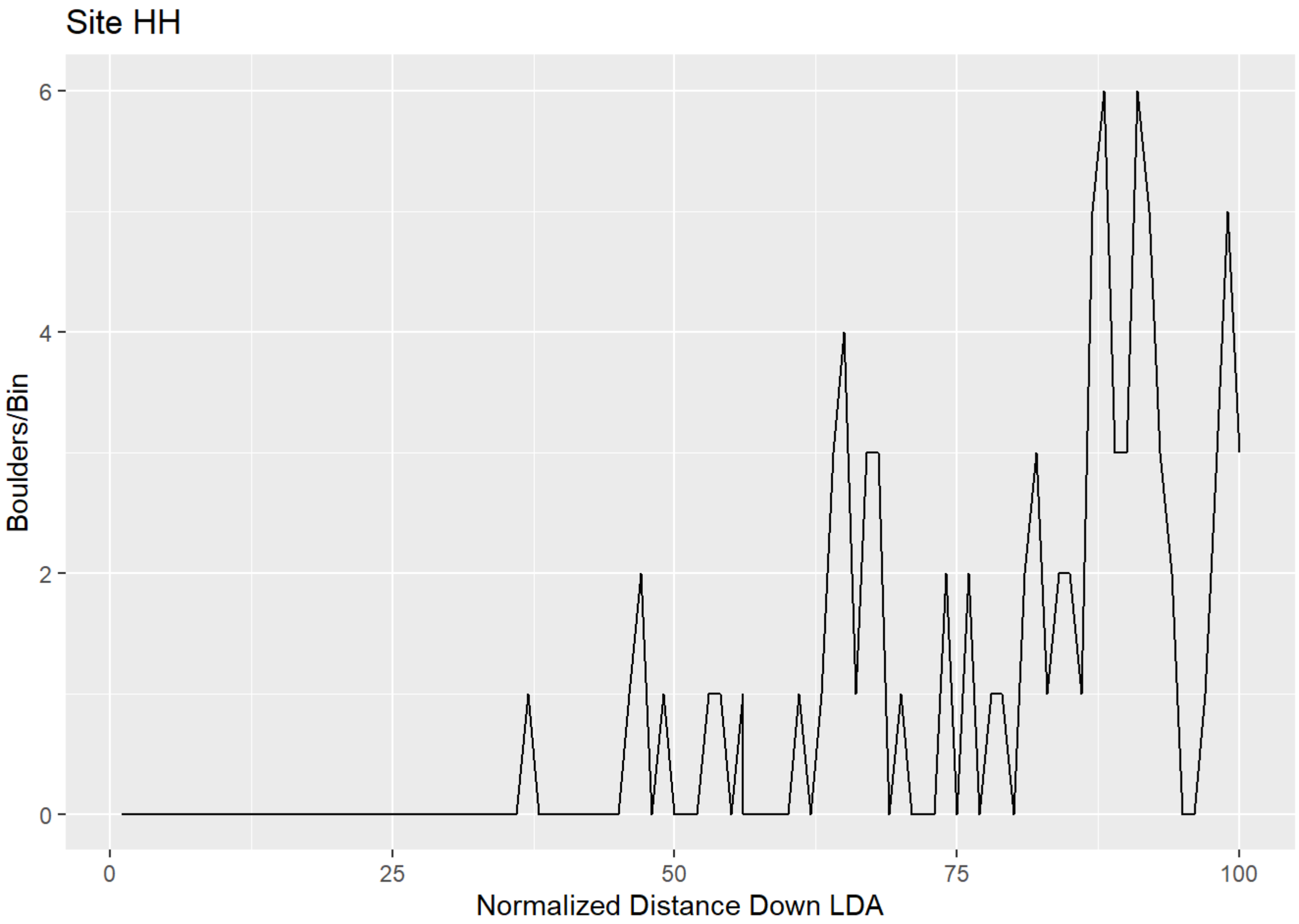
Site HH
Boulders/Bin
0
2
4
6
0
25
50
75
100
Normalized Distance Down LDA

Site J1

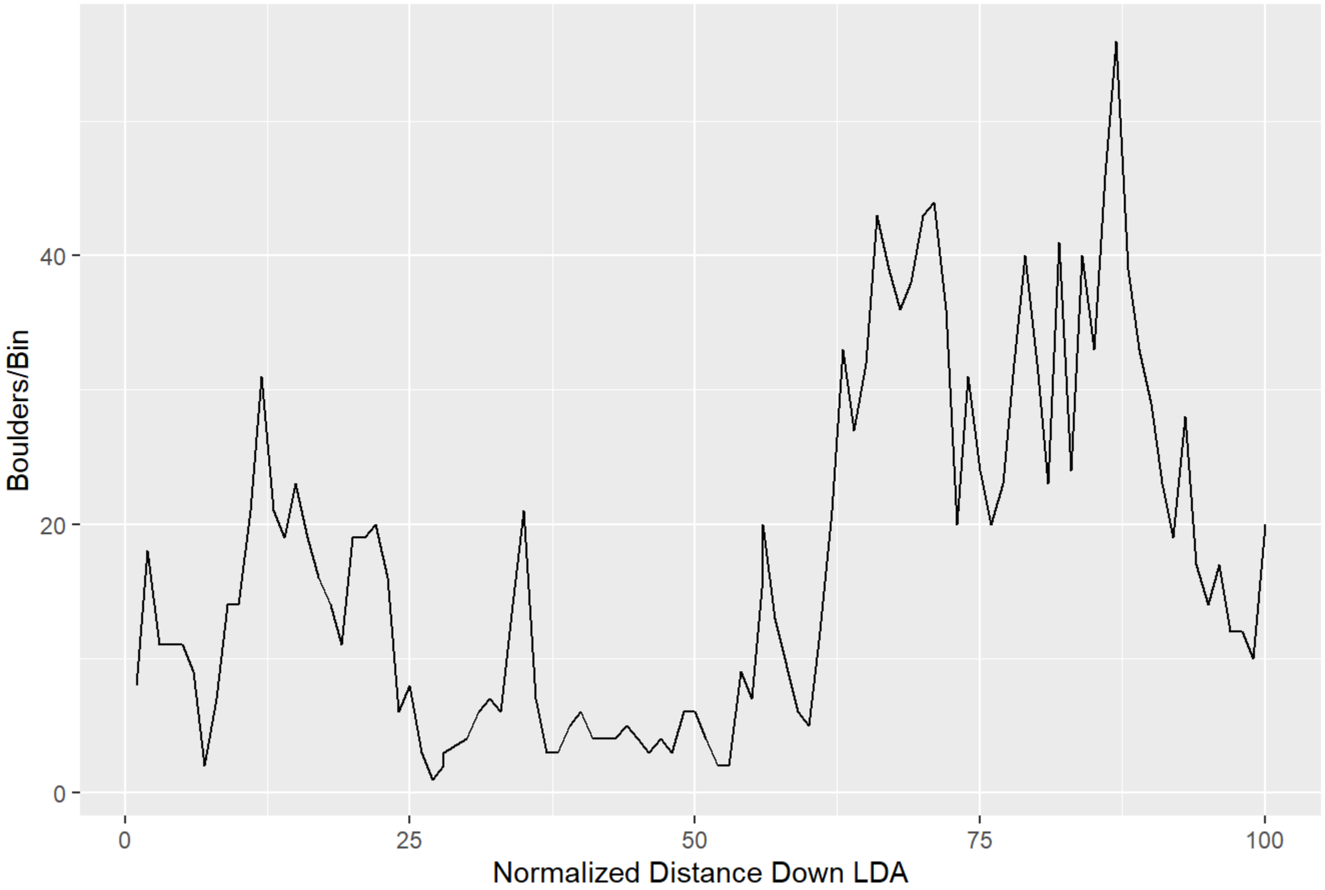

Site J2

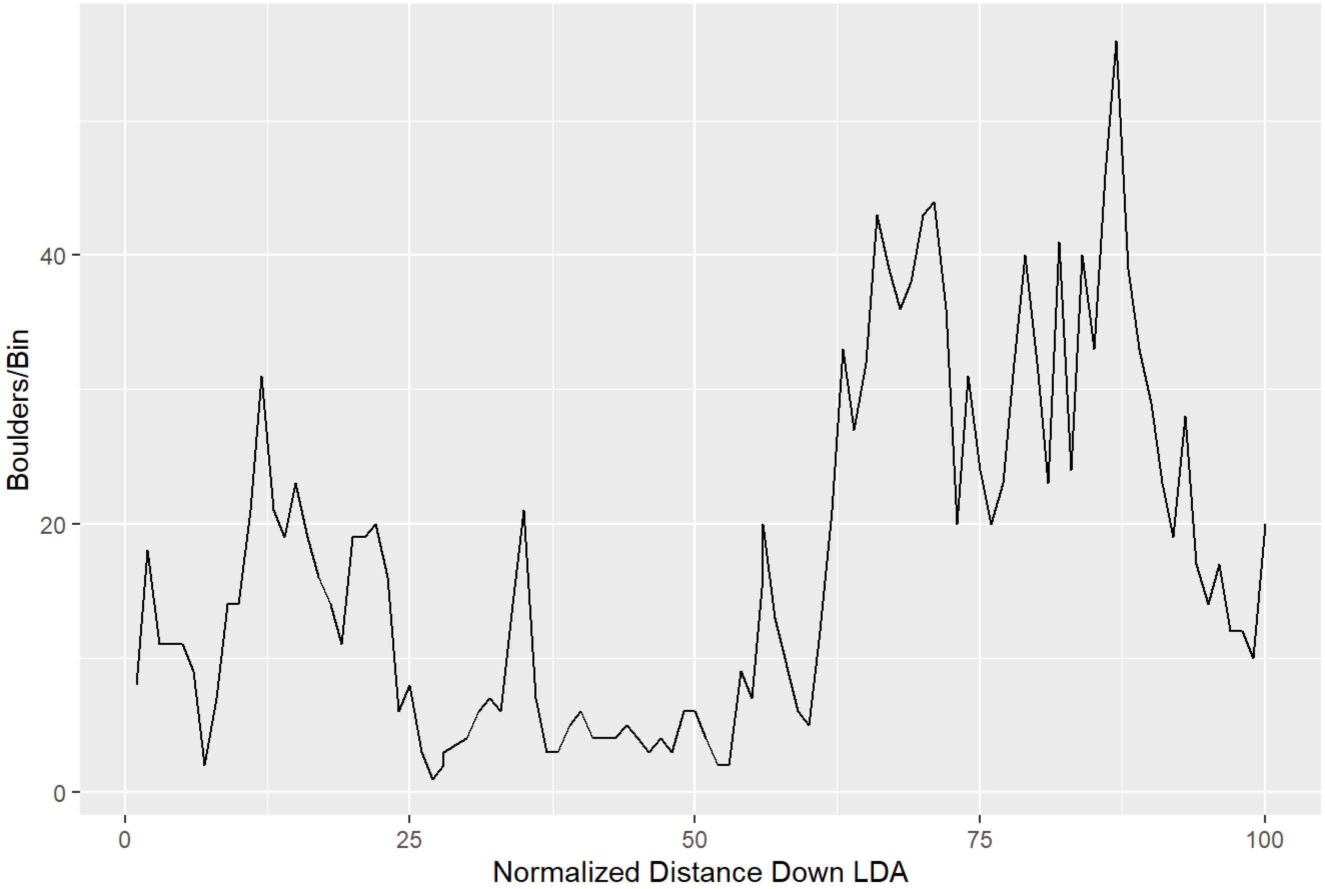

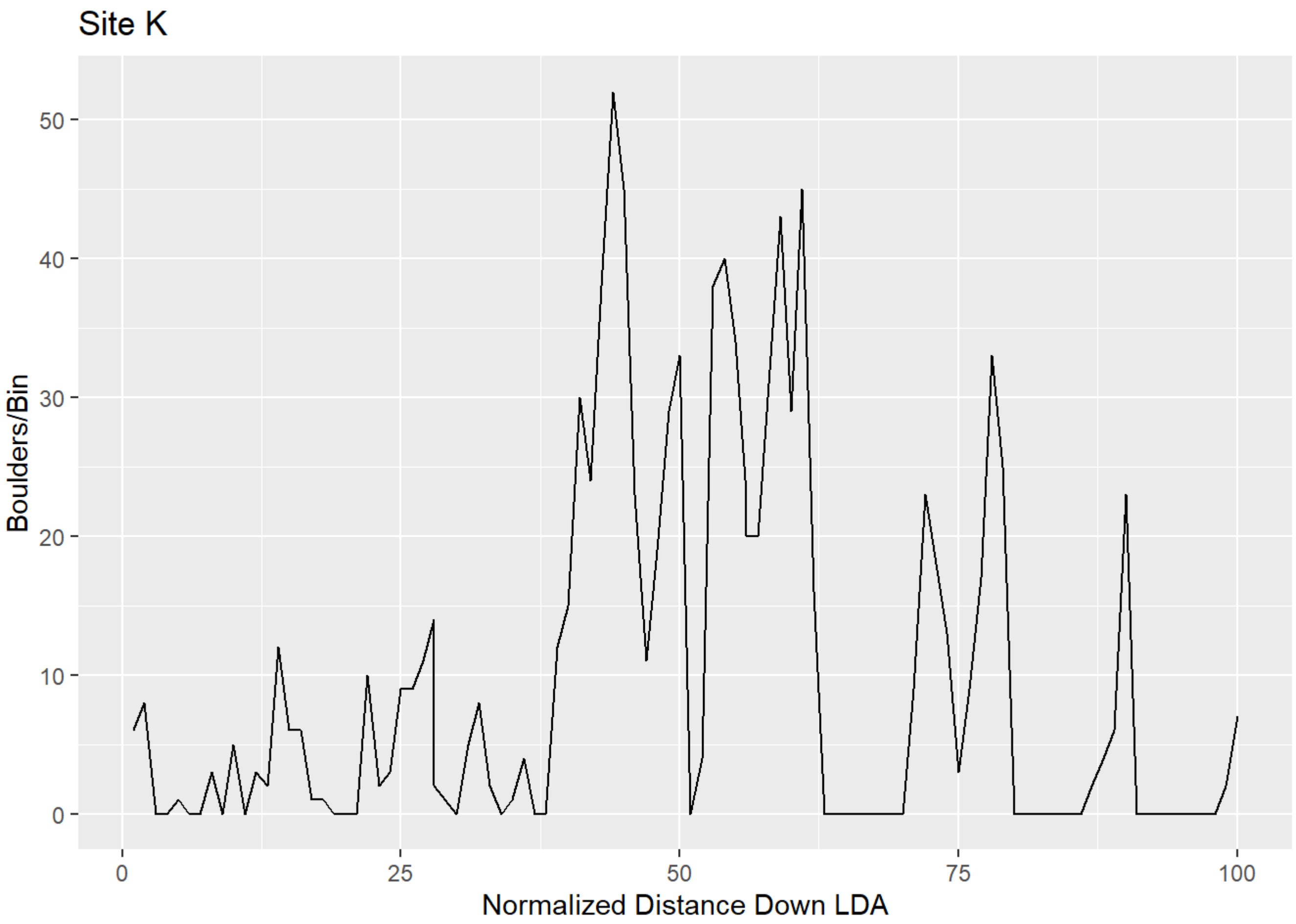
Site K
Boulders/Bin
0
10
20
30
40
50
0
25
50
75
100
Normalized Distance Down LDA

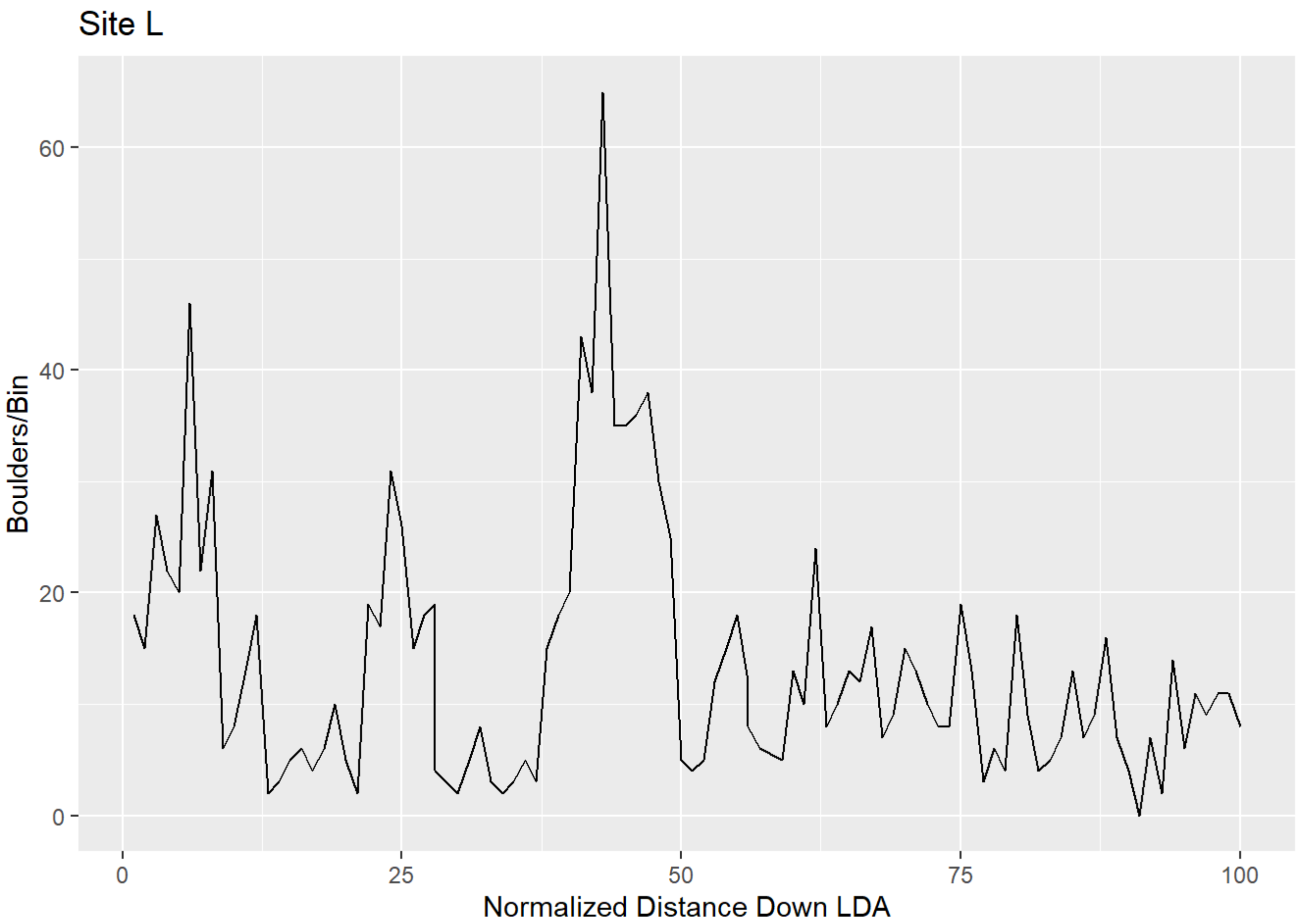
Site L
Boulders/Bin
0
20
40
60
0
25
50
75
100
Normalized Distance Down LDA

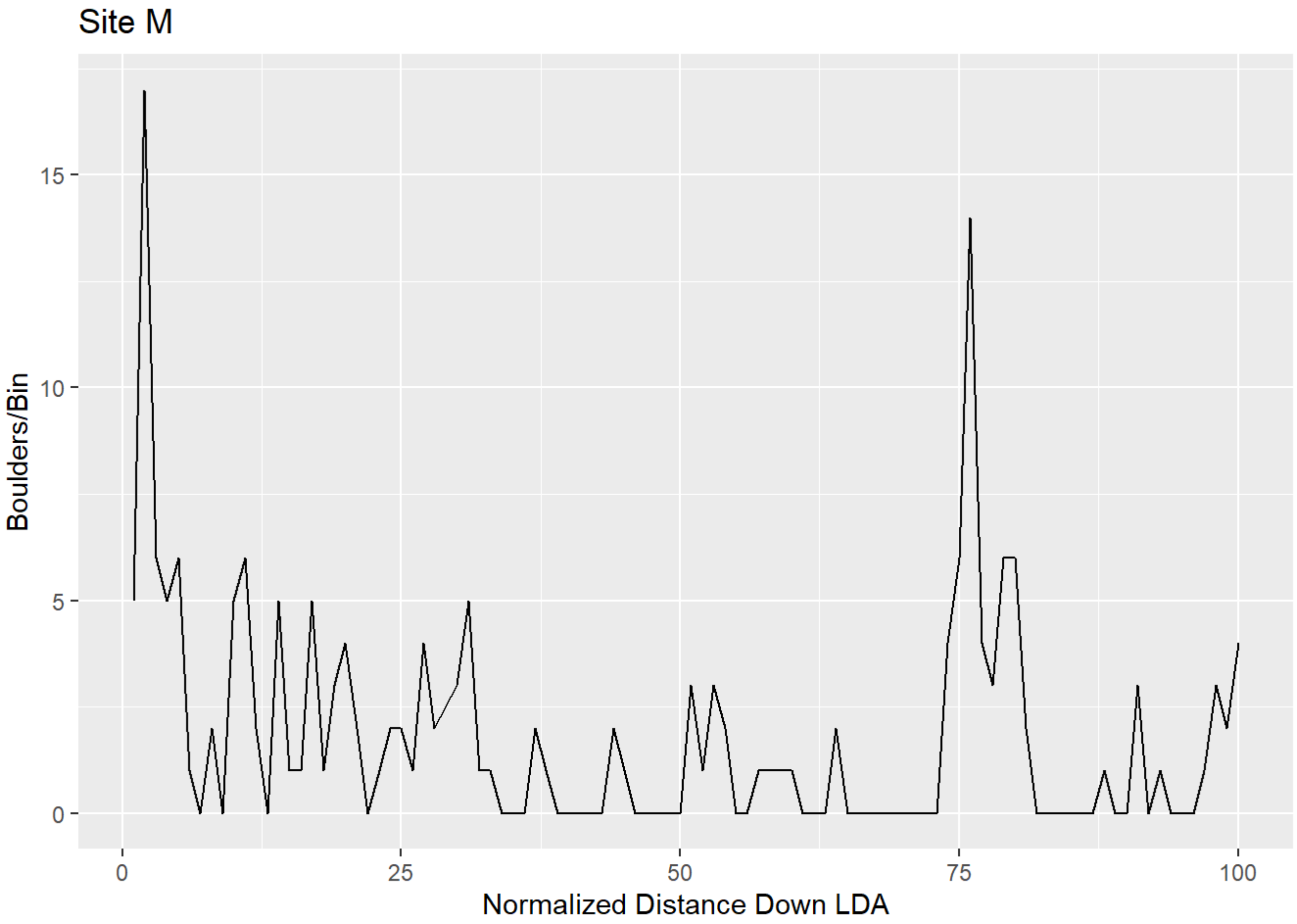
Site M
Boulders/Bin
0
5
10
15
0
25
50
75
100
Normalized Distance Down LDA

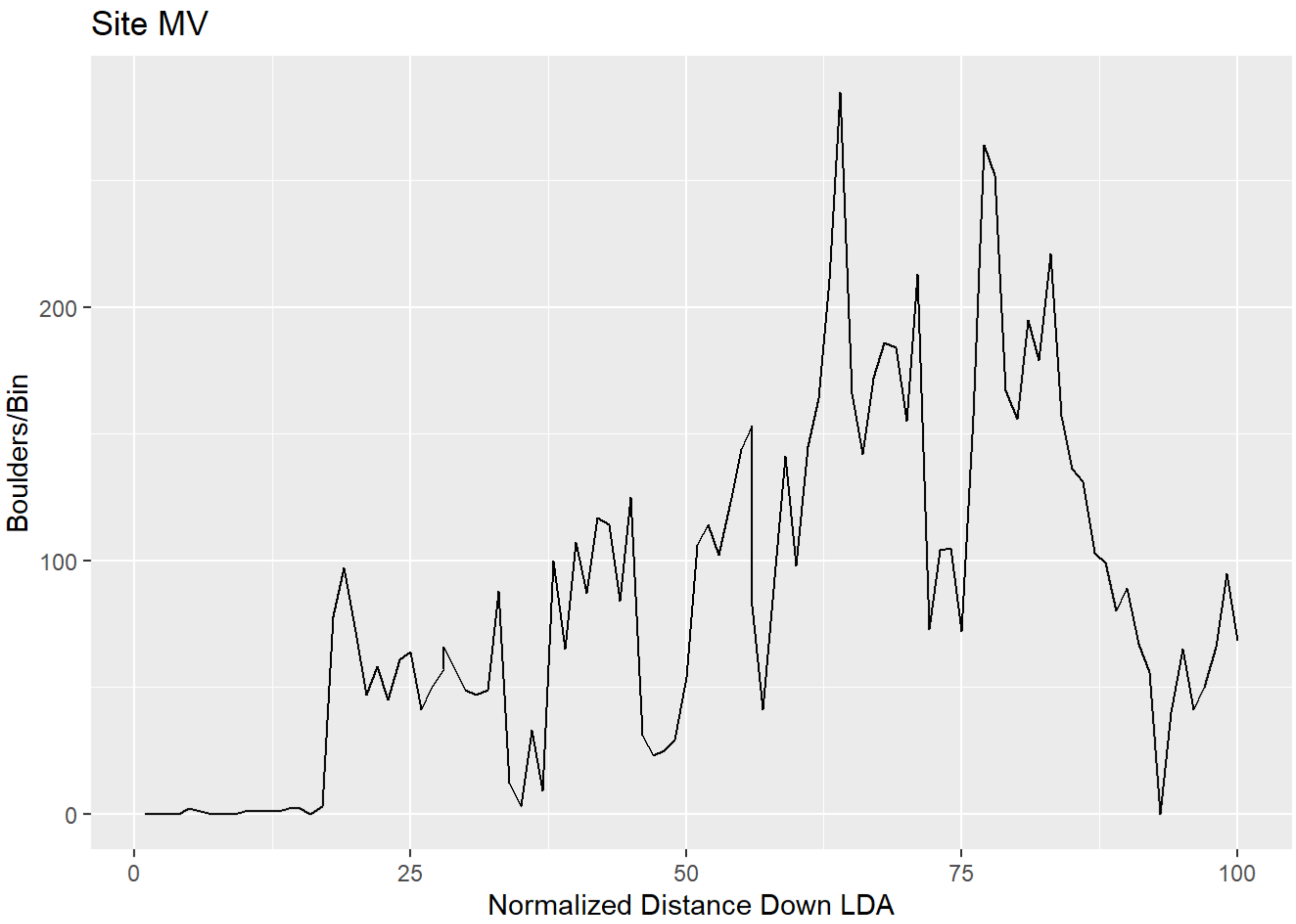
Site MV
Boulders/Bin
0
100
200
0
25
50
75
100
Normalized Distance Down LDA

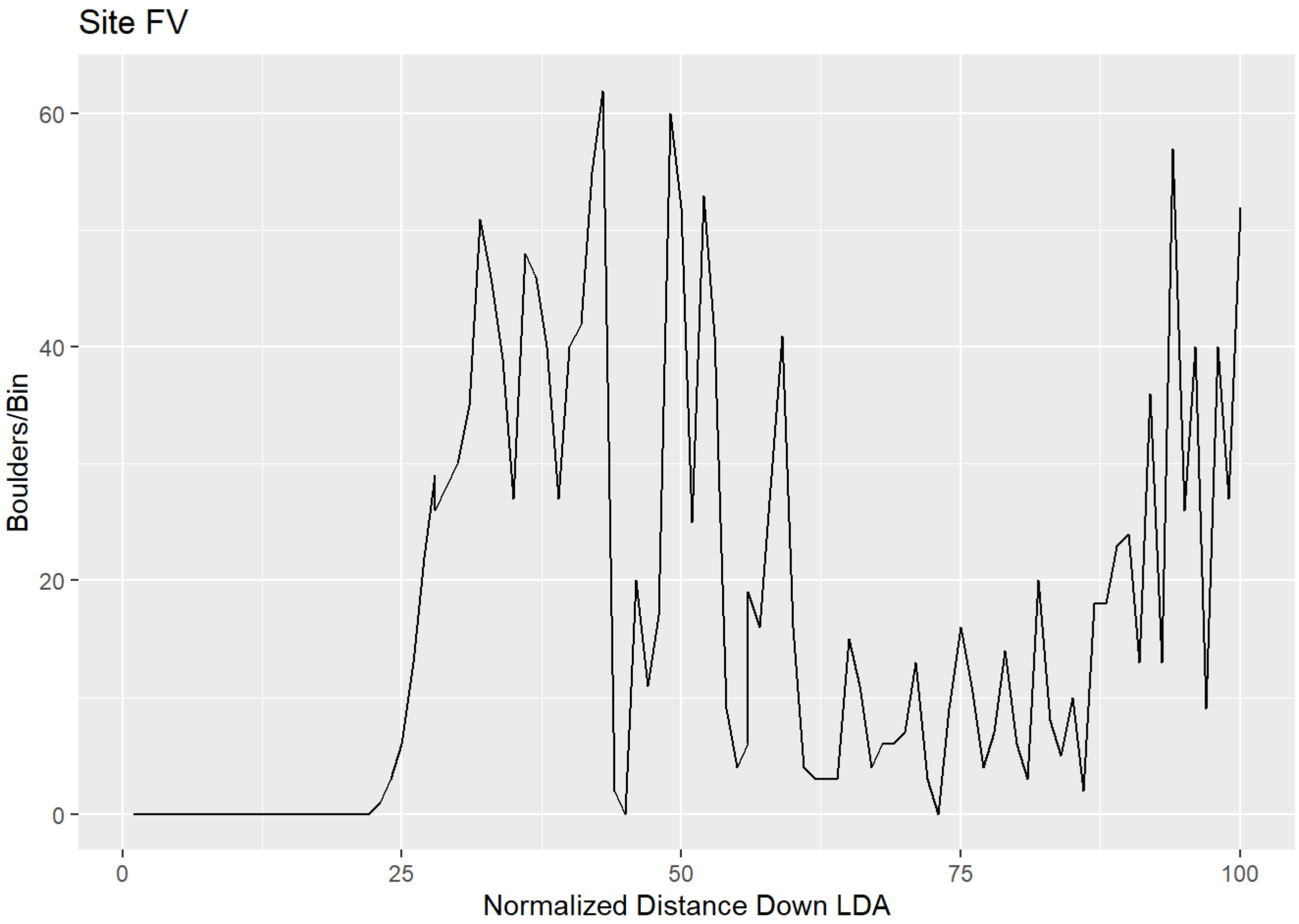
Site FV
Boulders/Bin
0
20
40
60
0
25
50
75
100
Normalized Distance Down LDA

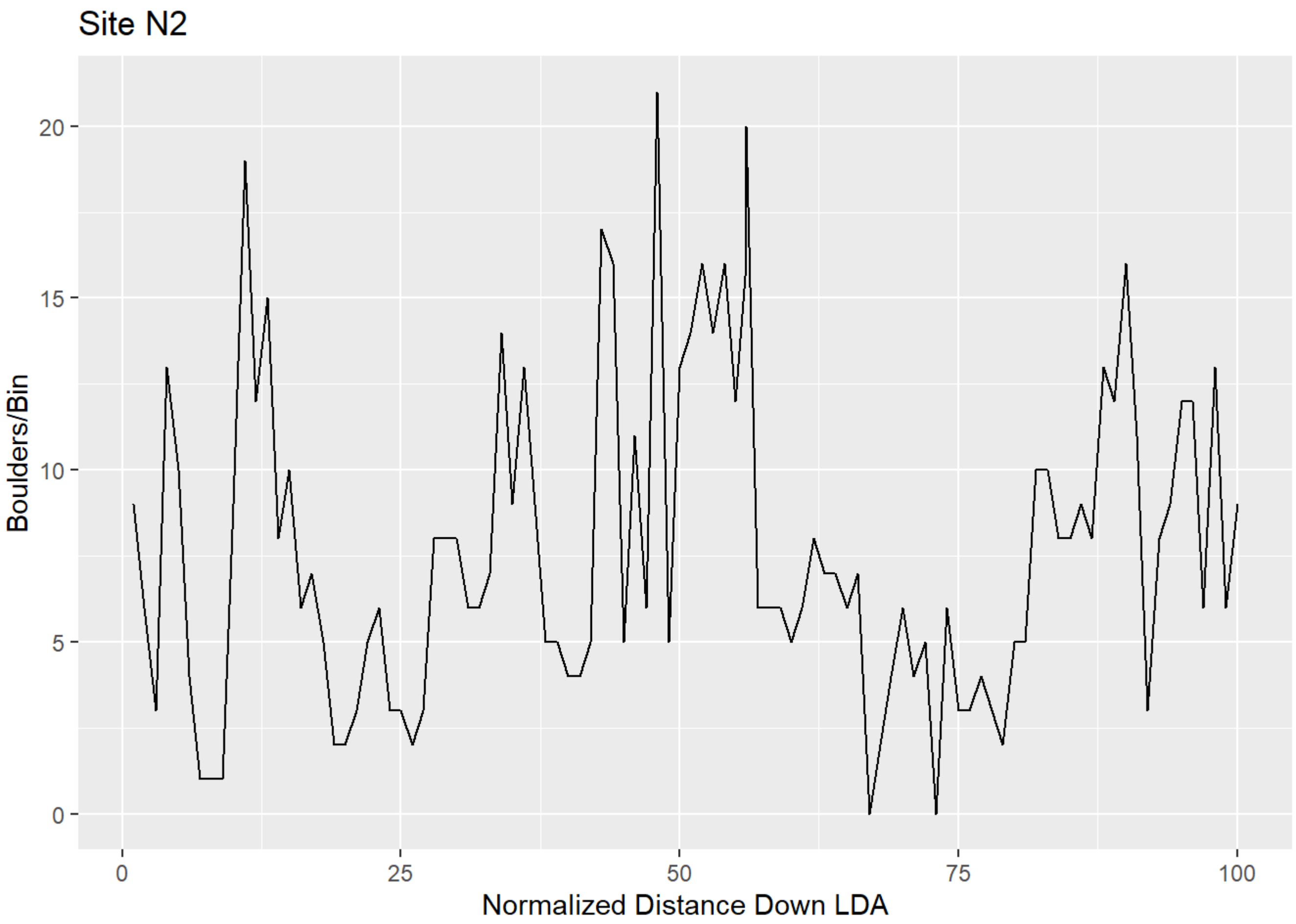
Site N2
Boulders/Bin
Normalized Distance Down LDA
0
5
10
15
20
0
25
50
75
100

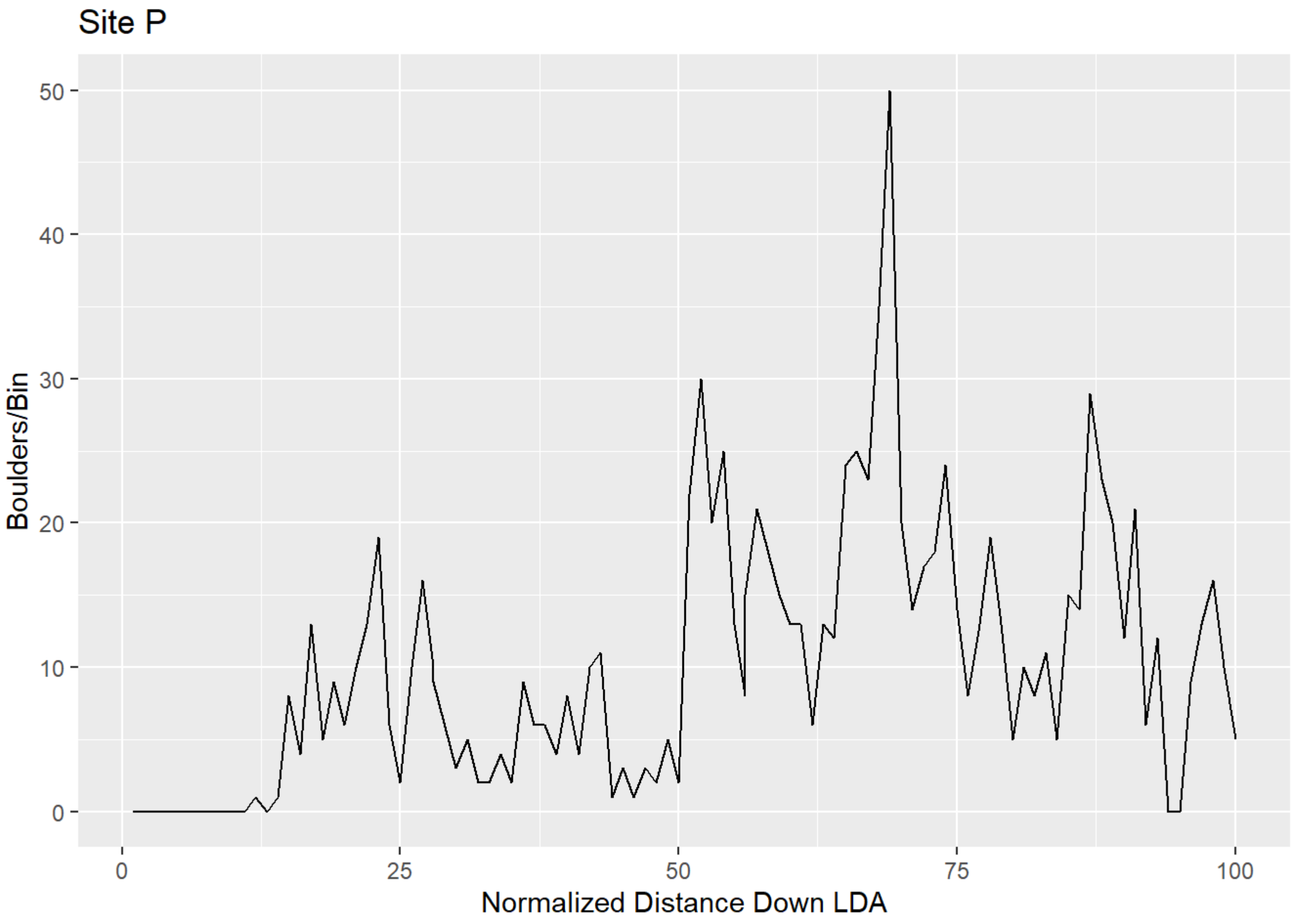
Site P
Boulders/Bin
Normalized Distance Down LDA
0
10
20
30
40
50
0
25
50
75
100

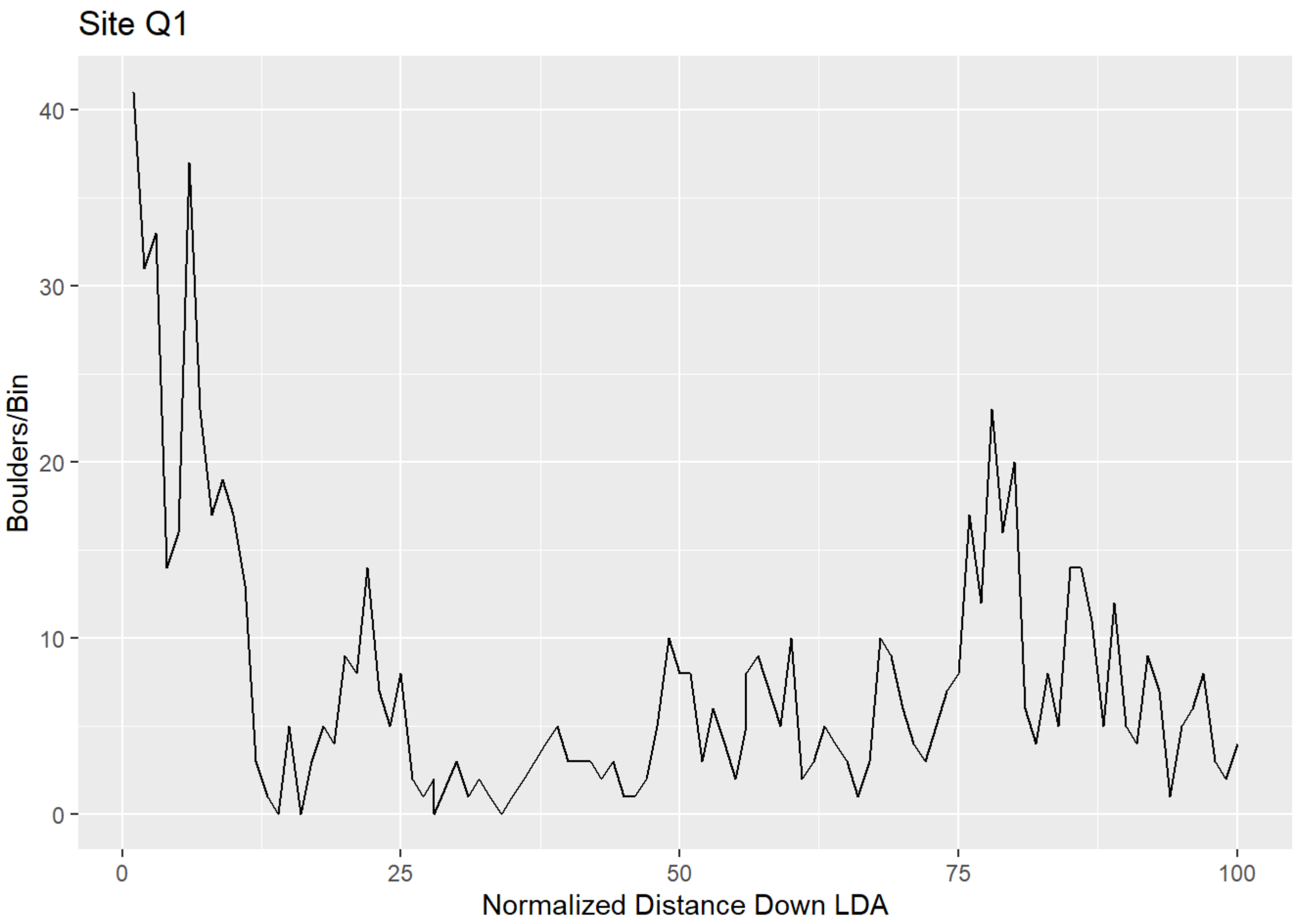
Site Q1
Boulders/Bin
40
30
20
10
0
0
25
50
75
100
Normalized Distance Down LDA

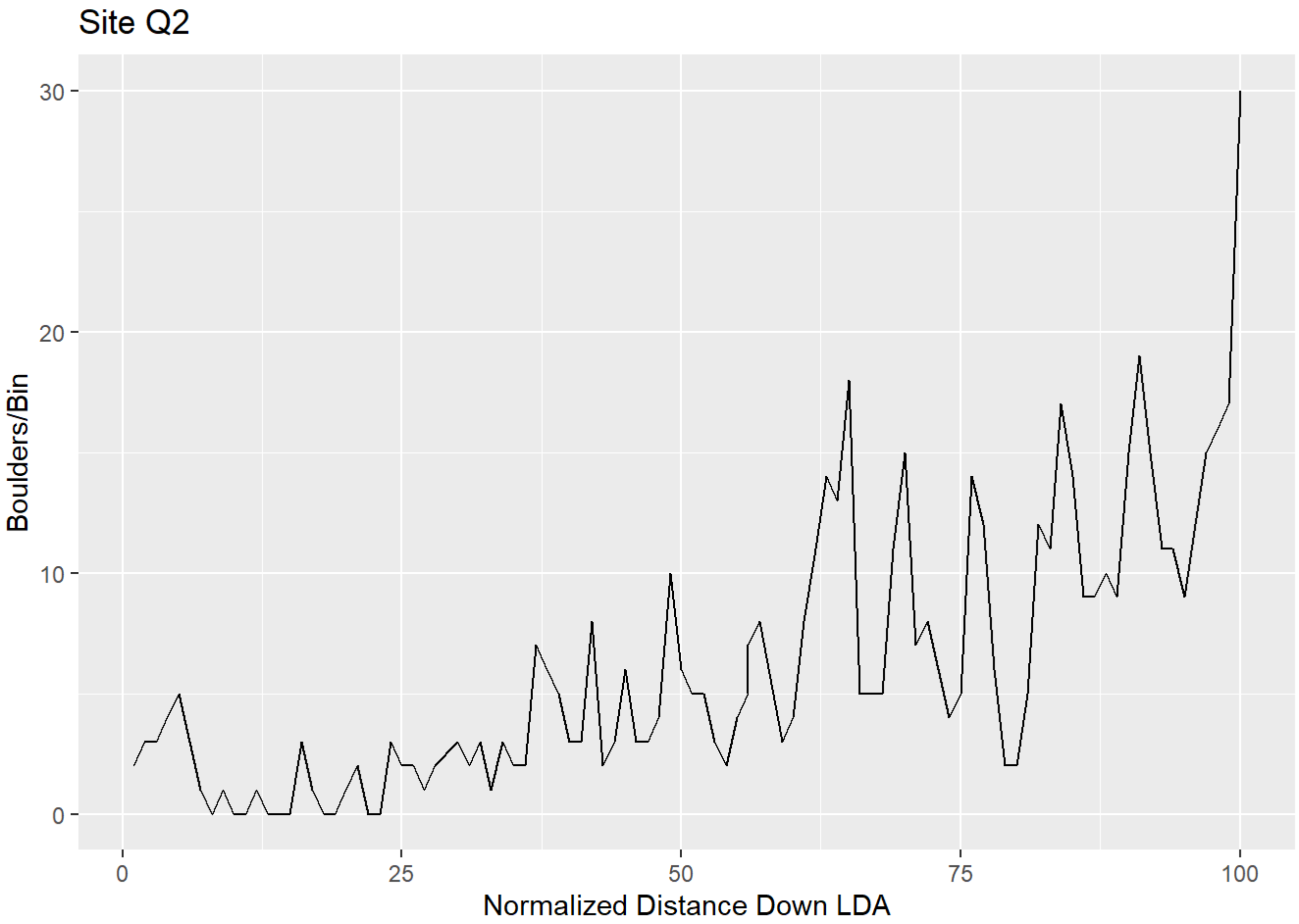
Site Q2
Boulders/Bin
30
20
10
0
0
25
50
75
100
Normalized Distance Down LDA

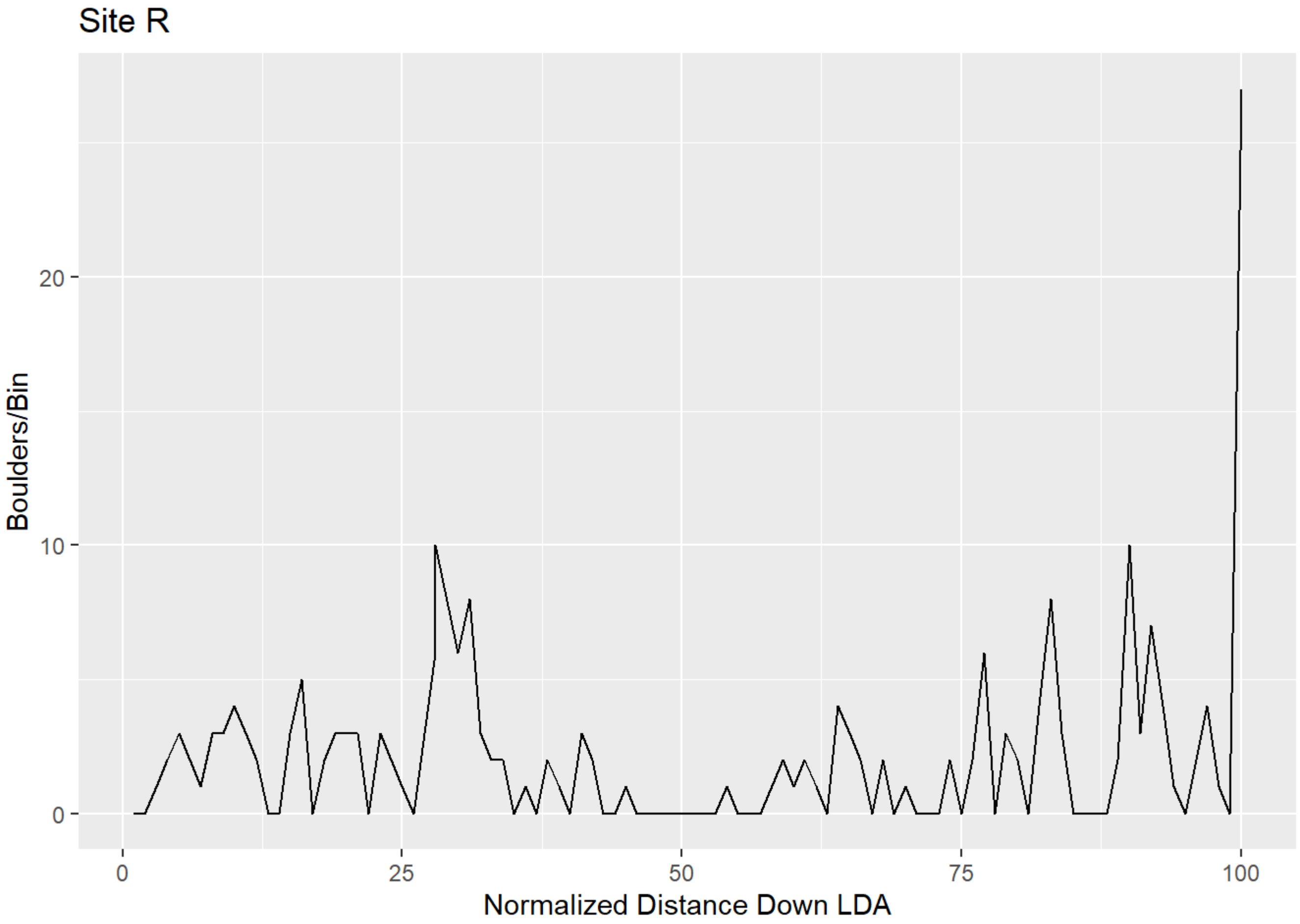
Site R
20
10
0
Boulders/Bin
0
25
50
75
100
Normalized Distance Down LDA

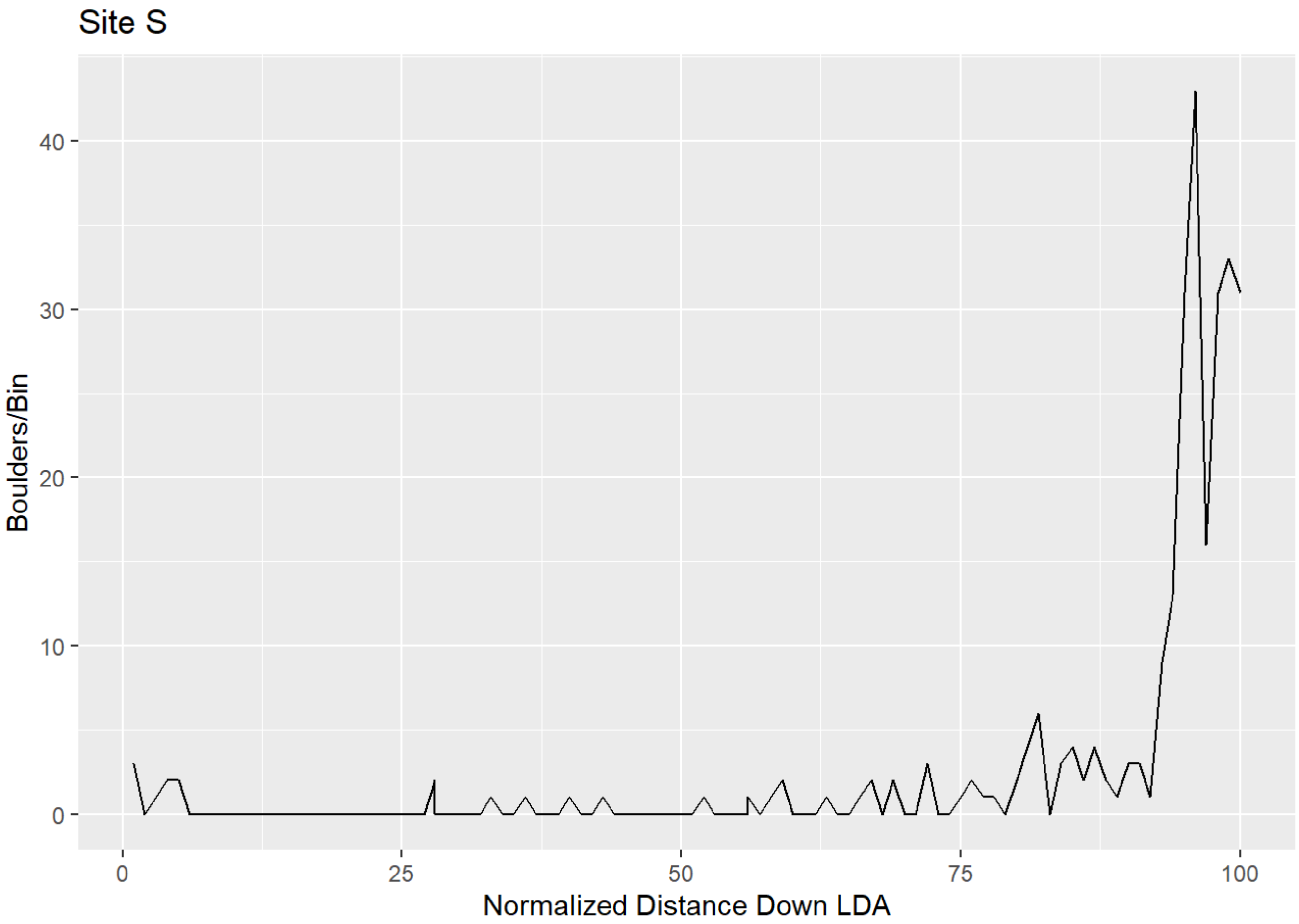
Site S
40
30
20
10
0
Boulders/Bin
0
25
50
75
100
Normalized Distance Down LDA

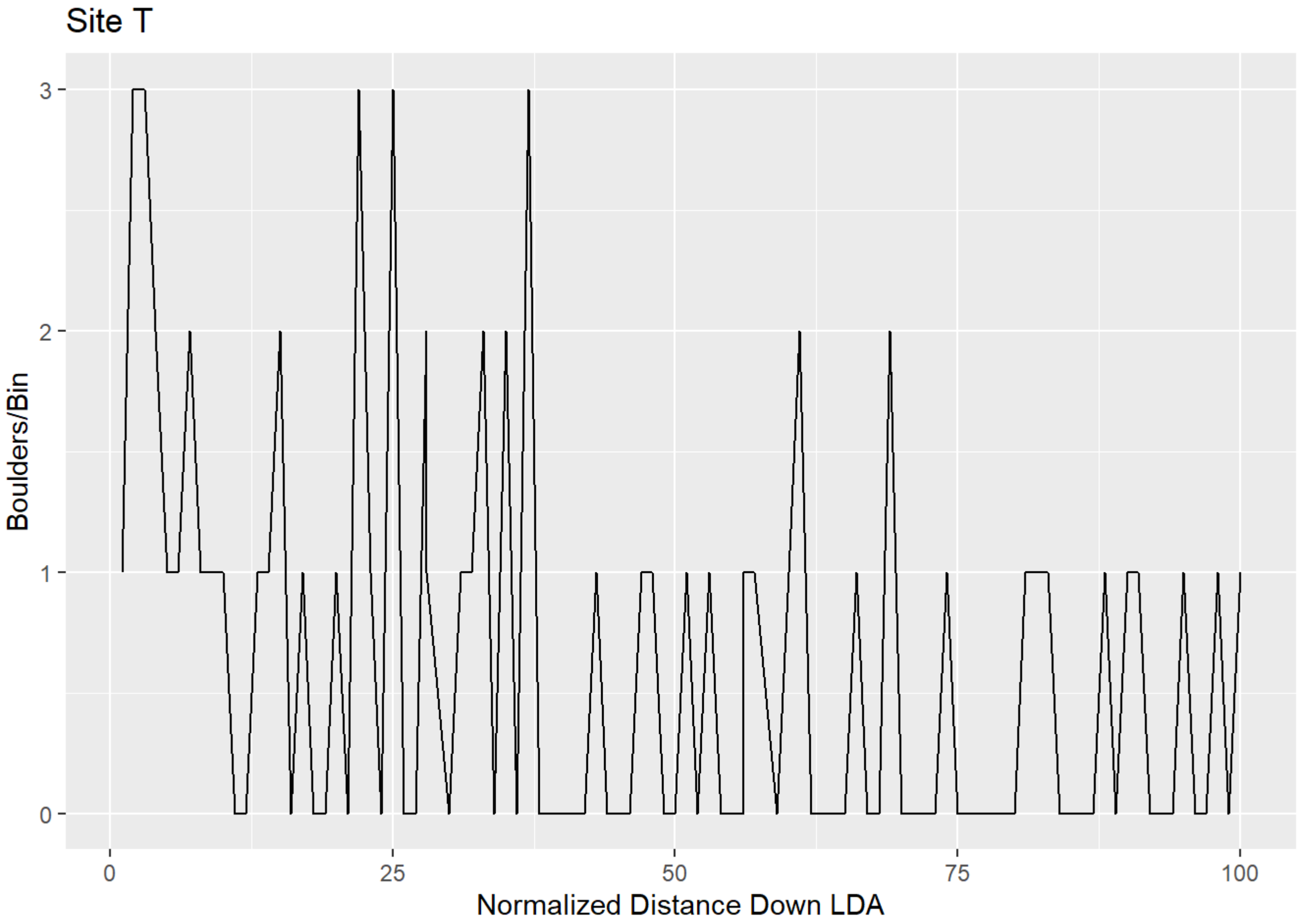
Site T
Boulders/Bin
3
2
1
0
0
25
50
75
100
Normalized Distance Down LDA

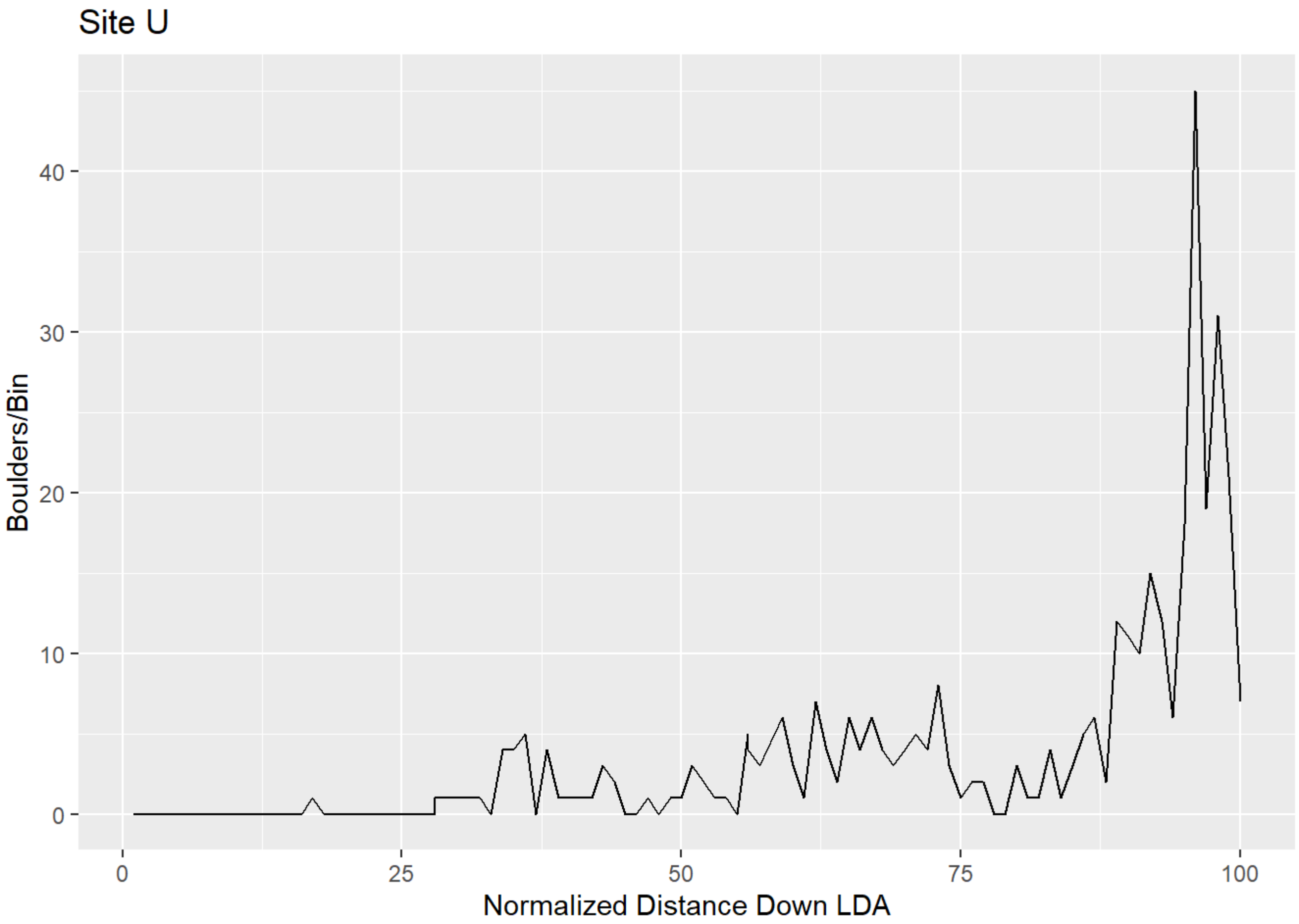
Site U
Boulders/Bin
40
30
20
10
0
0
25
50
75
100
Normalized Distance Down LDA

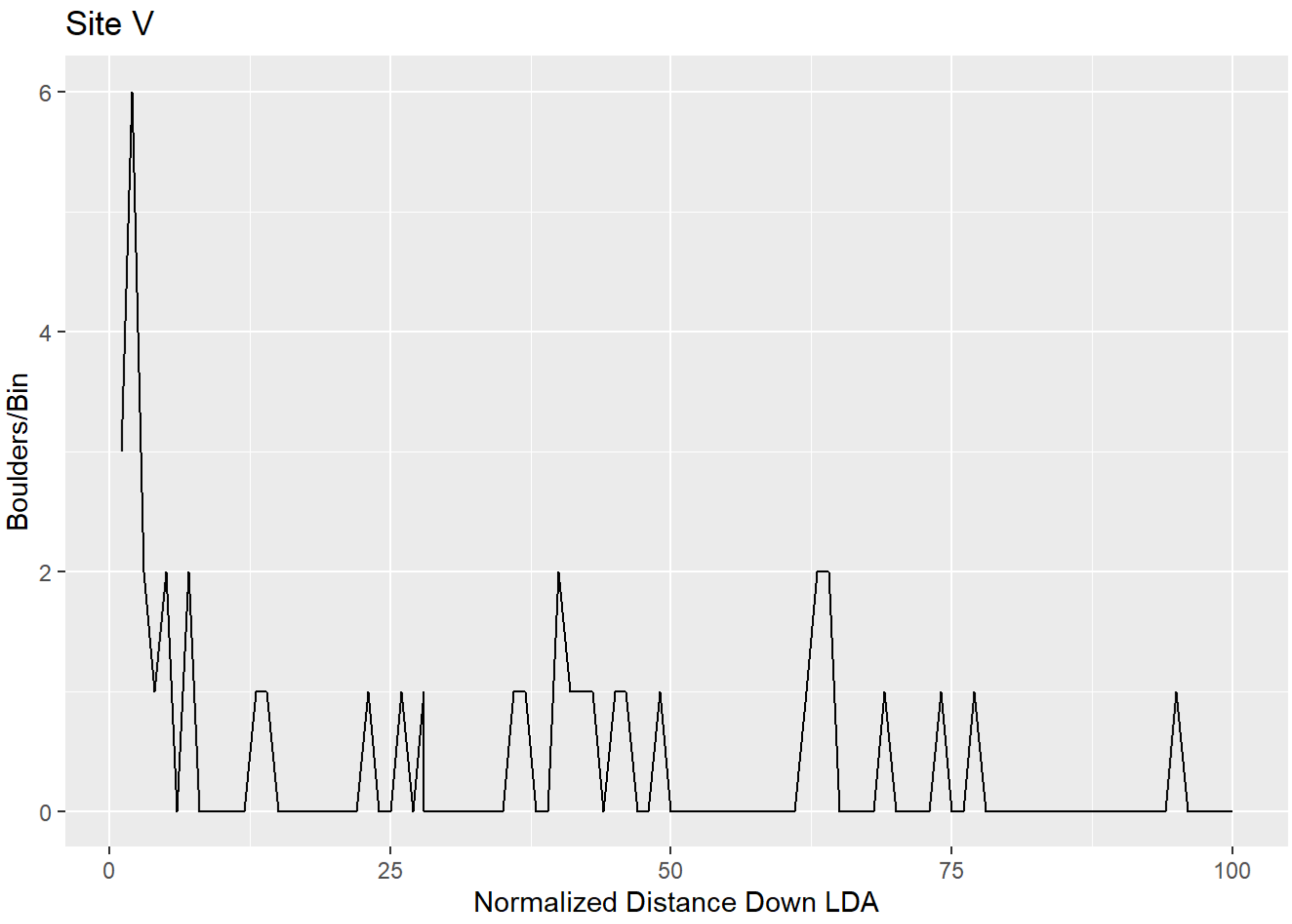
Site V
Boulders/Bin
0
2
4
6
0
25
50
75
100
Normalized Distance Down LDA

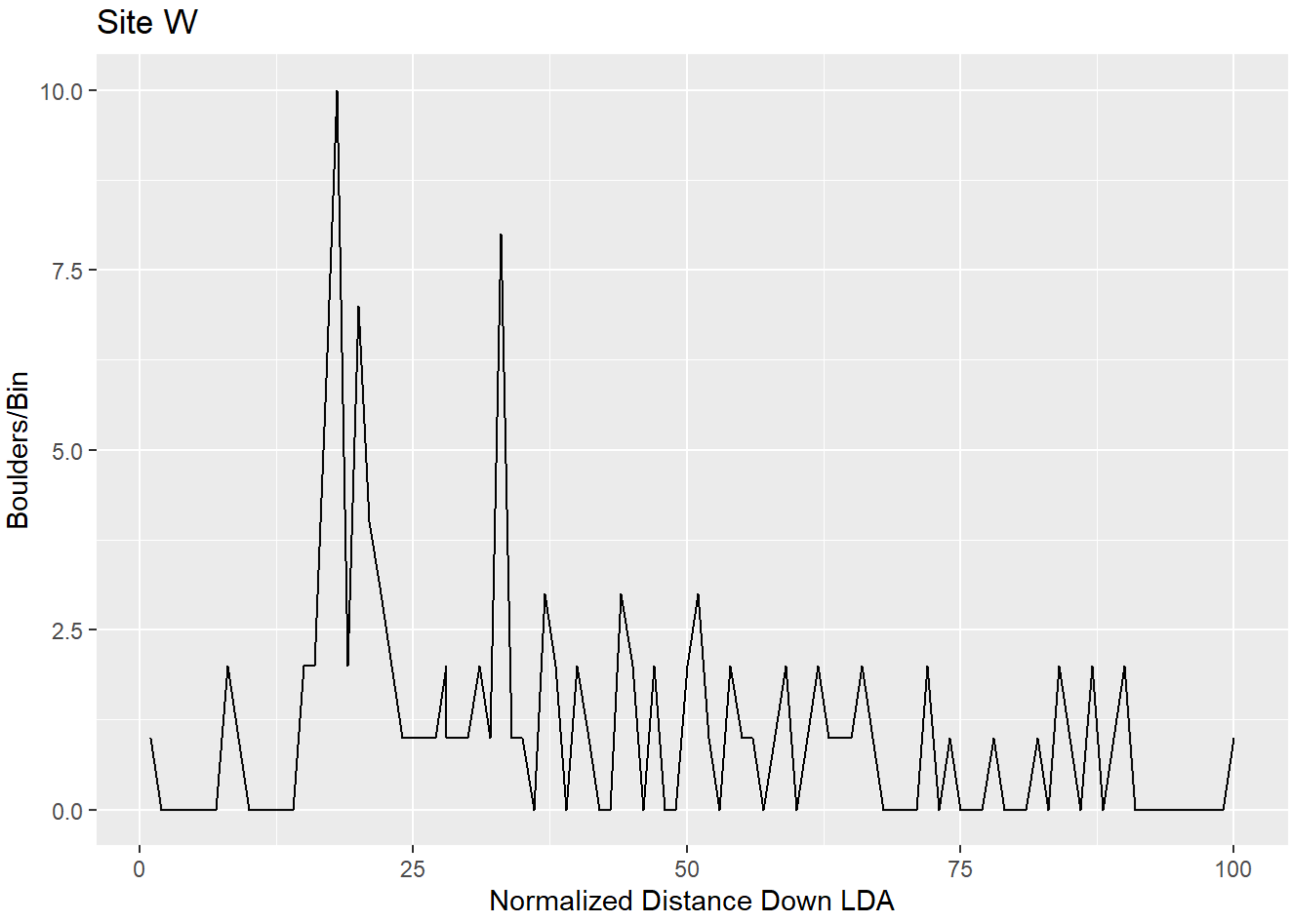
Site W
Boulders/Bin
0.0
2.5
5.0
7.5
10.0
0
25
50
75
100
Normalized Distance Down LDA

Site X

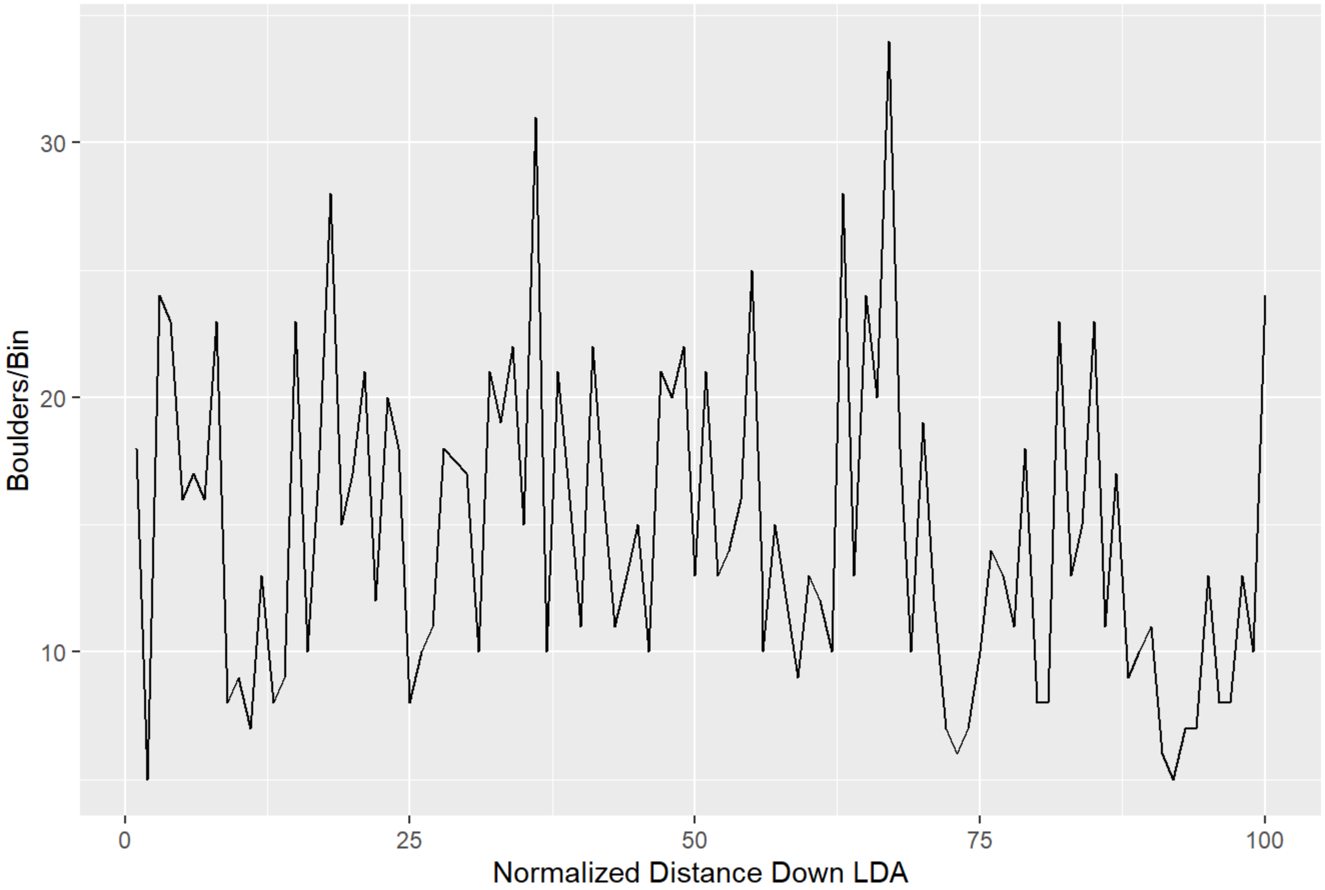
Boulders/Bin
30
20
10
0
25
50
75
100
Normalized Distance Down LDA

Site Y

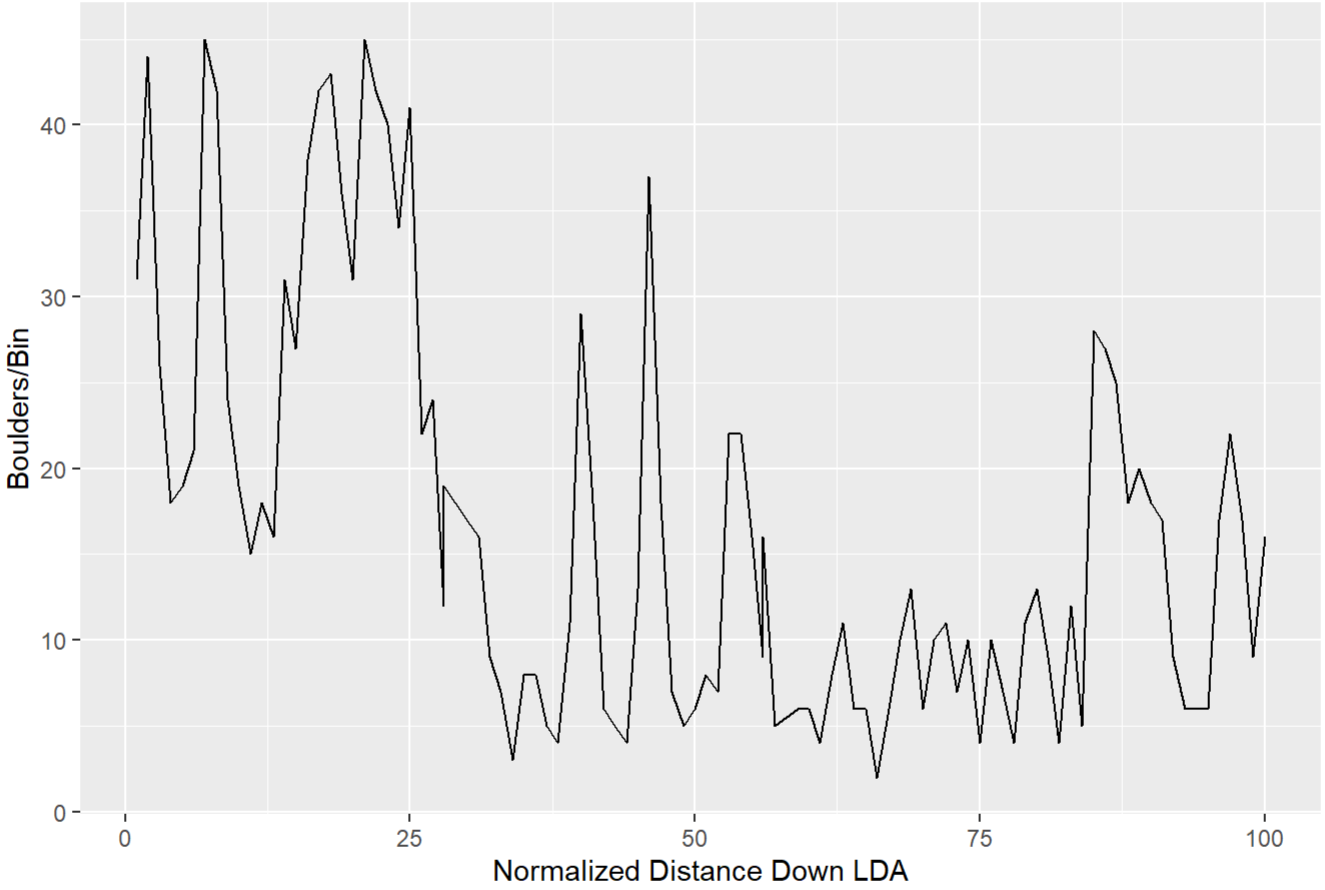
Boulders/Bin
40
30
20
10
0
0
25
50
75
100
Normalized Distance Down LDA

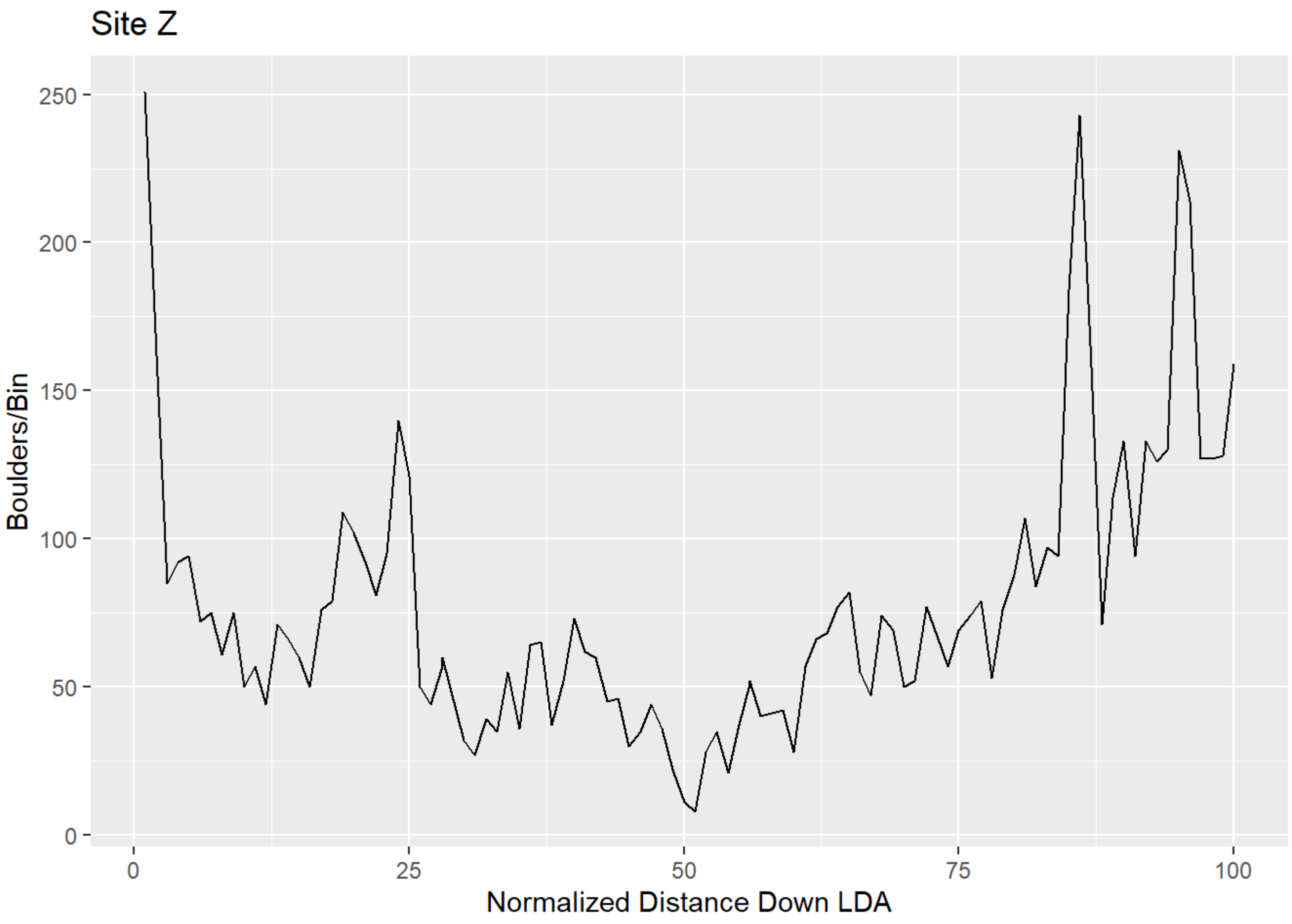
Site Z
Boulders/Bin
250
200
150
100
50
0
0
25
50
75
100
Normalized Distance Down LDA
